# Supplementary figures and images for: Lymphatic endothelium stimulates melanoma metastasis and invasion via MMP14-dependent Notch3 and β1-integrin activation (part 1 of 2)
Source: eLife. 2018 May 1;7:e32490. doi: 10.7554/eLife.32490 (PMC5929907; doi:10.7554/eLife.32490)

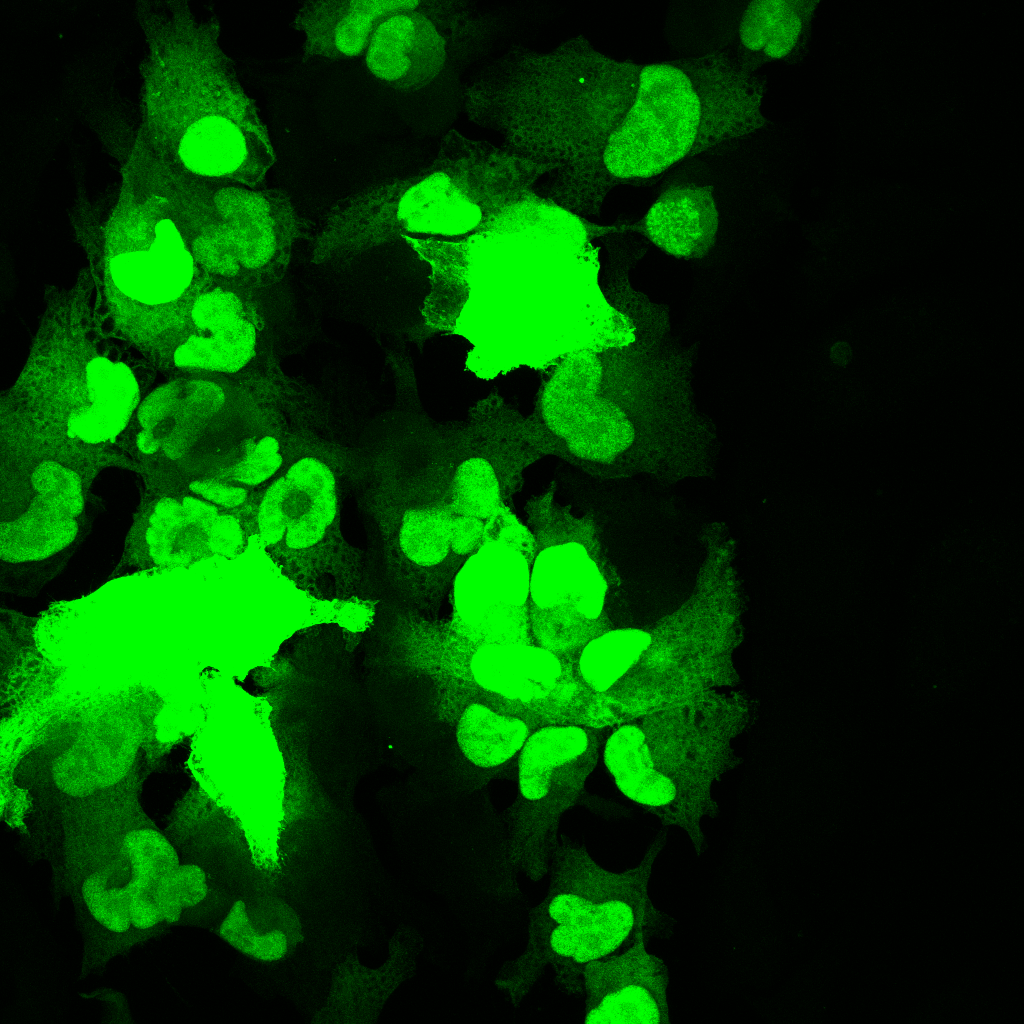

Supplement: Soure data 1. [file elife-32490-fig1.zip › Figure 2/Panel e/Bowes_LEC/LEC_Bowes_Notch3_594_Max_c1.tif]

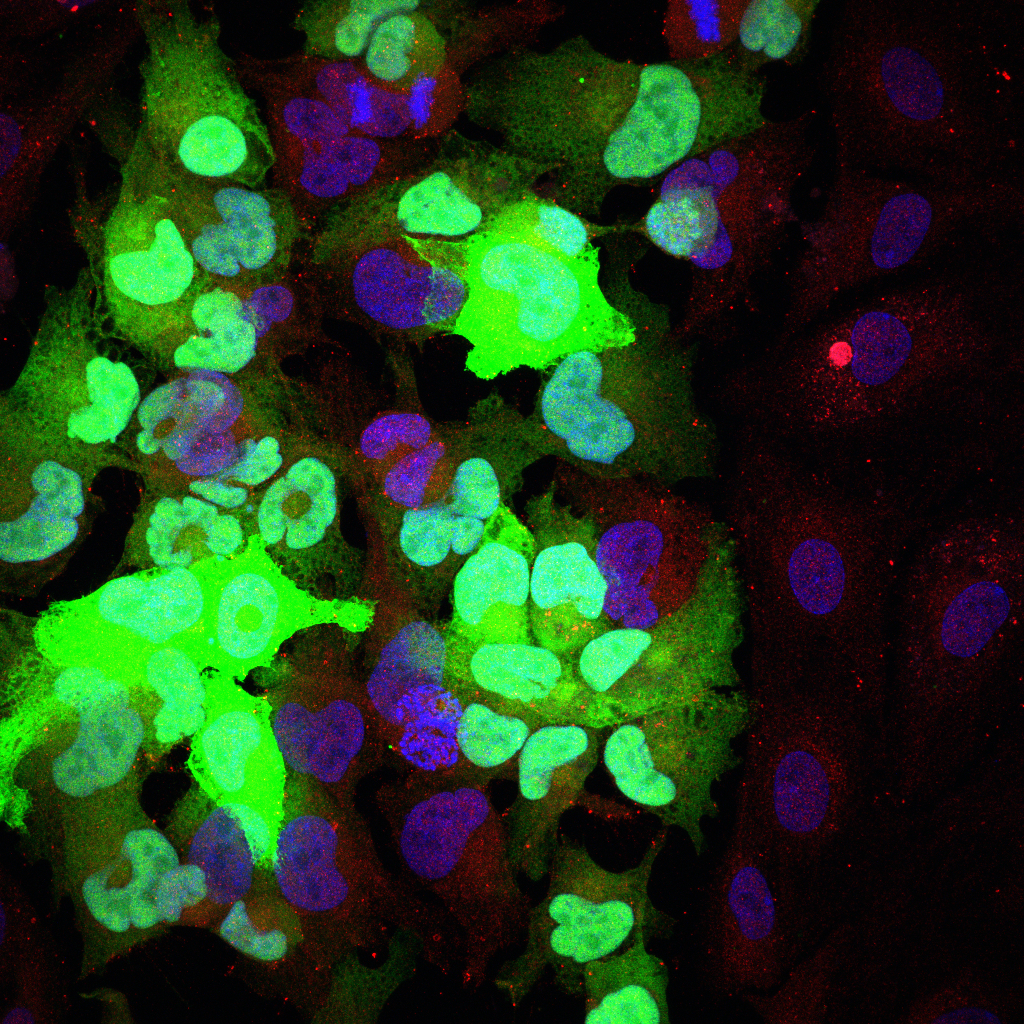

Supplement: Soure data 1. [file elife-32490-fig1.zip › Figure 2/Panel e/Bowes_LEC/LEC_Bowes_Notch3_594_Max_c1+2+3.tif]

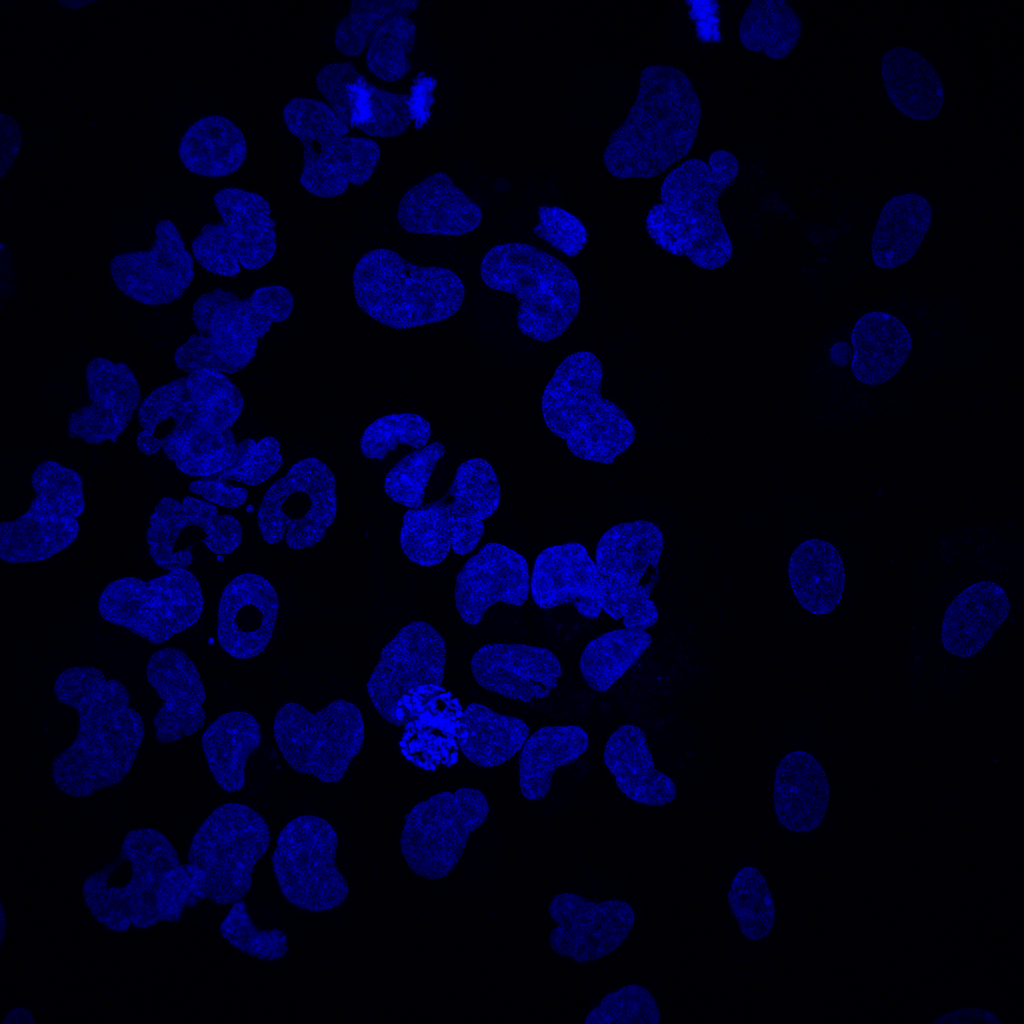

Supplement: Soure data 1. [file elife-32490-fig1.zip › Figure 2/Panel e/Bowes_LEC/LEC_Bowes_Notch3_594_Max_c2.tif]

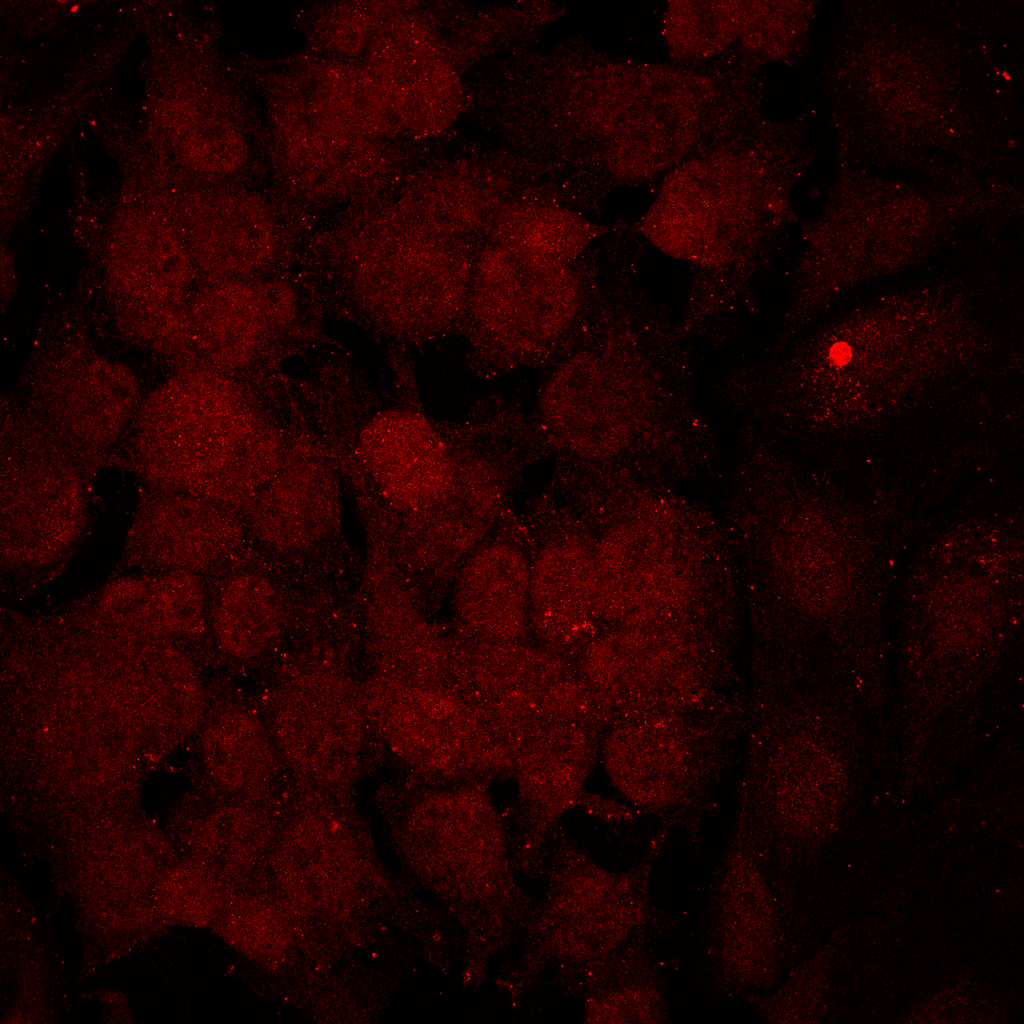

Supplement: Soure data 1. [file elife-32490-fig1.zip › Figure 2/Panel e/Bowes_LEC/LEC_Bowes_Notch3_594_Max_c3.tif]

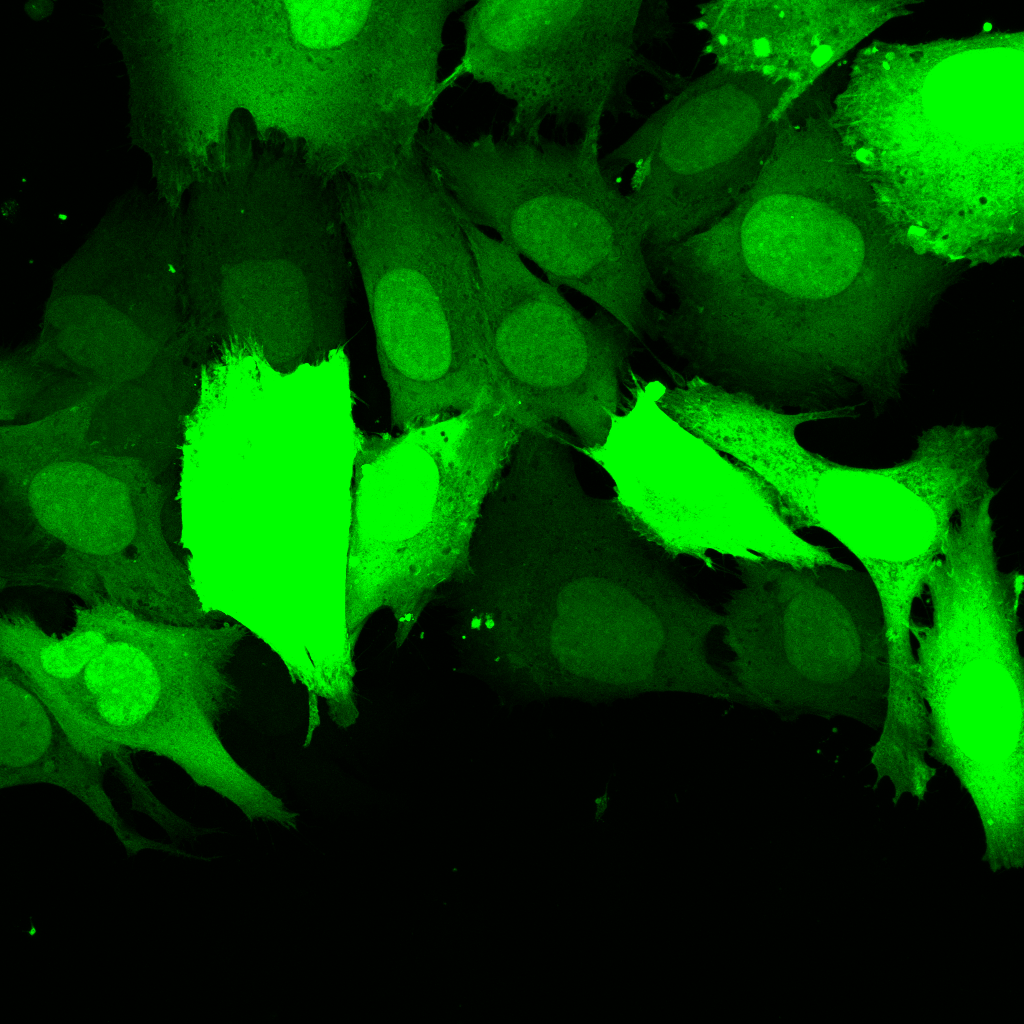

Supplement: Soure data 1. [file elife-32490-fig1.zip › Figure 2/Panel e/WM165/LEC_WM165_Notch3_594_Max_c1.tif]

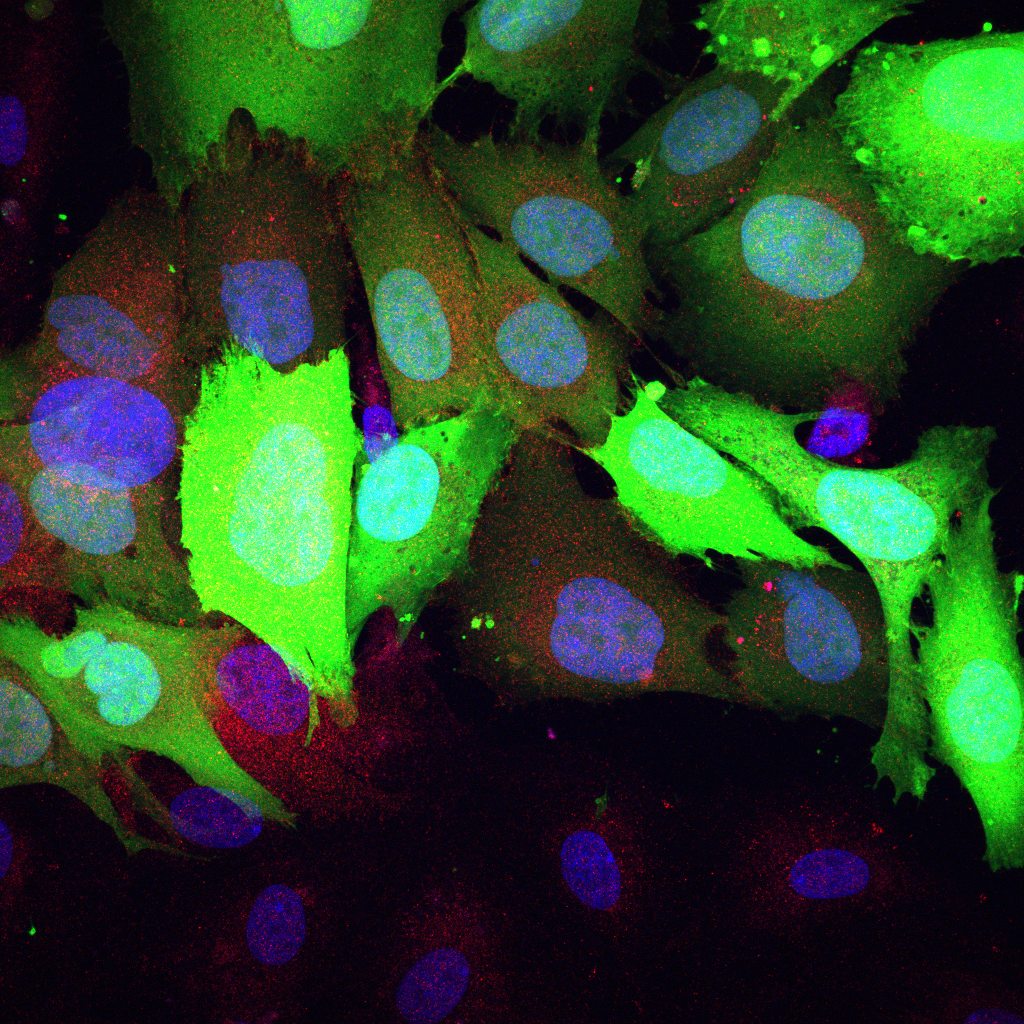

Supplement: Soure data 1. [file elife-32490-fig1.zip › Figure 2/Panel e/WM165/LEC_WM165_Notch3_594_Max_c1+2+3.tif]

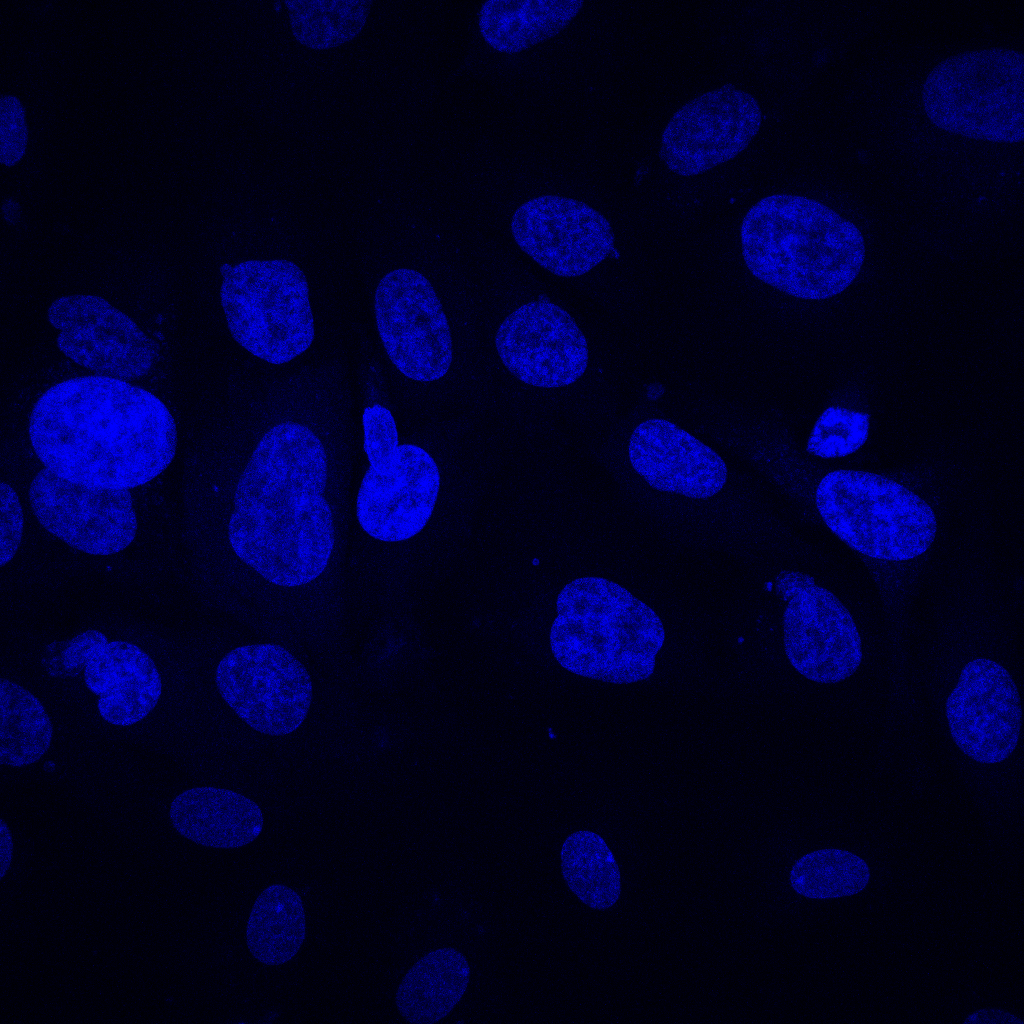

Supplement: Soure data 1. [file elife-32490-fig1.zip › Figure 2/Panel e/WM165/LEC_WM165_Notch3_594_Max_c2.tif]

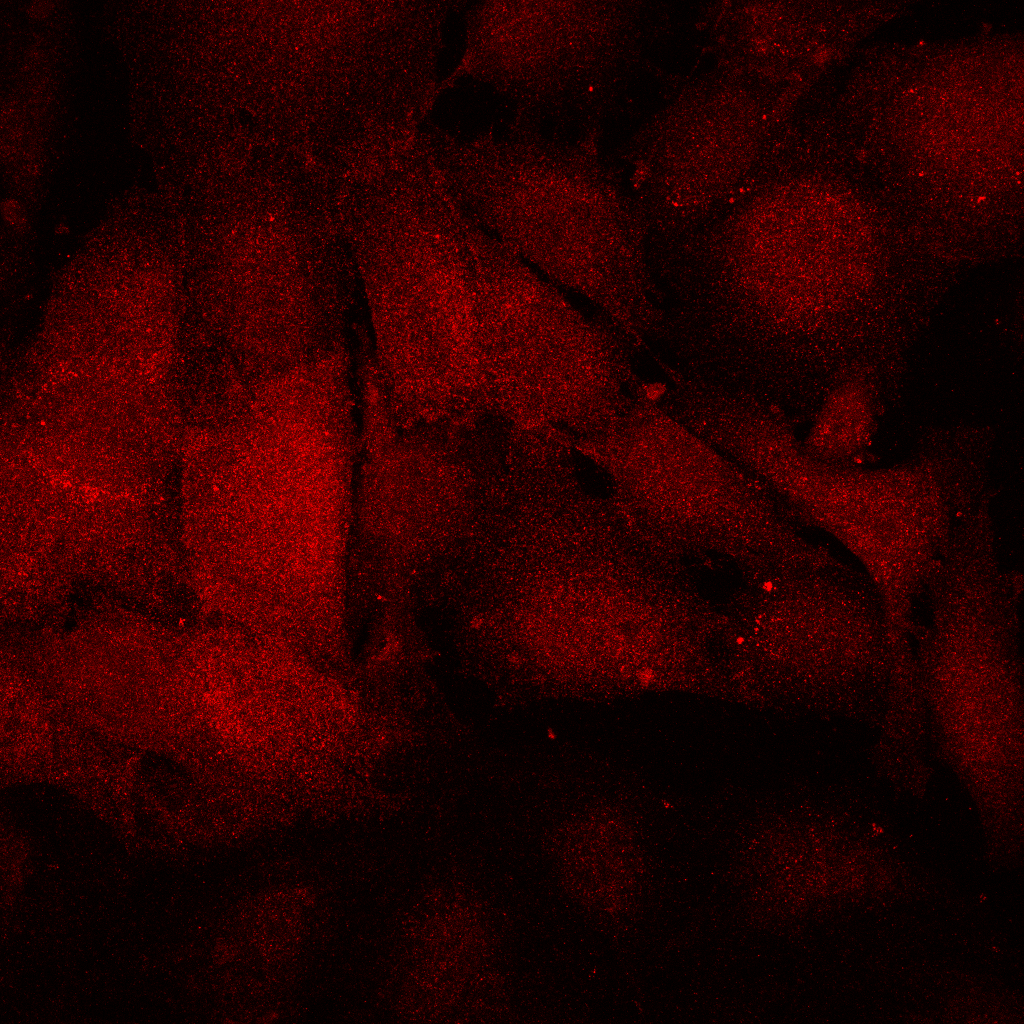

Supplement: Soure data 1. [file elife-32490-fig1.zip › Figure 2/Panel e/WM165/LEC_WM165_Notch3_594_Max_c3.tif]

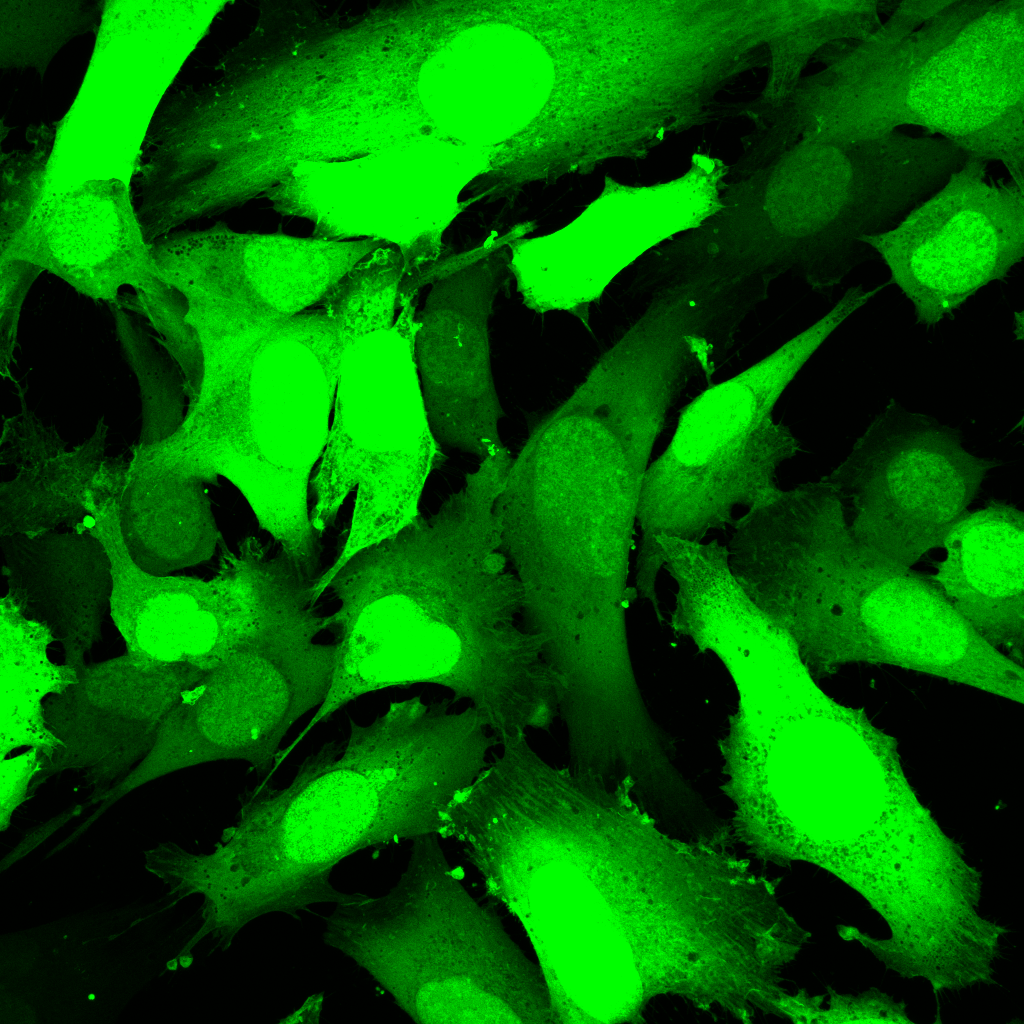

Supplement: Soure data 1. [file elife-32490-fig1.zip › Figure 2/Panel e/WM165_LEC/WM165_Notch3_594_Max_c1.tif]

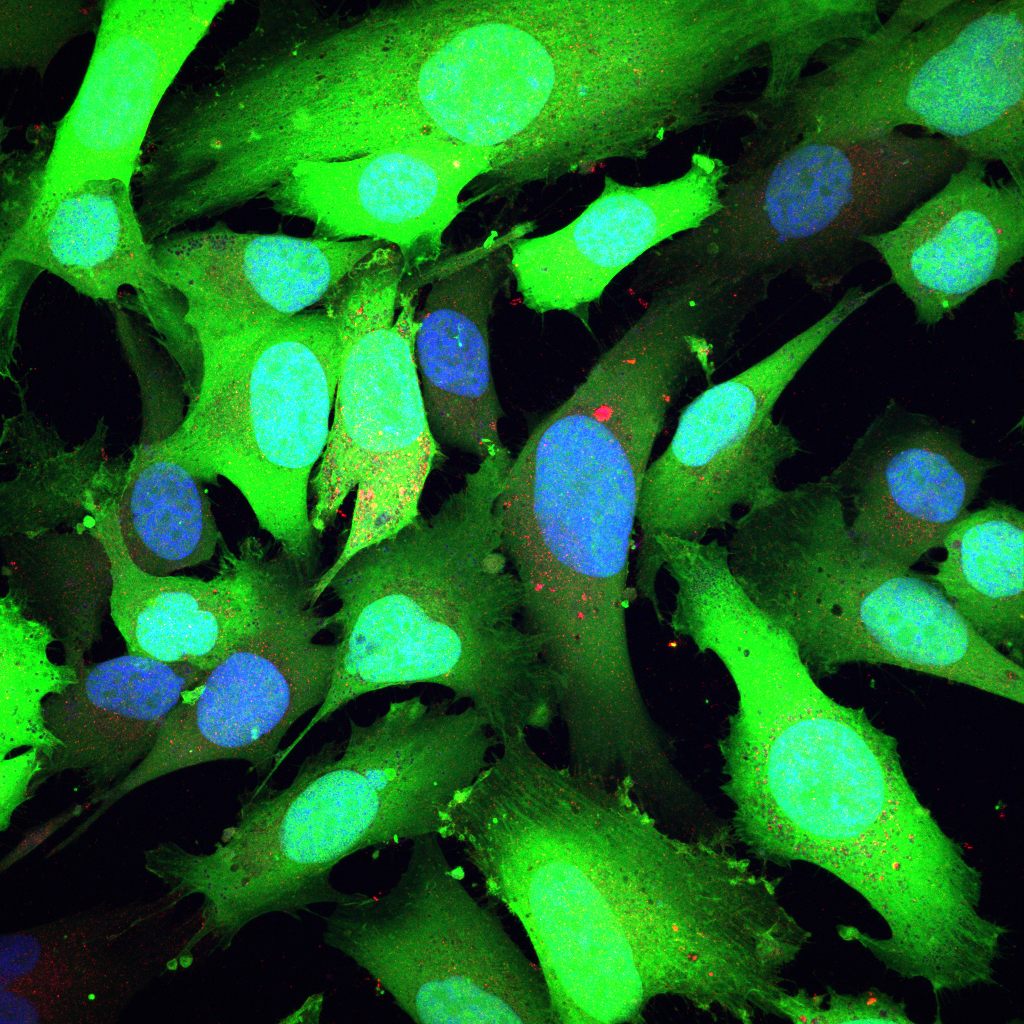

Supplement: Soure data 1. [file elife-32490-fig1.zip › Figure 2/Panel e/WM165_LEC/WM165_Notch3_594_Max_c1+2+3.tif]

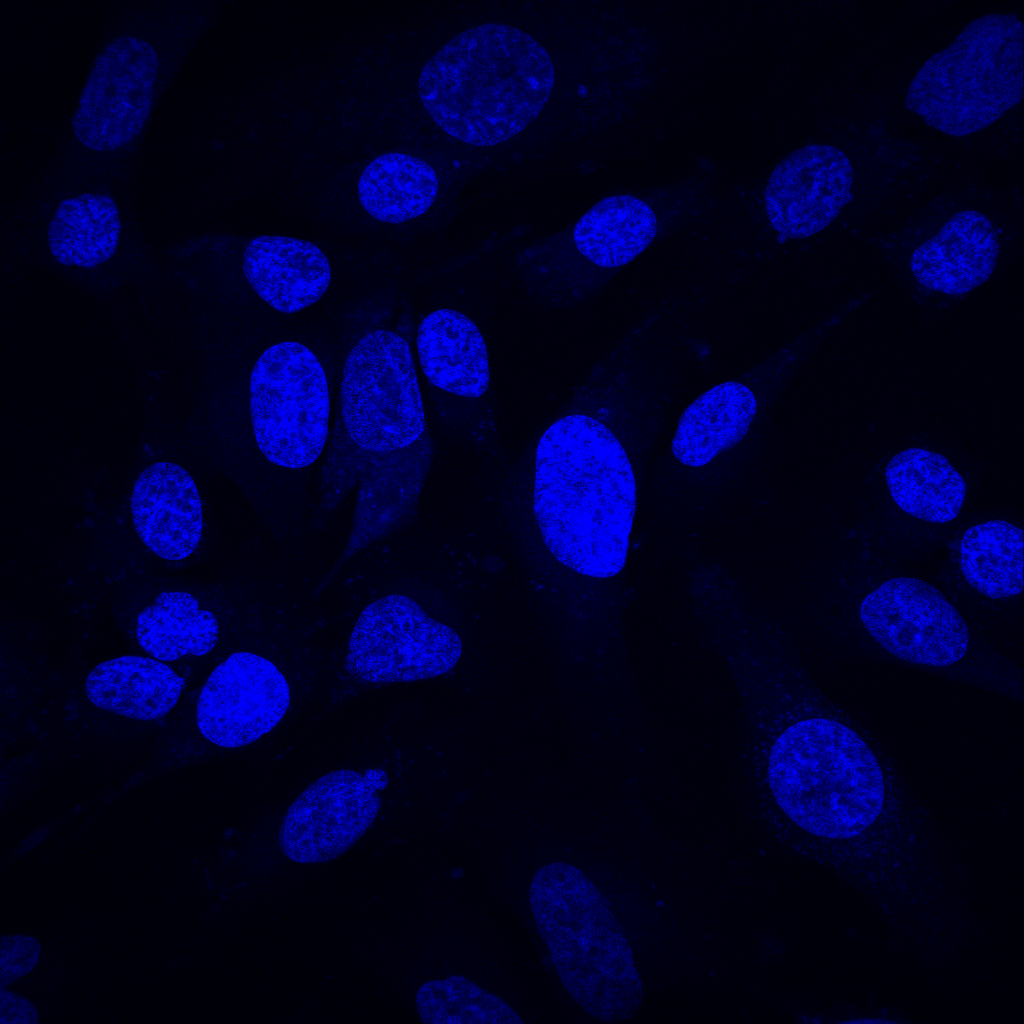

Supplement: Soure data 1. [file elife-32490-fig1.zip › Figure 2/Panel e/WM165_LEC/WM165_Notch3_594_Max_c2.tif]

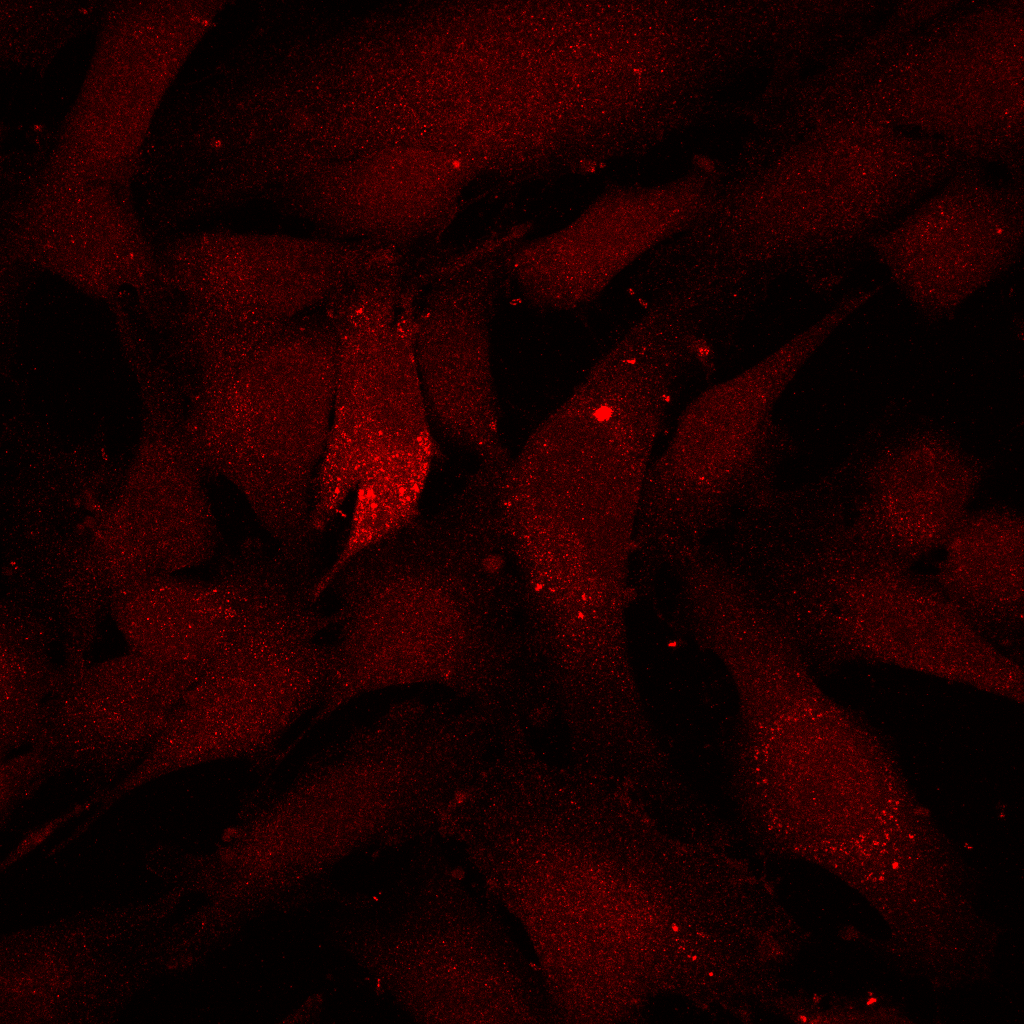

Supplement: Soure data 1. [file elife-32490-fig1.zip › Figure 2/Panel e/WM165_LEC/WM165_Notch3_594_Max_c3.tif]

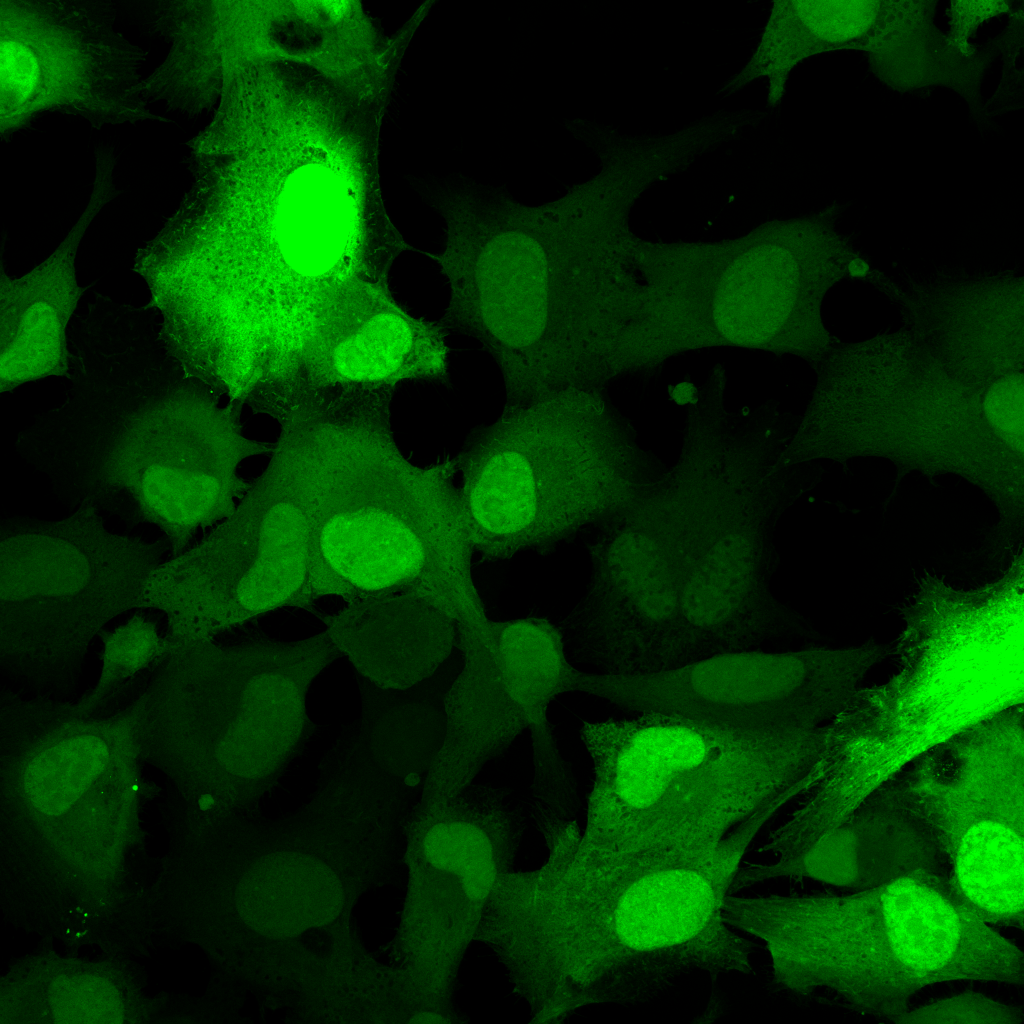

Supplement: Soure data 1. [file elife-32490-fig1.zip › Figure 2/Panel e/WM793/WM793_Notch3_594_Max_c1.tif]

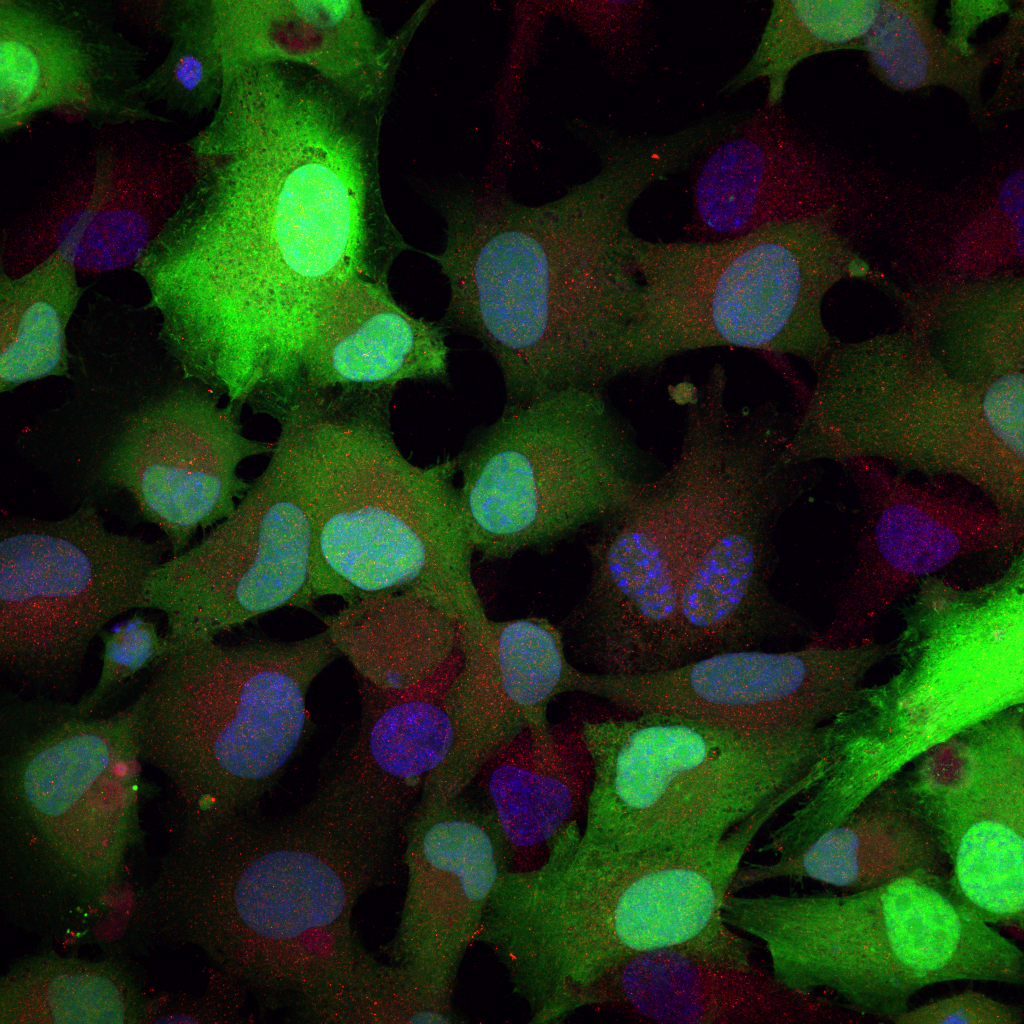

Supplement: Soure data 1. [file elife-32490-fig1.zip › Figure 2/Panel e/WM793/WM793_Notch3_594_Max_c1+2+3.tif]

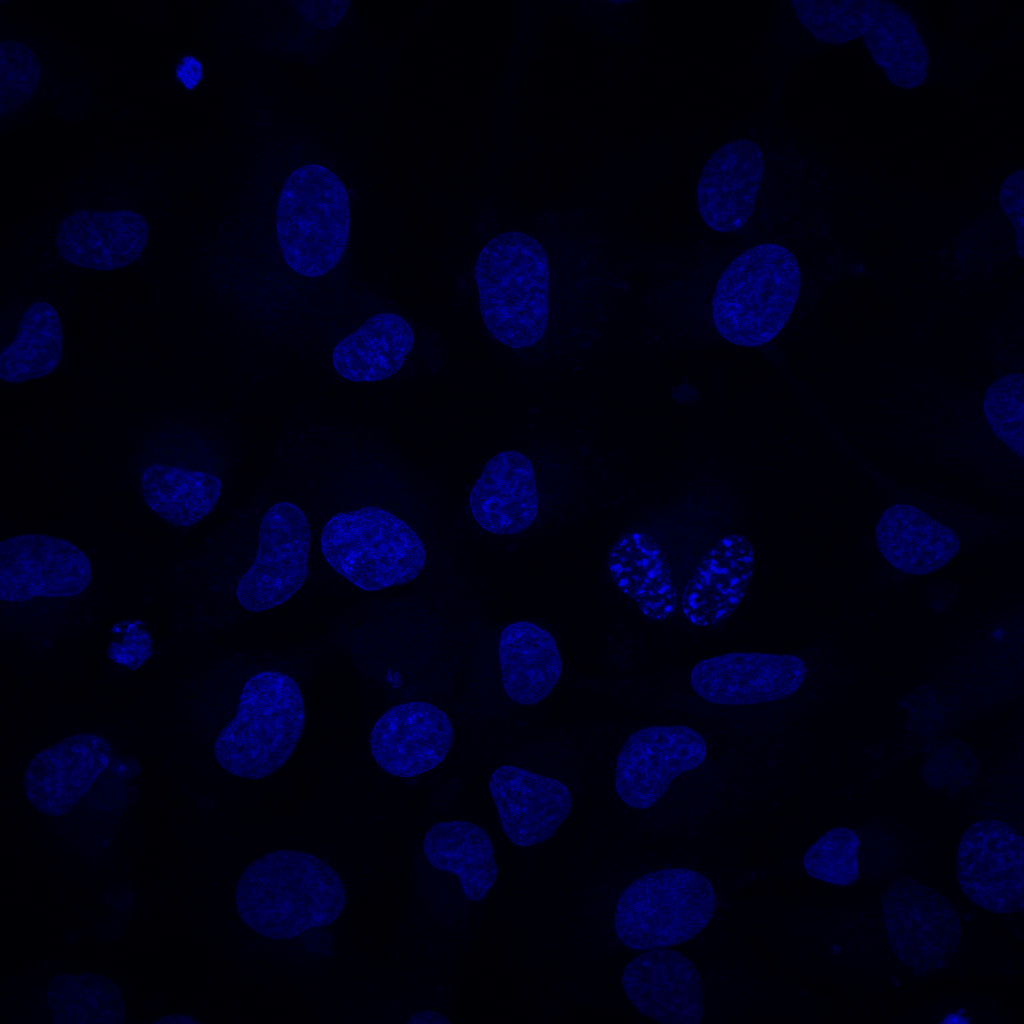

Supplement: Soure data 1. [file elife-32490-fig1.zip › Figure 2/Panel e/WM793/WM793_Notch3_594_Max_c2.tif]

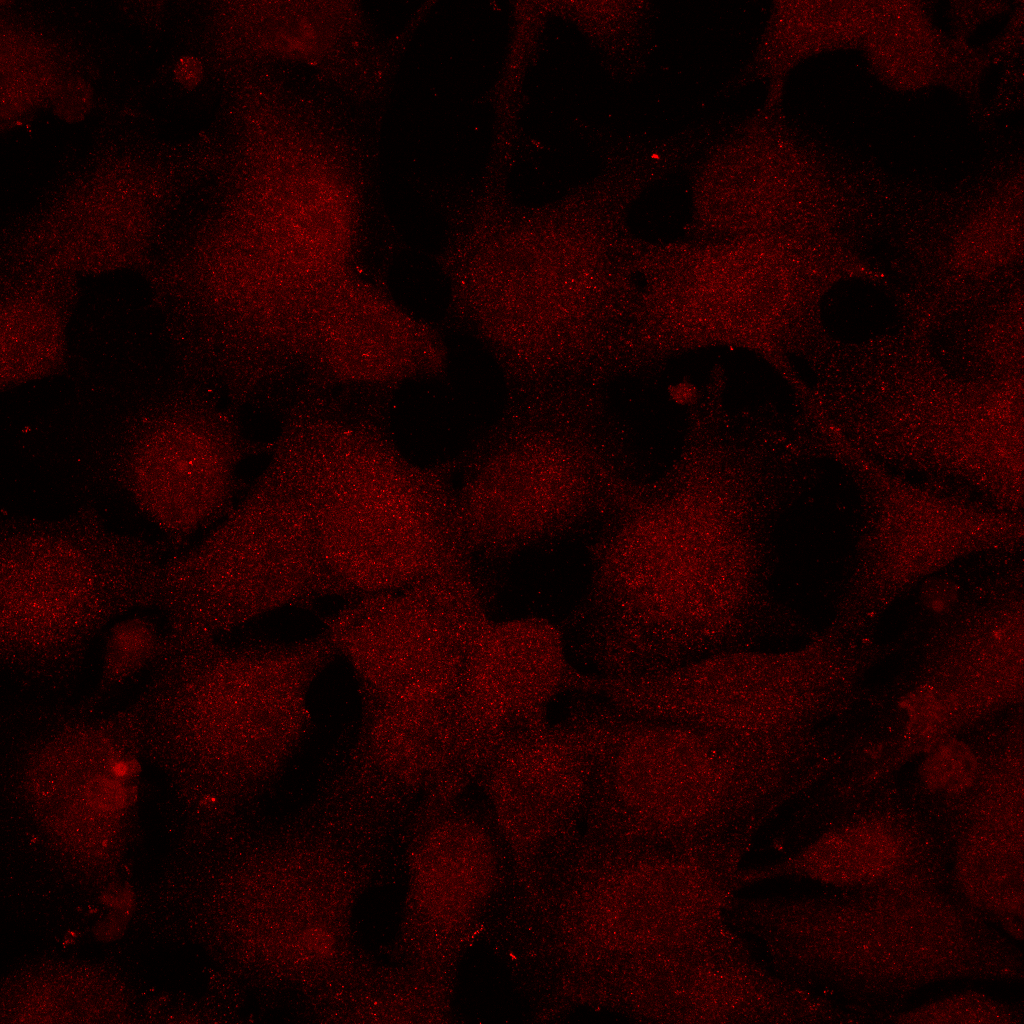

Supplement: Soure data 1. [file elife-32490-fig1.zip › Figure 2/Panel e/WM793/WM793_Notch3_594_Max_c3.tif]

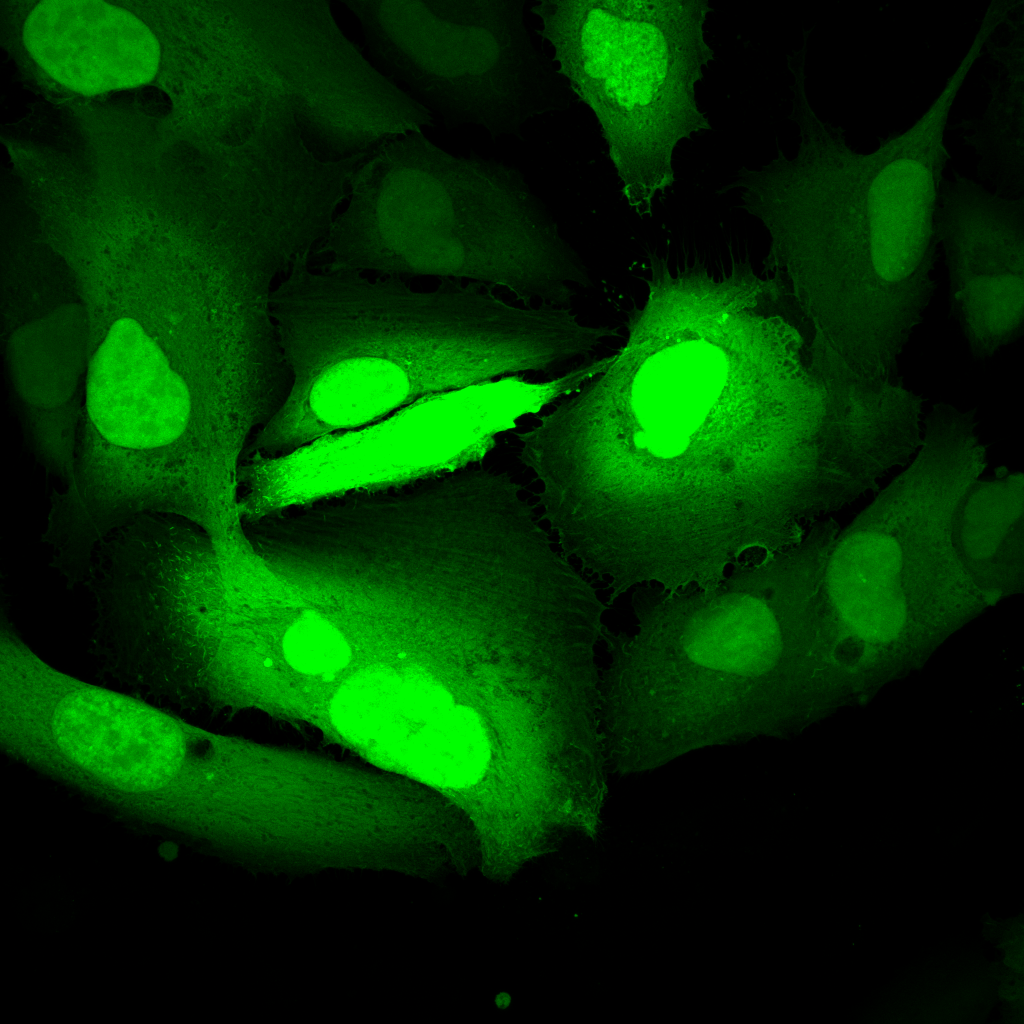

Supplement: Soure data 1. [file elife-32490-fig1.zip › Figure 2/Panel e/WM793_LEC/LEC_WM793_Notch3_594_Max_c1.tif]

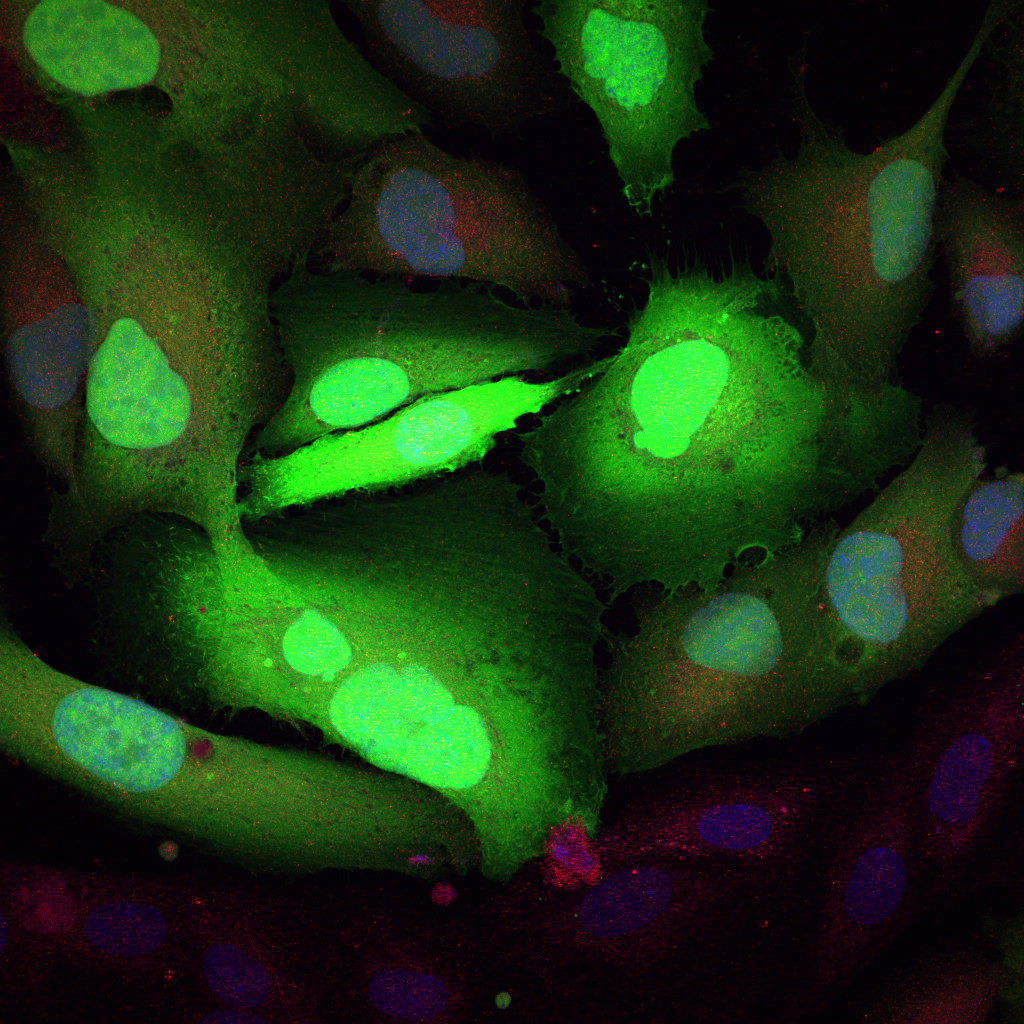

Supplement: Soure data 1. [file elife-32490-fig1.zip › Figure 2/Panel e/WM793_LEC/LEC_WM793_Notch3_594_Max_c1+2+3.tif]

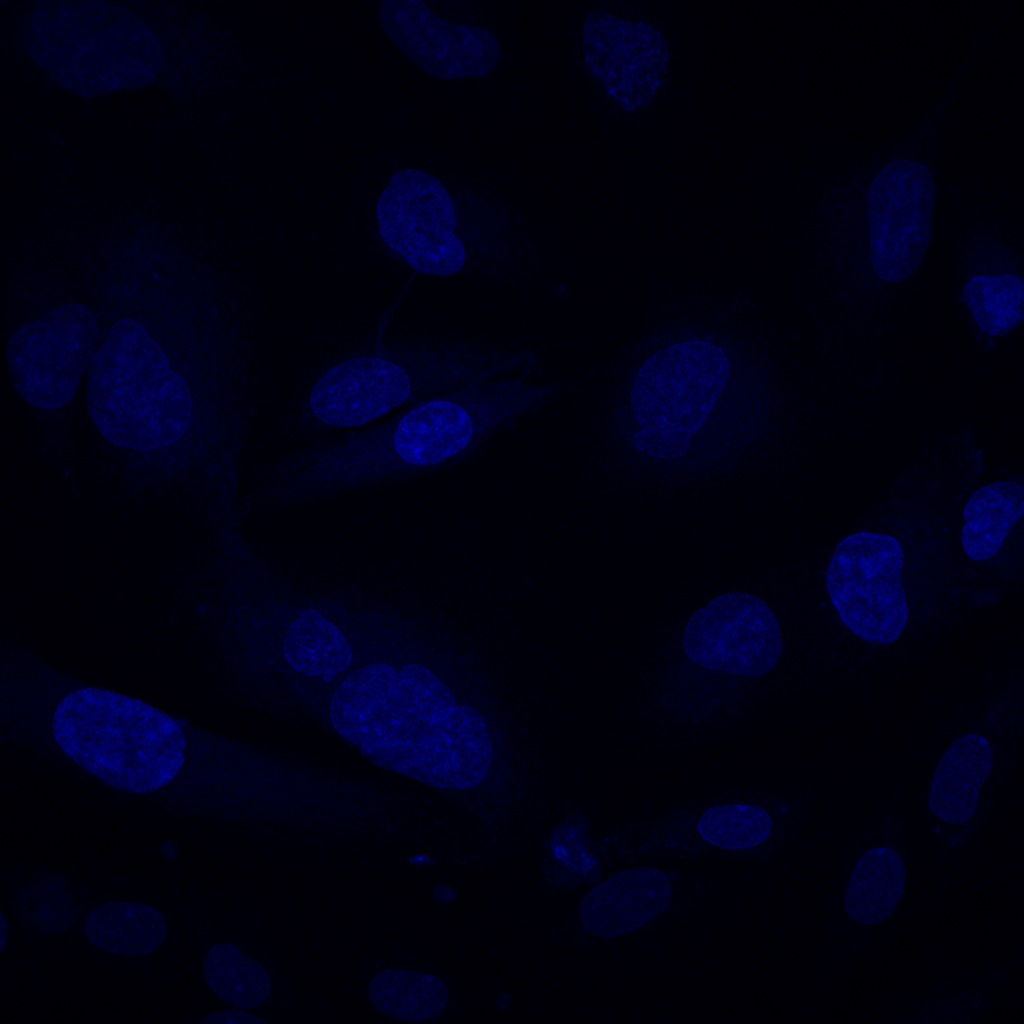

Supplement: Soure data 1. [file elife-32490-fig1.zip › Figure 2/Panel e/WM793_LEC/LEC_WM793_Notch3_594_Max_c2.tif]

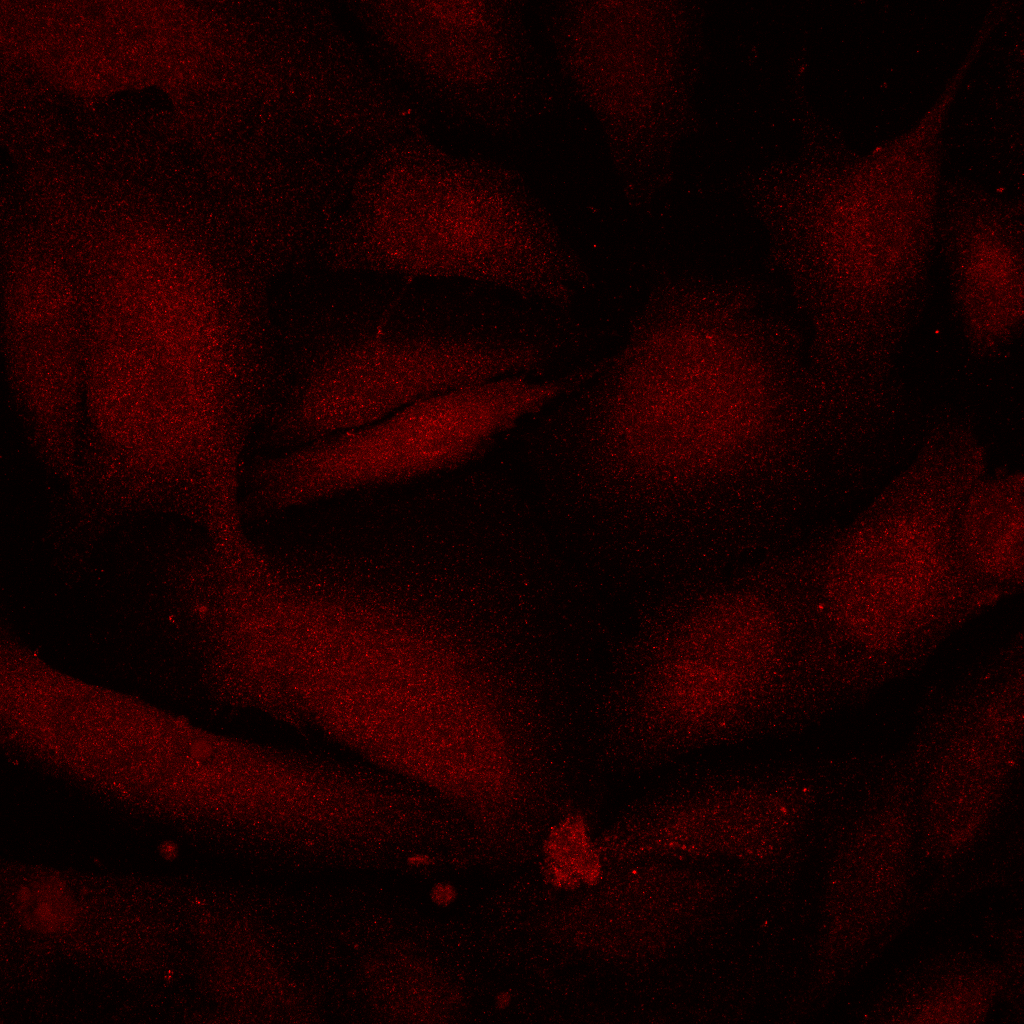

Supplement: Soure data 1. [file elife-32490-fig1.zip › Figure 2/Panel e/WM793_LEC/LEC_WM793_Notch3_594_Max_c3.tif]

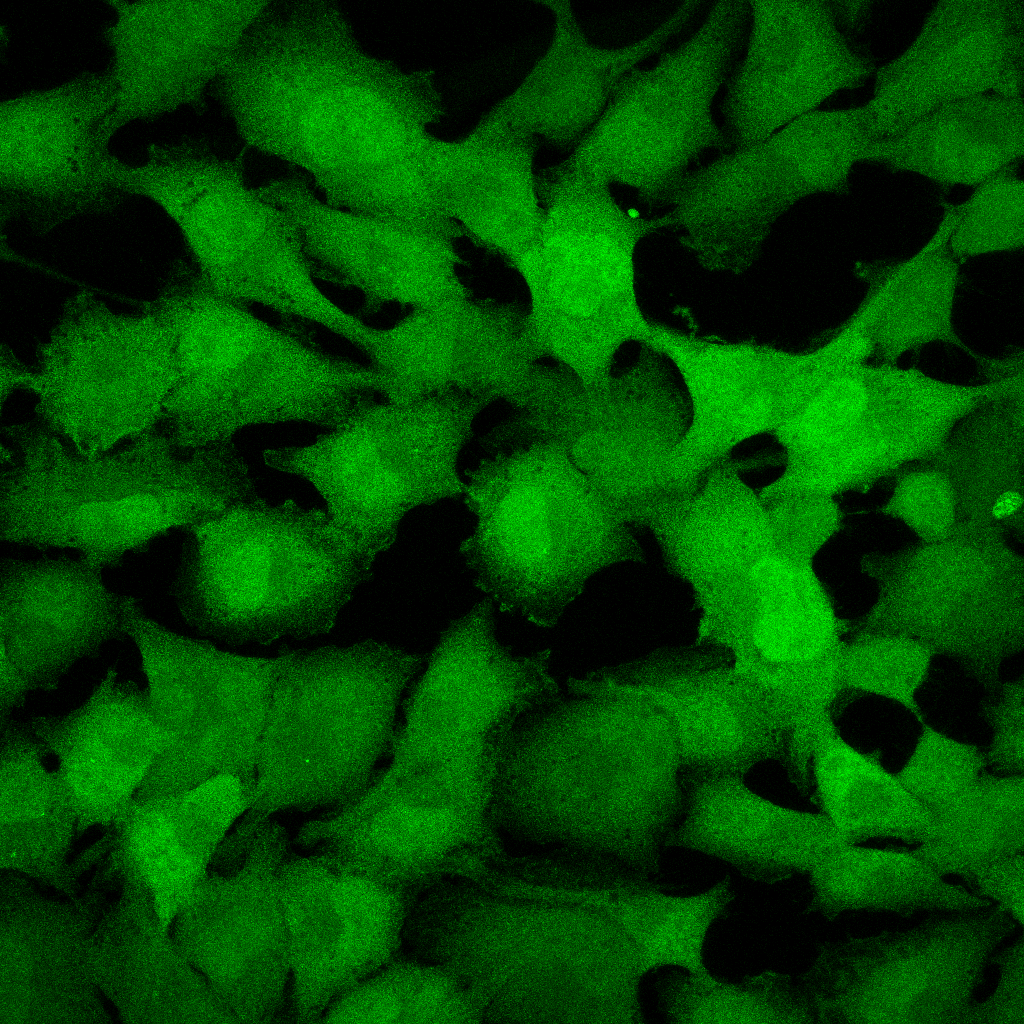

Supplement: Soure data 1. [file elife-32490-fig1.zip › Figure 2/Panel e/WM852/WM852_Notch3_594_Max_c1.tif]

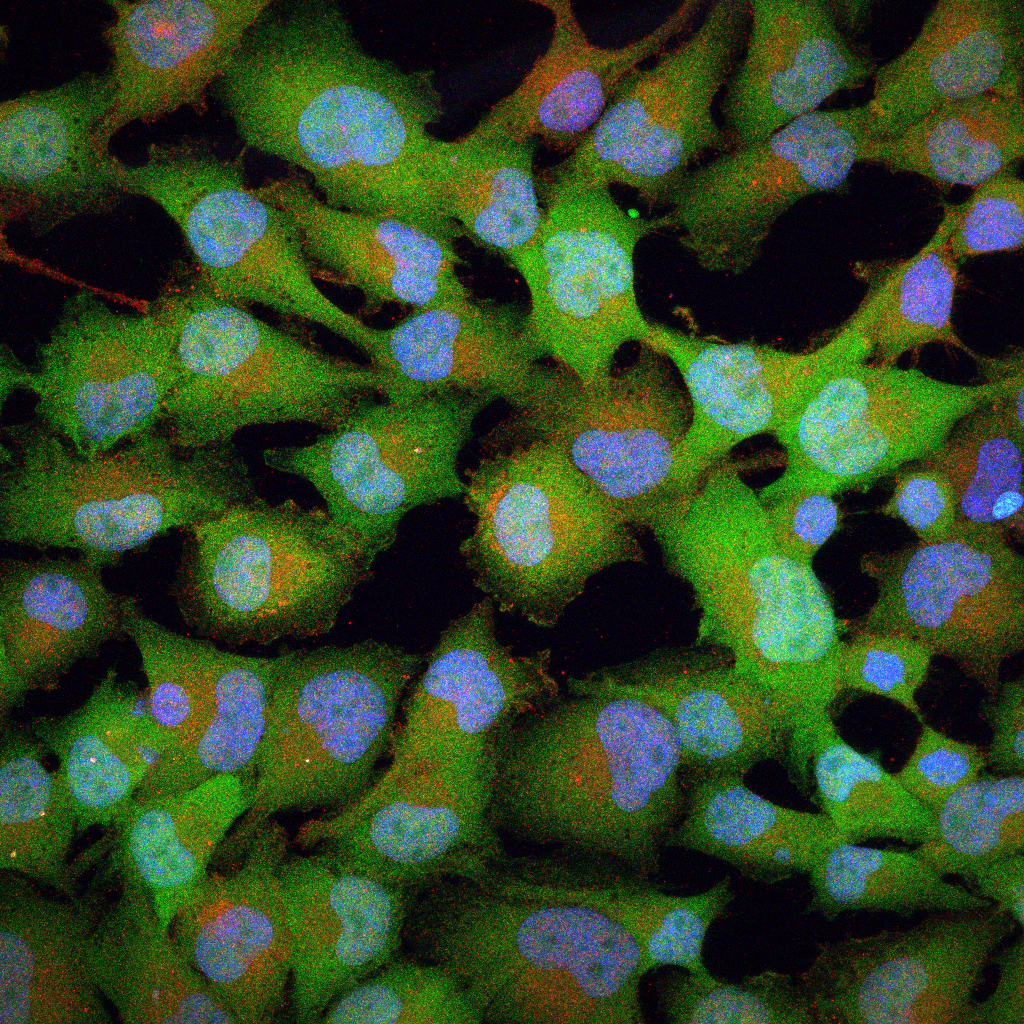

Supplement: Soure data 1. [file elife-32490-fig1.zip › Figure 2/Panel e/WM852/WM852_Notch3_594_Max_c1+2+3.tif]

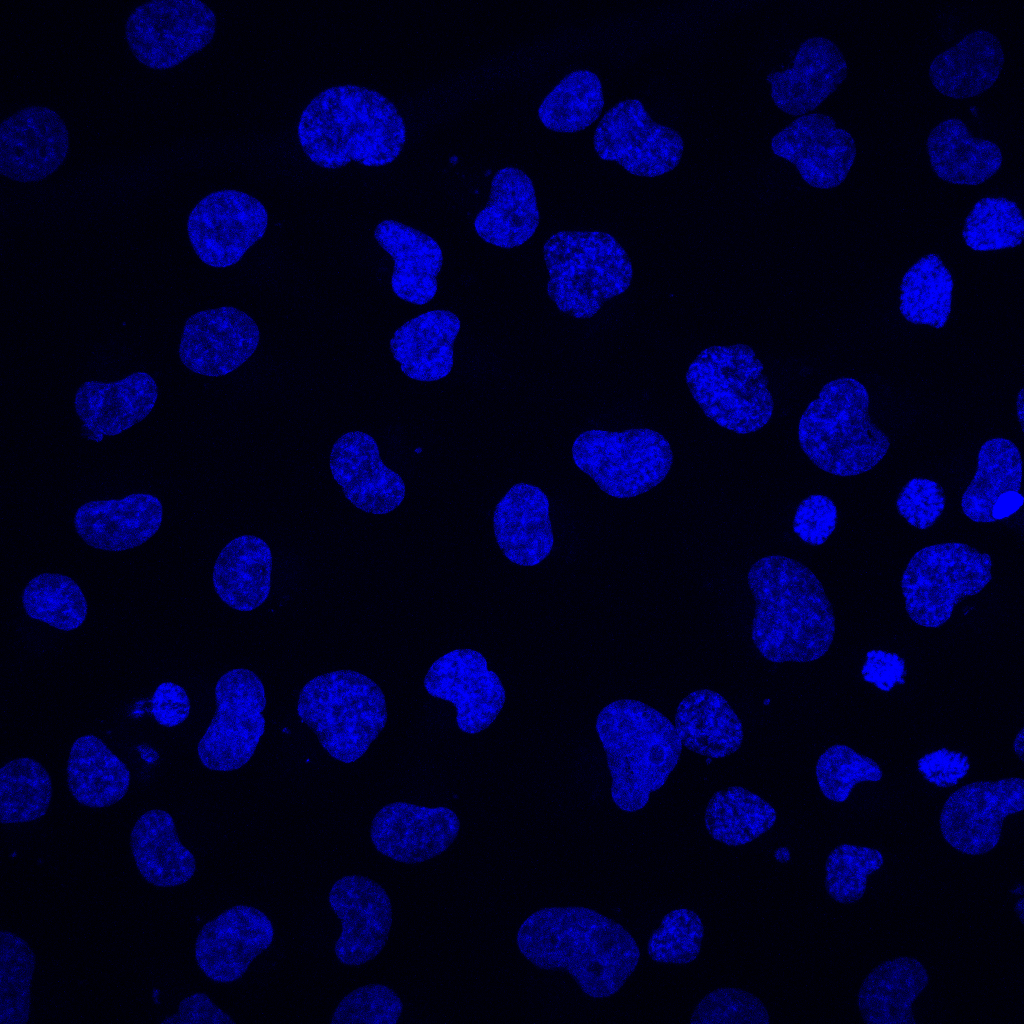

Supplement: Soure data 1. [file elife-32490-fig1.zip › Figure 2/Panel e/WM852/WM852_Notch3_594_Max_c2.tif]

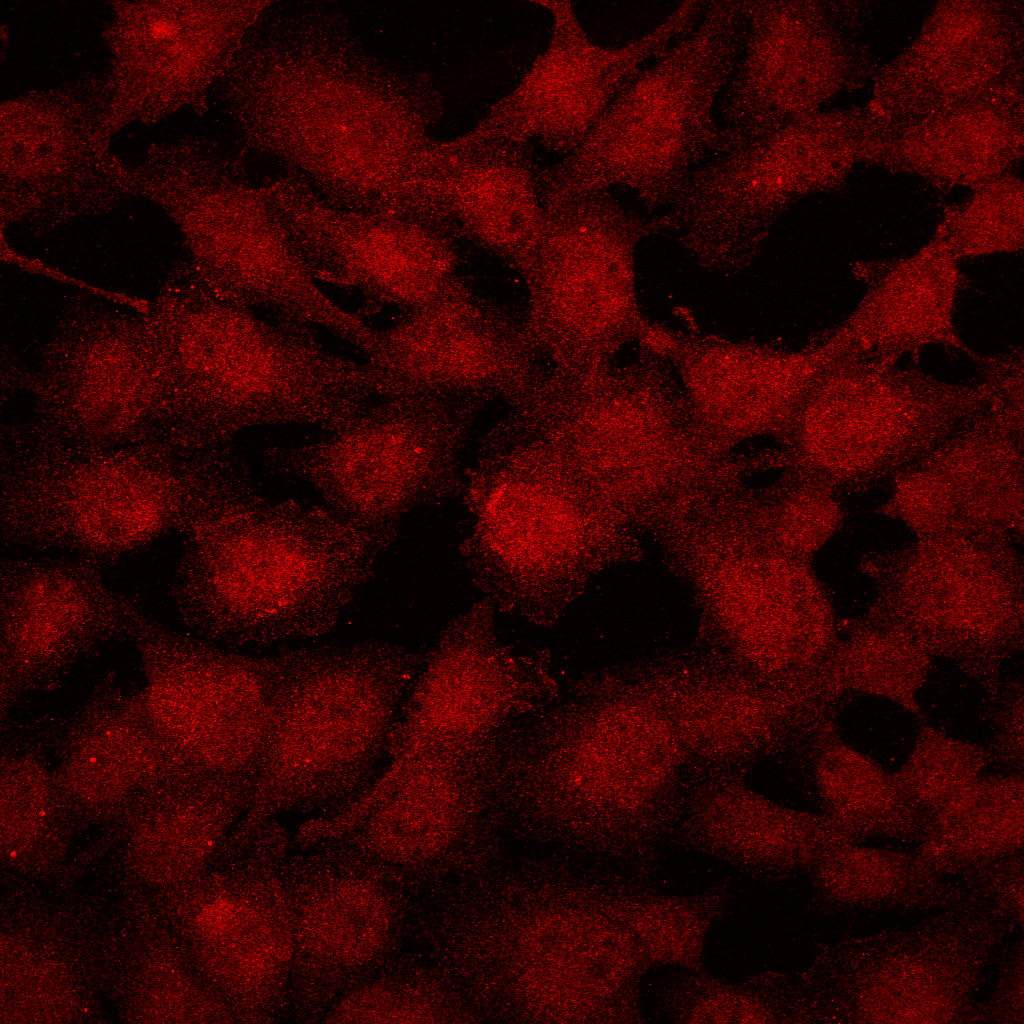

Supplement: Soure data 1. [file elife-32490-fig1.zip › Figure 2/Panel e/WM852/WM852_Notch3_594_Max_c3.tif]

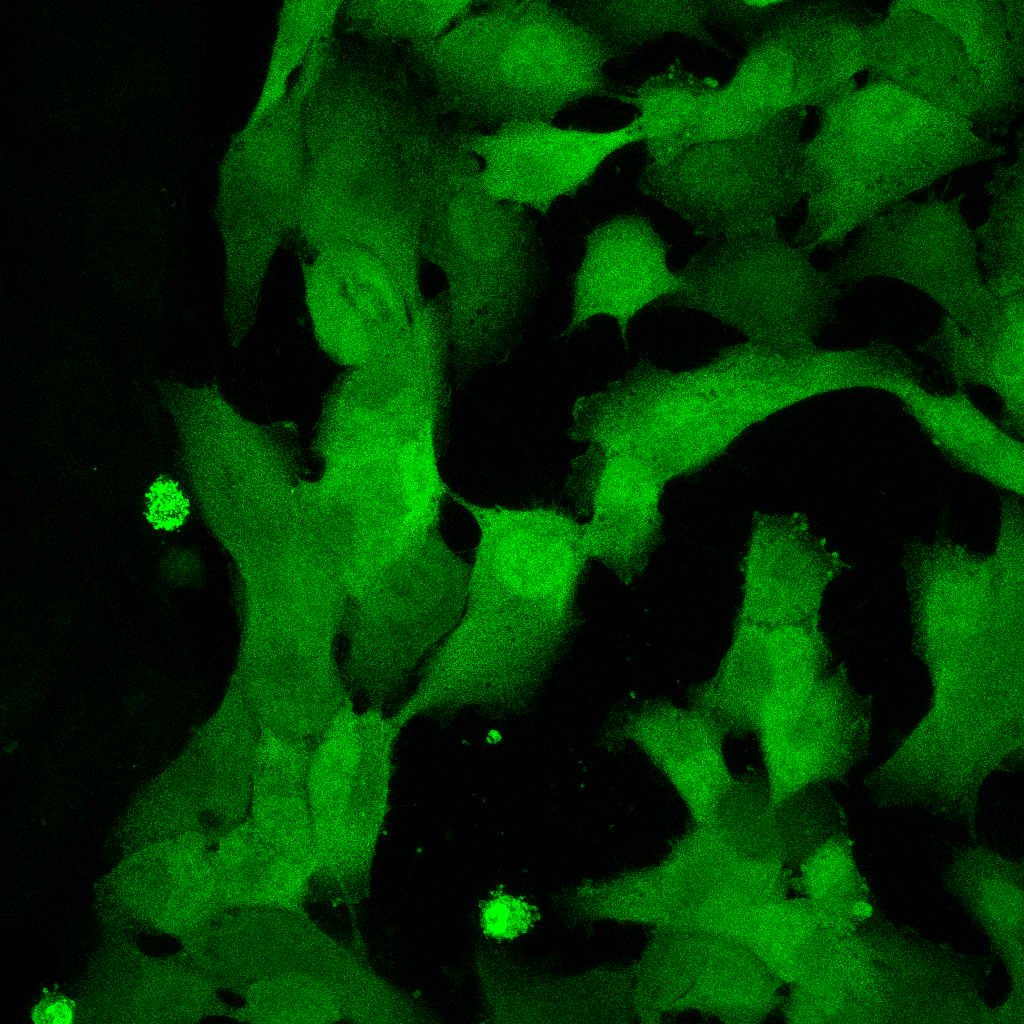

Supplement: Soure data 1. [file elife-32490-fig1.zip › Figure 2/Panel e/WM852_LEC/LEC_WM852_Notch3_594_Max_c1.tif]

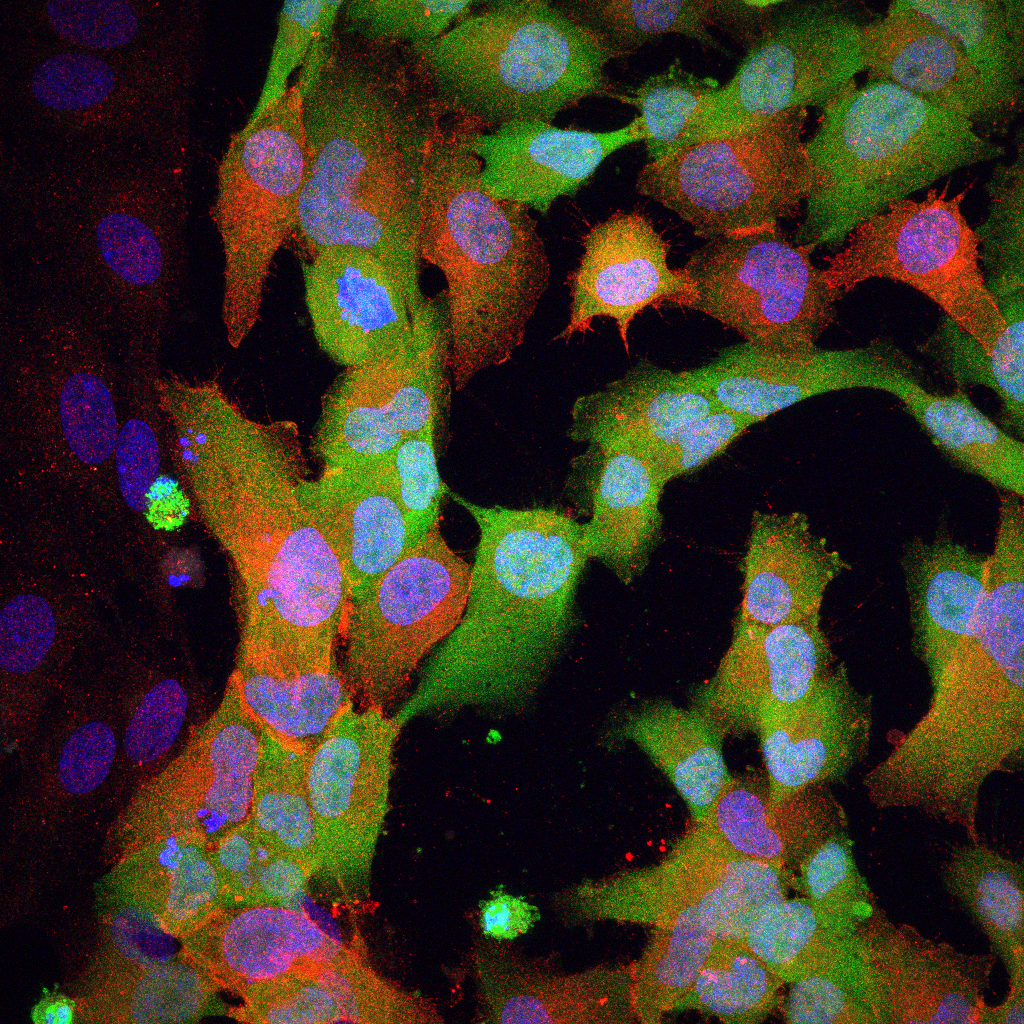

Supplement: Soure data 1. [file elife-32490-fig1.zip › Figure 2/Panel e/WM852_LEC/LEC_WM852_Notch3_594_Max_c1+2+3.tif]

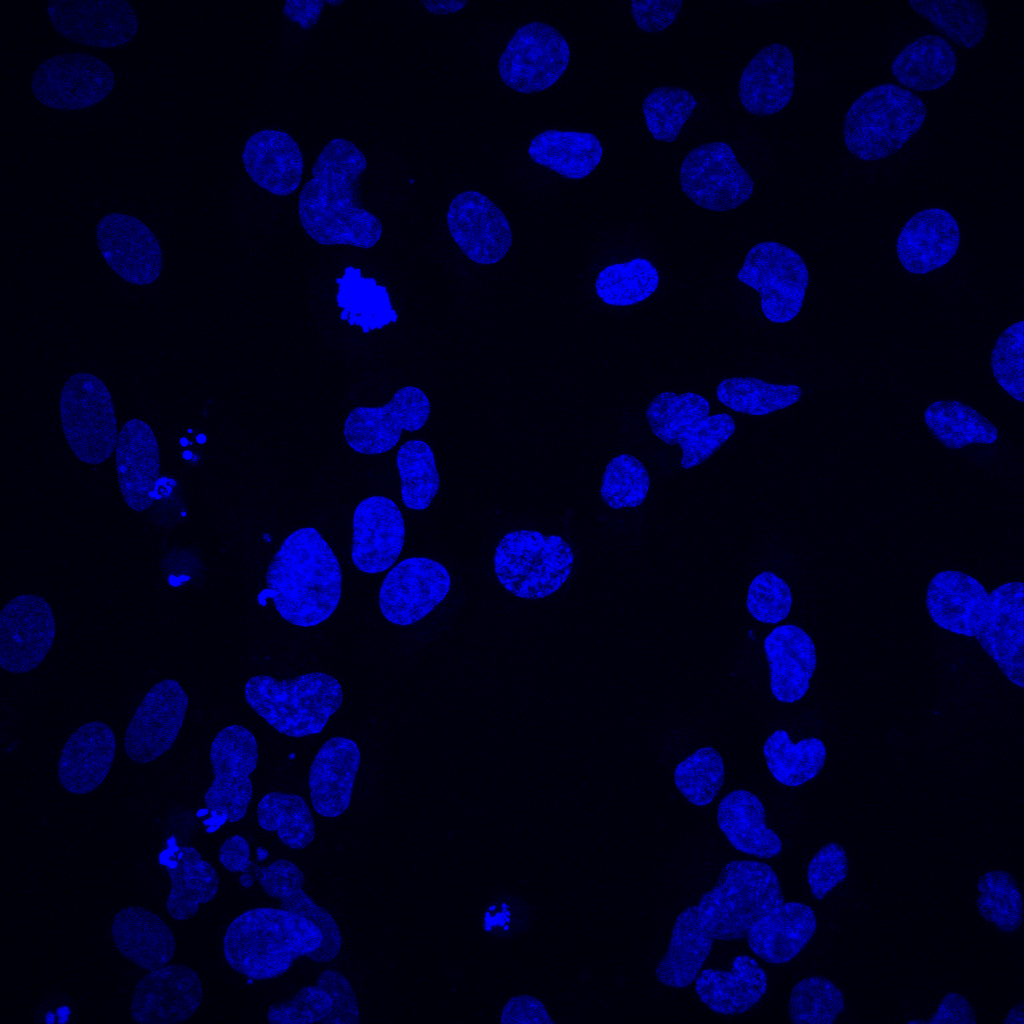

Supplement: Soure data 1. [file elife-32490-fig1.zip › Figure 2/Panel e/WM852_LEC/LEC_WM852_Notch3_594_Max_c2.tif]

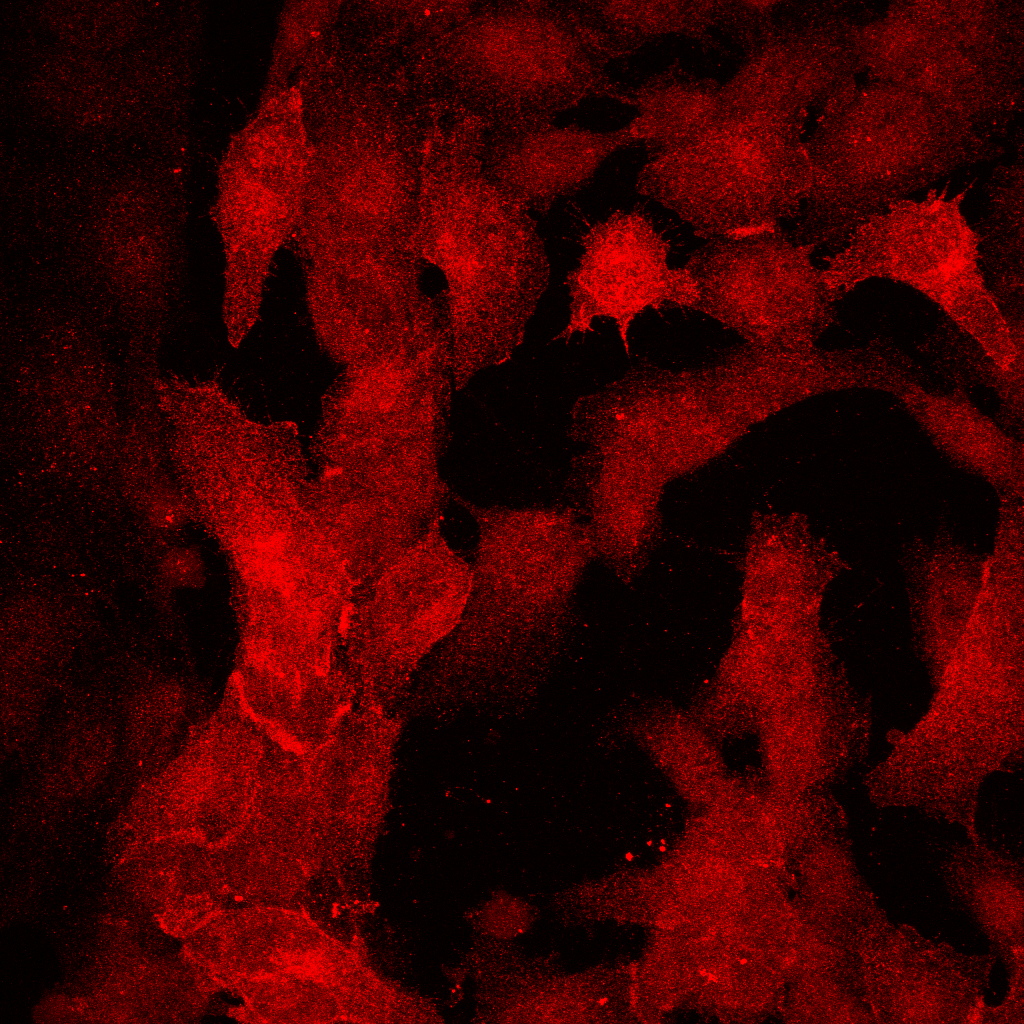

Supplement: Soure data 1. [file elife-32490-fig1.zip › Figure 2/Panel e/WM852_LEC/LEC_WM852_Notch3_594_Max_c3.tif]

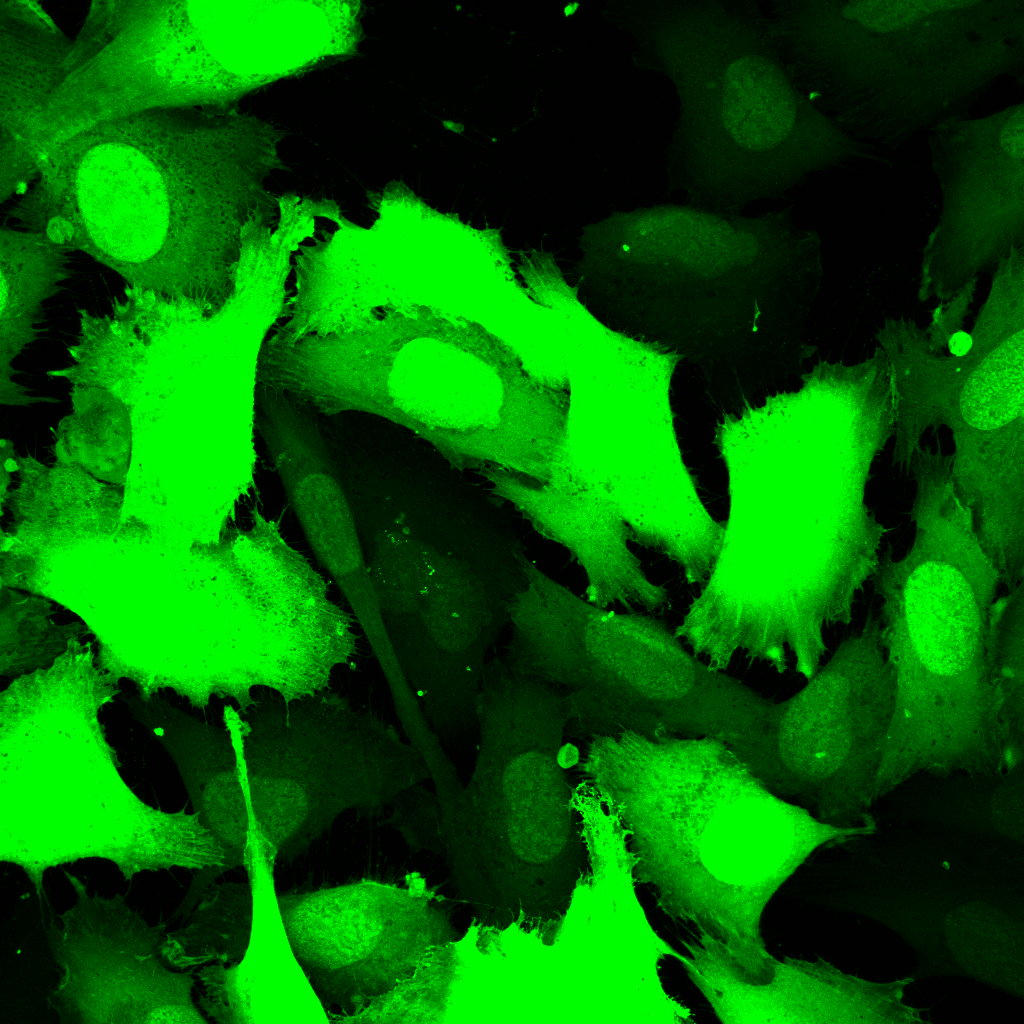

Supplement: Soure data 2. [file elife-32490-fig2.zip › Figure 4- figure supplement 1/Panel b/WM165/WM165_MMP14_594_Max_c1.tif]

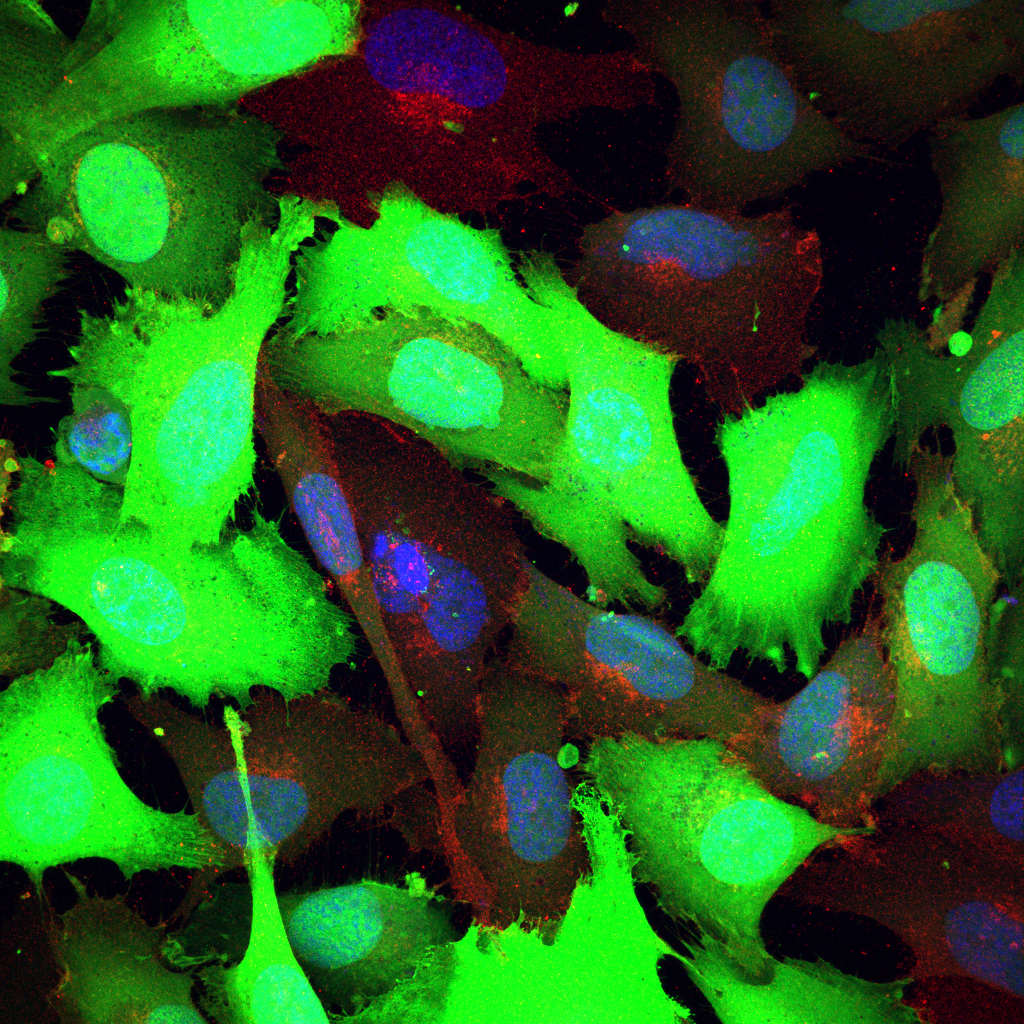

Supplement: Soure data 2. [file elife-32490-fig2.zip › Figure 4- figure supplement 1/Panel b/WM165/WM165_MMP14_594_Max_c1+2+3.tif]

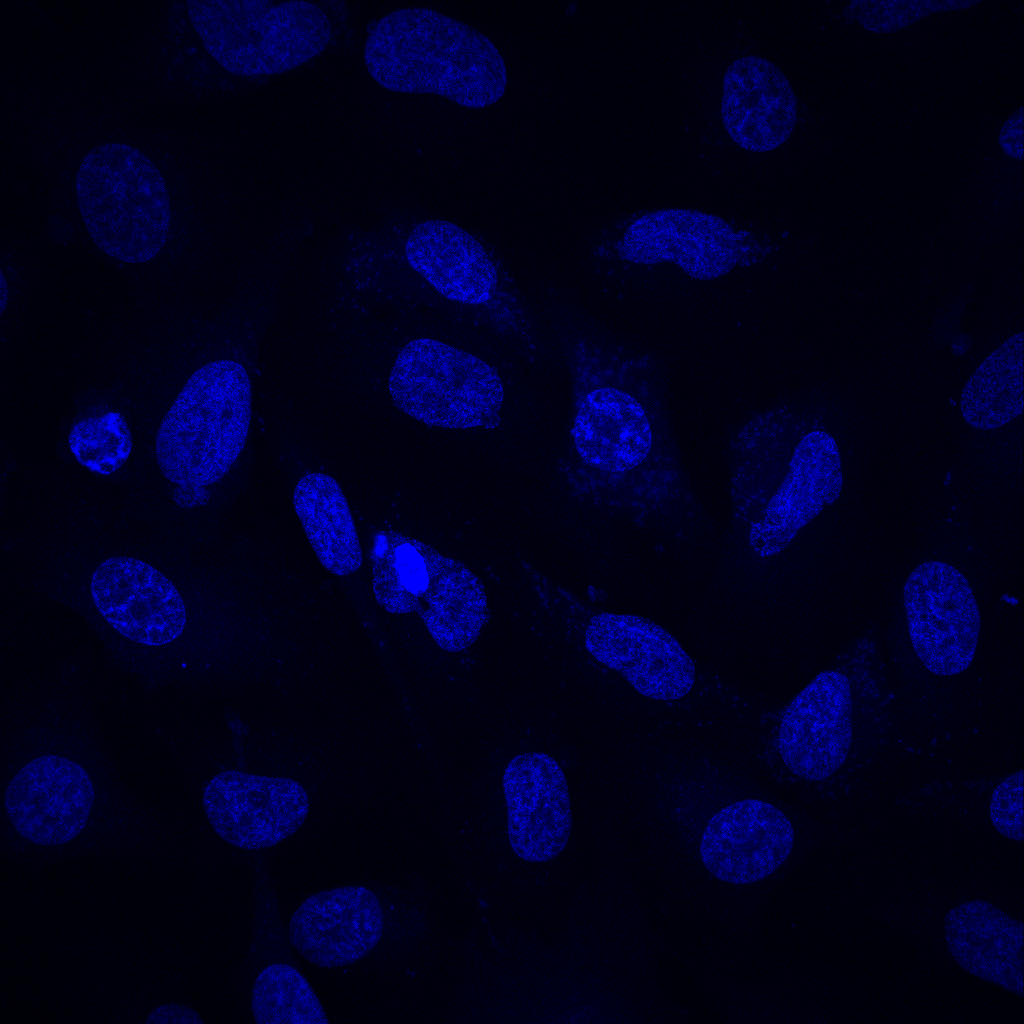

Supplement: Soure data 2. [file elife-32490-fig2.zip › Figure 4- figure supplement 1/Panel b/WM165/WM165_MMP14_594_Max_c2.tif]

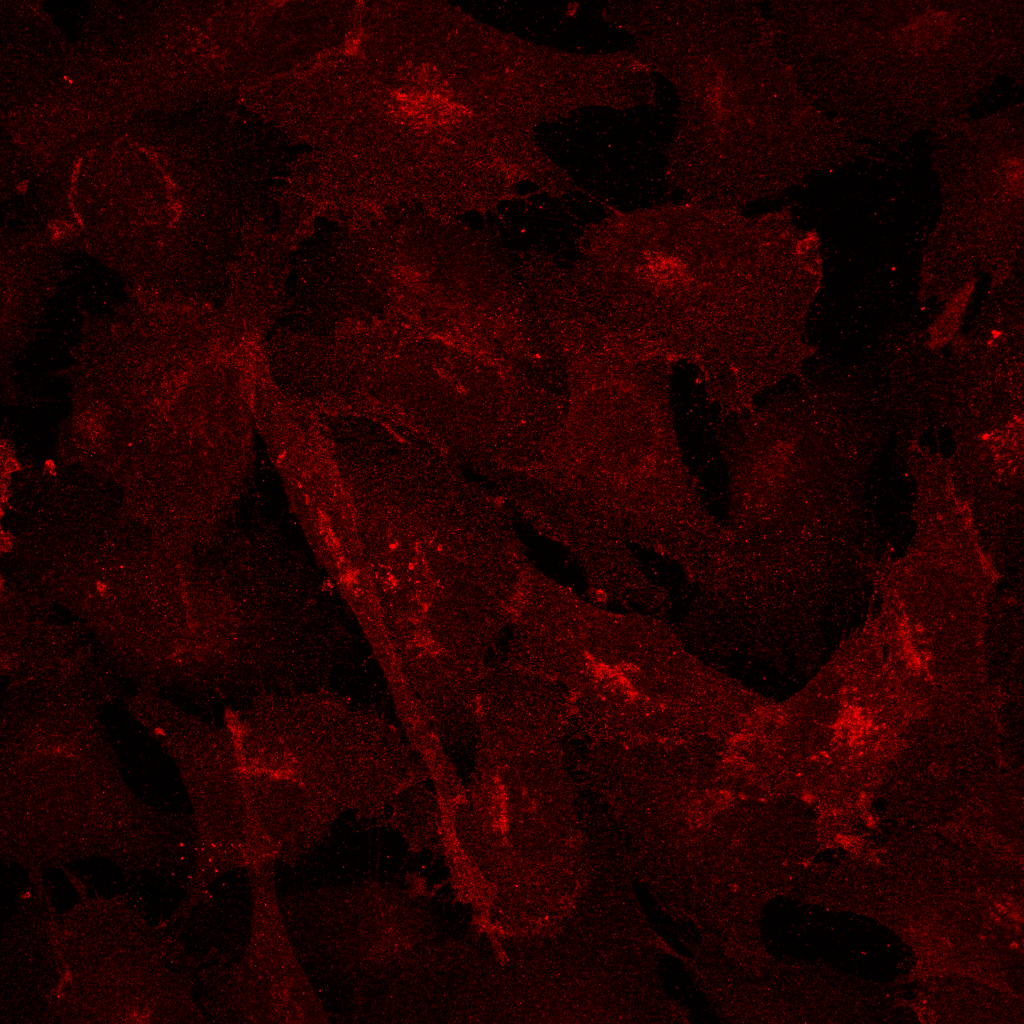

Supplement: Soure data 2. [file elife-32490-fig2.zip › Figure 4- figure supplement 1/Panel b/WM165/WM165_MMP14_594_Max_c3.tif]

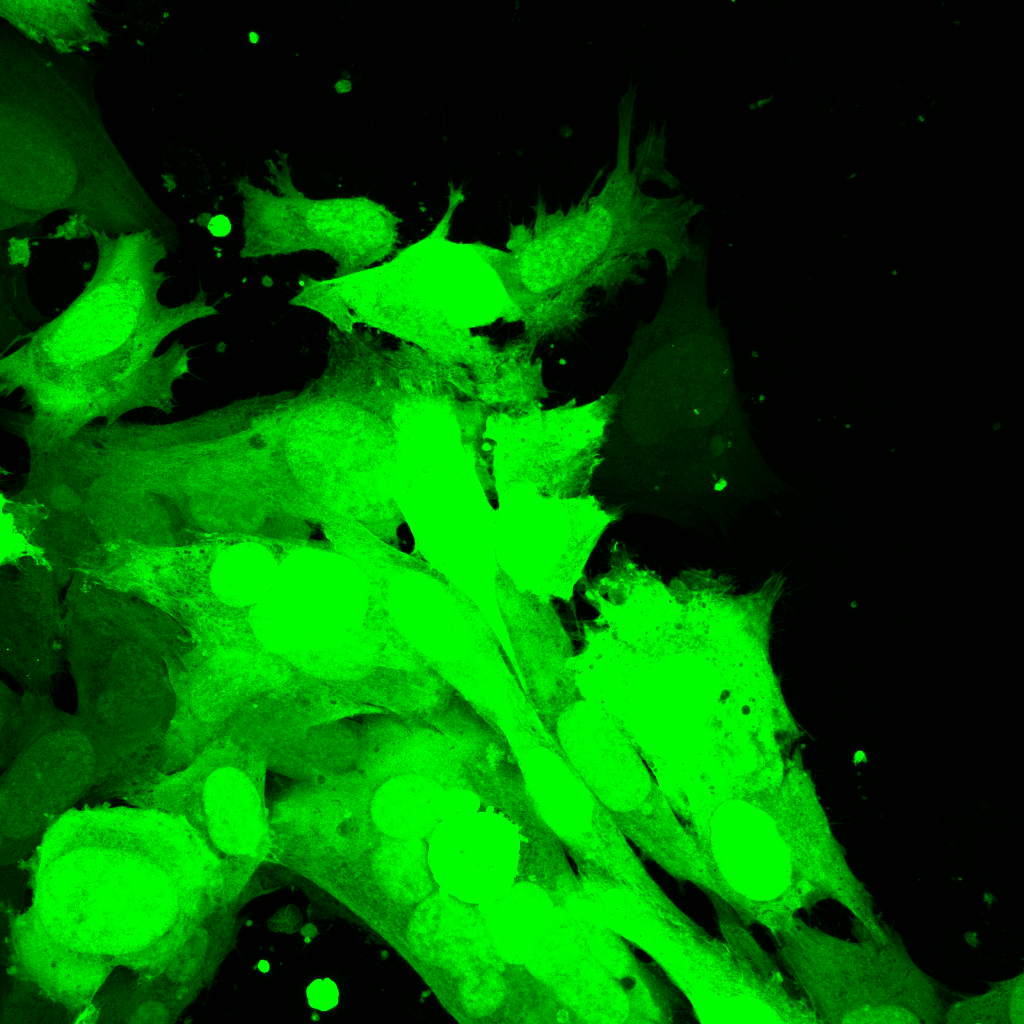

Supplement: Soure data 2. [file elife-32490-fig2.zip › Figure 4- figure supplement 1/Panel b/WM165_LEC/LEC_WM165_MMP14_Maximum intensity projection_c1.tif]

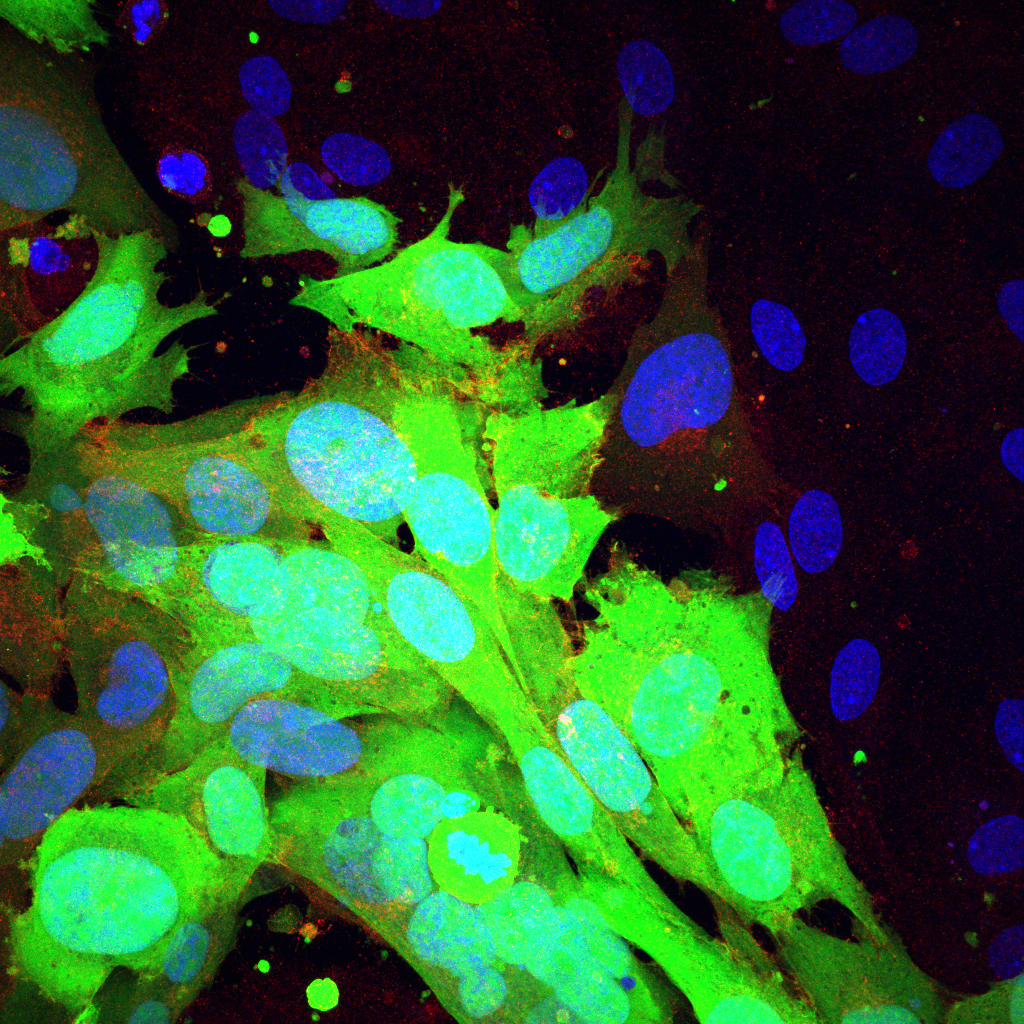

Supplement: Soure data 2. [file elife-32490-fig2.zip › Figure 4- figure supplement 1/Panel b/WM165_LEC/LEC_WM165_MMP14_Maximum intensity projection_c1+2+3.tif]

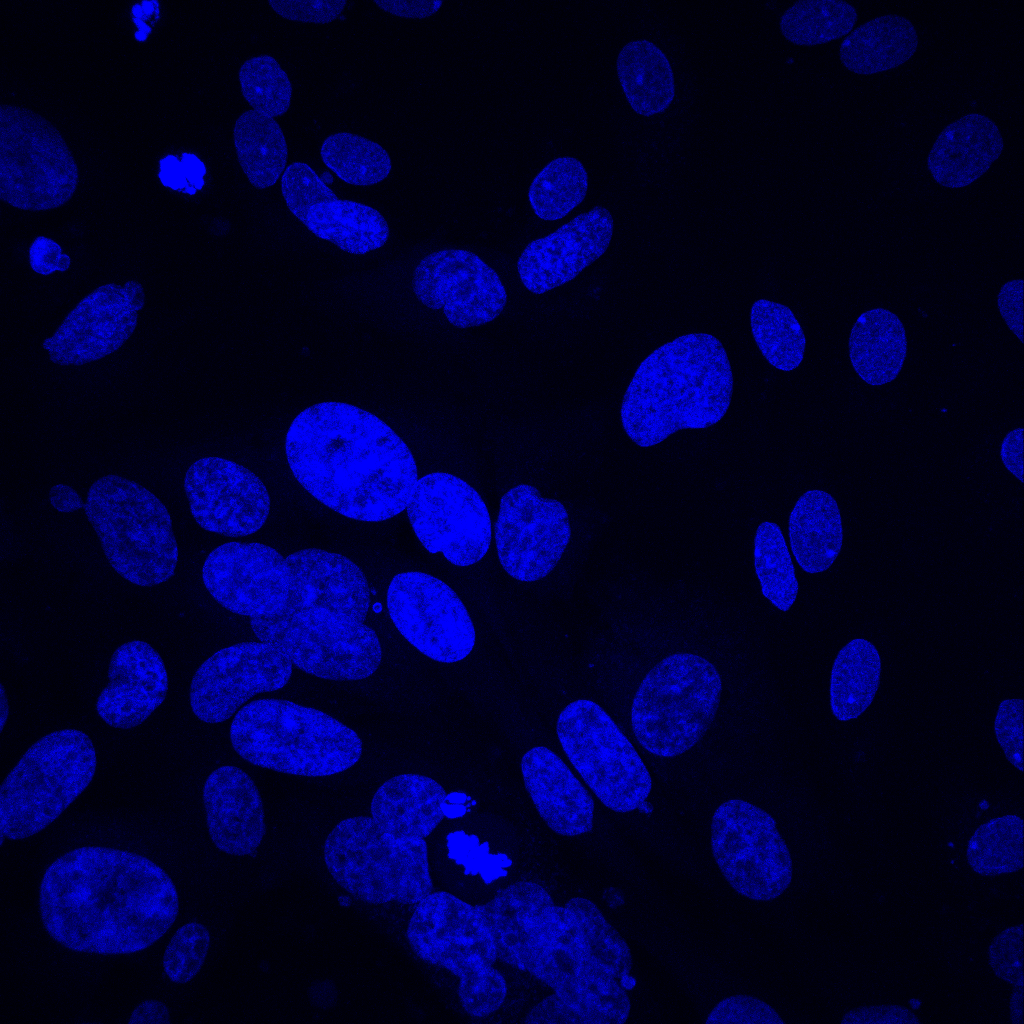

Supplement: Soure data 2. [file elife-32490-fig2.zip › Figure 4- figure supplement 1/Panel b/WM165_LEC/LEC_WM165_MMP14_Maximum intensity projection_c2.tif]

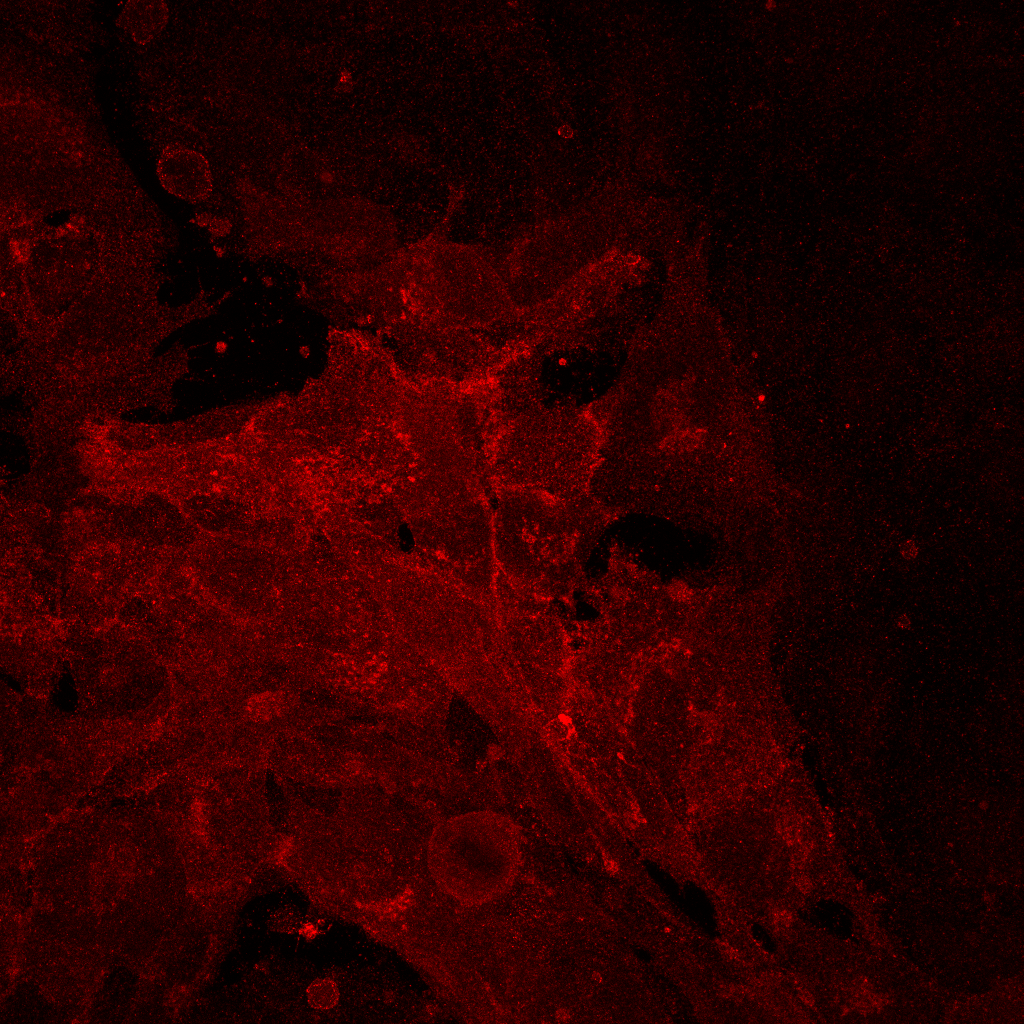

Supplement: Soure data 2. [file elife-32490-fig2.zip › Figure 4- figure supplement 1/Panel b/WM165_LEC/LEC_WM165_MMP14_Maximum intensity projection_c3.tif]

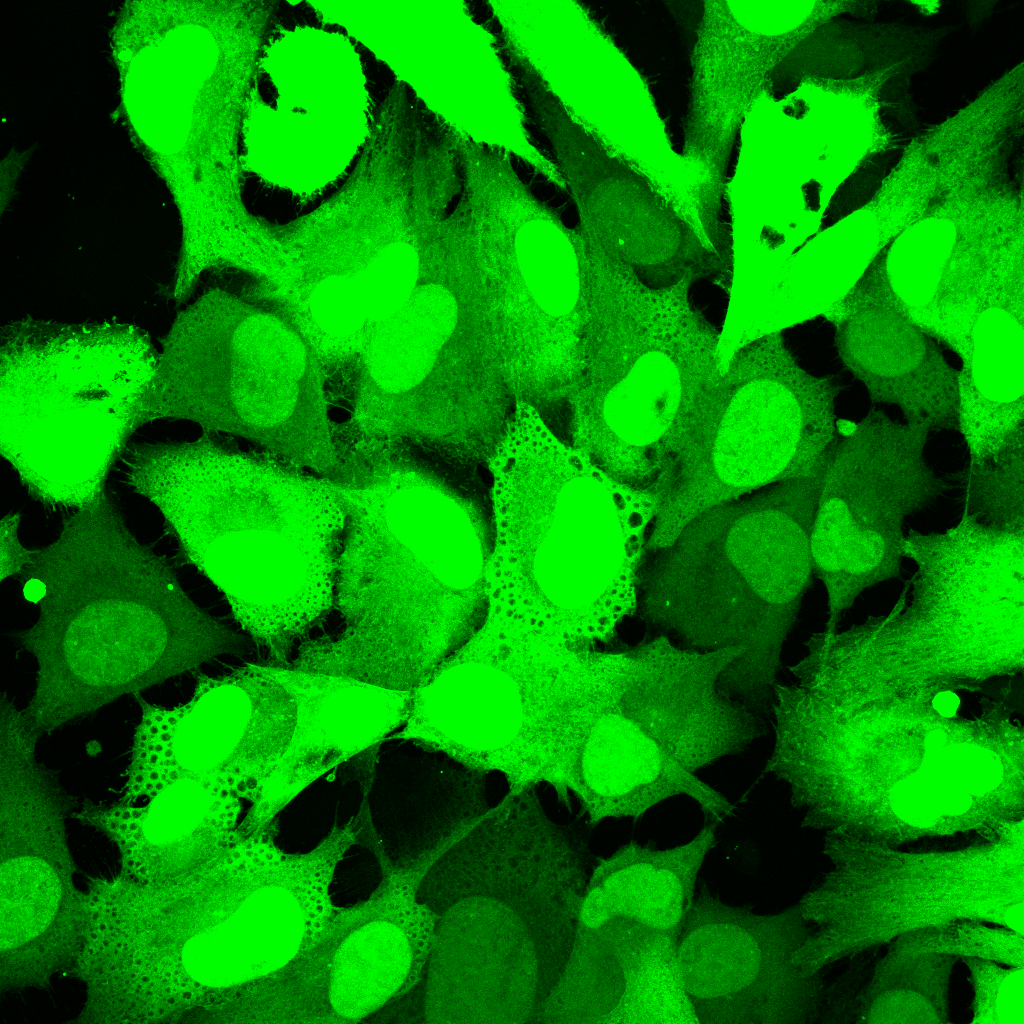

Supplement: Soure data 2. [file elife-32490-fig2.zip › Figure 4- figure supplement 1/Panel b/WM793/wm793_mmp14_Maximum intensity projection_c1.tif]

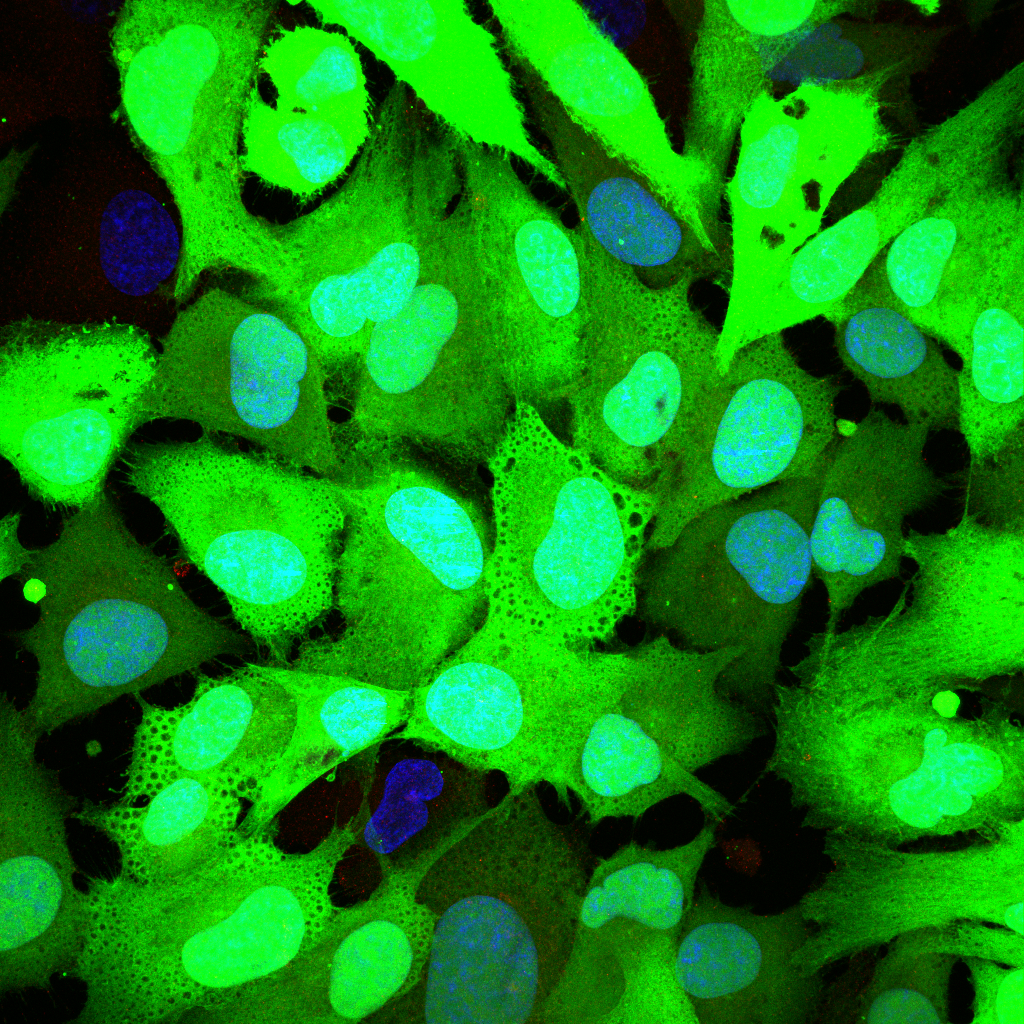

Supplement: Soure data 2. [file elife-32490-fig2.zip › Figure 4- figure supplement 1/Panel b/WM793/wm793_mmp14_Maximum intensity projection_c1+2+3.tif]

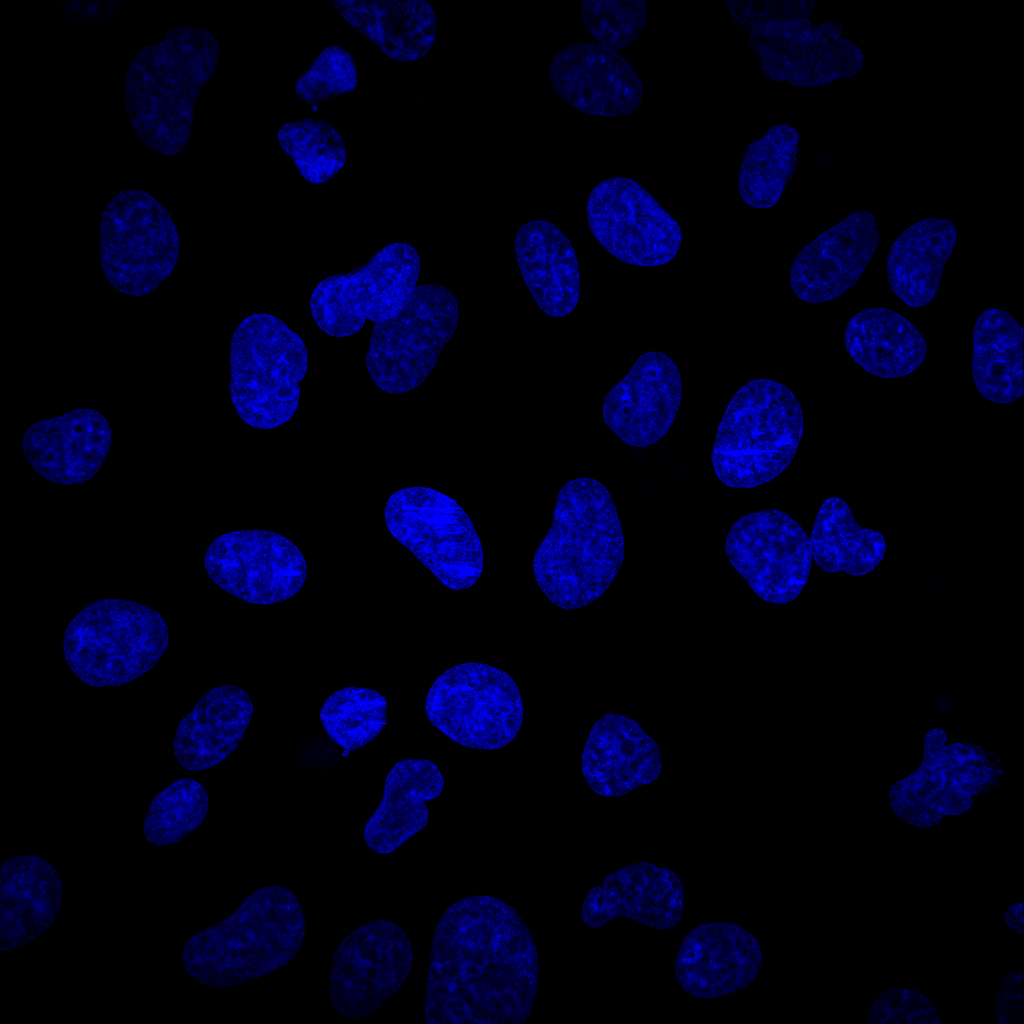

Supplement: Soure data 2. [file elife-32490-fig2.zip › Figure 4- figure supplement 1/Panel b/WM793/wm793_mmp14_Maximum intensity projection_c2.tif]

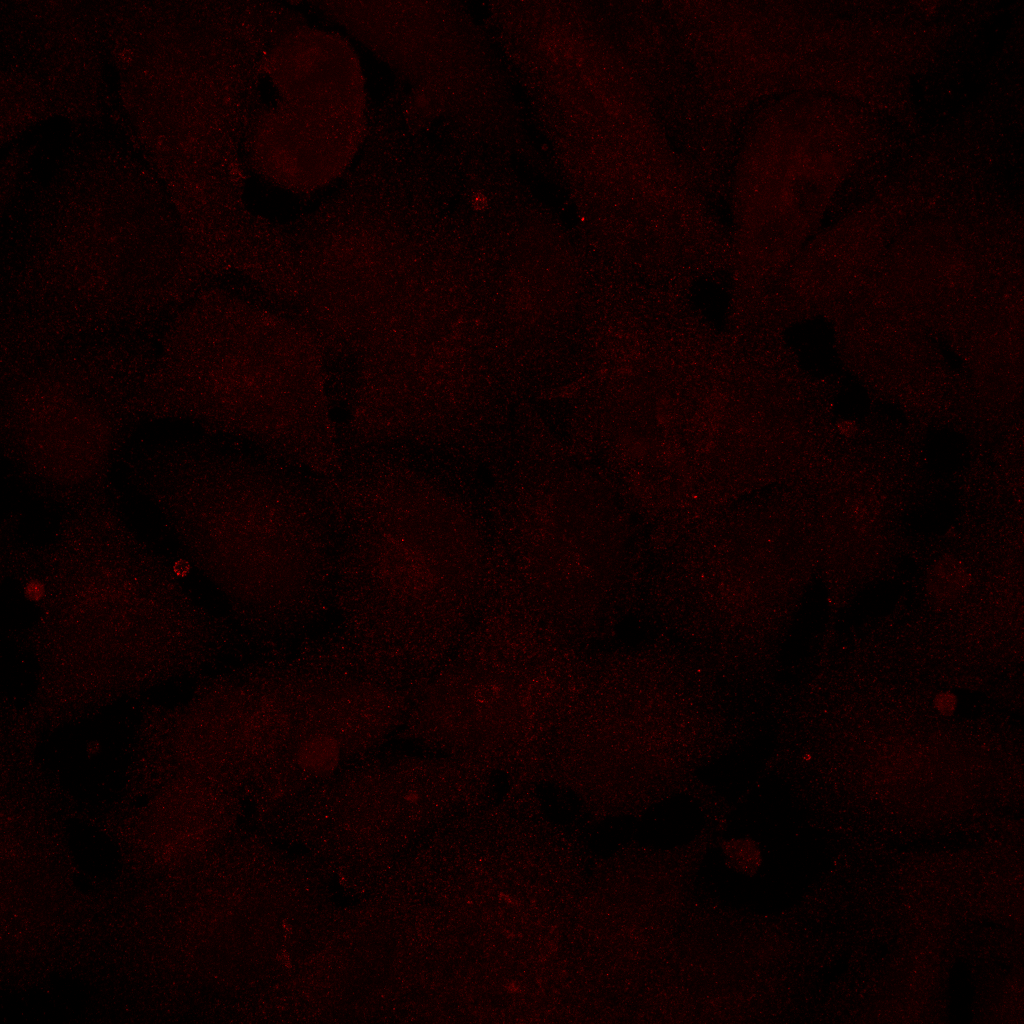

Supplement: Soure data 2. [file elife-32490-fig2.zip › Figure 4- figure supplement 1/Panel b/WM793/wm793_mmp14_Maximum intensity projection_c3.tif]

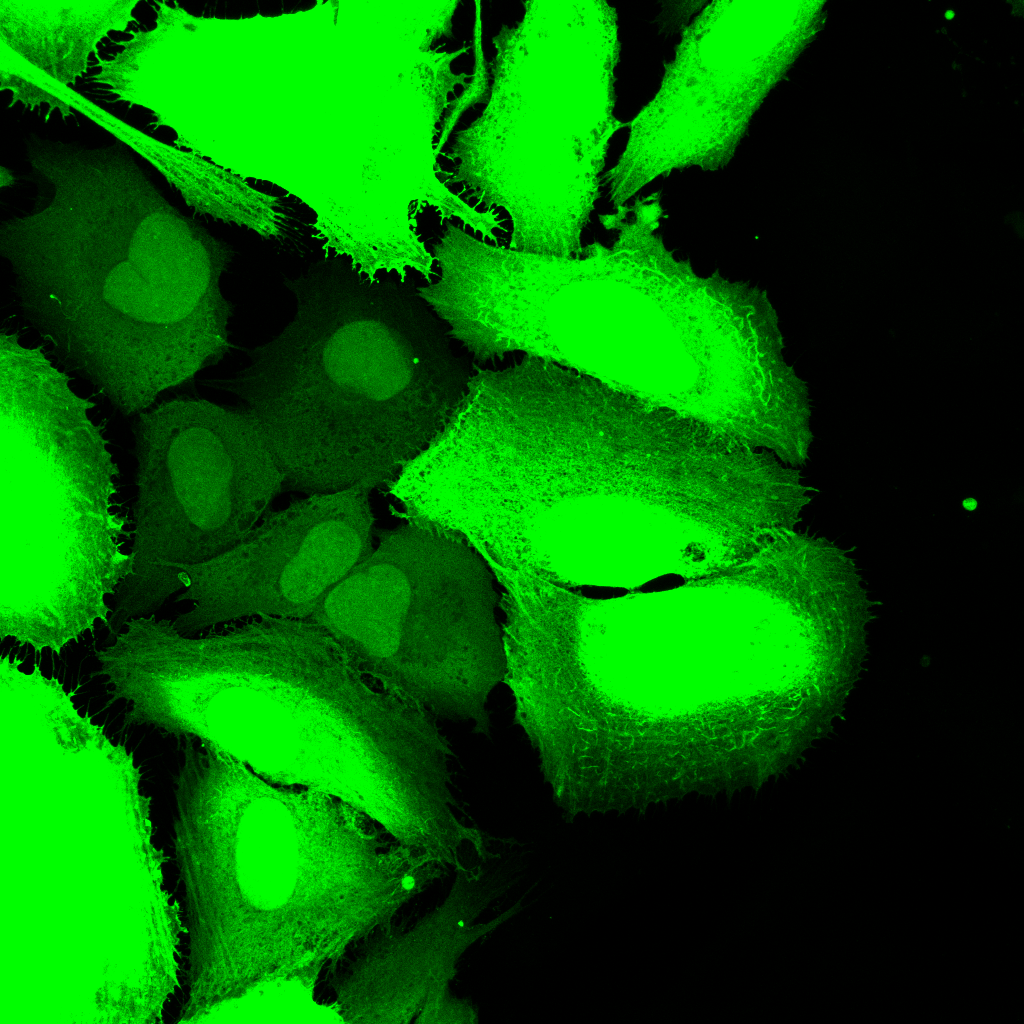

Supplement: Soure data 2. [file elife-32490-fig2.zip › Figure 4- figure supplement 1/Panel b/WM793_LEC/LEC_WM793_MMP14_594_Max_c1.tif]

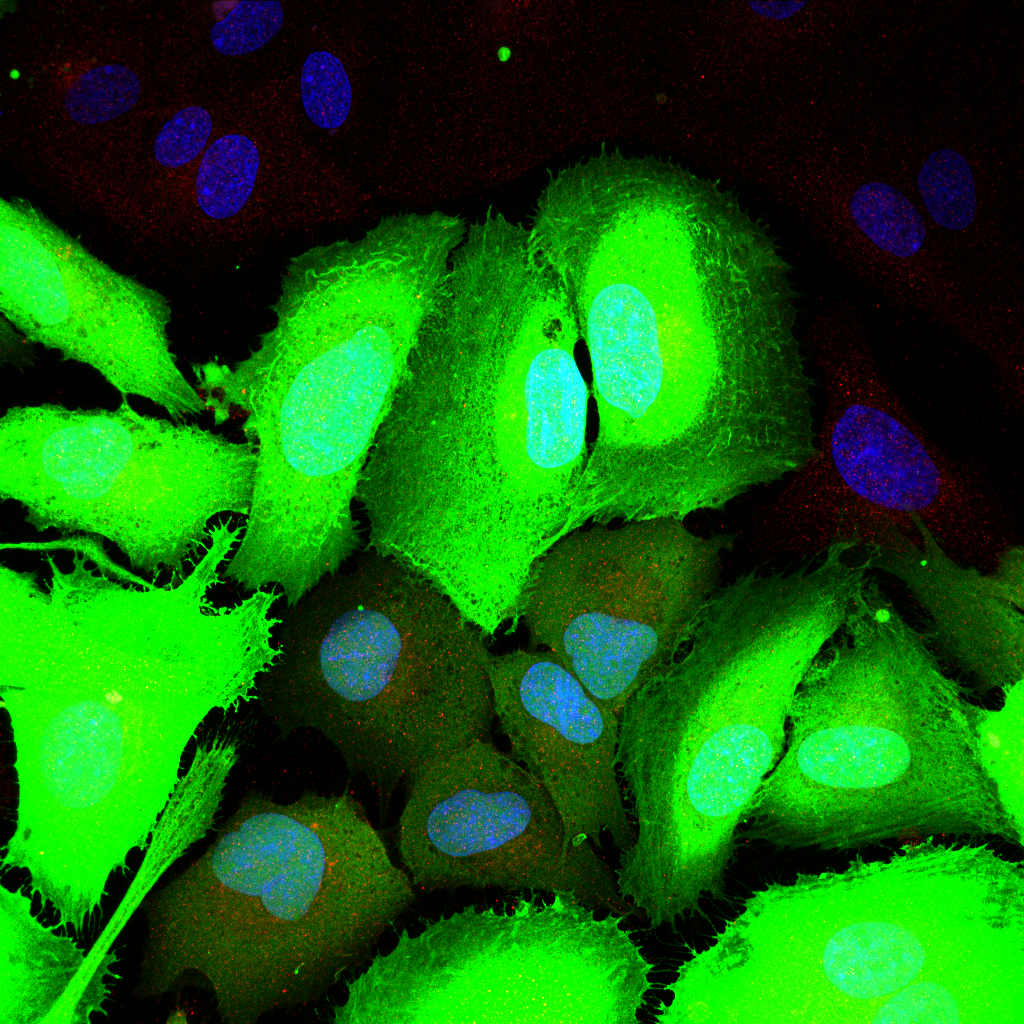

Supplement: Soure data 2. [file elife-32490-fig2.zip › Figure 4- figure supplement 1/Panel b/WM793_LEC/LEC_WM793_MMP14_594_Max_c1+2+3.tif]

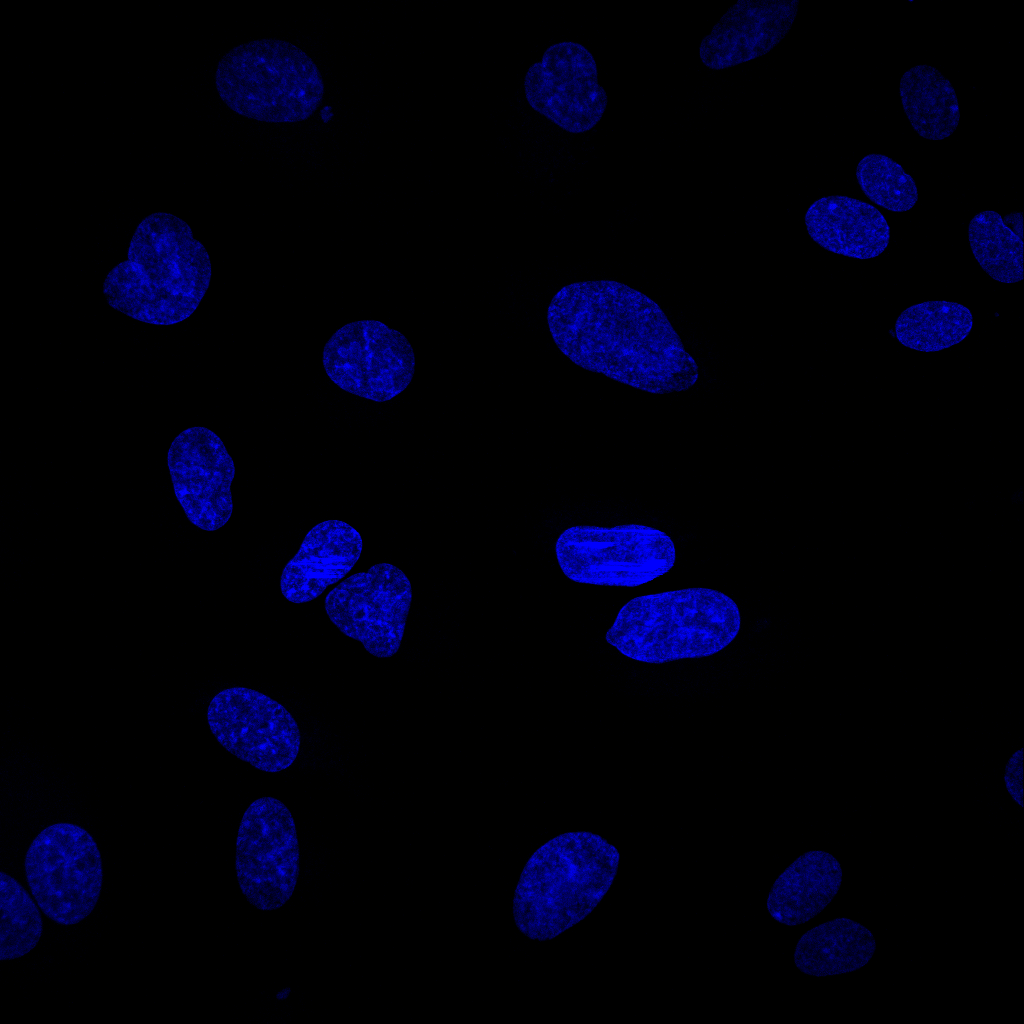

Supplement: Soure data 2. [file elife-32490-fig2.zip › Figure 4- figure supplement 1/Panel b/WM793_LEC/LEC_WM793_MMP14_594_Max_c2.tif]

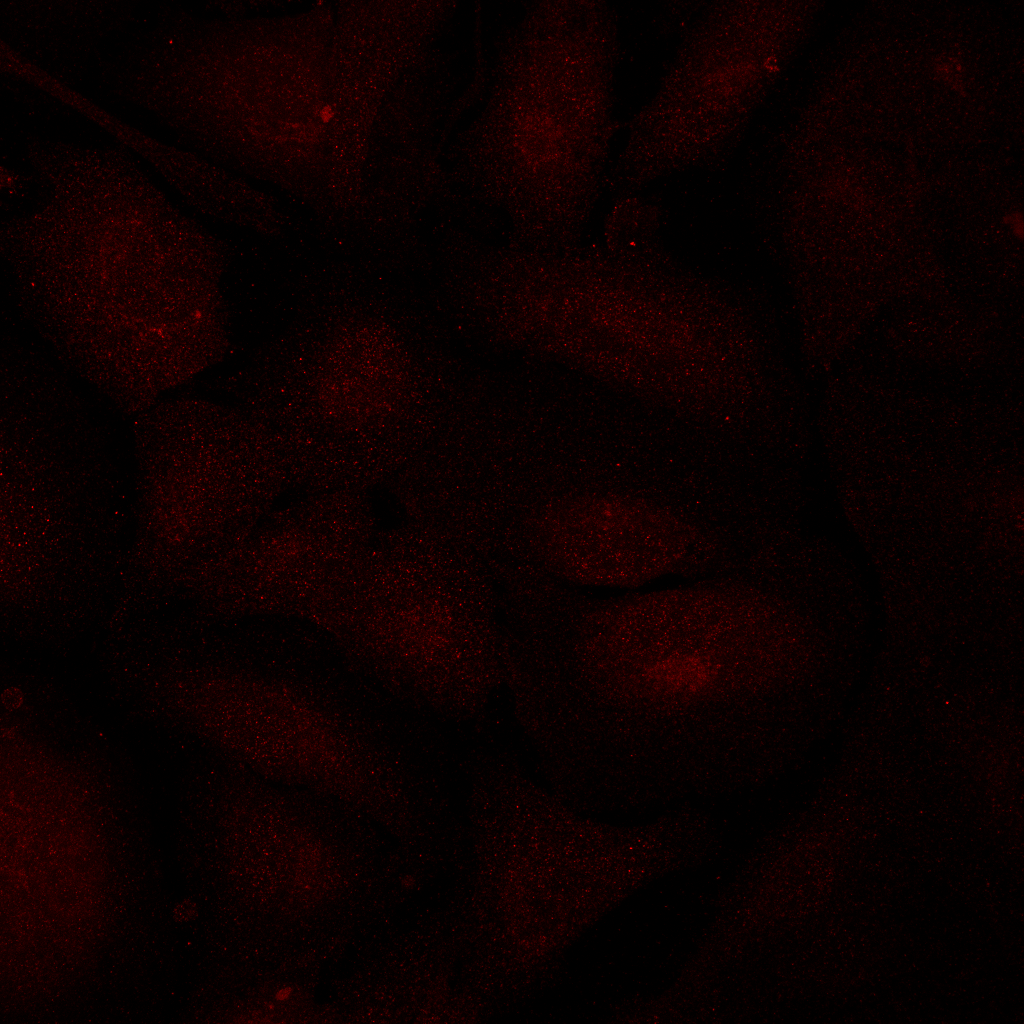

Supplement: Soure data 2. [file elife-32490-fig2.zip › Figure 4- figure supplement 1/Panel b/WM793_LEC/LEC_WM793_MMP14_594_Max_c3.tif]

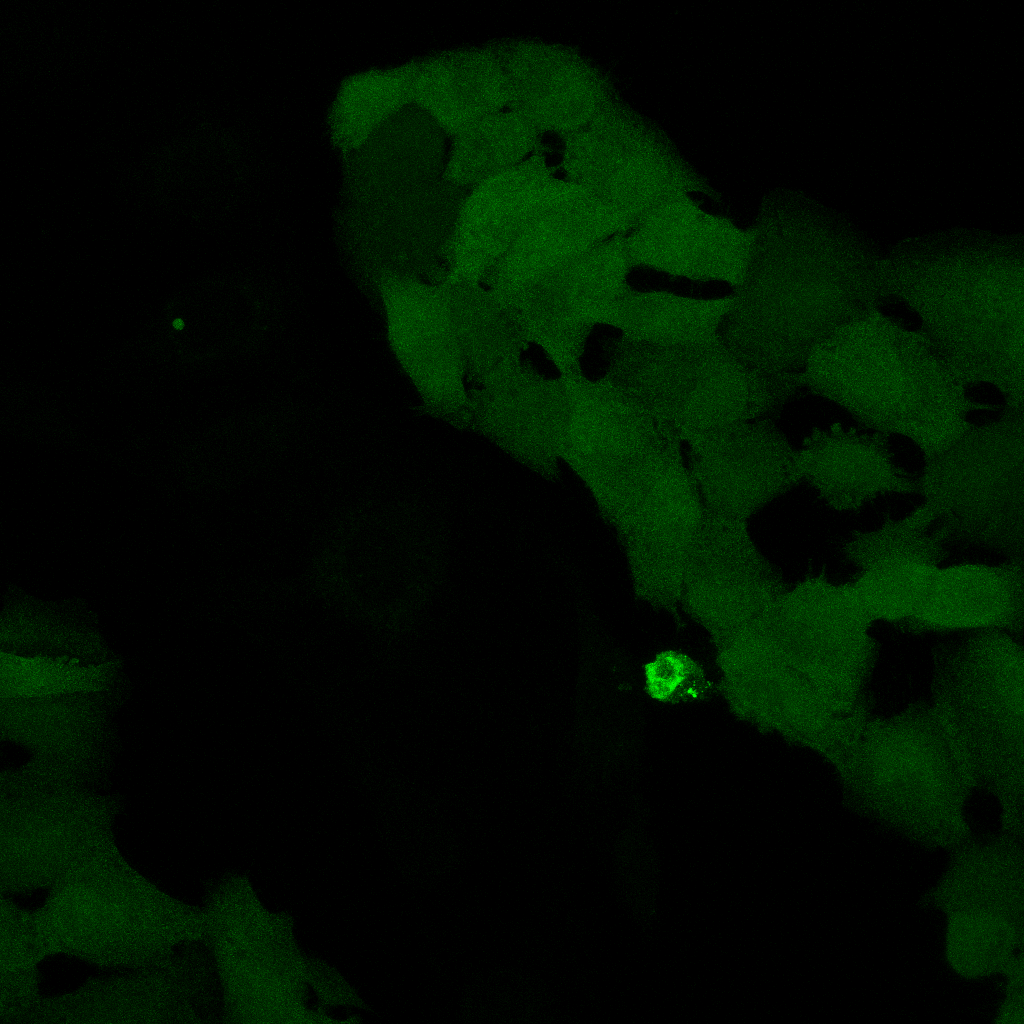

Supplement: Soure data 2. [file elife-32490-fig2.zip › Figure 4-figure supplement 2/Panel c/siCtrl/LEC_siCtrl_MMP14_Maximum intensity projection_c1.tif]

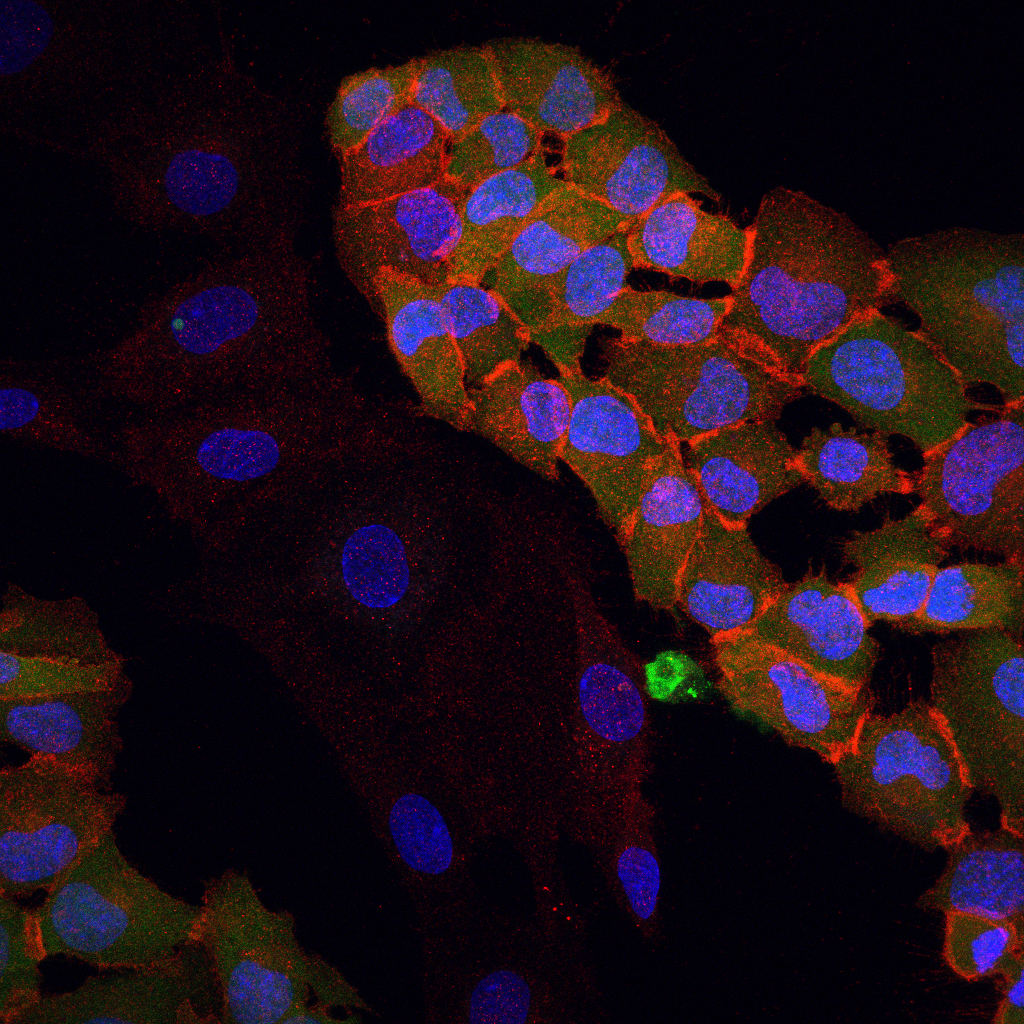

Supplement: Soure data 2. [file elife-32490-fig2.zip › Figure 4-figure supplement 2/Panel c/siCtrl/LEC_siCtrl_MMP14_Maximum intensity projection_c1+2+3.tif]

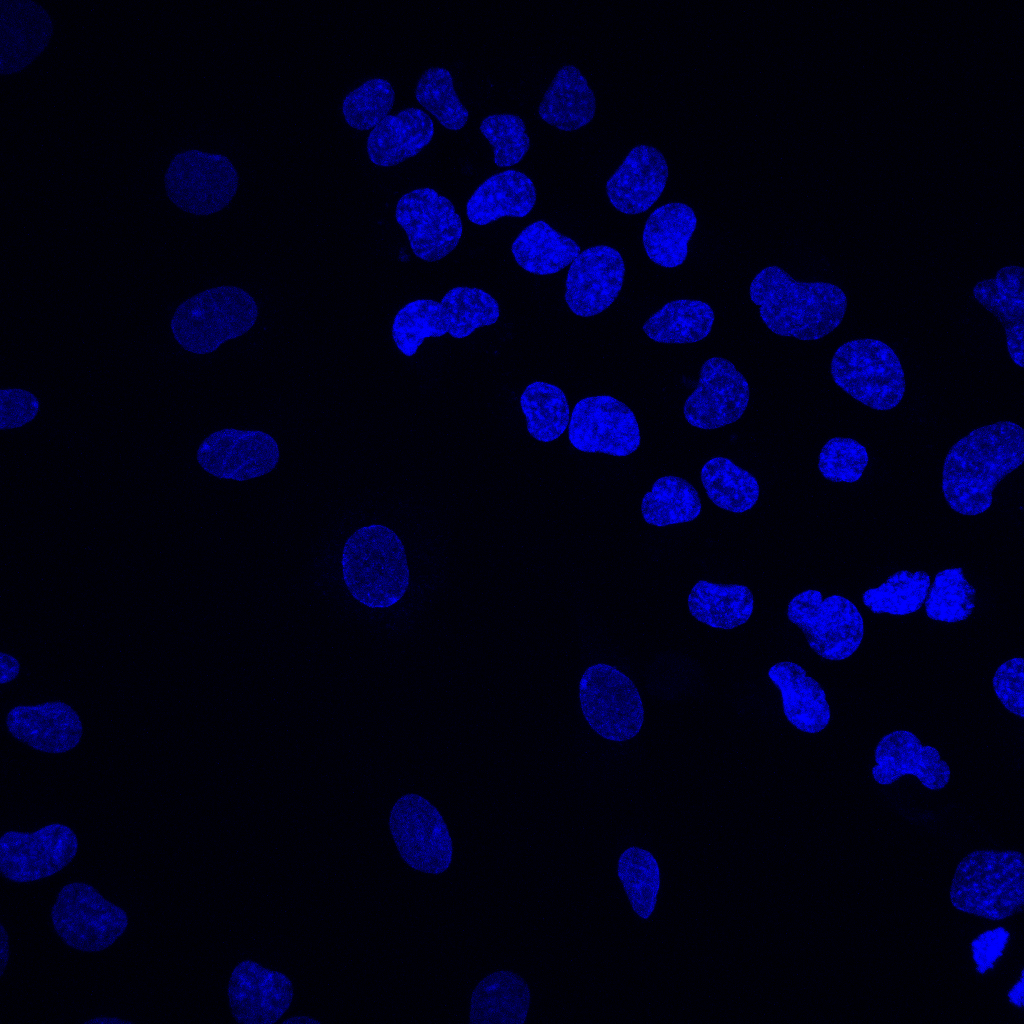

Supplement: Soure data 2. [file elife-32490-fig2.zip › Figure 4-figure supplement 2/Panel c/siCtrl/LEC_siCtrl_MMP14_Maximum intensity projection_c2.tif]

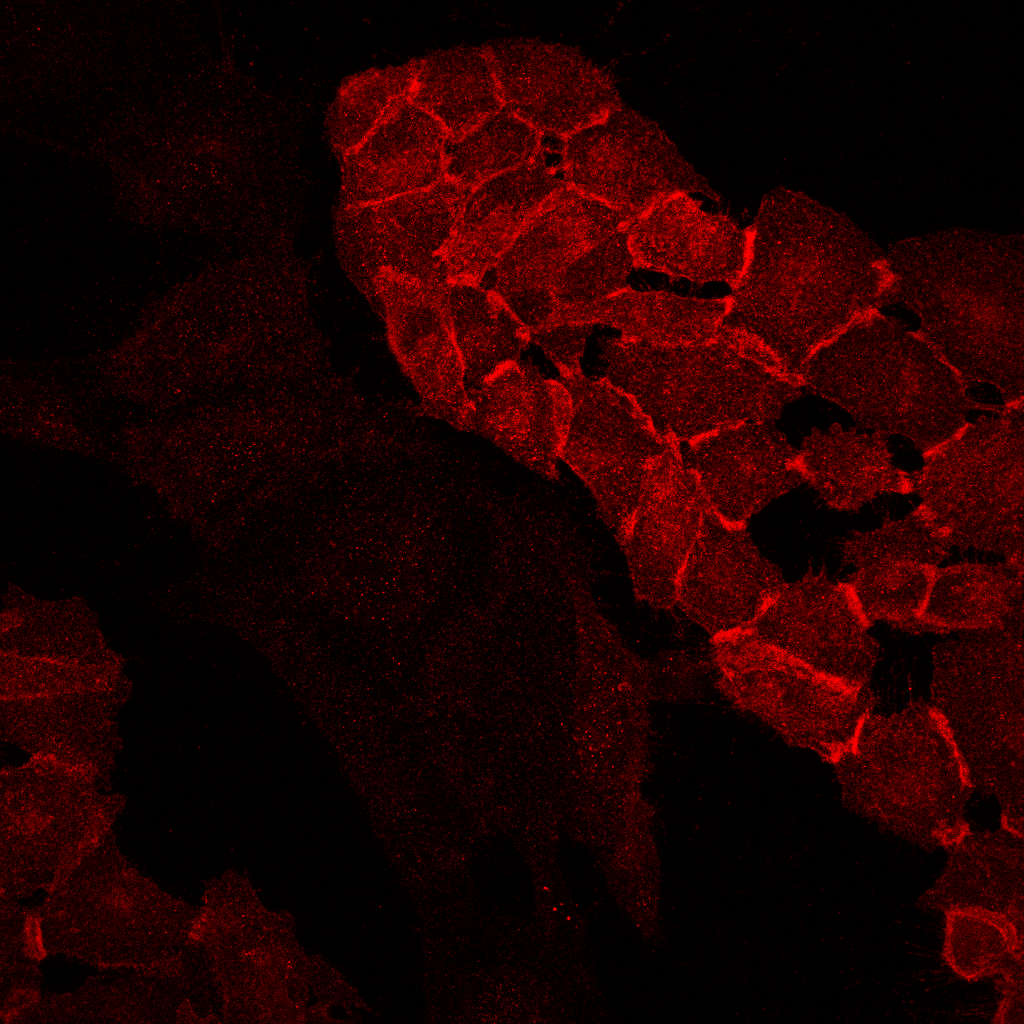

Supplement: Soure data 2. [file elife-32490-fig2.zip › Figure 4-figure supplement 2/Panel c/siCtrl/LEC_siCtrl_MMP14_Maximum intensity projection_c3.tif]

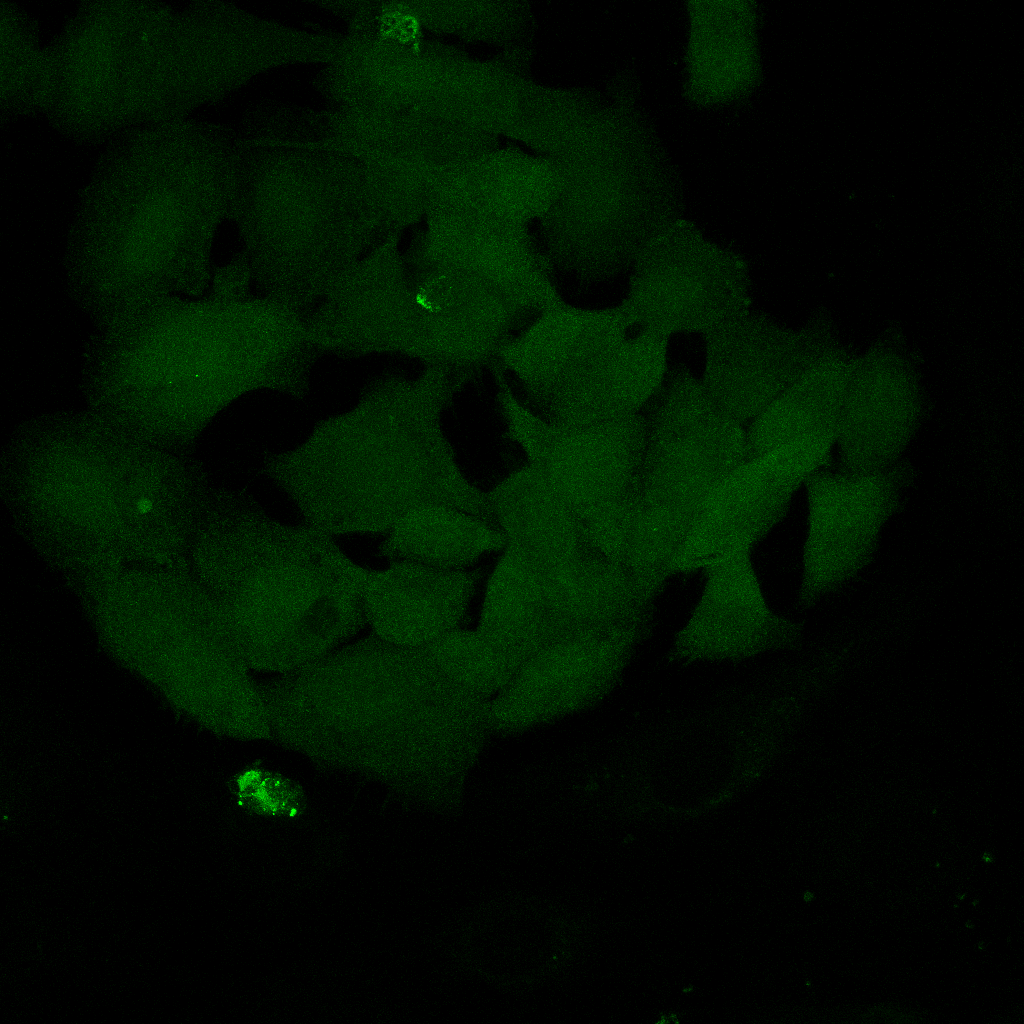

Supplement: Soure data 2. [file elife-32490-fig2.zip › Figure 4-figure supplement 2/Panel c/siNotch3/LEC_siNotch3_MMP14_Maximum intensity projection_c1.tif]

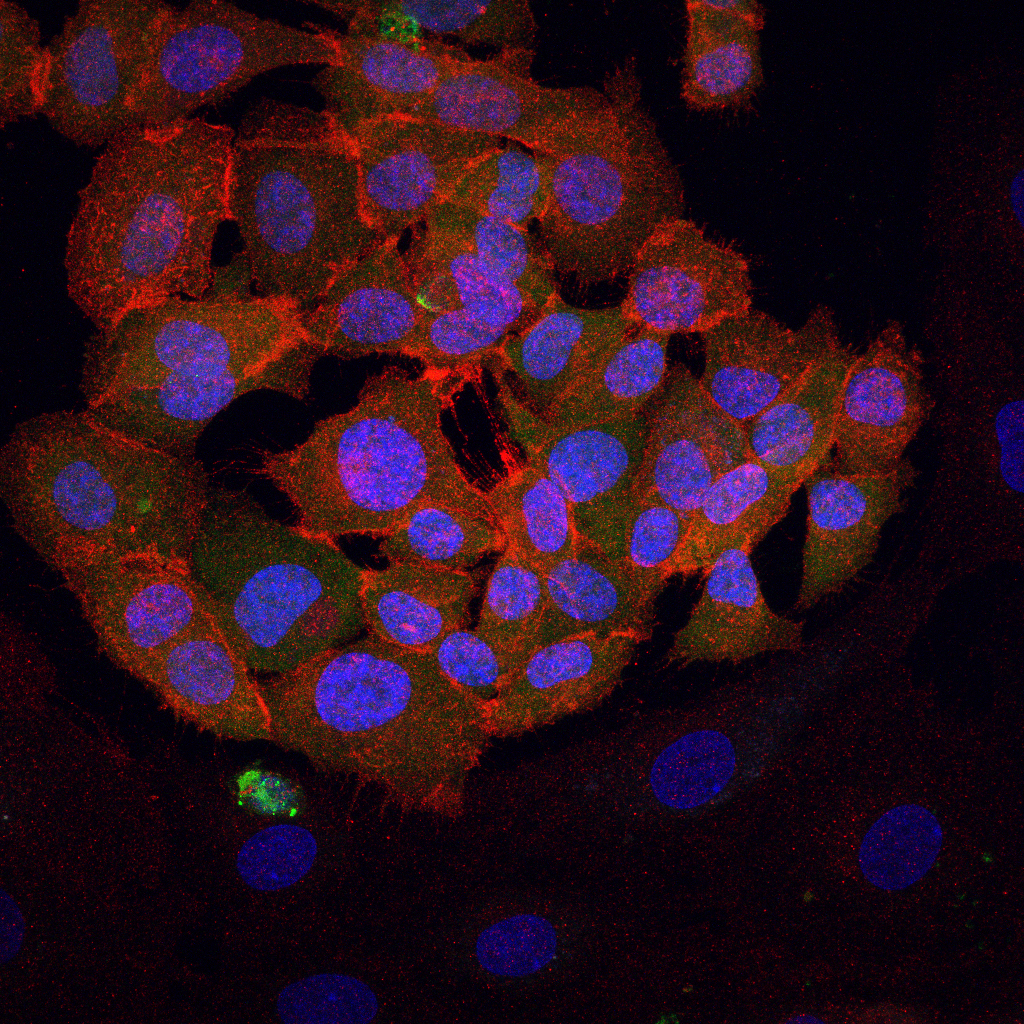

Supplement: Soure data 2. [file elife-32490-fig2.zip › Figure 4-figure supplement 2/Panel c/siNotch3/LEC_siNotch3_MMP14_Maximum intensity projection_c1+2+3.tif]

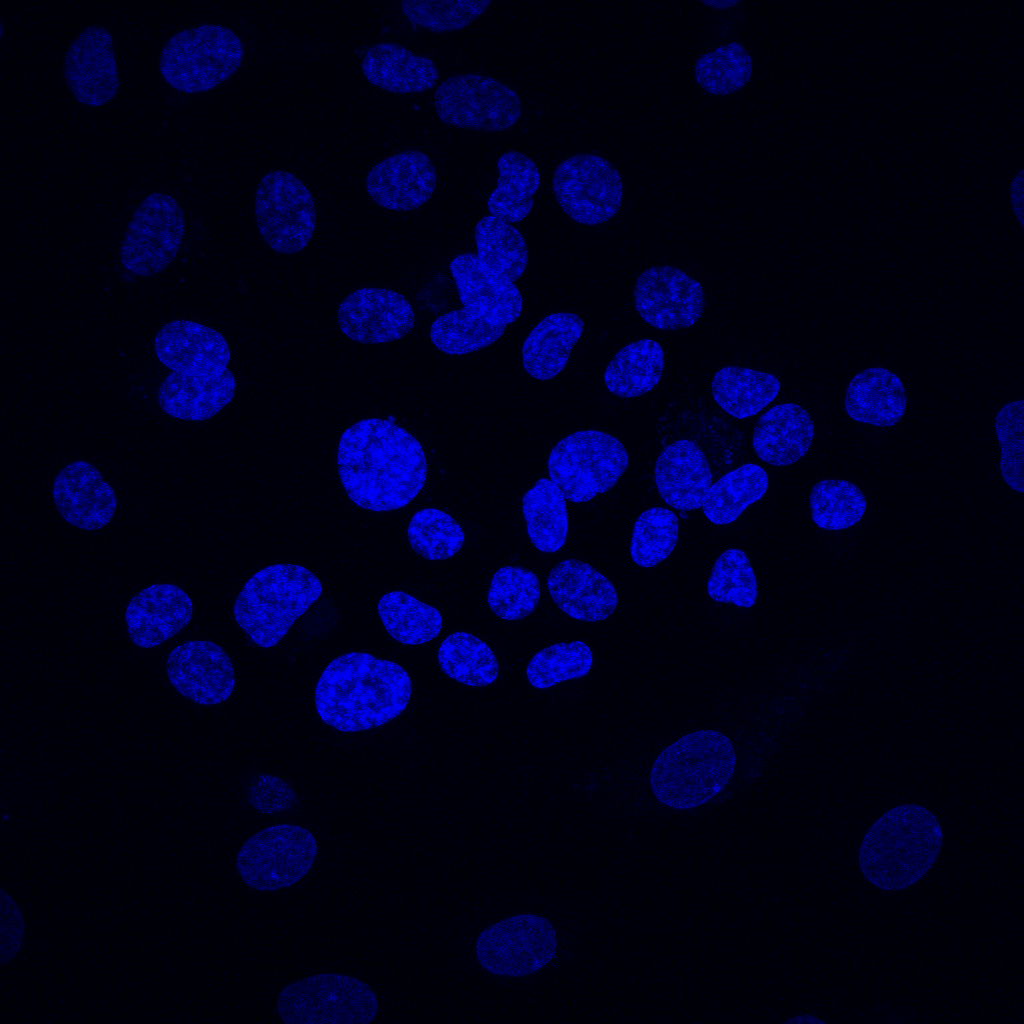

Supplement: Soure data 2. [file elife-32490-fig2.zip › Figure 4-figure supplement 2/Panel c/siNotch3/LEC_siNotch3_MMP14_Maximum intensity projection_c2.tif]

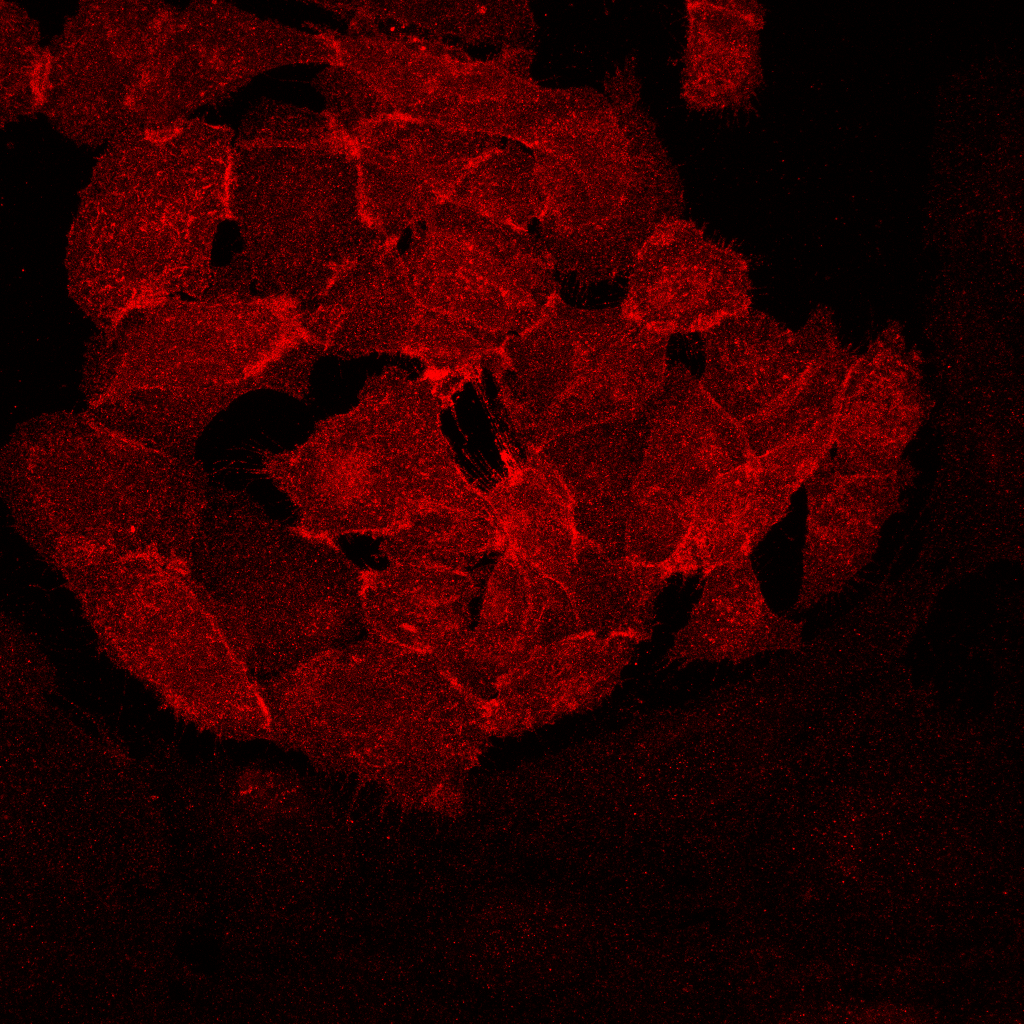

Supplement: Soure data 2. [file elife-32490-fig2.zip › Figure 4-figure supplement 2/Panel c/siNotch3/LEC_siNotch3_MMP14_Maximum intensity projection_c3.tif]

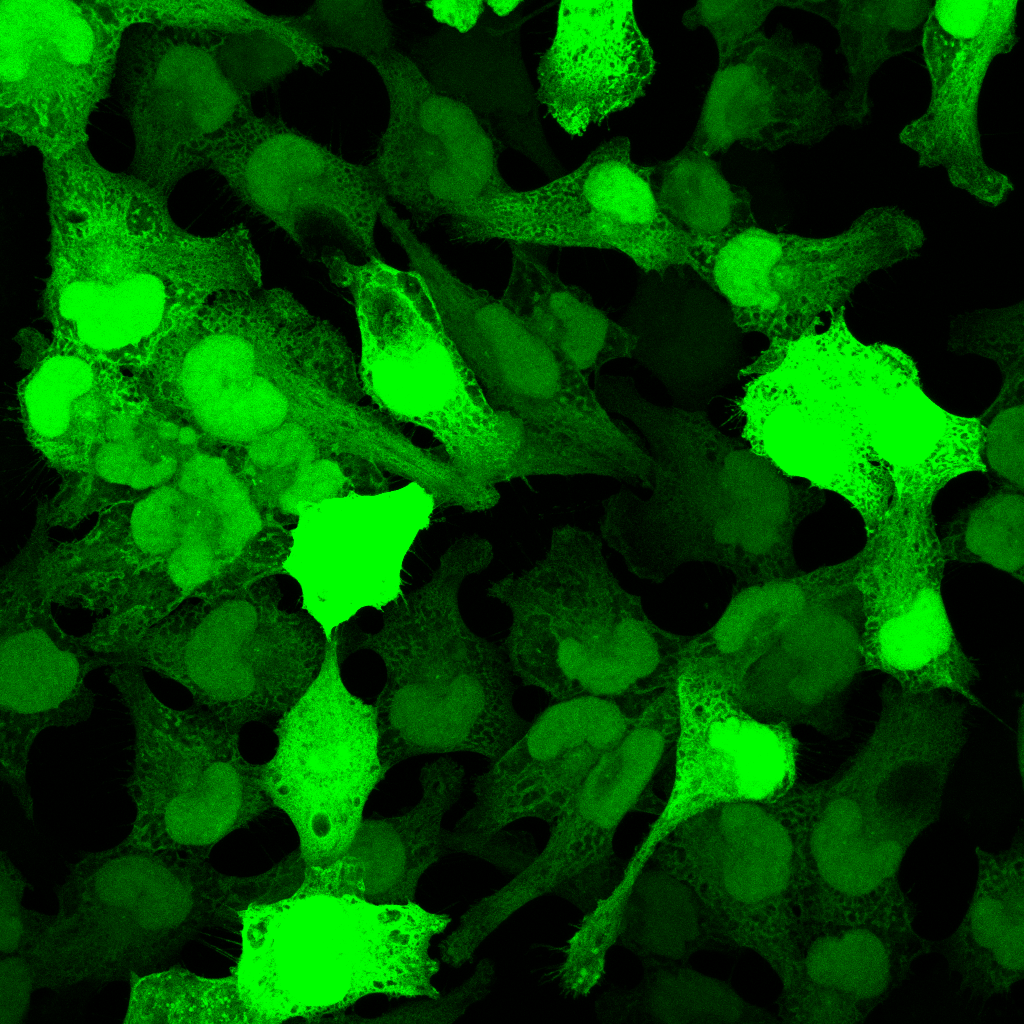

Supplement: Soure data 2. [file elife-32490-fig2.zip › Figure 4/Panel a/Bowes/Bowes_MMP14_594_Max_c1.tif]

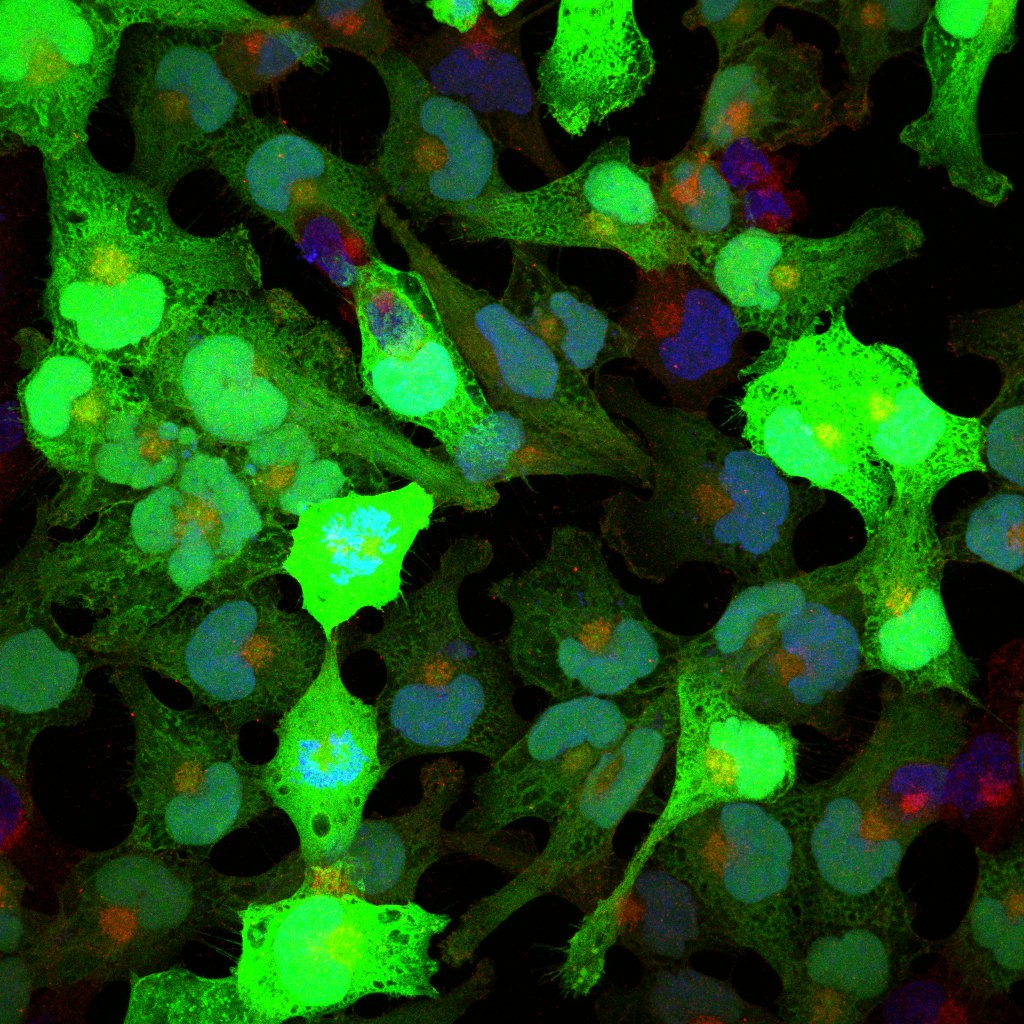

Supplement: Soure data 2. [file elife-32490-fig2.zip › Figure 4/Panel a/Bowes/Bowes_MMP14_594_Max_c1+2+3.tif]

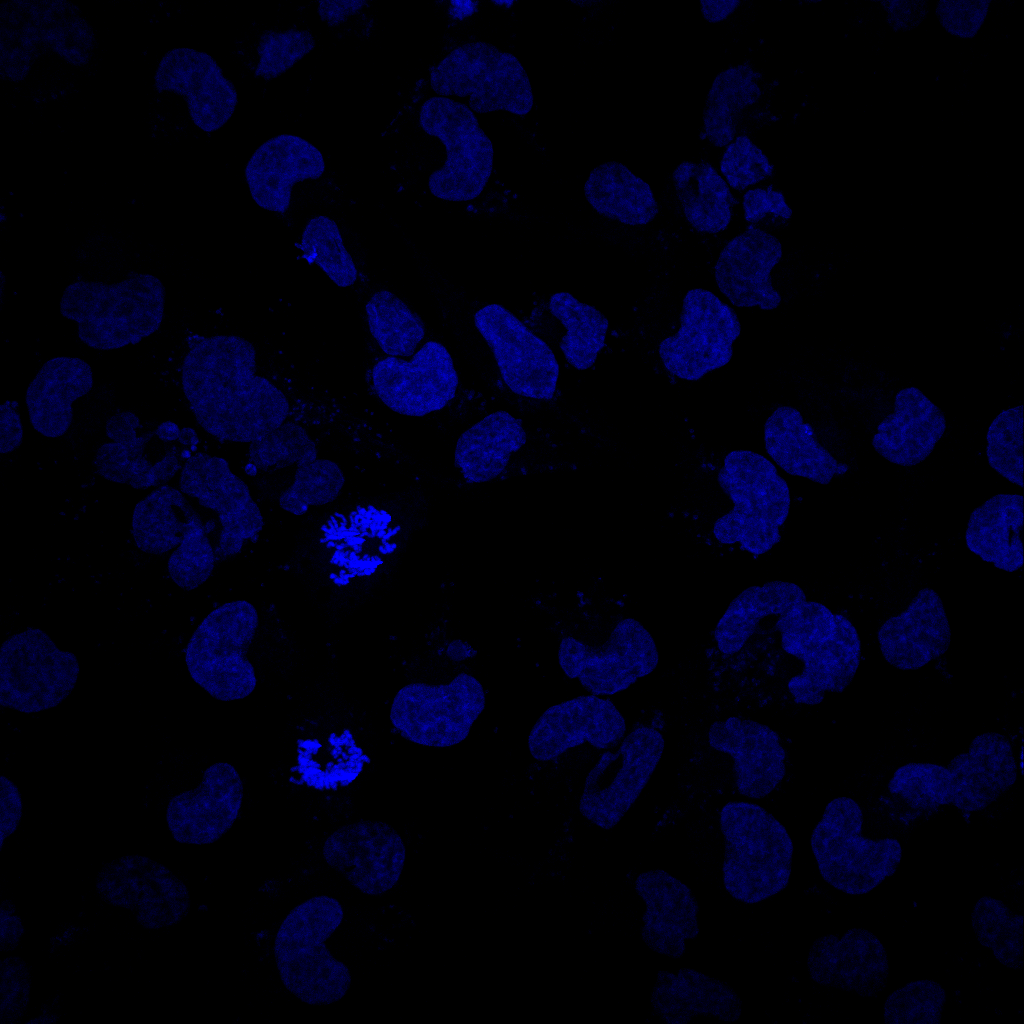

Supplement: Soure data 2. [file elife-32490-fig2.zip › Figure 4/Panel a/Bowes/Bowes_MMP14_594_Max_c2.tif]

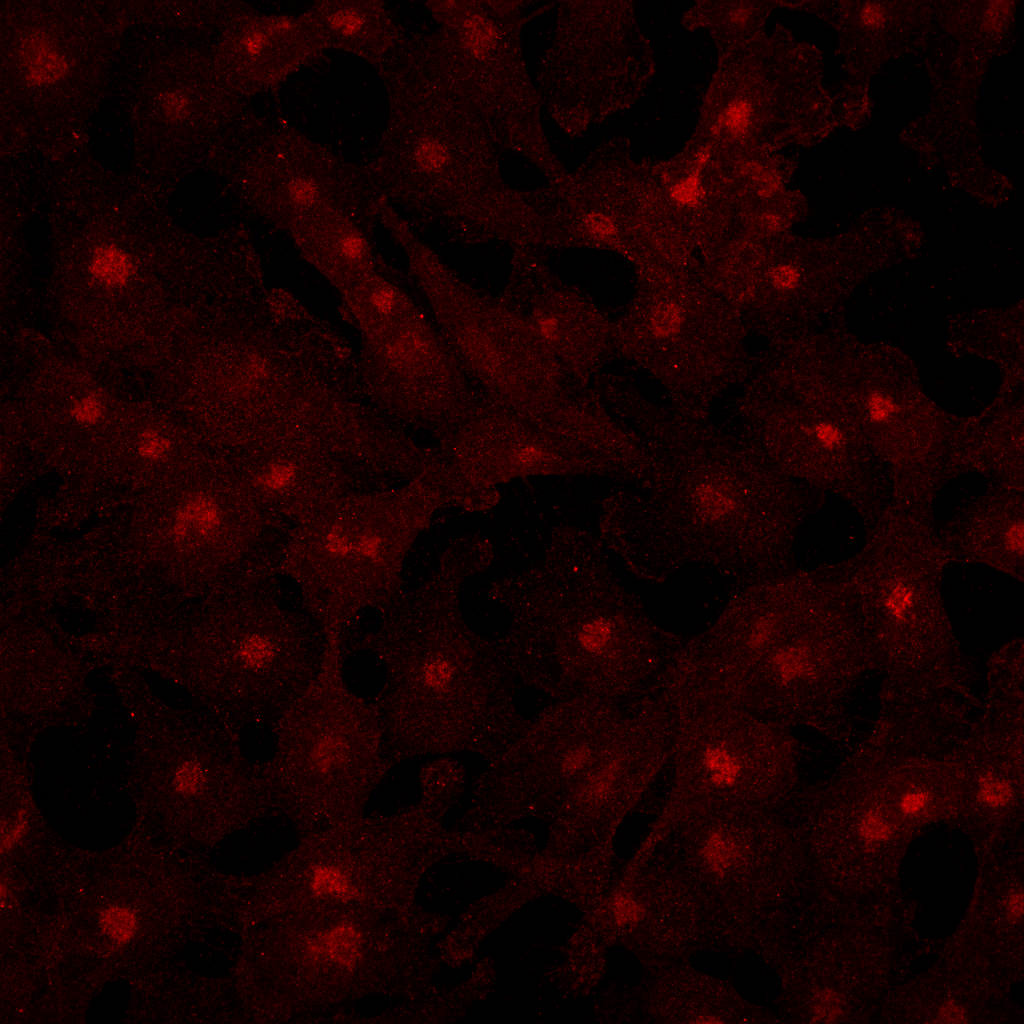

Supplement: Soure data 2. [file elife-32490-fig2.zip › Figure 4/Panel a/Bowes/Bowes_MMP14_594_Max_c3.tif]

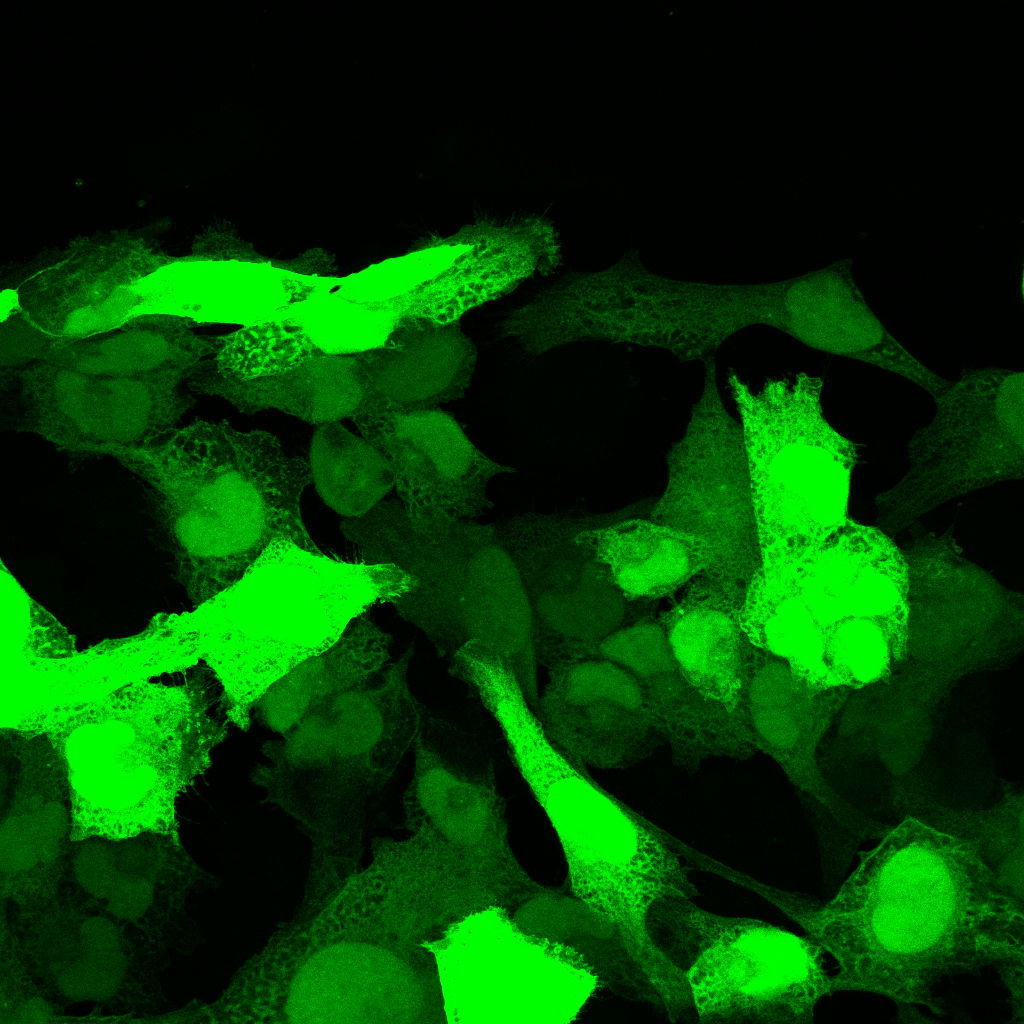

Supplement: Soure data 2. [file elife-32490-fig2.zip › Figure 4/Panel a/Bowes_LEC/LEC_Bowes_MMP14_594_Max_c1.tif]

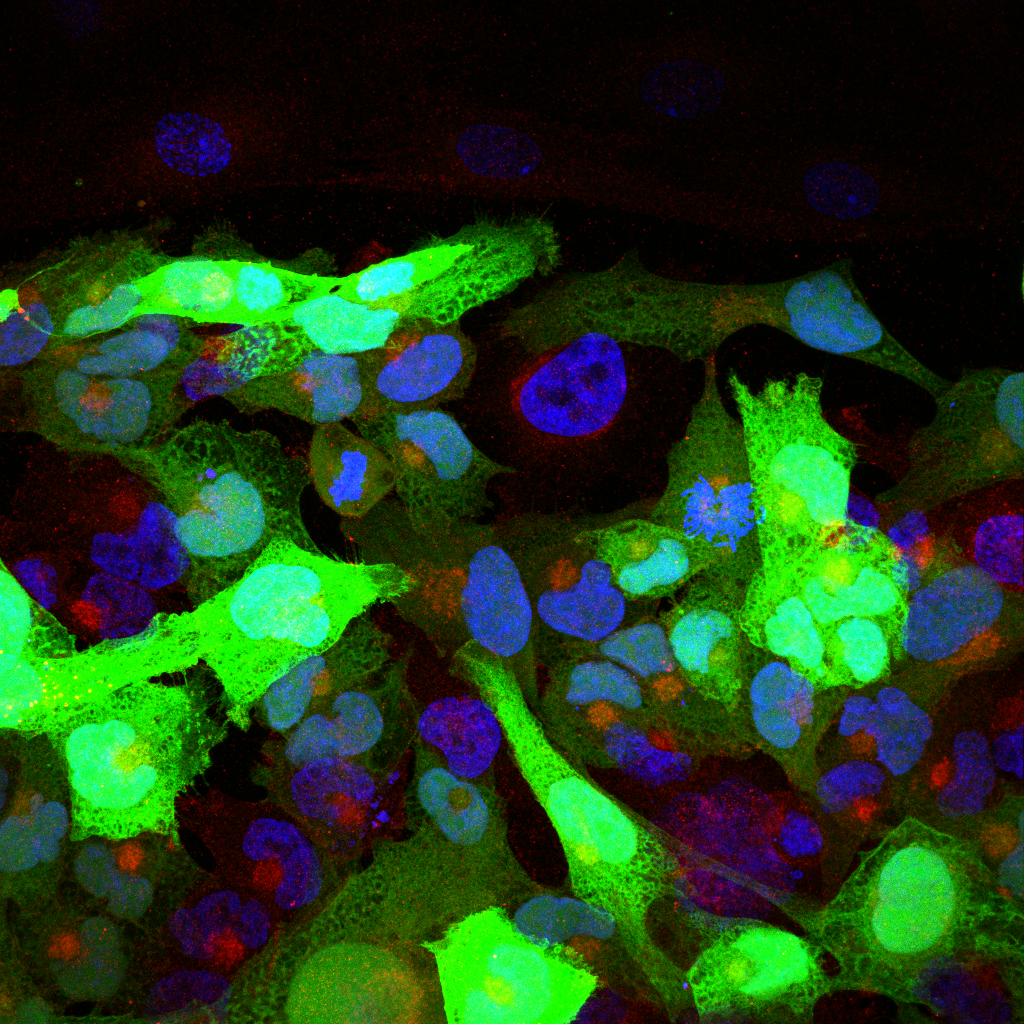

Supplement: Soure data 2. [file elife-32490-fig2.zip › Figure 4/Panel a/Bowes_LEC/LEC_Bowes_MMP14_594_Max_c1+2+3.tif]

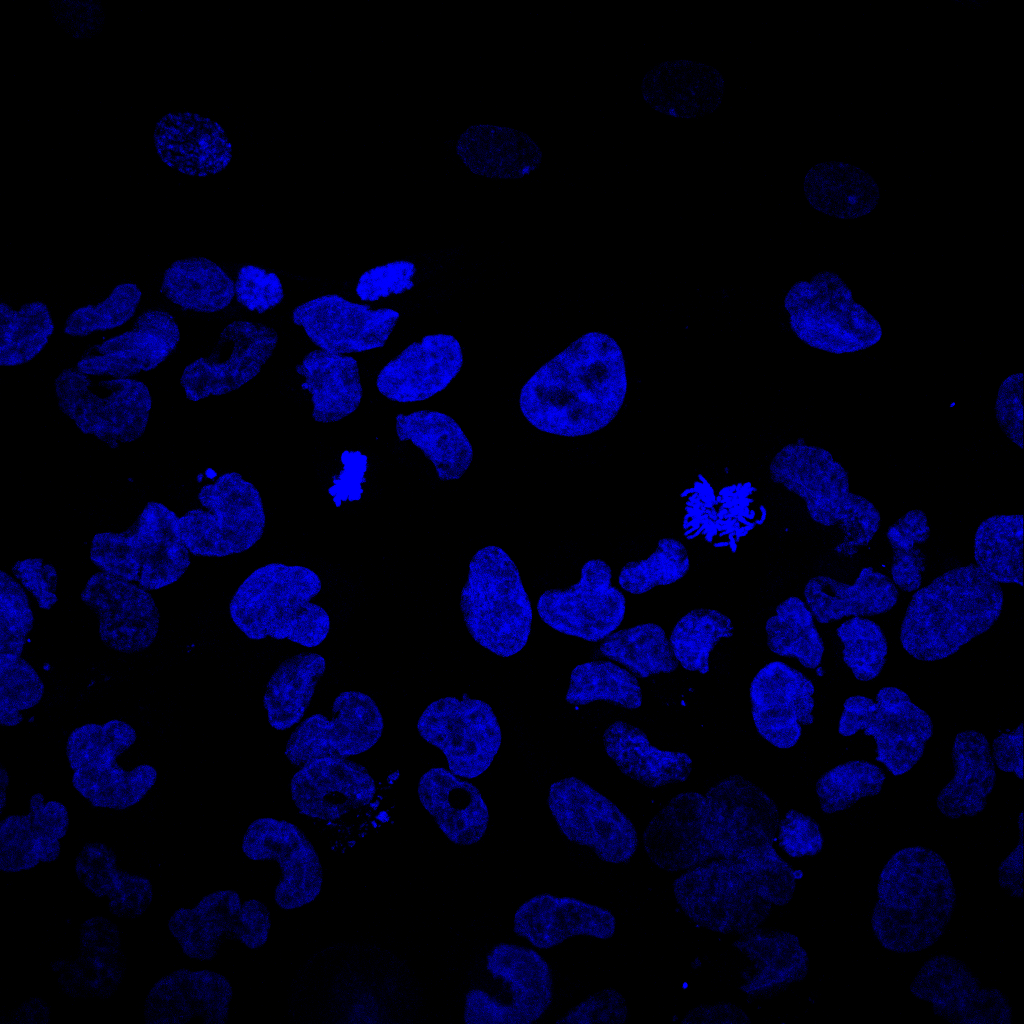

Supplement: Soure data 2. [file elife-32490-fig2.zip › Figure 4/Panel a/Bowes_LEC/LEC_Bowes_MMP14_594_Max_c2.tif]

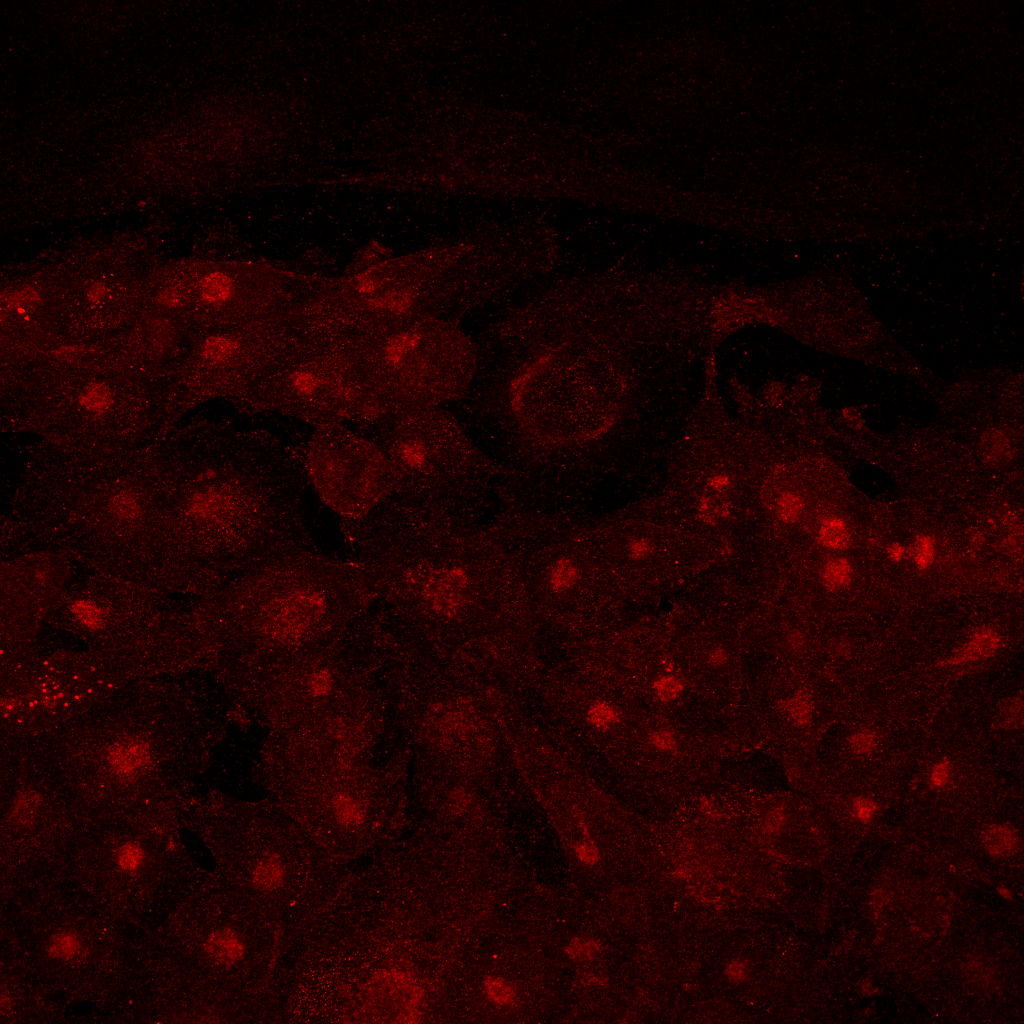

Supplement: Soure data 2. [file elife-32490-fig2.zip › Figure 4/Panel a/Bowes_LEC/LEC_Bowes_MMP14_594_Max_c3.tif]

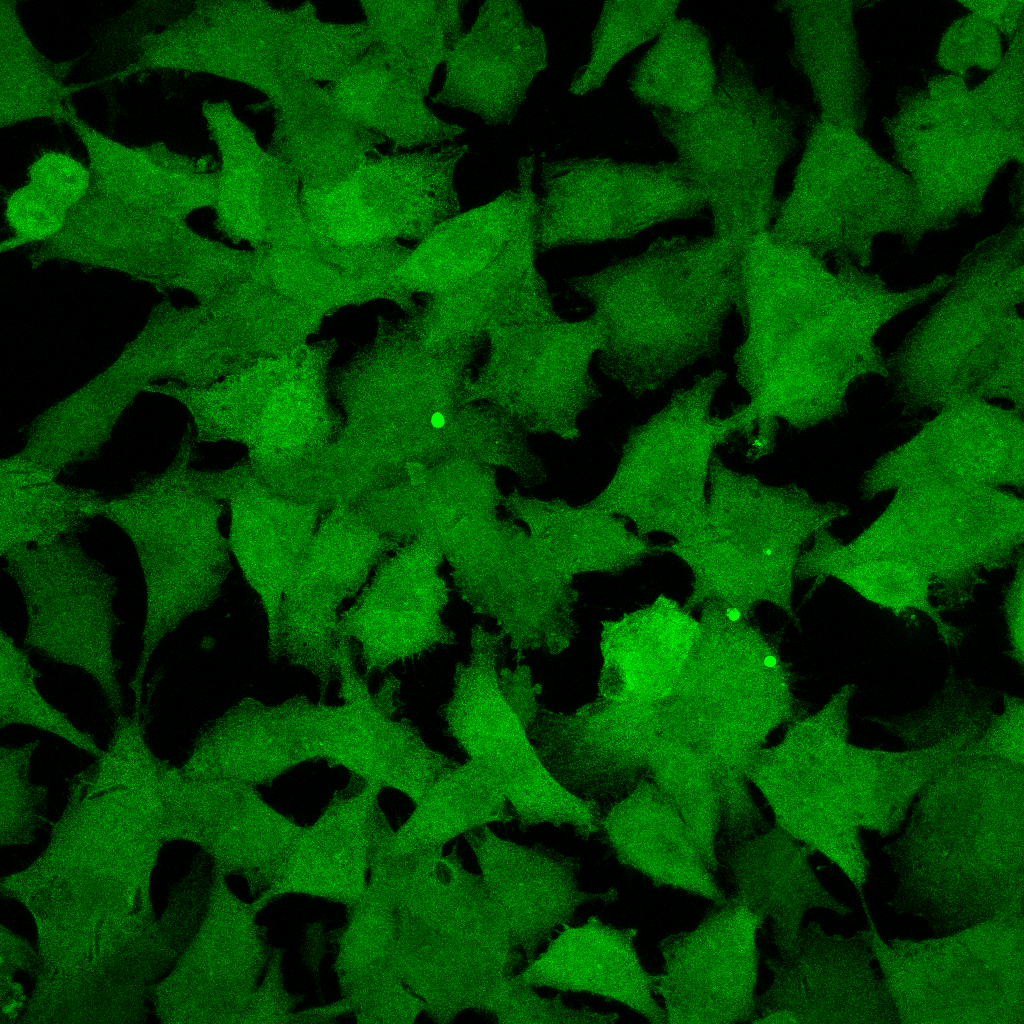

Supplement: Soure data 2. [file elife-32490-fig2.zip › Figure 4/Panel a/WM852/WM852_MMP14_594_Max_c1.tif]

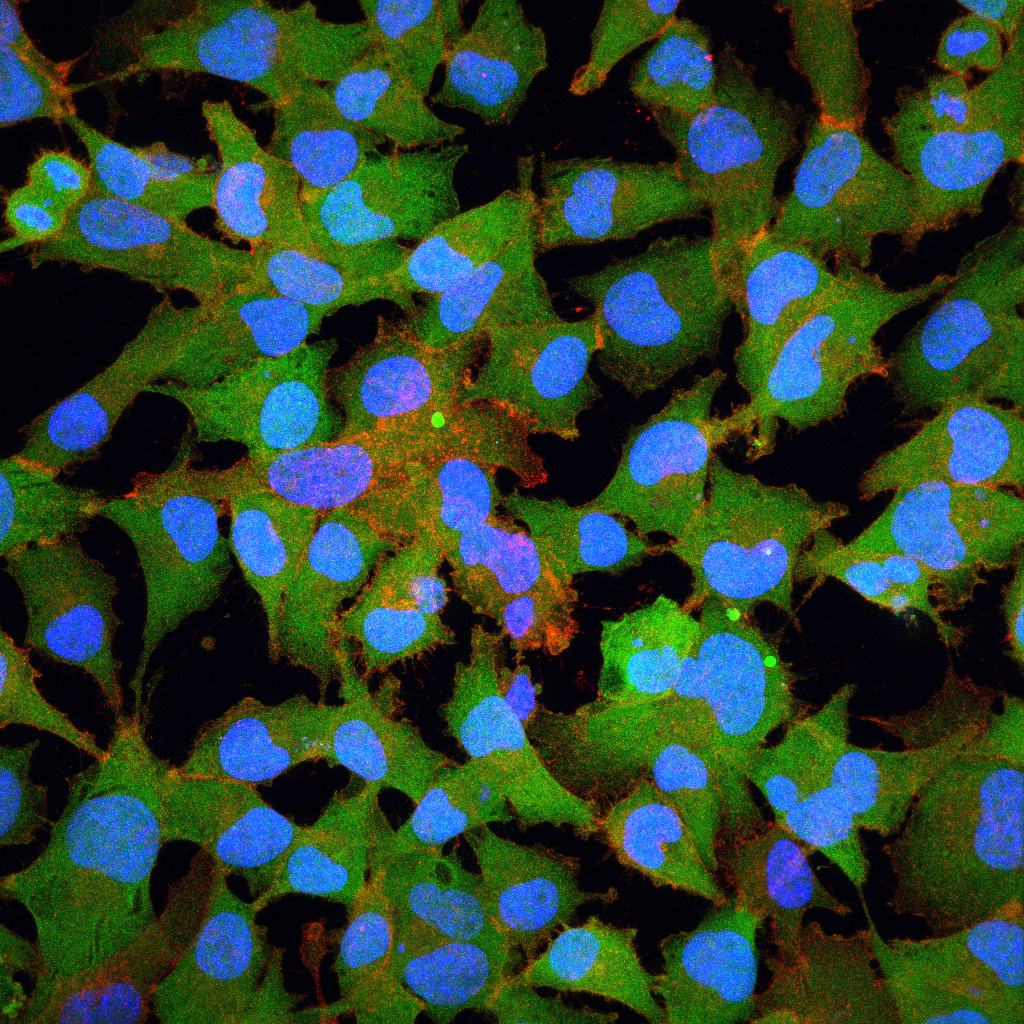

Supplement: Soure data 2. [file elife-32490-fig2.zip › Figure 4/Panel a/WM852/WM852_MMP14_594_Max_c1+2+3.tif]

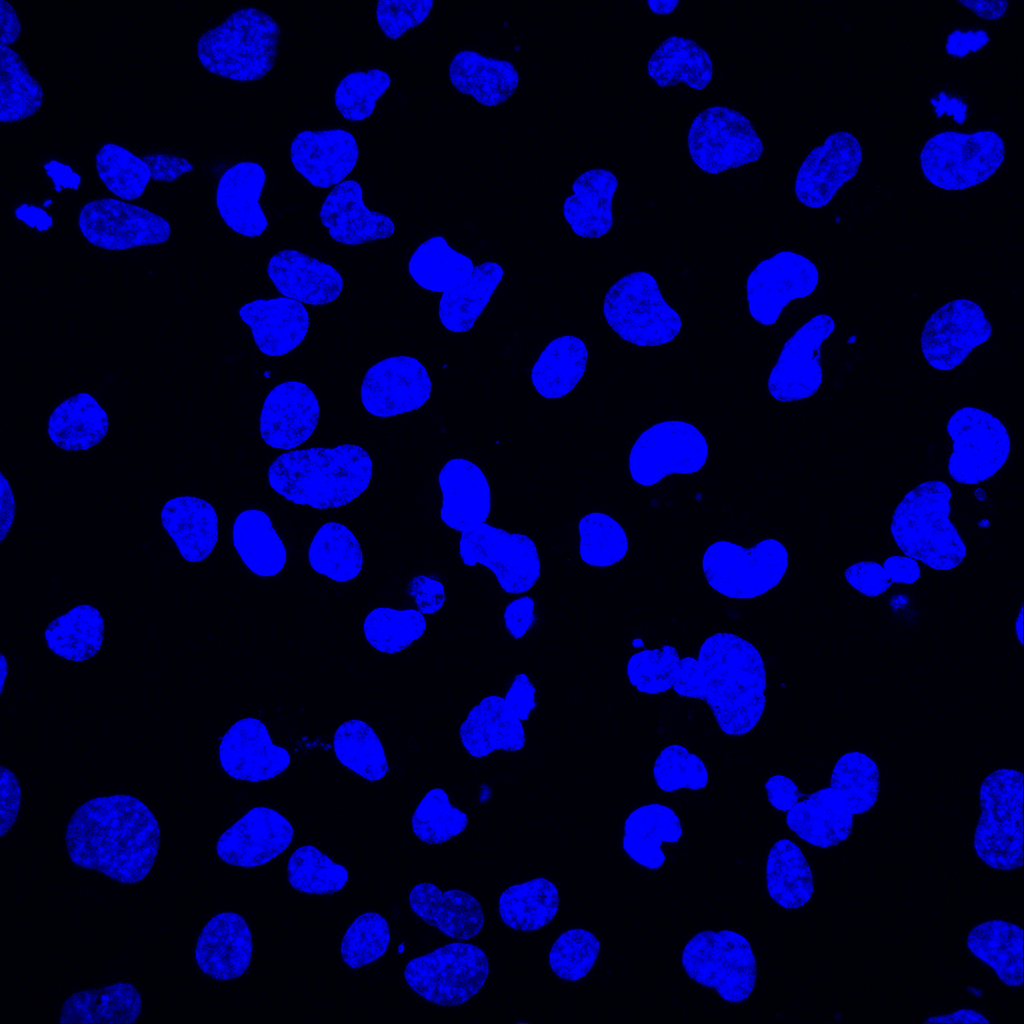

Supplement: Soure data 2. [file elife-32490-fig2.zip › Figure 4/Panel a/WM852/WM852_MMP14_594_Max_c2.tif]

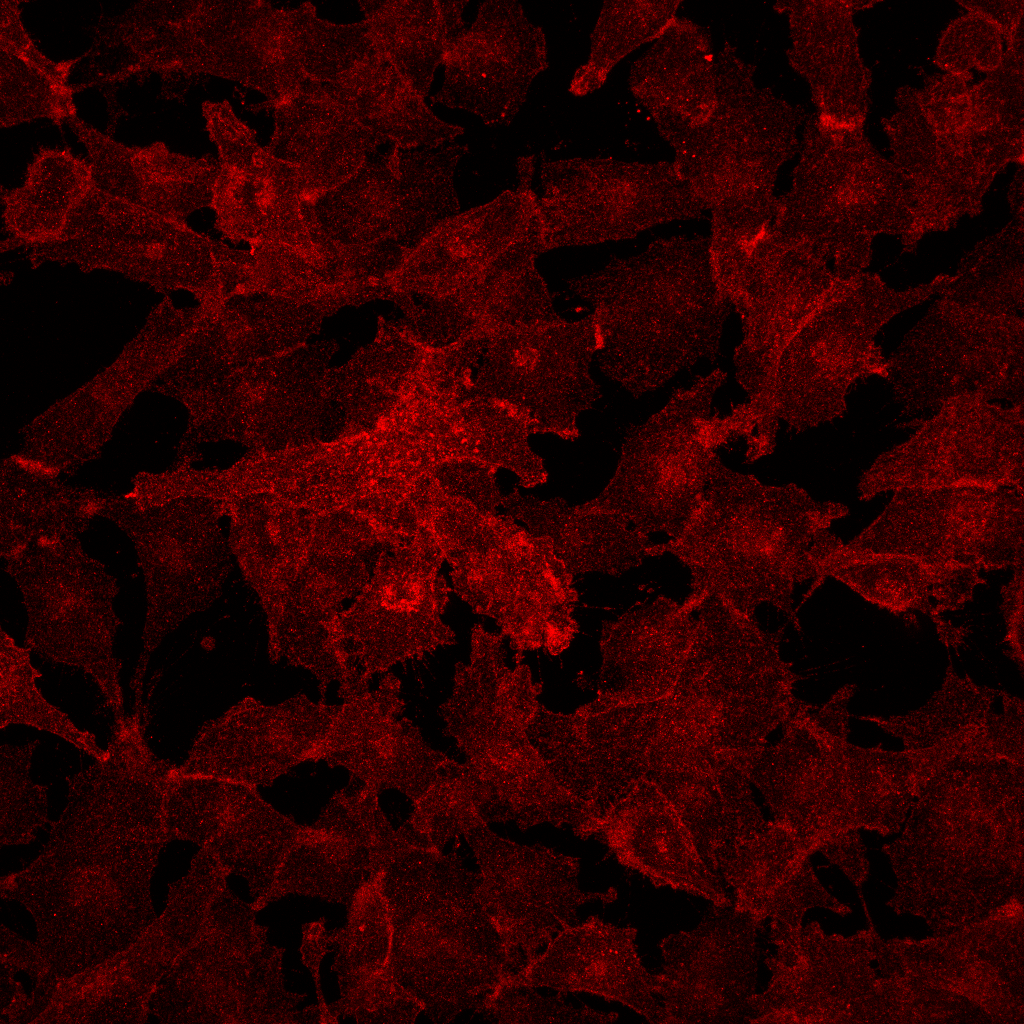

Supplement: Soure data 2. [file elife-32490-fig2.zip › Figure 4/Panel a/WM852/WM852_MMP14_594_Max_c3.tif]

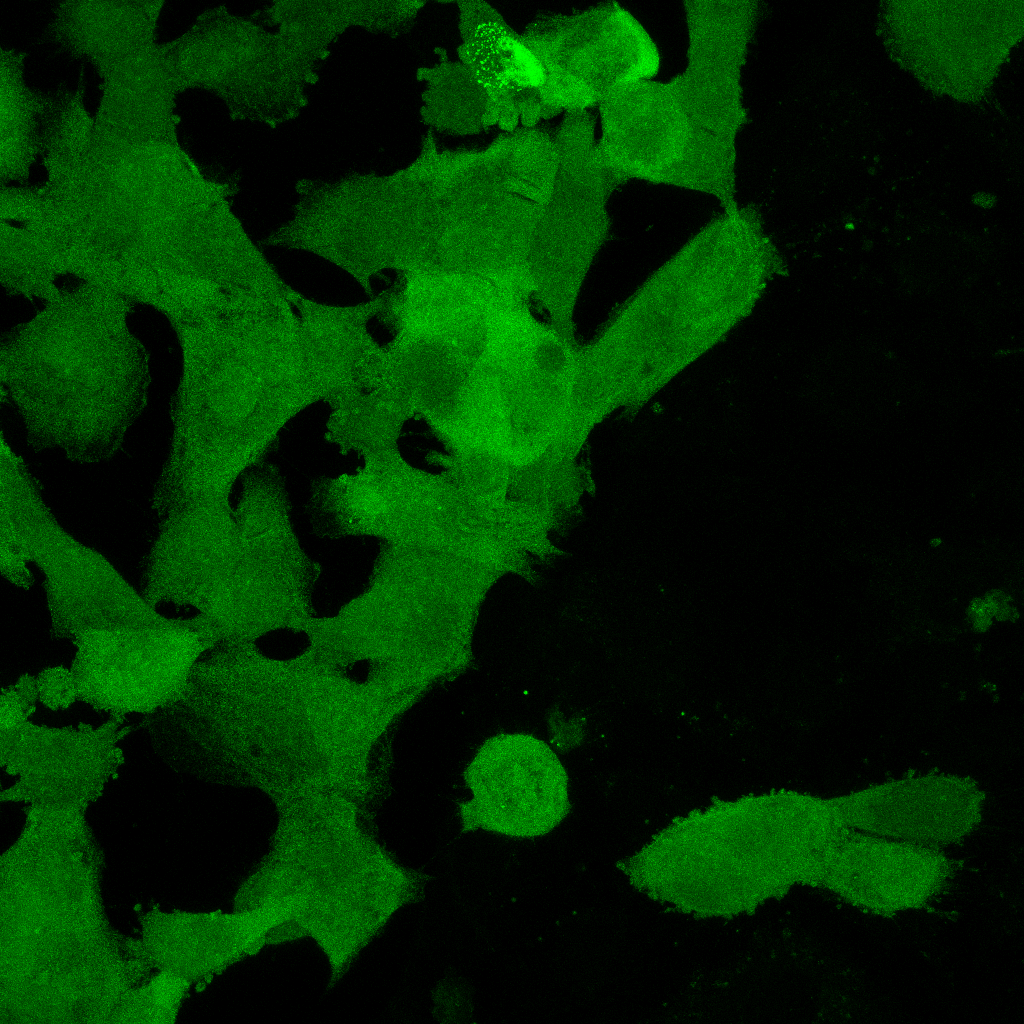

Supplement: Soure data 2. [file elife-32490-fig2.zip › Figure 4/Panel a/WM852_LEC/LEC_WM852_MMP14_594_Max_c1.tif]

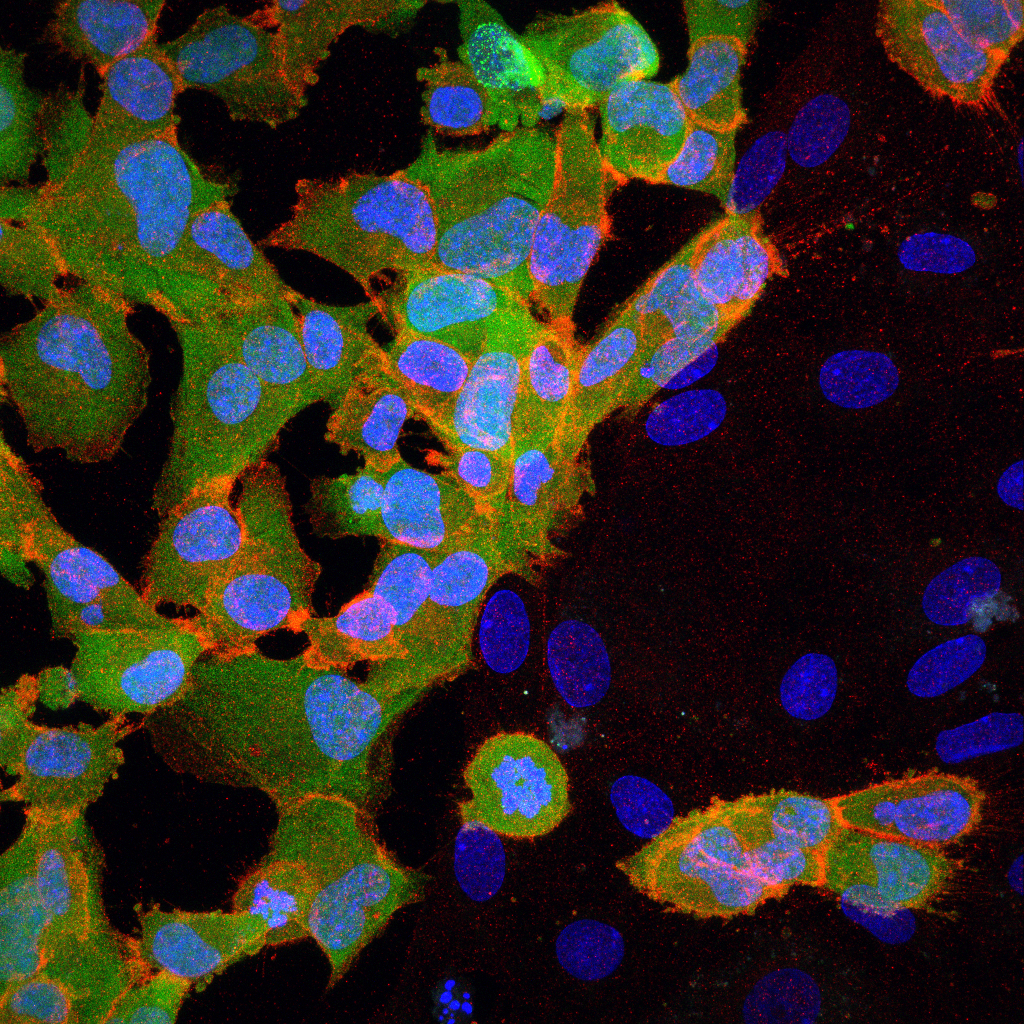

Supplement: Soure data 2. [file elife-32490-fig2.zip › Figure 4/Panel a/WM852_LEC/LEC_WM852_MMP14_594_Max_c1+2+3.tif]

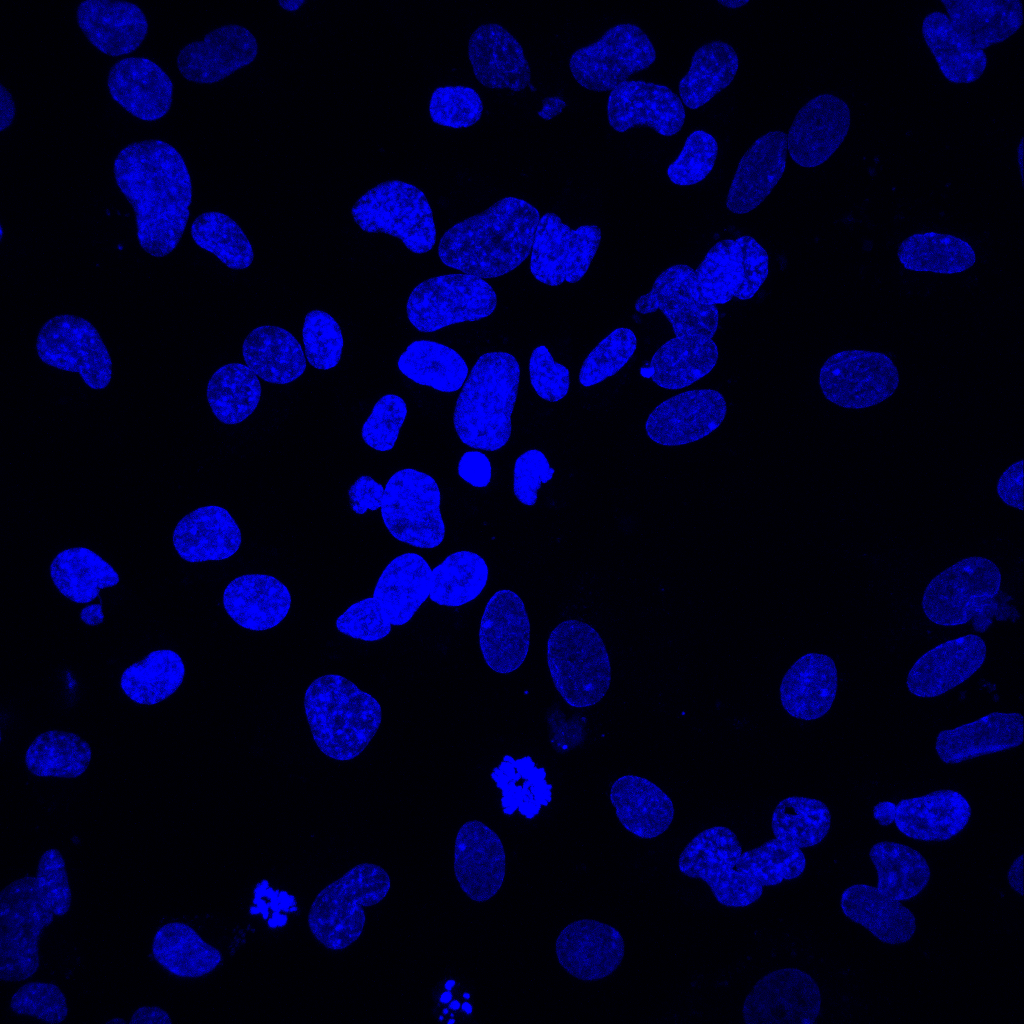

Supplement: Soure data 2. [file elife-32490-fig2.zip › Figure 4/Panel a/WM852_LEC/LEC_WM852_MMP14_594_Max_c2.tif]

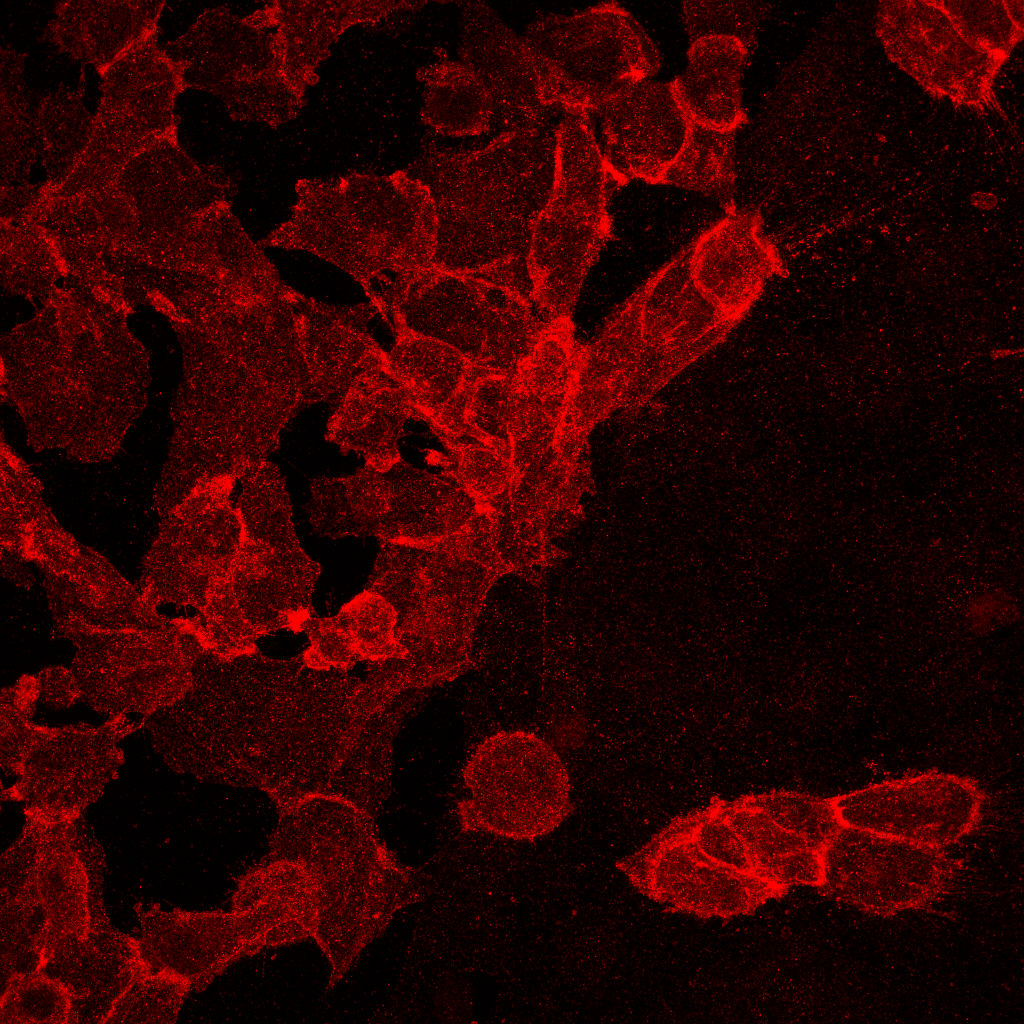

Supplement: Soure data 2. [file elife-32490-fig2.zip › Figure 4/Panel a/WM852_LEC/LEC_WM852_MMP14_594_Max_c3.tif]

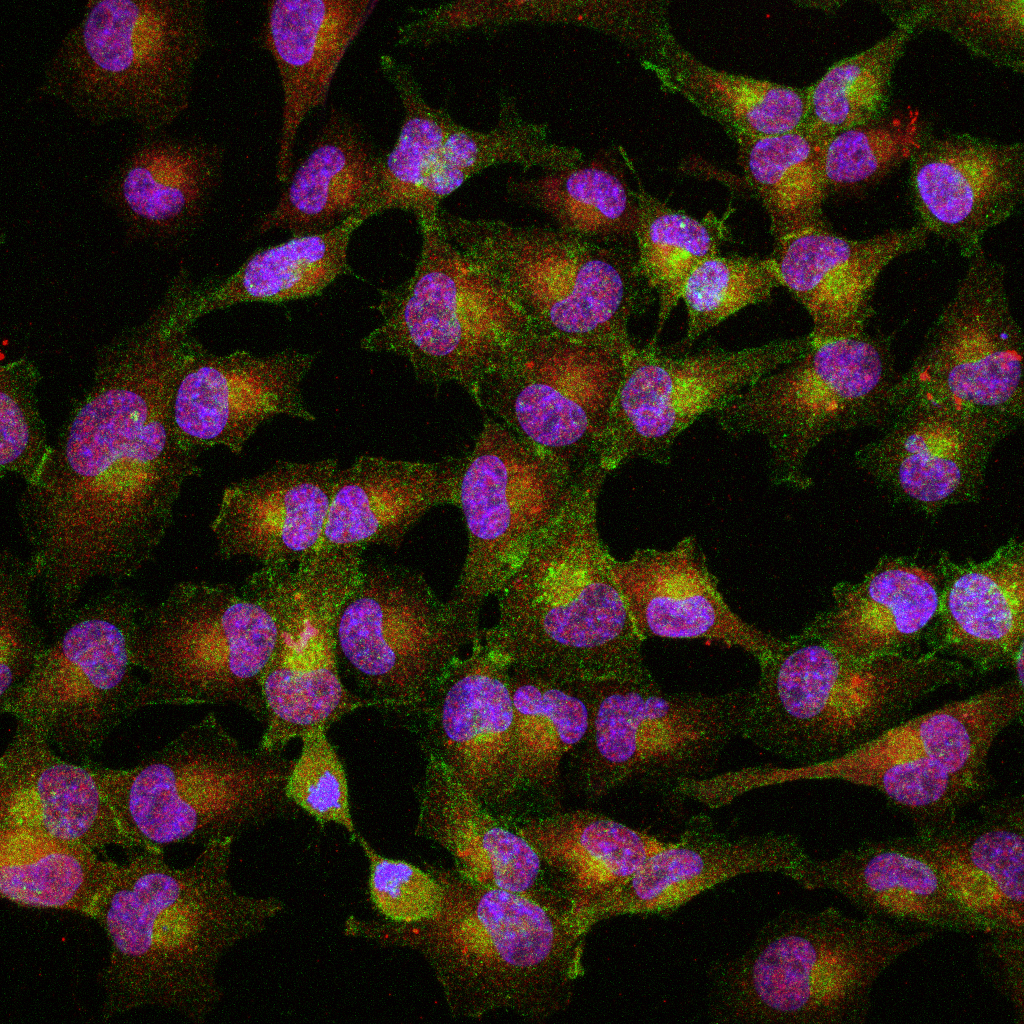

Supplement: Soure data 2. [file elife-32490-fig2.zip › Figure 4/Panel c/WM842/WM852_Notch3_594_MMP14_647_Maximum intensity projection_c2+3+4.tif]

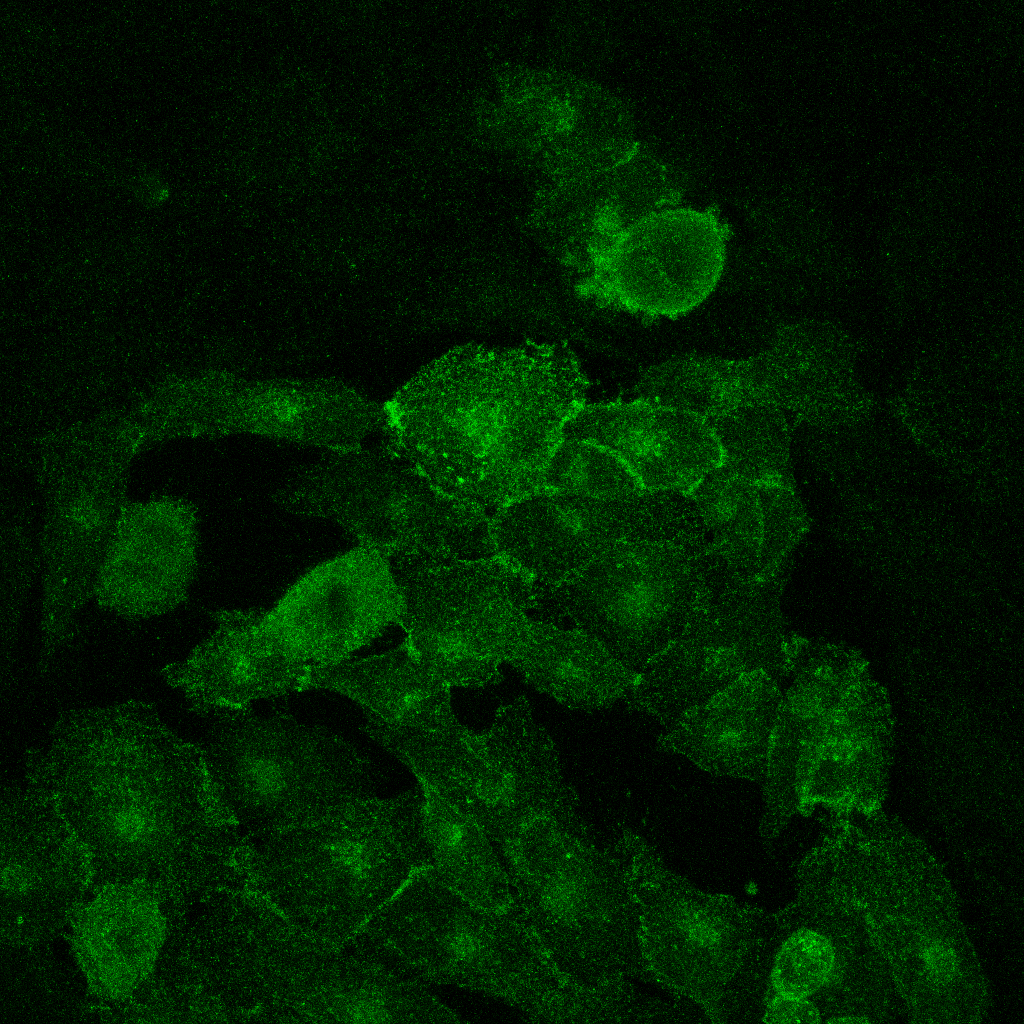

Supplement: Soure data 2. [file elife-32490-fig2.zip › Figure 4/Panel c/WM852_LEC/LEC_WM852_Notch3_594_MMP14_647_Maximum intensity projection_c2.tif]

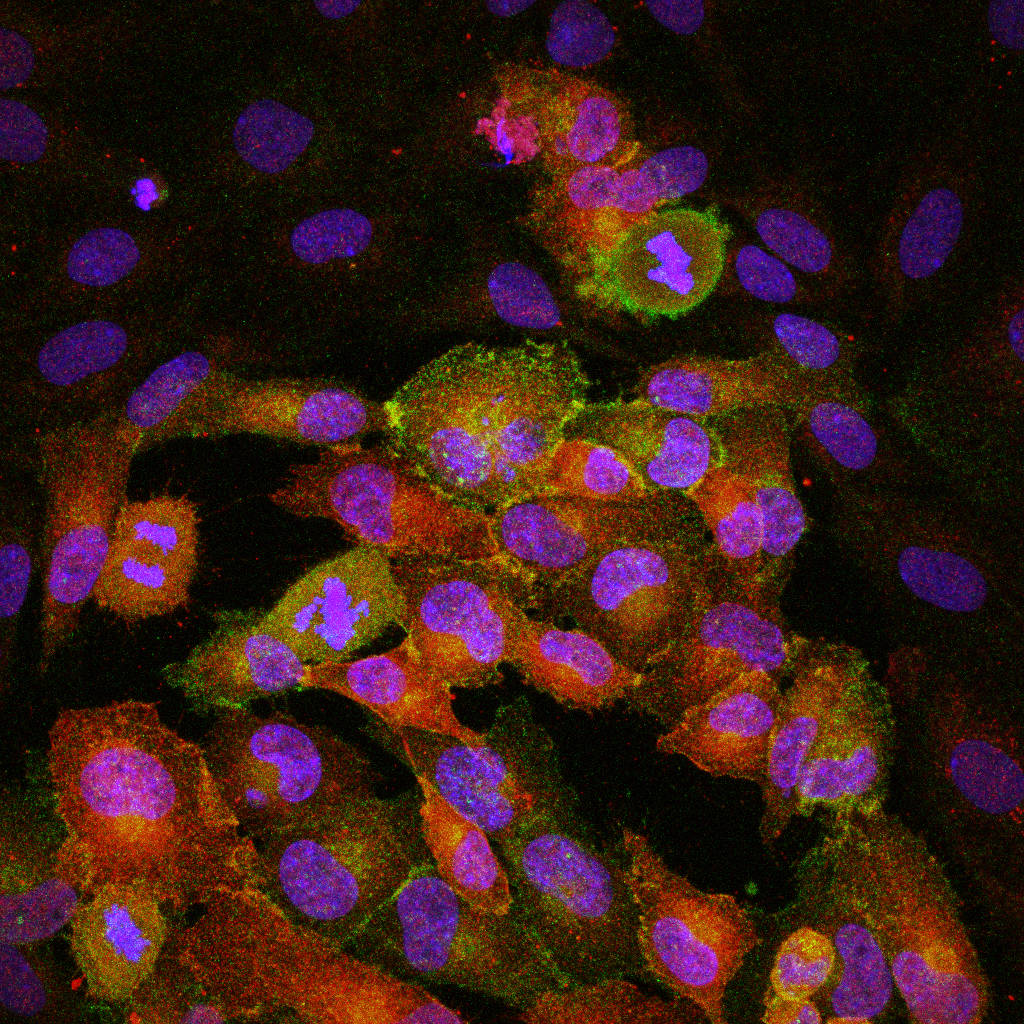

Supplement: Soure data 2. [file elife-32490-fig2.zip › Figure 4/Panel c/WM852_LEC/LEC_WM852_Notch3_594_MMP14_647_Maximum intensity projection_c2+3+4.tif]

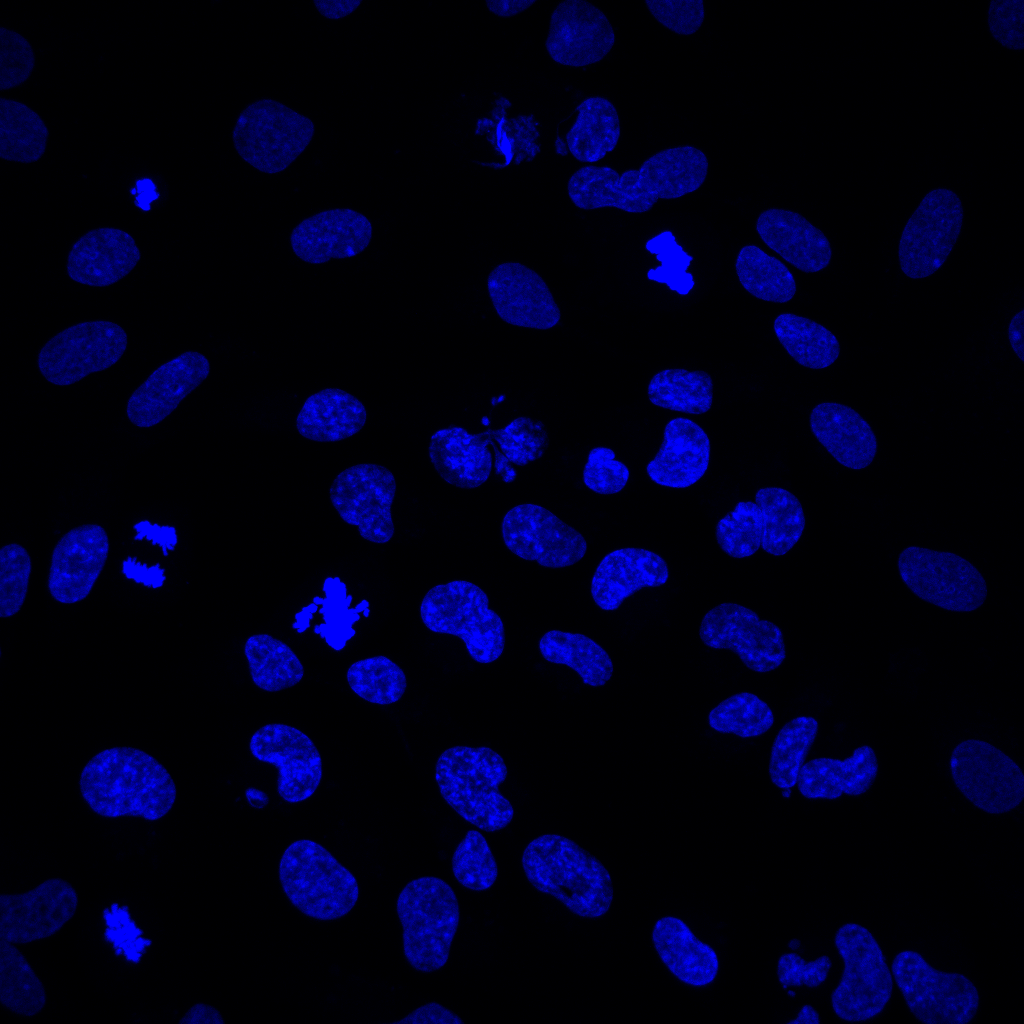

Supplement: Soure data 2. [file elife-32490-fig2.zip › Figure 4/Panel c/WM852_LEC/LEC_WM852_Notch3_594_MMP14_647_Maximum intensity projection_c3.tif]

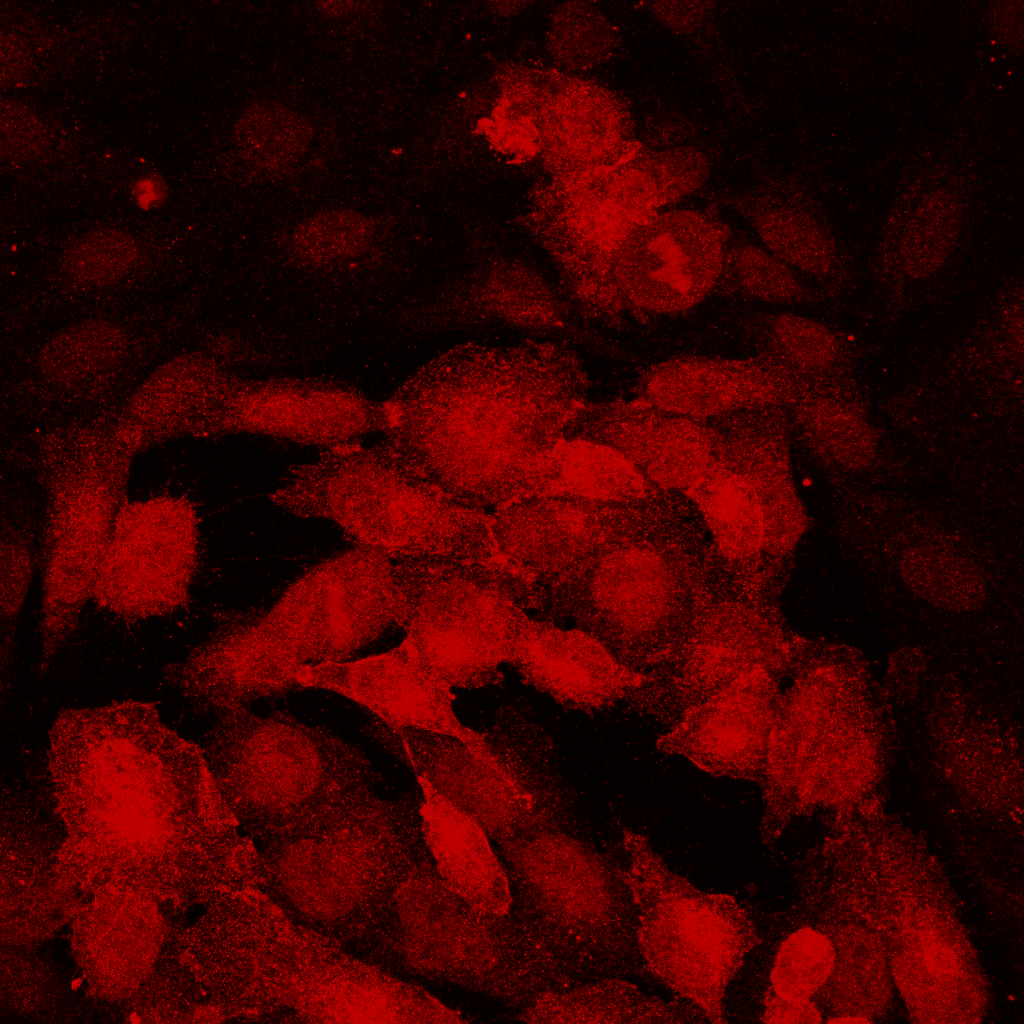

Supplement: Soure data 2. [file elife-32490-fig2.zip › Figure 4/Panel c/WM852_LEC/LEC_WM852_Notch3_594_MMP14_647_Maximum intensity projection_c4.tif]

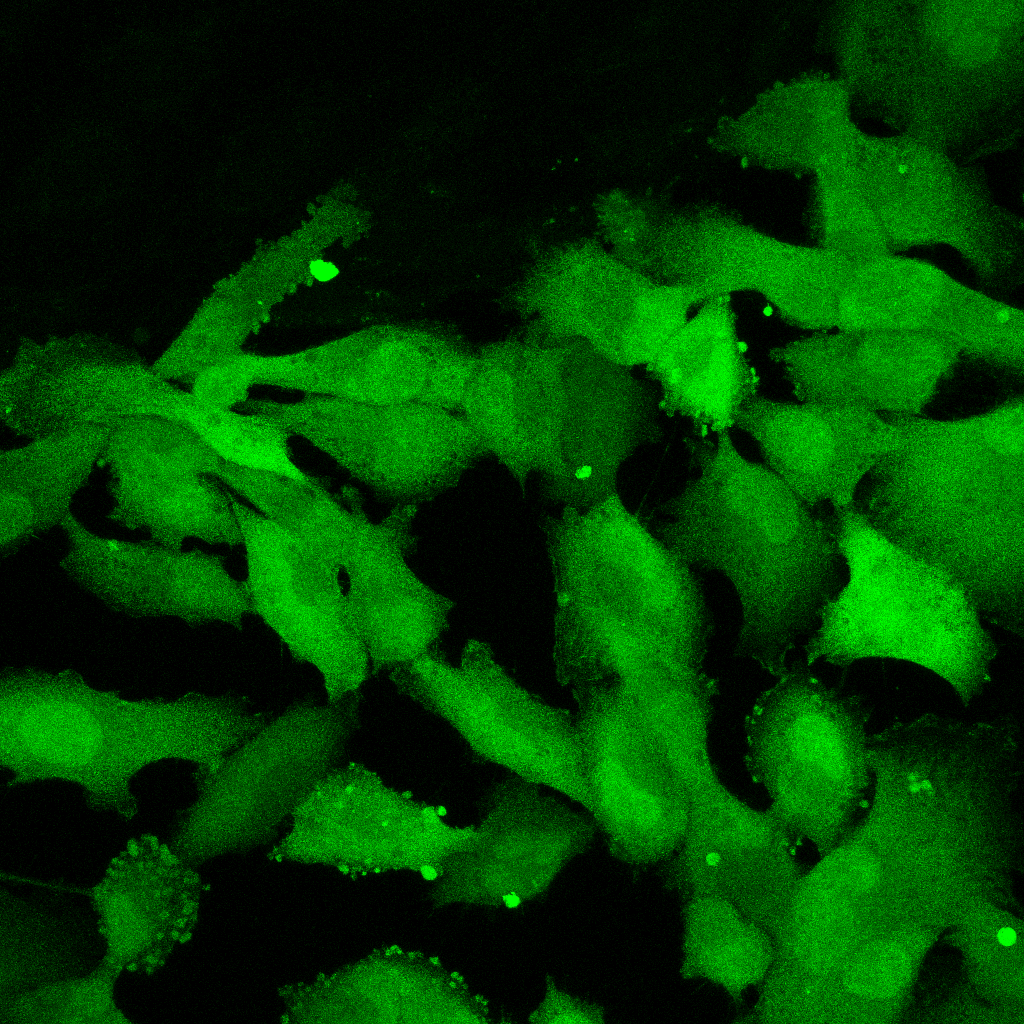

Supplement: Soure data 2. [file elife-32490-fig2.zip › Figure 4/Panel e/siCtrl/LEC_siSCR_Notch3_Maximum intensity projection_c1.tif]

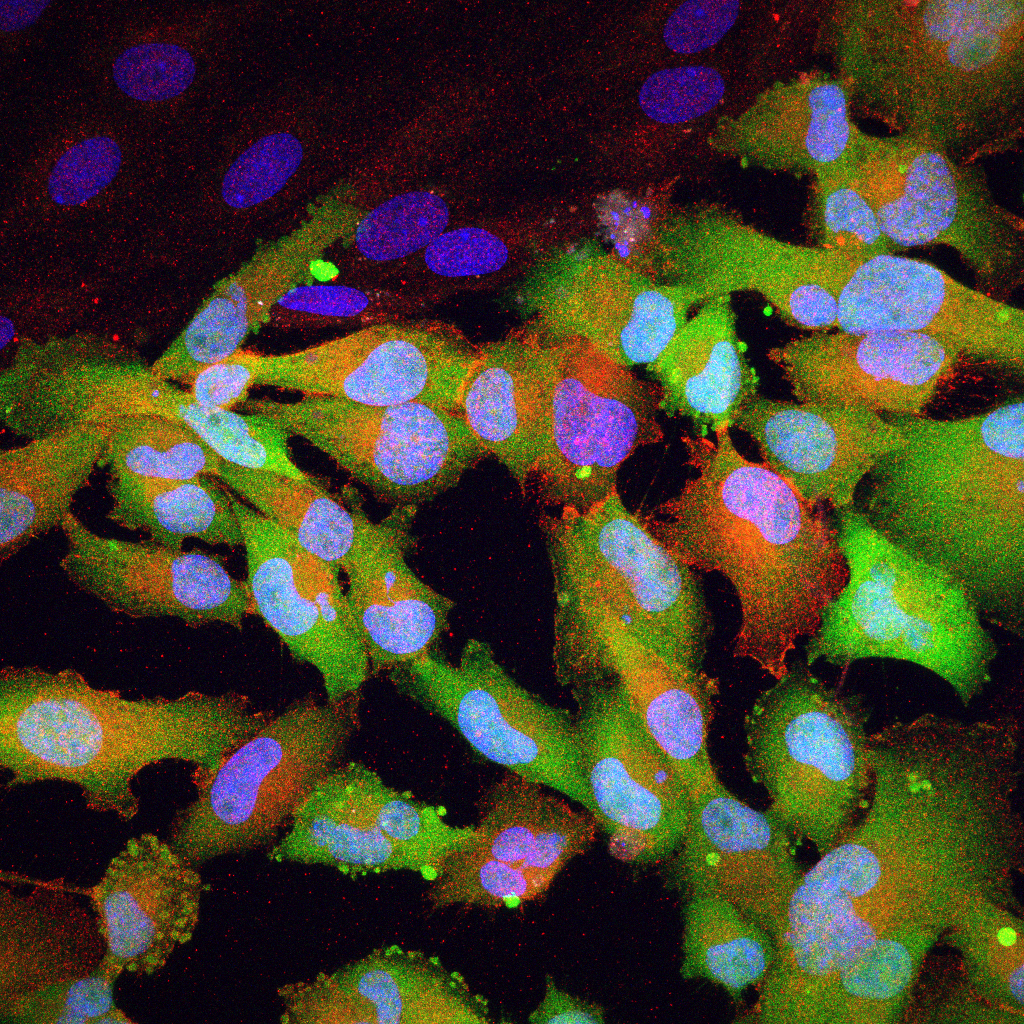

Supplement: Soure data 2. [file elife-32490-fig2.zip › Figure 4/Panel e/siCtrl/LEC_siSCR_Notch3_Maximum intensity projection_c1+2+3.tif]

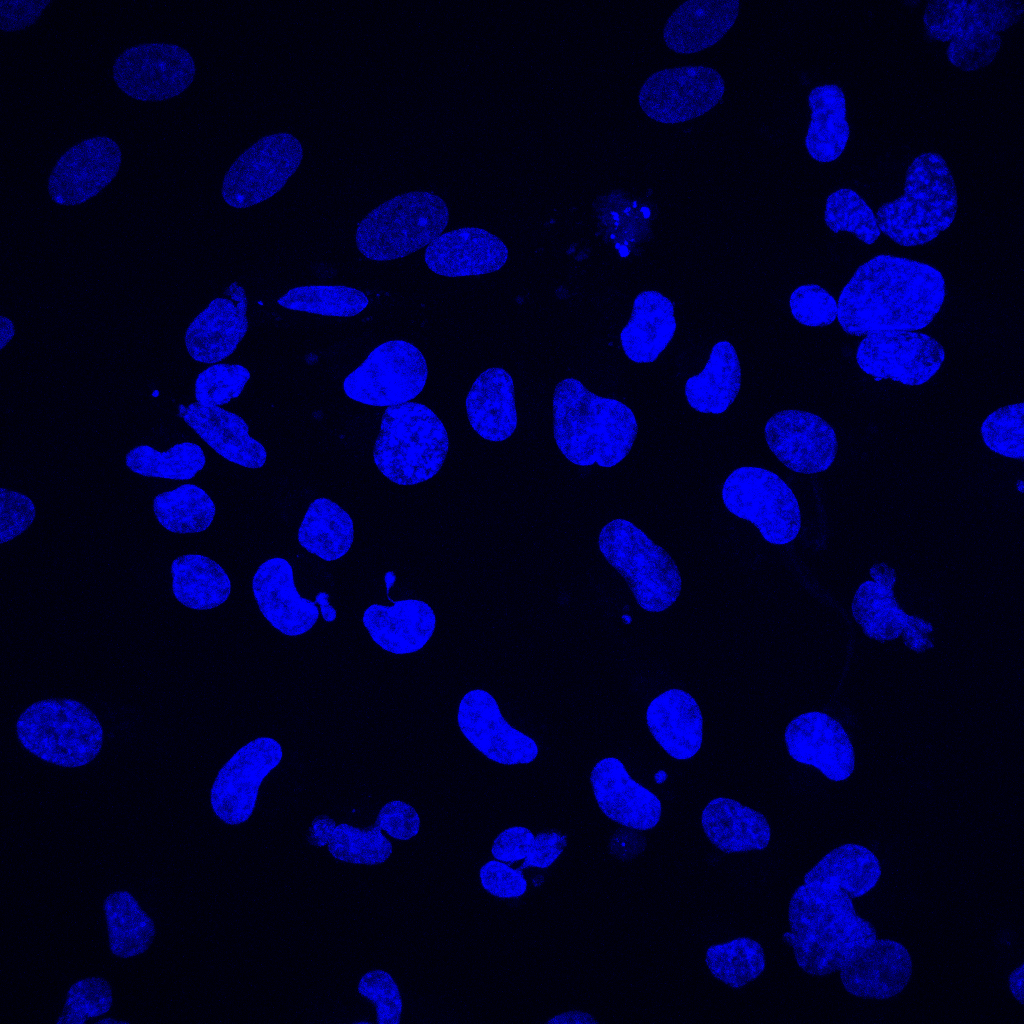

Supplement: Soure data 2. [file elife-32490-fig2.zip › Figure 4/Panel e/siCtrl/LEC_siSCR_Notch3_Maximum intensity projection_c2.tif]

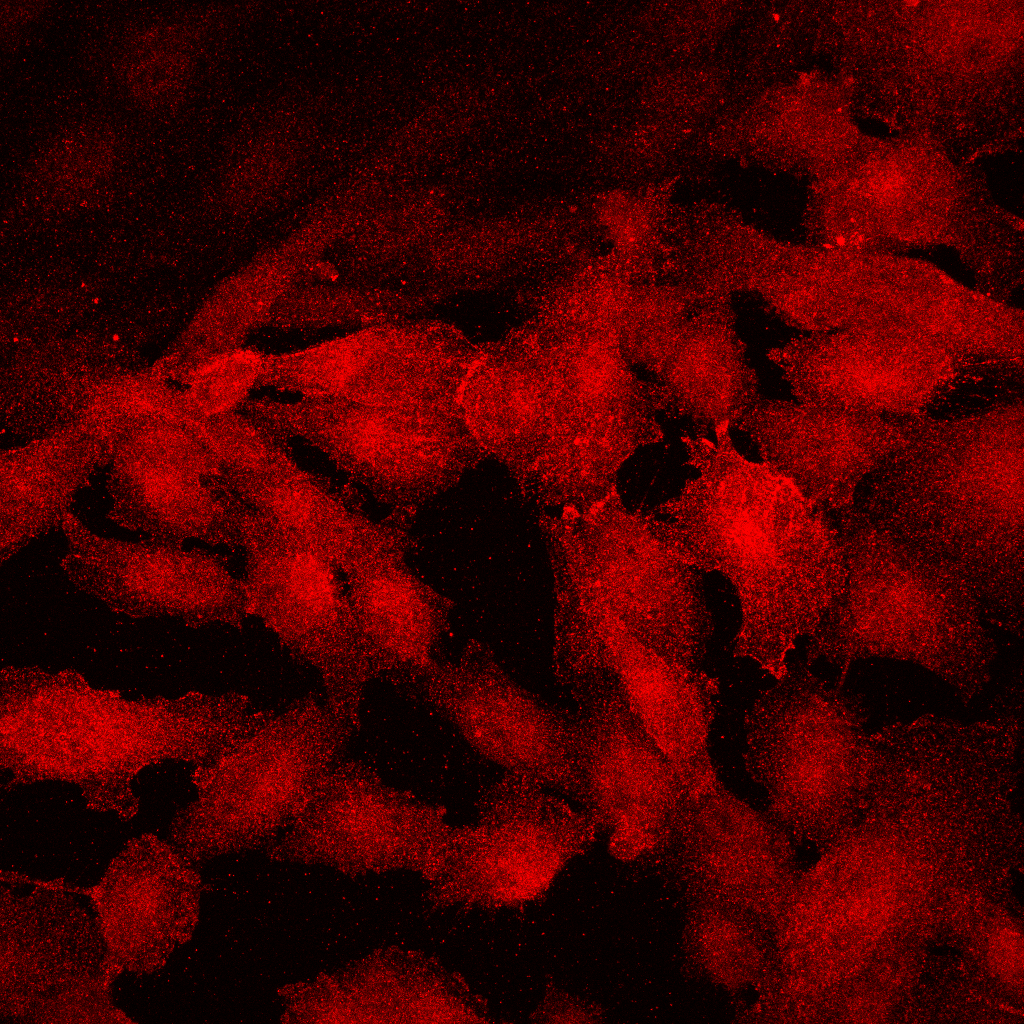

Supplement: Soure data 2. [file elife-32490-fig2.zip › Figure 4/Panel e/siCtrl/LEC_siSCR_Notch3_Maximum intensity projection_c3.tif]

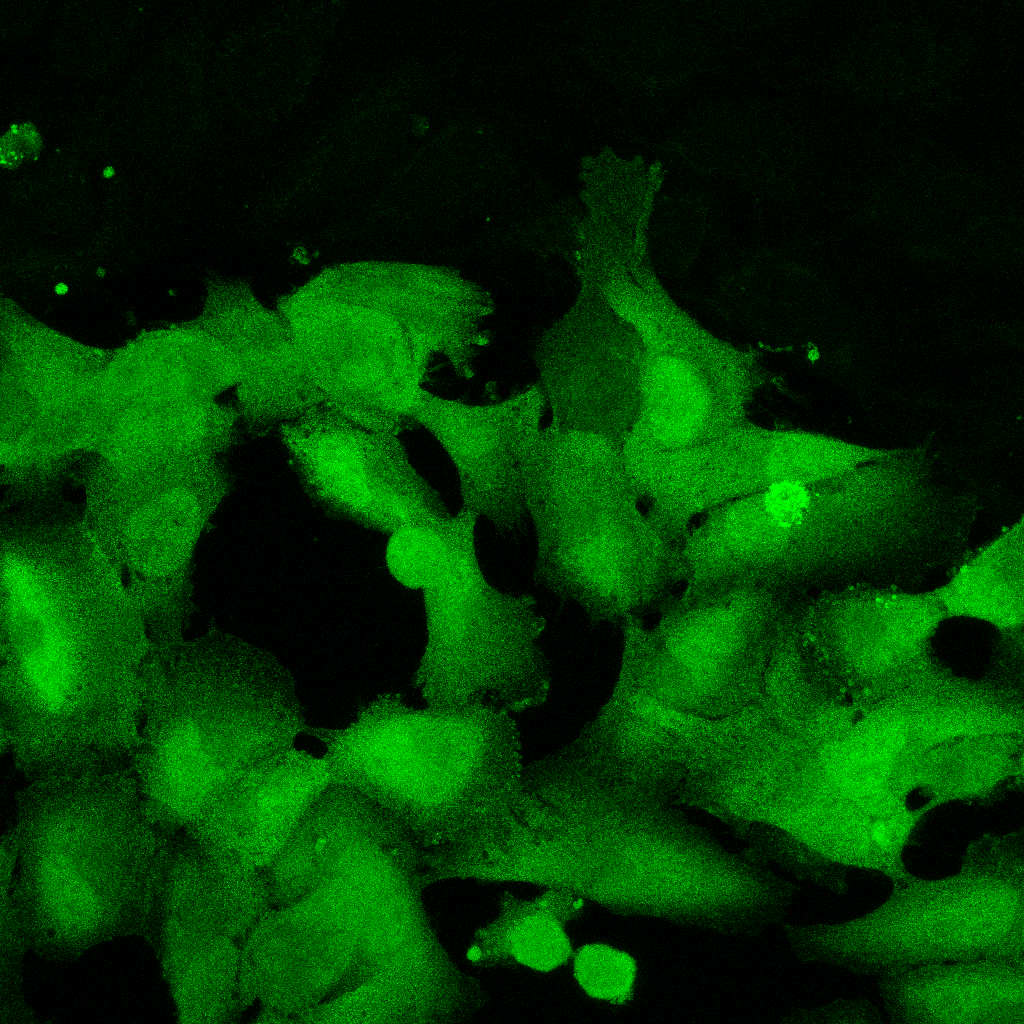

Supplement: Soure data 2. [file elife-32490-fig2.zip › Figure 4/Panel e/siMMP14/LEC_siMMP14_Notch3_Maximum intensity projection_c1.tif]

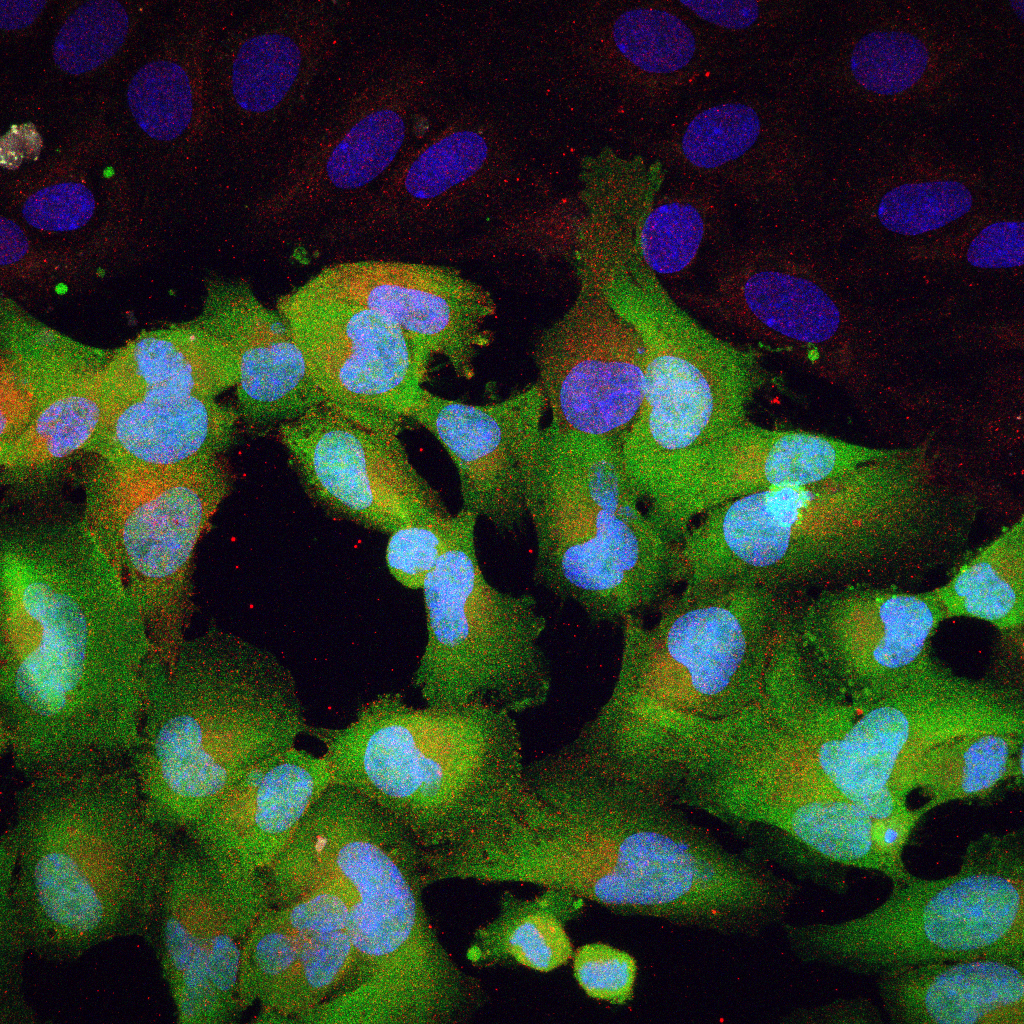

Supplement: Soure data 2. [file elife-32490-fig2.zip › Figure 4/Panel e/siMMP14/LEC_siMMP14_Notch3_Maximum intensity projection_c1+2+3.tif]

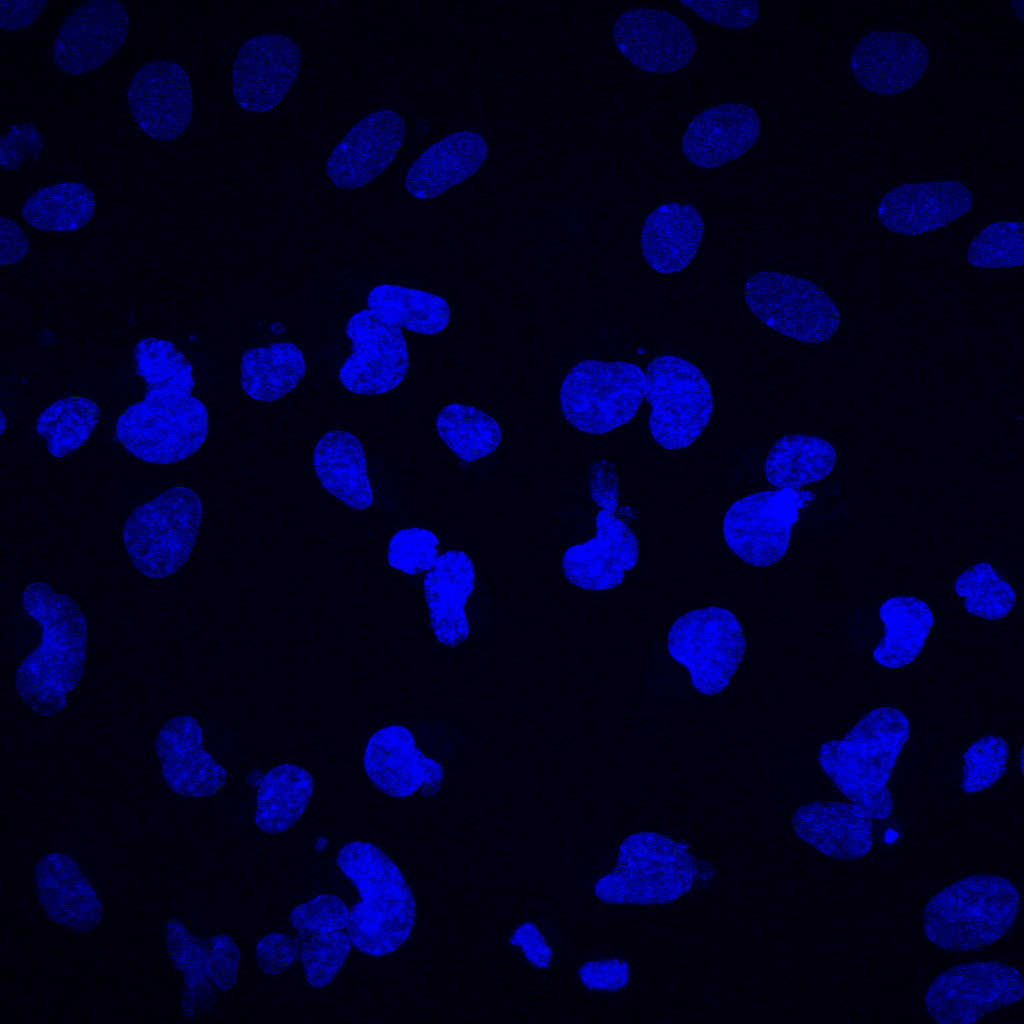

Supplement: Soure data 2. [file elife-32490-fig2.zip › Figure 4/Panel e/siMMP14/LEC_siMMP14_Notch3_Maximum intensity projection_c2.tif]

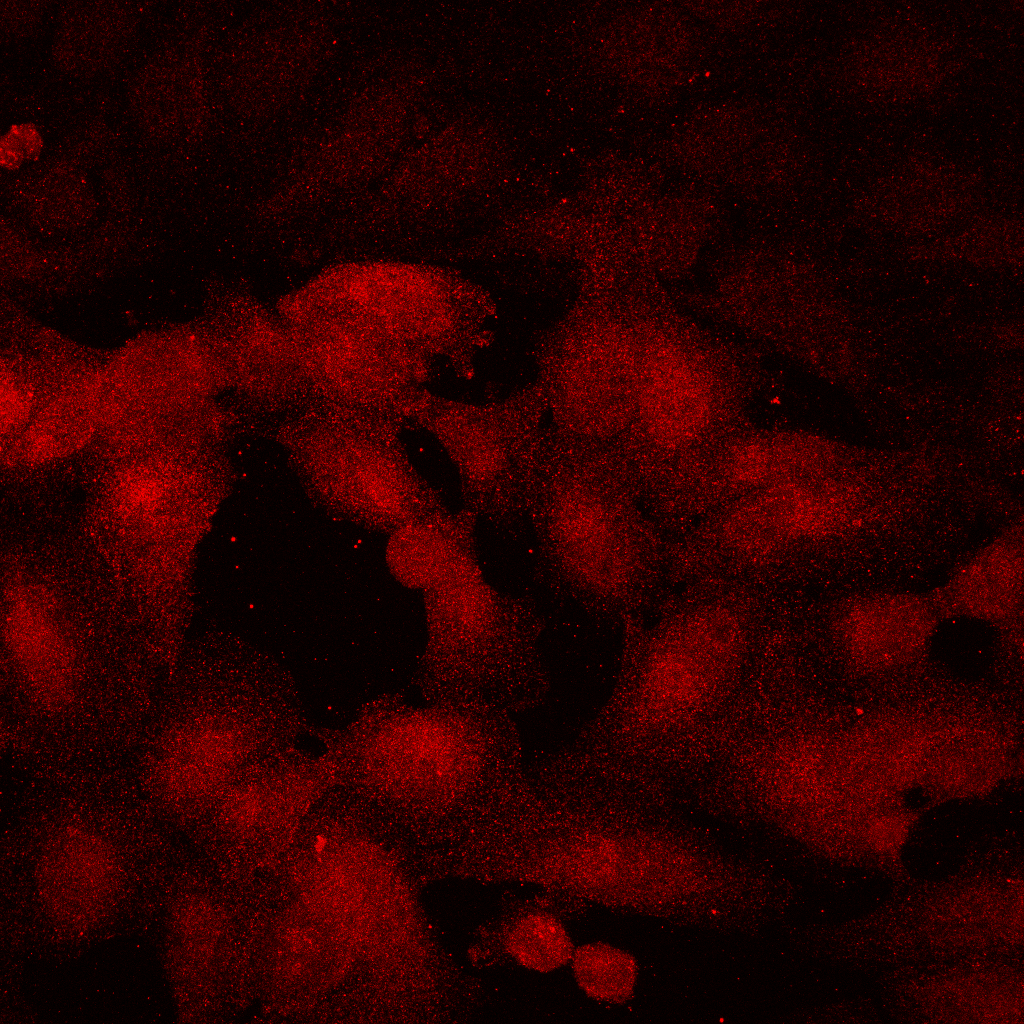

Supplement: Soure data 2. [file elife-32490-fig2.zip › Figure 4/Panel e/siMMP14/LEC_siMMP14_Notch3_Maximum intensity projection_c3.tif]

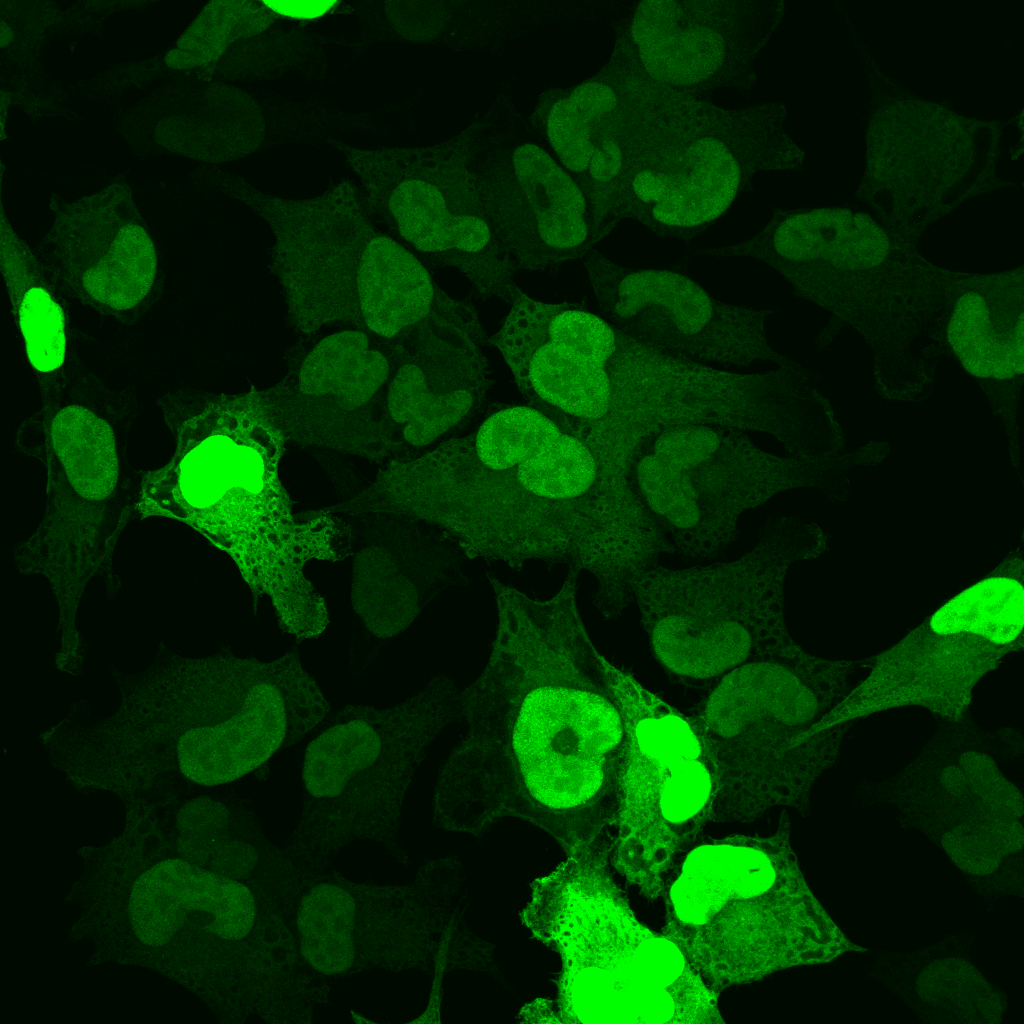

Supplement: Soure data 3. [file elife-32490-fig3.zip › Figure 5/Panel a/Bowes/Bowes_activeITGB1_594_Max_c1.tif]

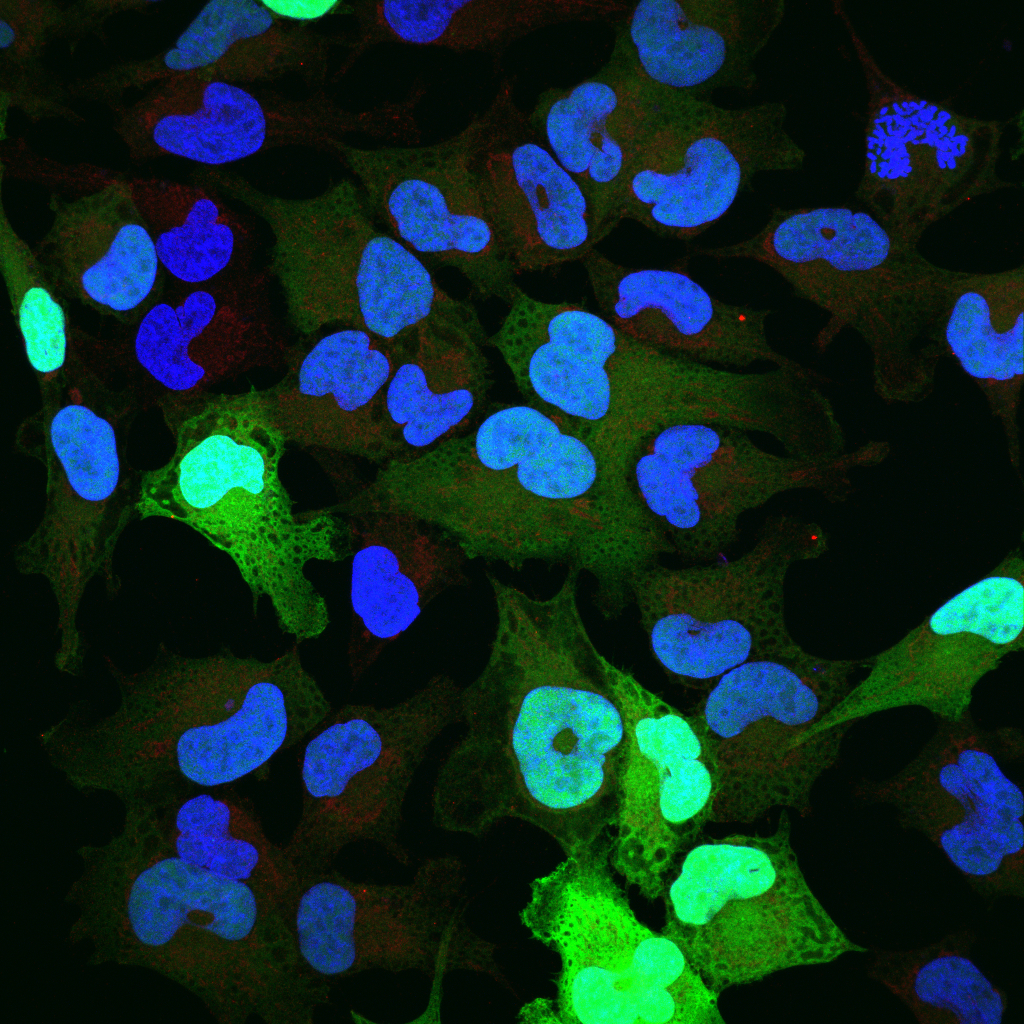

Supplement: Soure data 3. [file elife-32490-fig3.zip › Figure 5/Panel a/Bowes/Bowes_activeITGB1_594_Max_c1+2+3.tif]

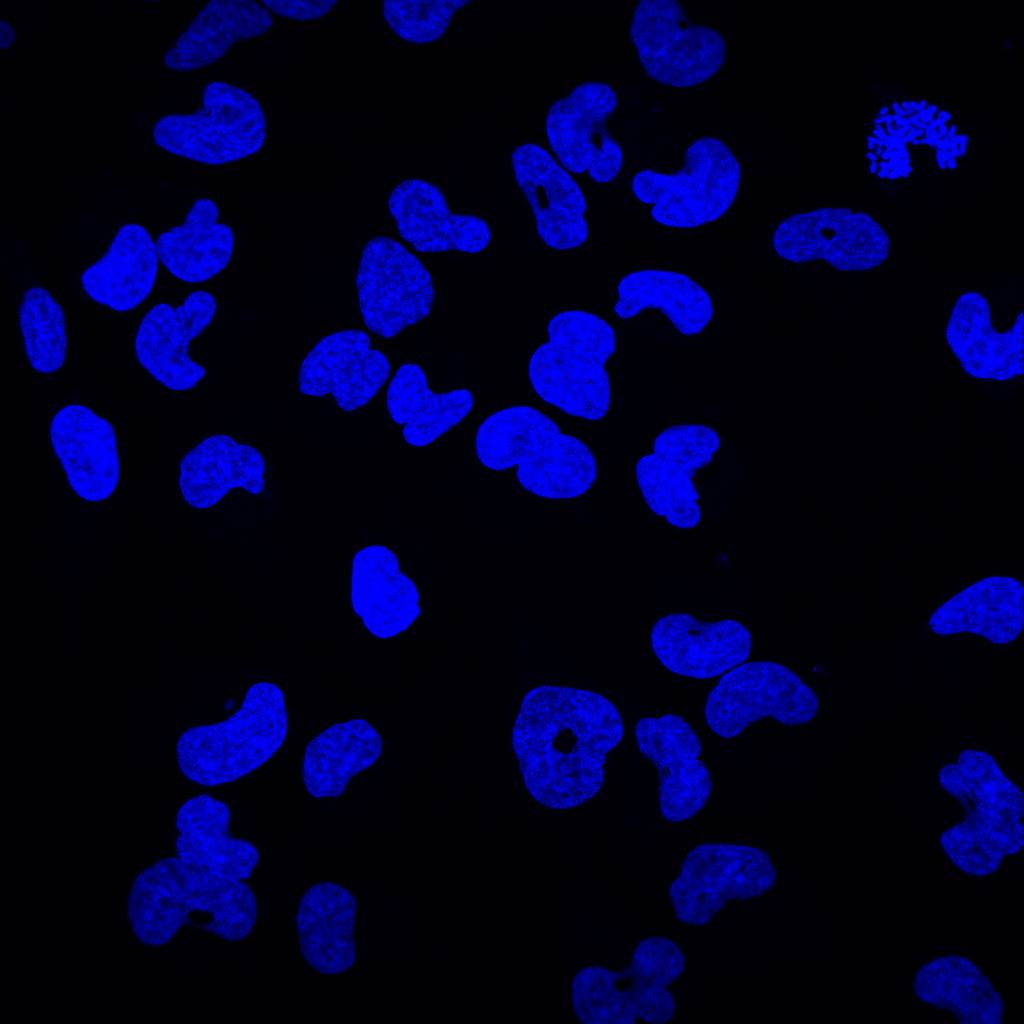

Supplement: Soure data 3. [file elife-32490-fig3.zip › Figure 5/Panel a/Bowes/Bowes_activeITGB1_594_Max_c2.tif]

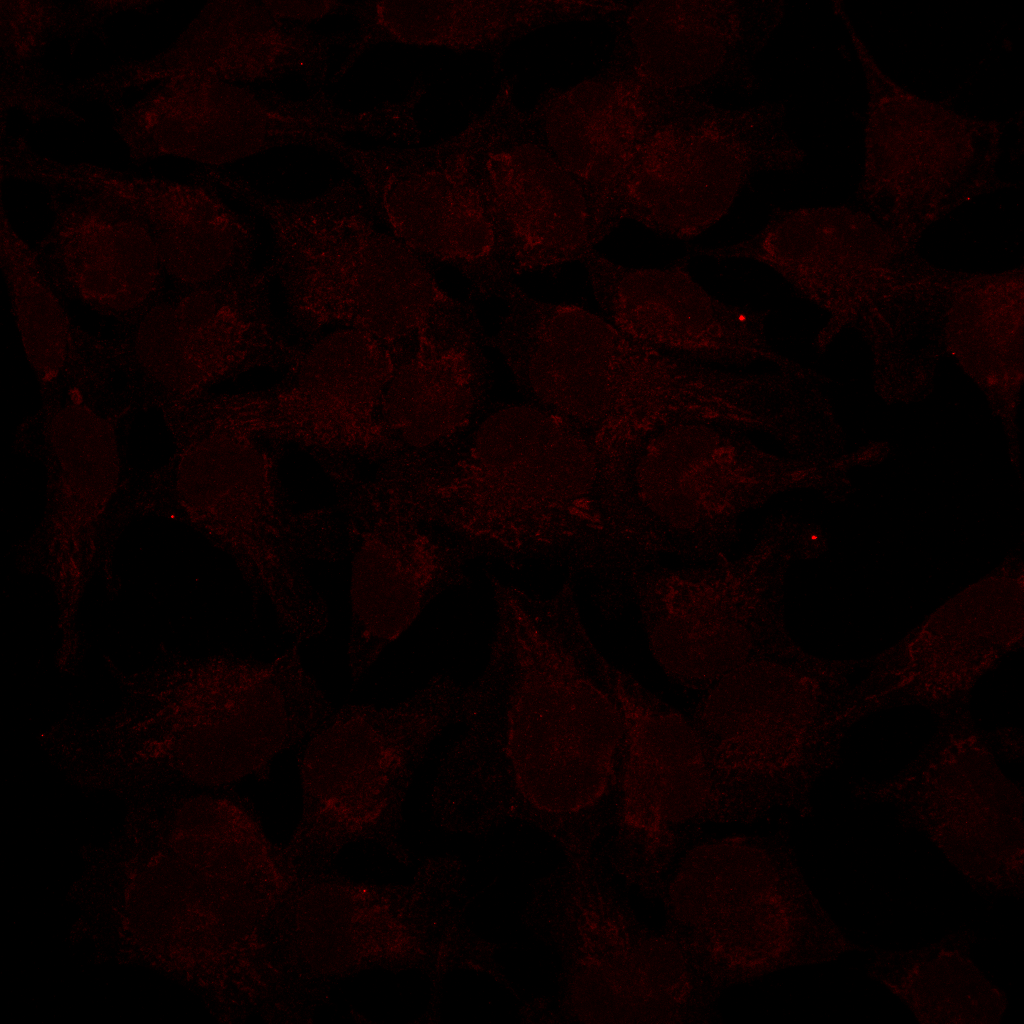

Supplement: Soure data 3. [file elife-32490-fig3.zip › Figure 5/Panel a/Bowes/Bowes_activeITGB1_594_Max_c3.tif]

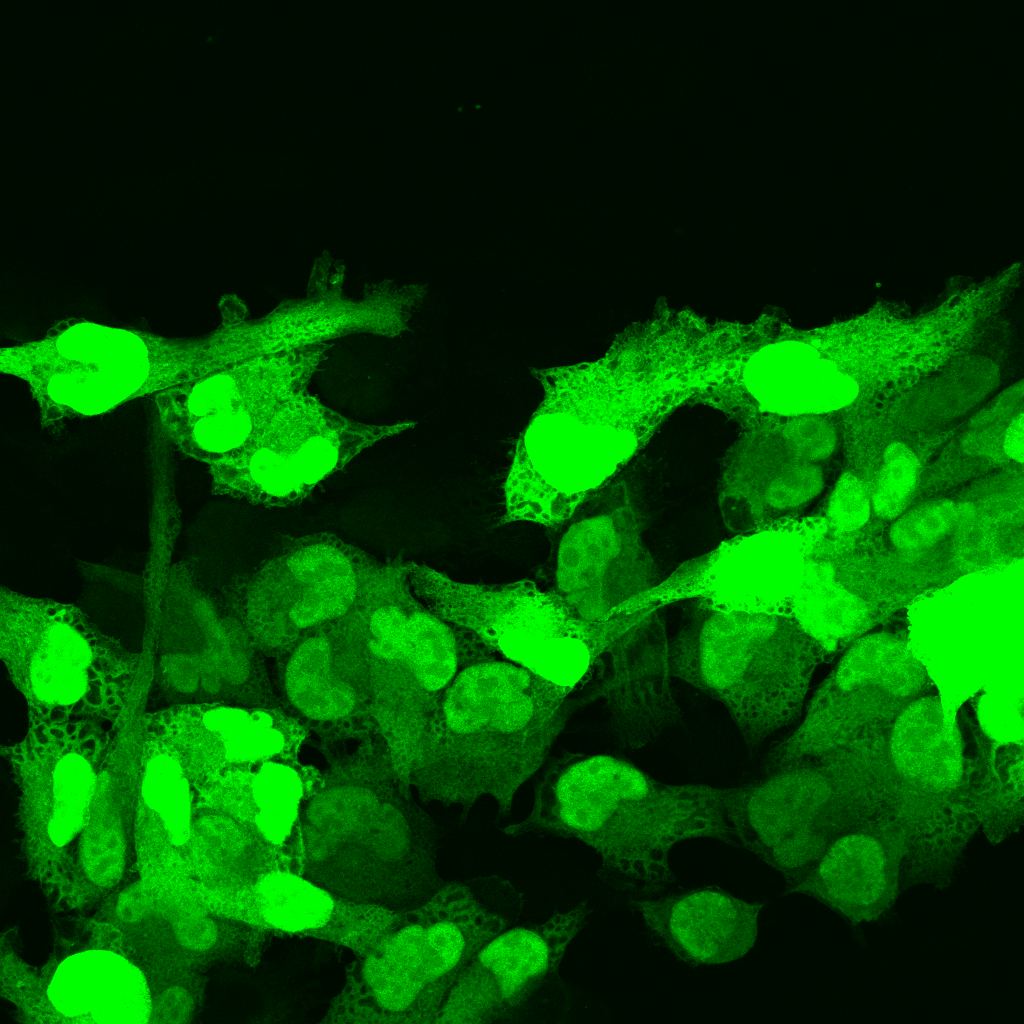

Supplement: Soure data 3. [file elife-32490-fig3.zip › Figure 5/Panel a/Bowes_LEC/LEC_Bowes_activeITGB1_594_Max_c1.tif]

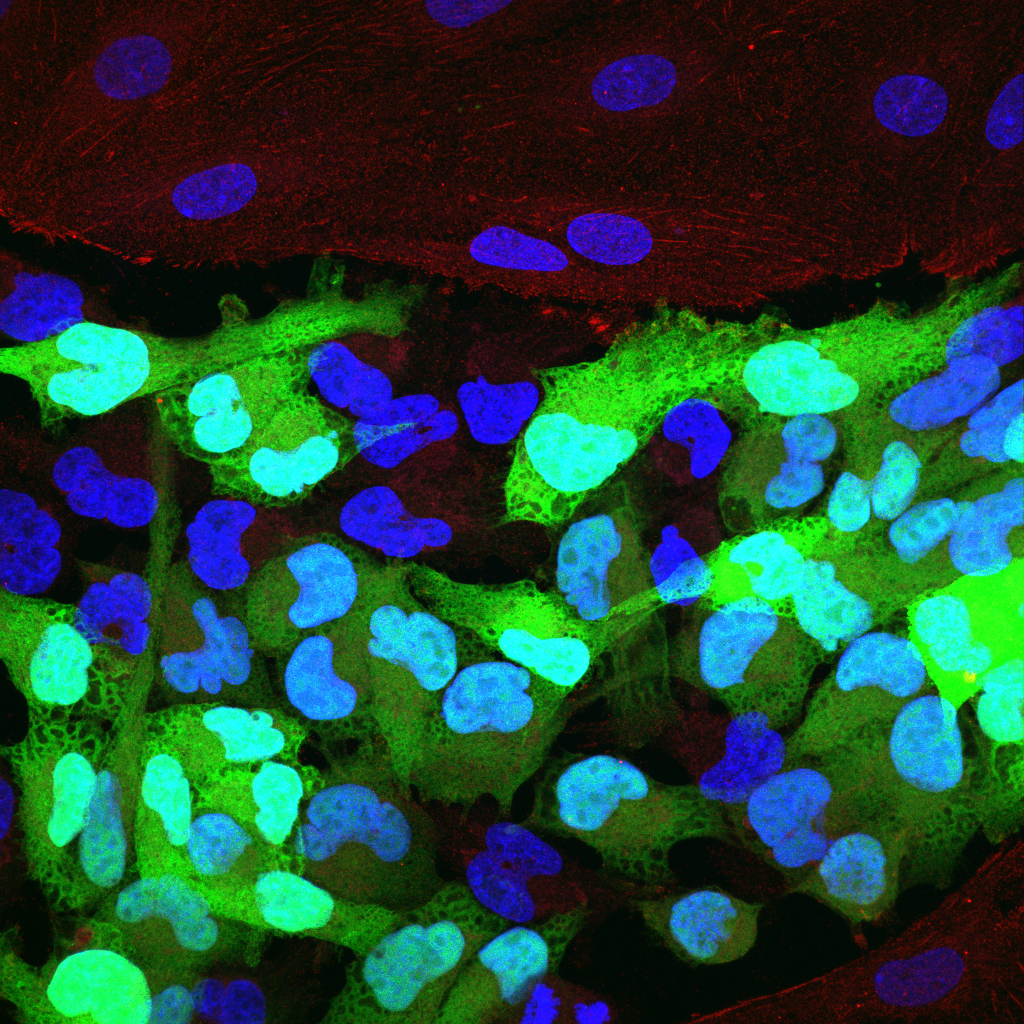

Supplement: Soure data 3. [file elife-32490-fig3.zip › Figure 5/Panel a/Bowes_LEC/LEC_Bowes_activeITGB1_594_Max_c1+2+3.tif]

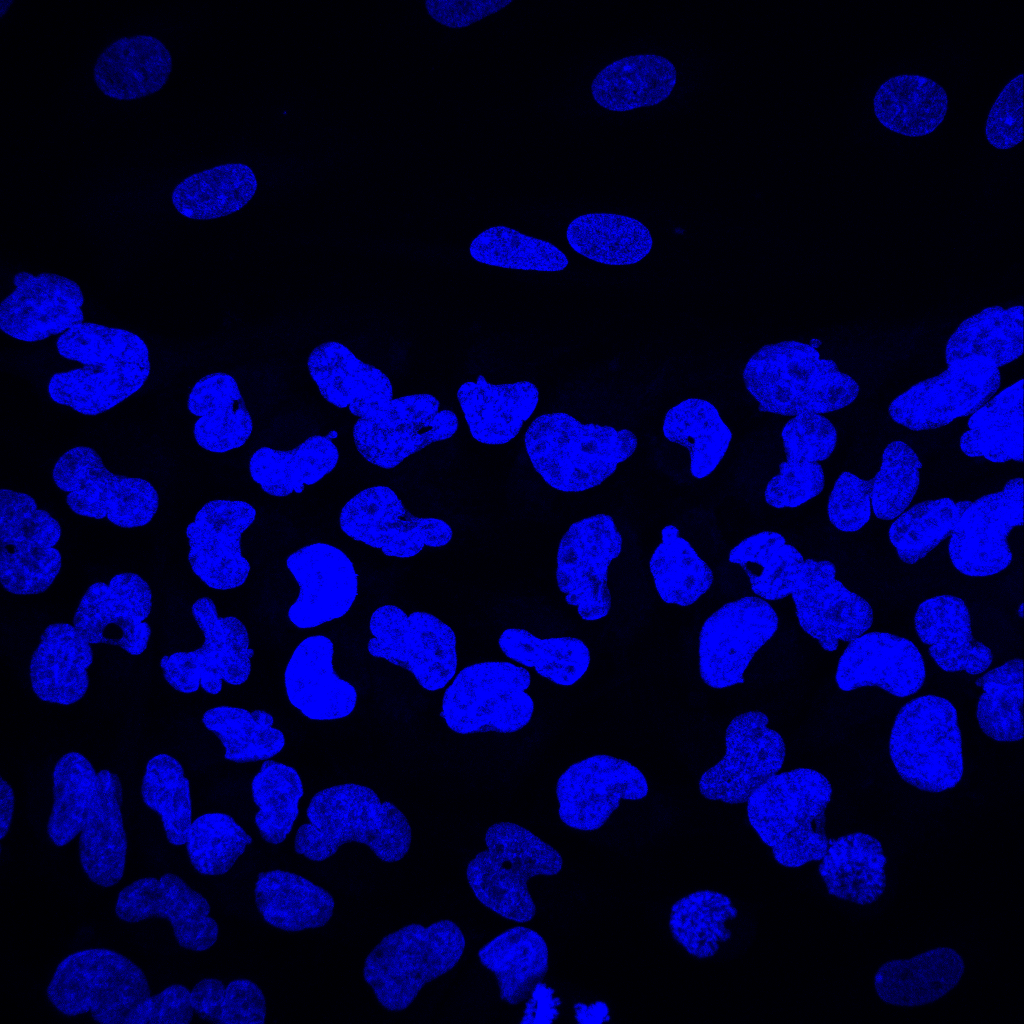

Supplement: Soure data 3. [file elife-32490-fig3.zip › Figure 5/Panel a/Bowes_LEC/LEC_Bowes_activeITGB1_594_Max_c2.tif]

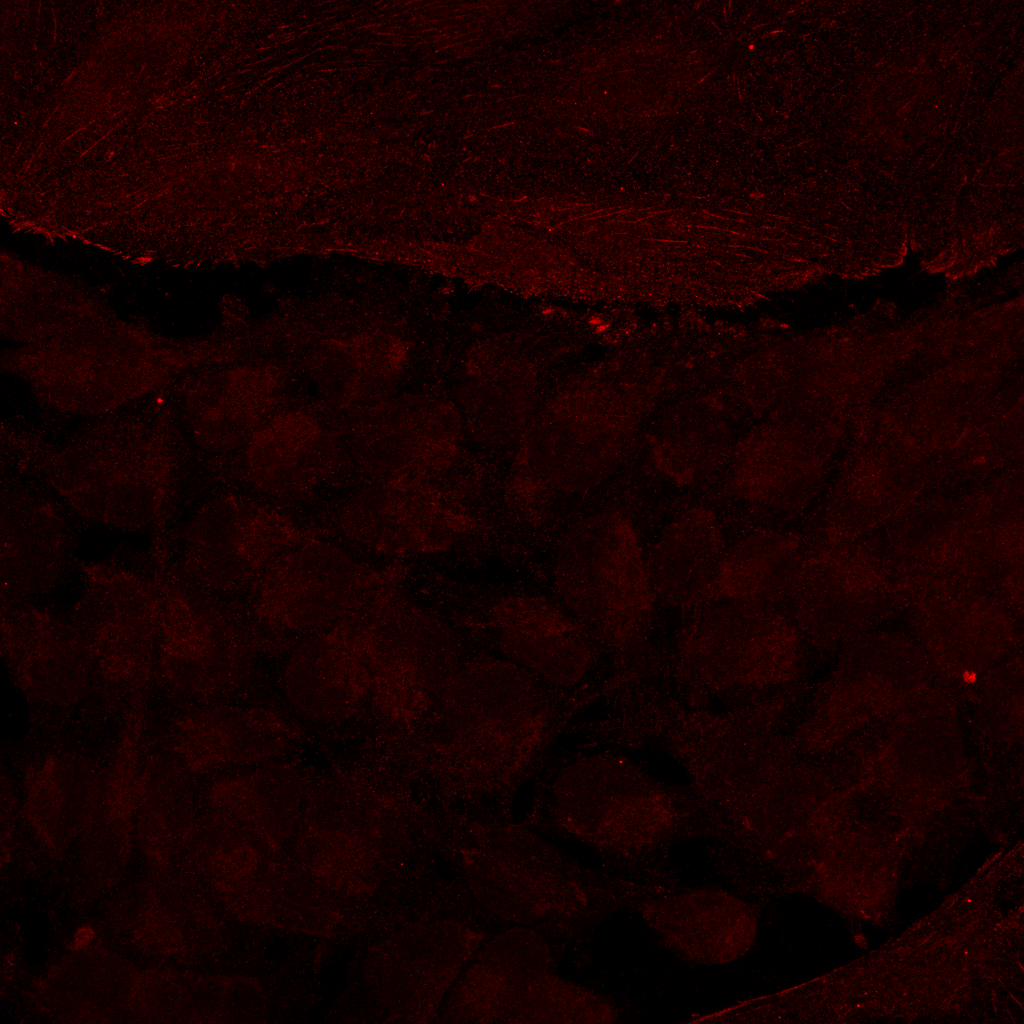

Supplement: Soure data 3. [file elife-32490-fig3.zip › Figure 5/Panel a/Bowes_LEC/LEC_Bowes_activeITGB1_594_Max_c3.tif]

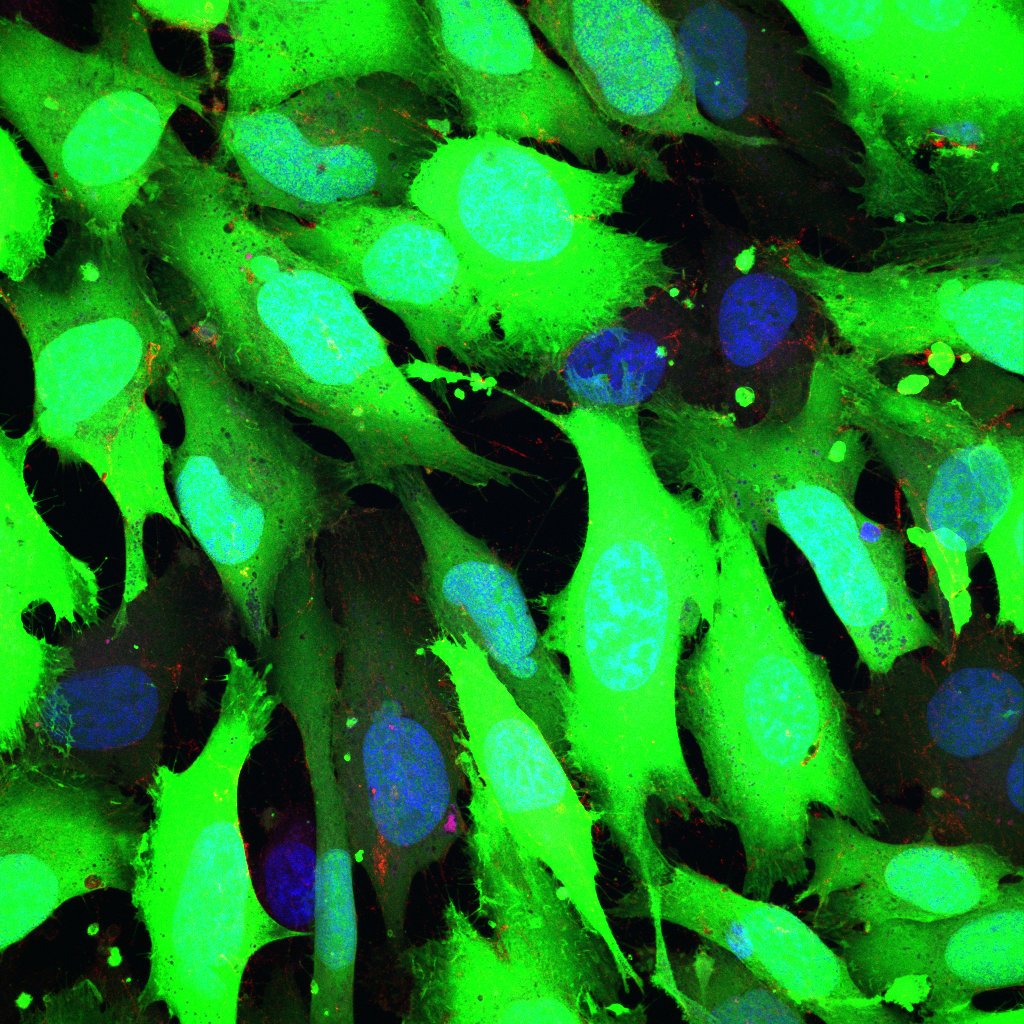

Supplement: Soure data 3. [file elife-32490-fig3.zip › Figure 5/Panel a/WM165/WM165_activeITGB1_594_Max_c1+2+3.tif]

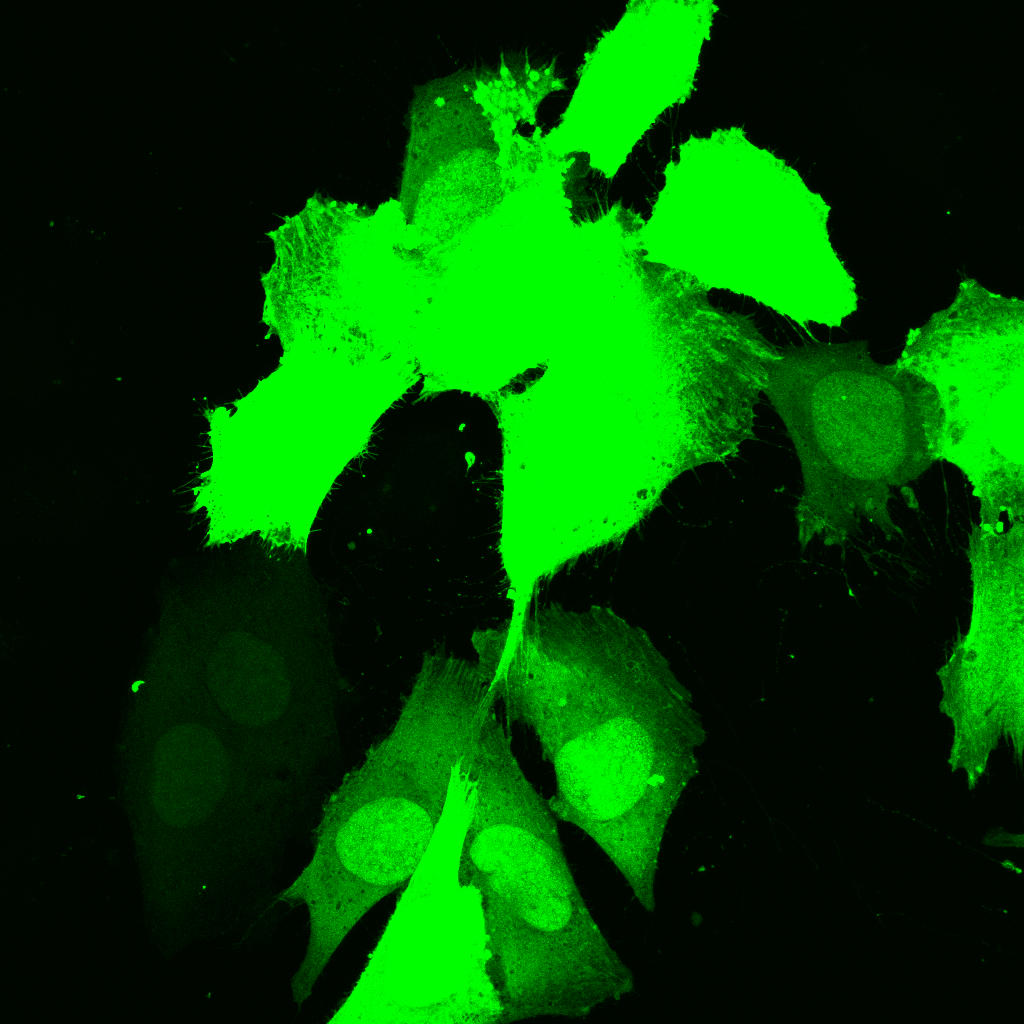

Supplement: Soure data 3. [file elife-32490-fig3.zip › Figure 5/Panel a/WM165_LEC/LEC_WM165_activeITGB1_Maximum intensity projection_c1.tif]

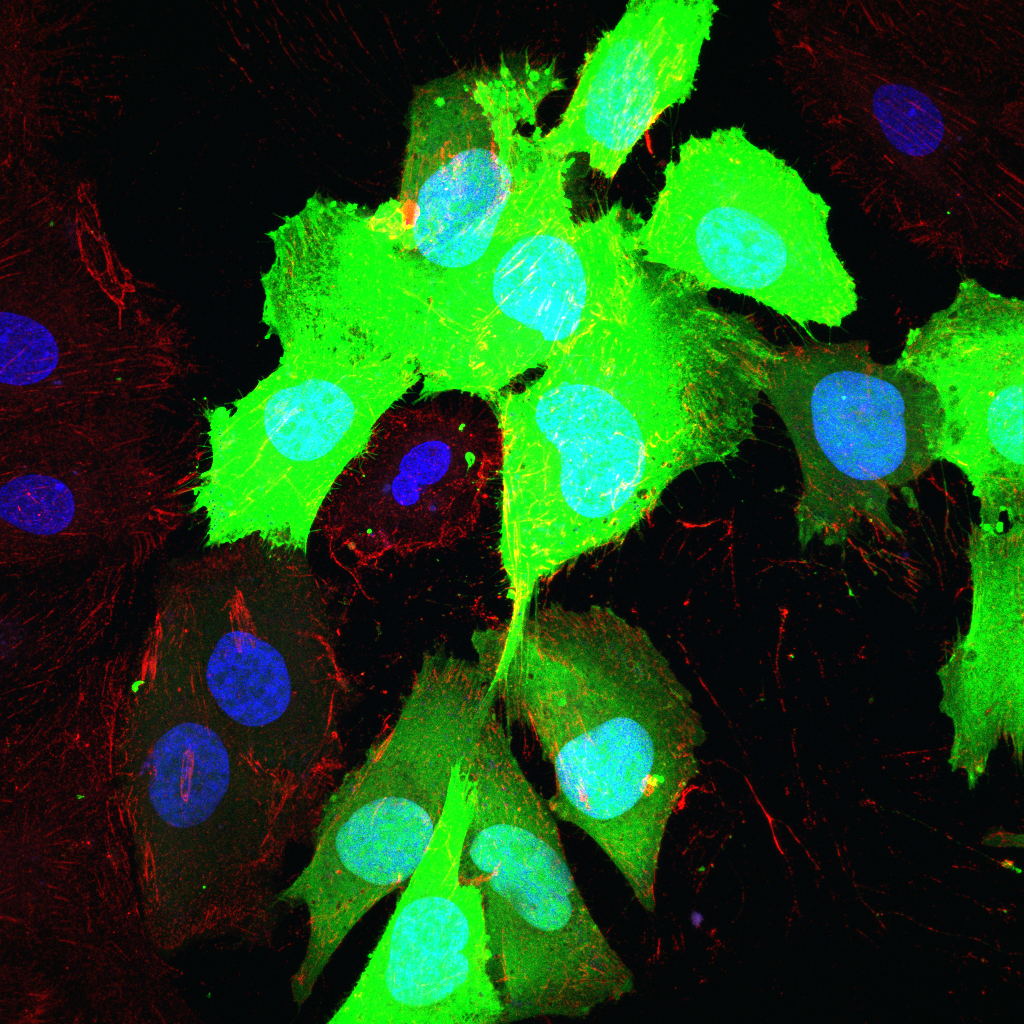

Supplement: Soure data 3. [file elife-32490-fig3.zip › Figure 5/Panel a/WM165_LEC/LEC_WM165_activeITGB1_Maximum intensity projection_c1+2+3.tif]

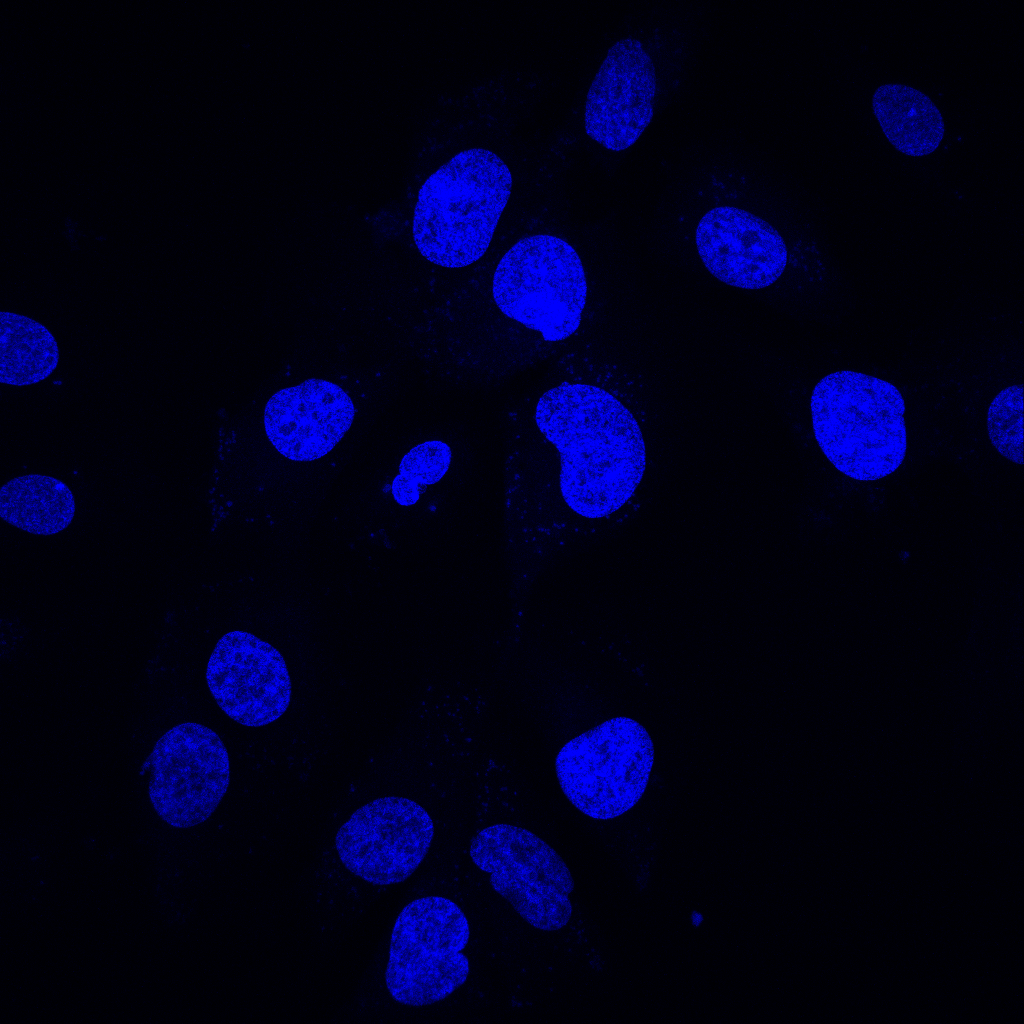

Supplement: Soure data 3. [file elife-32490-fig3.zip › Figure 5/Panel a/WM165_LEC/LEC_WM165_activeITGB1_Maximum intensity projection_c2.tif]

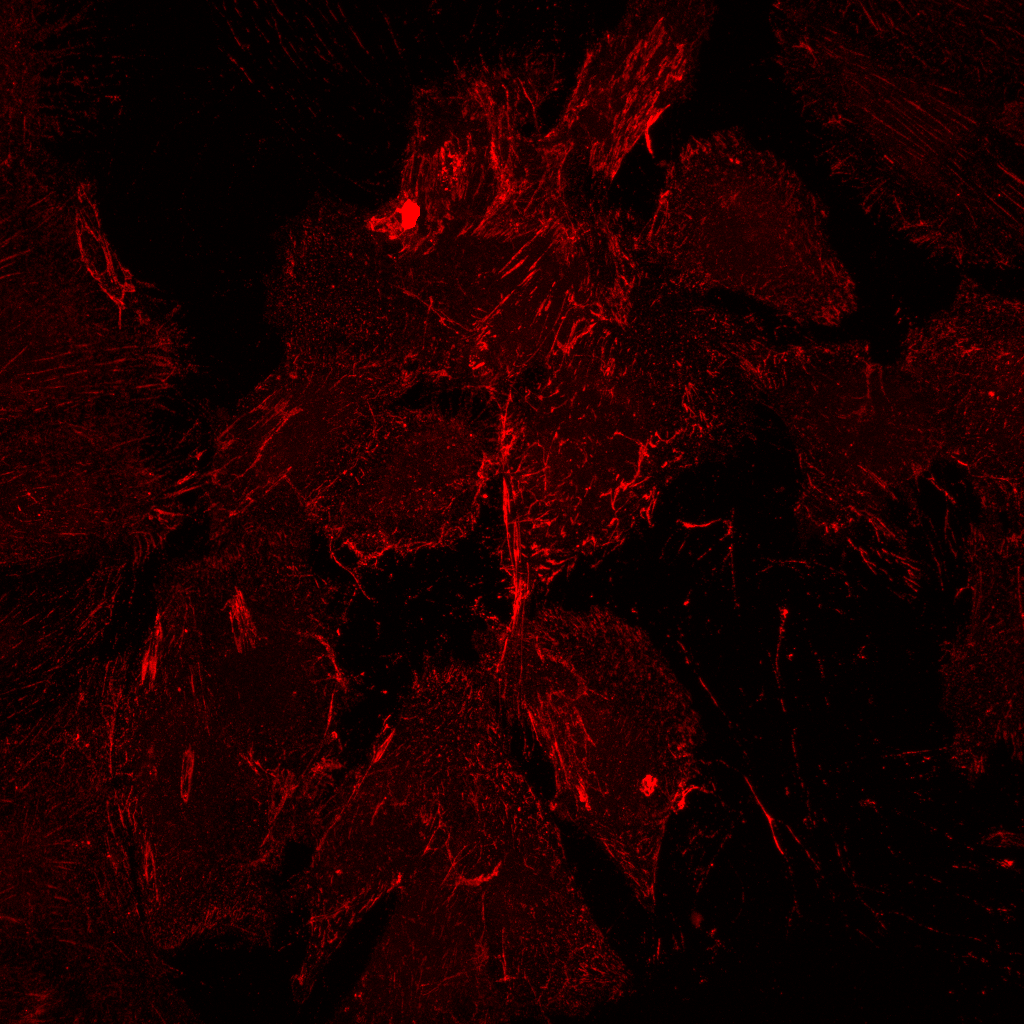

Supplement: Soure data 3. [file elife-32490-fig3.zip › Figure 5/Panel a/WM165_LEC/LEC_WM165_activeITGB1_Maximum intensity projection_c3.tif]

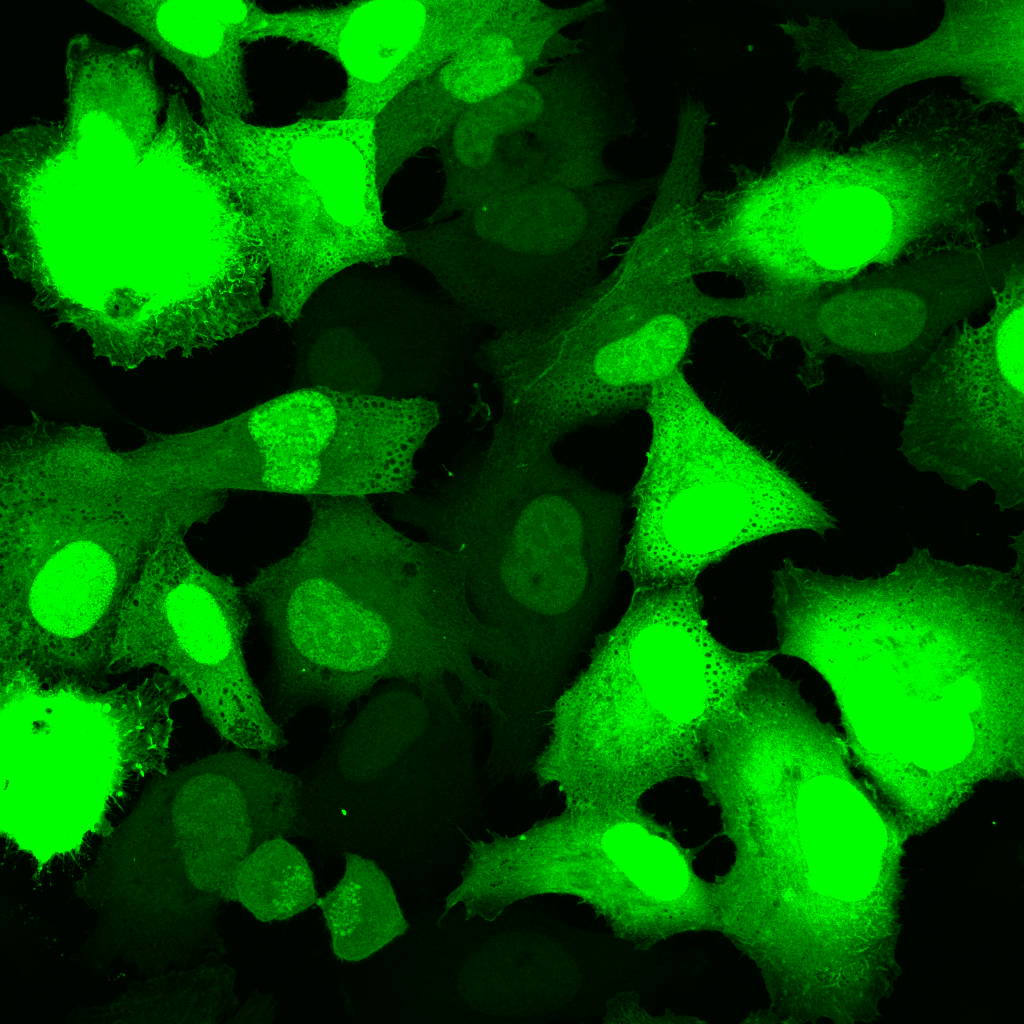

Supplement: Soure data 3. [file elife-32490-fig3.zip › Figure 5/Panel a/WM793/WM793_activeITGB1_594_Max_c1.tif]

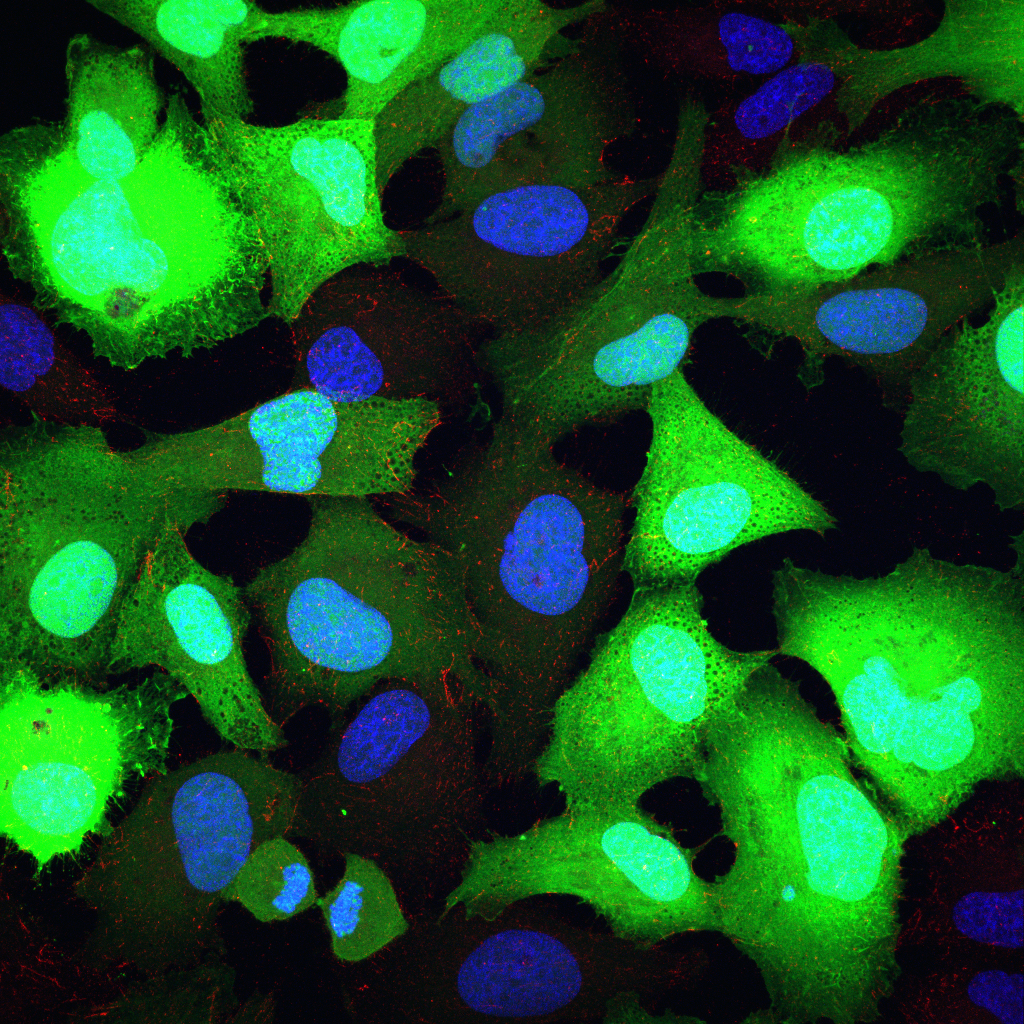

Supplement: Soure data 3. [file elife-32490-fig3.zip › Figure 5/Panel a/WM793/WM793_activeITGB1_594_Max_c1+2+3.tif]

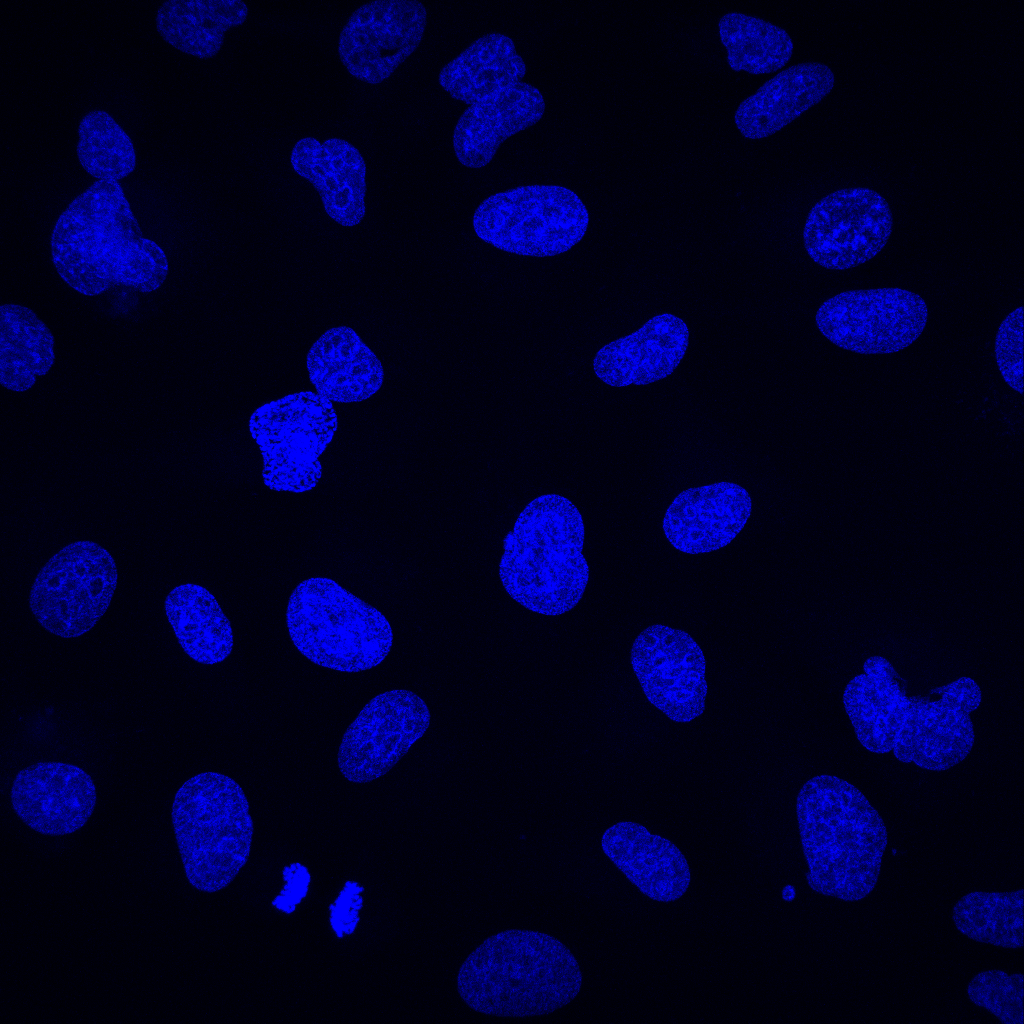

Supplement: Soure data 3. [file elife-32490-fig3.zip › Figure 5/Panel a/WM793/WM793_activeITGB1_594_Max_c2.tif]

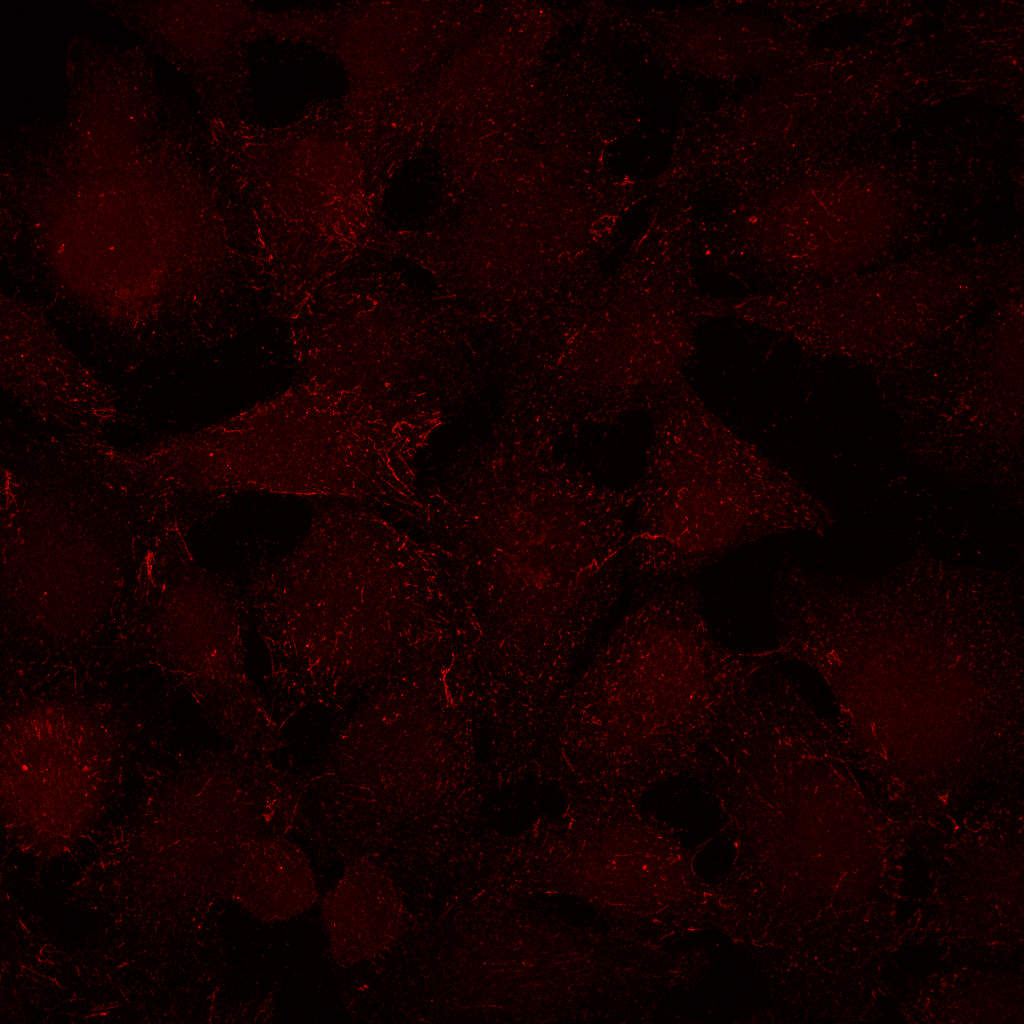

Supplement: Soure data 3. [file elife-32490-fig3.zip › Figure 5/Panel a/WM793/WM793_activeITGB1_594_Max_c3.tif]

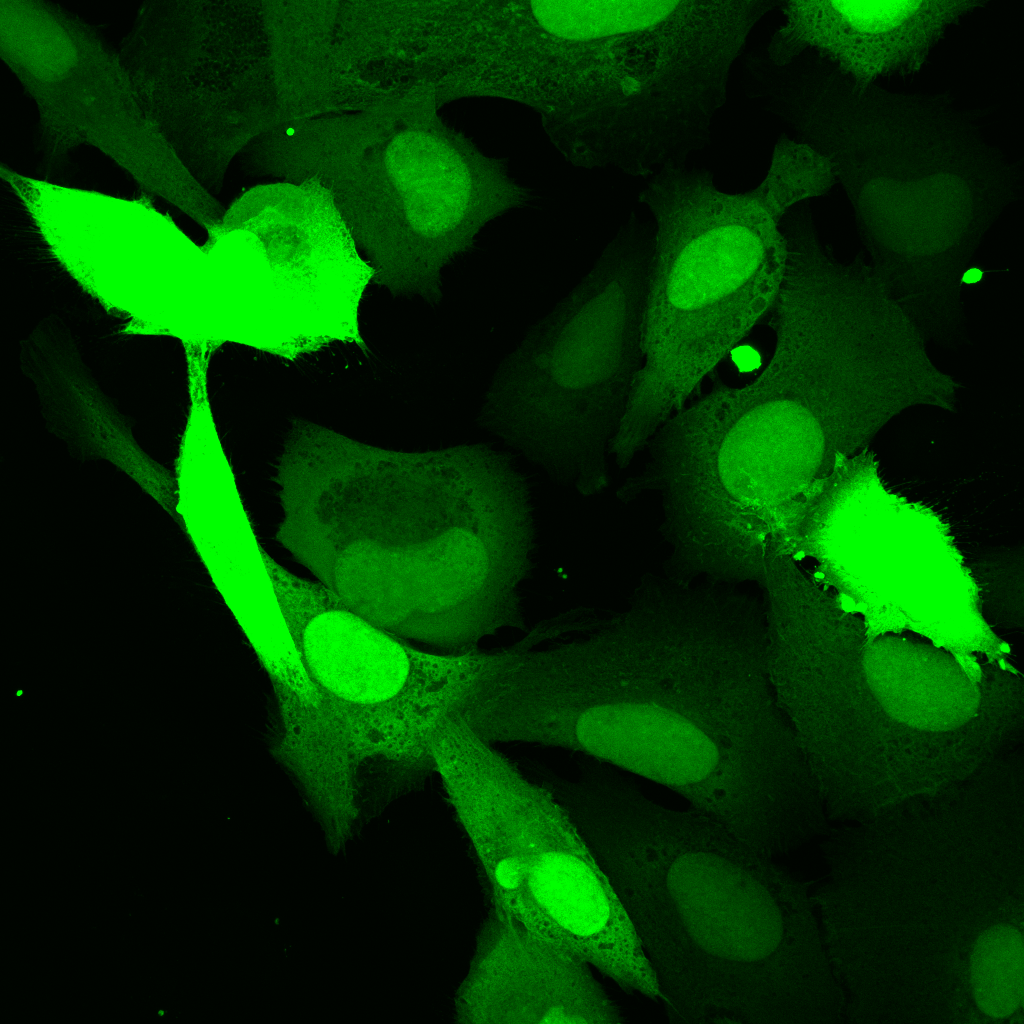

Supplement: Soure data 3. [file elife-32490-fig3.zip › Figure 5/Panel a/WM793_LEC/LEC_WM793_activeITGB1_594_Max_c1.tif]

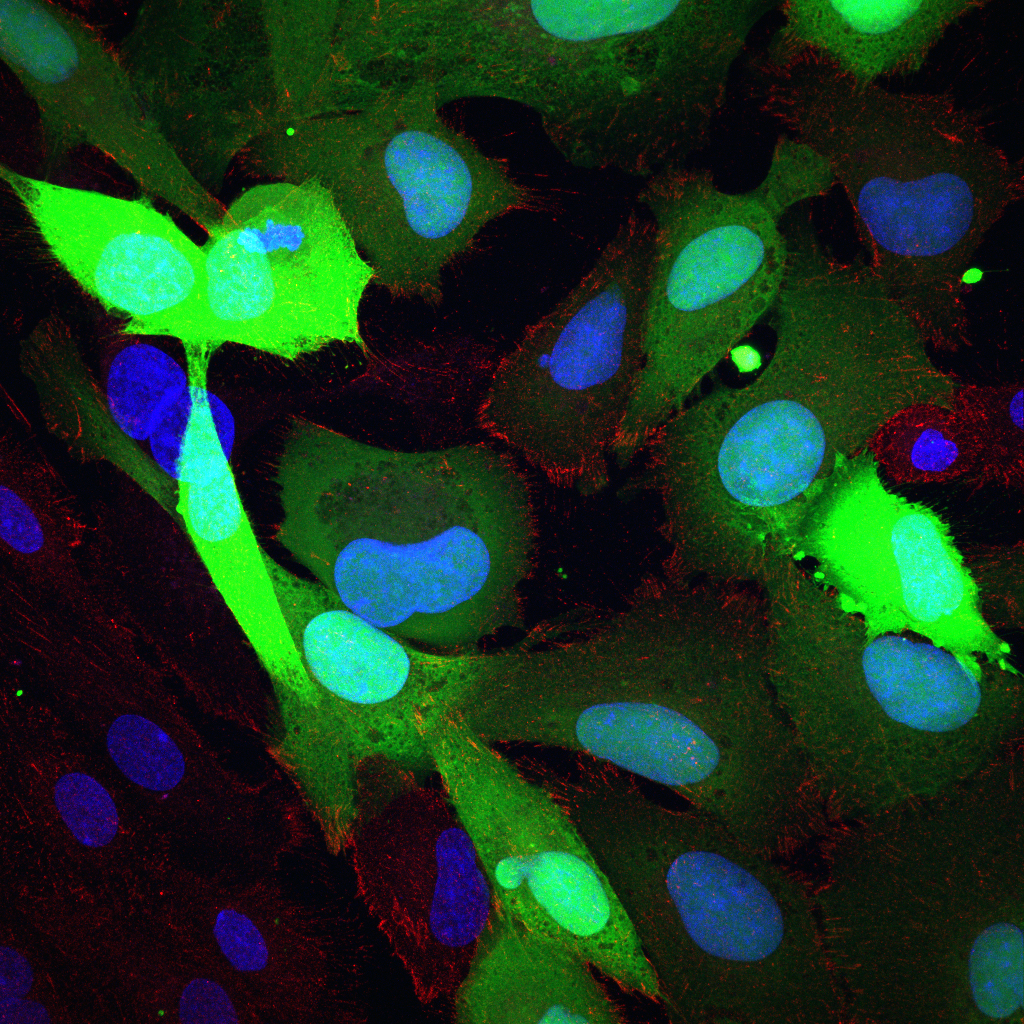

Supplement: Soure data 3. [file elife-32490-fig3.zip › Figure 5/Panel a/WM793_LEC/LEC_WM793_activeITGB1_594_Max_c1+2+3.tif]
