# Supplementary figures and images for: Lymphatic endothelium stimulates melanoma metastasis and invasion via MMP14-dependent Notch3 and β1-integrin activation (part 2 of 2)
Source: eLife. 2018 May 1;7:e32490. doi: 10.7554/eLife.32490 (PMC5929907; doi:10.7554/eLife.32490)

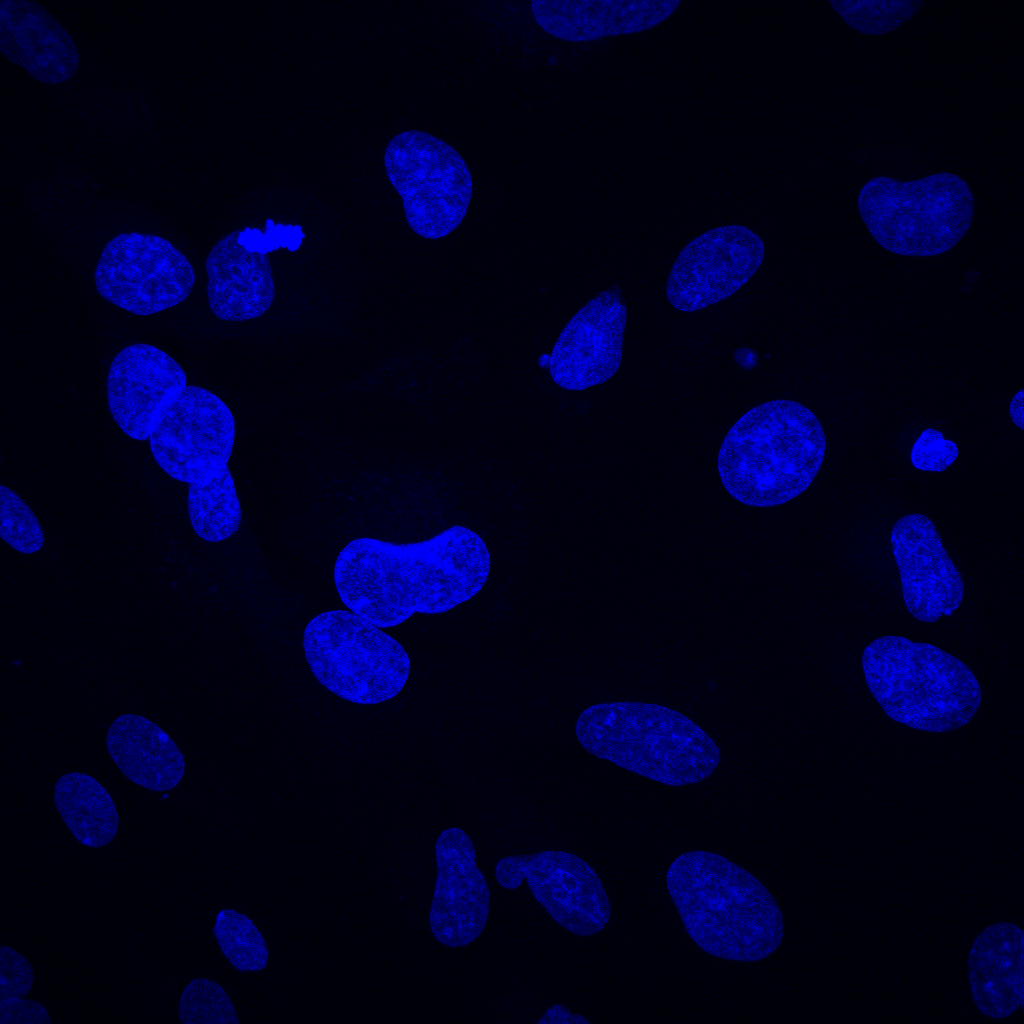

Supplement: Soure data 3. [file elife-32490-fig3.zip › Figure 5/Panel a/WM793_LEC/LEC_WM793_activeITGB1_594_Max_c2.tif]

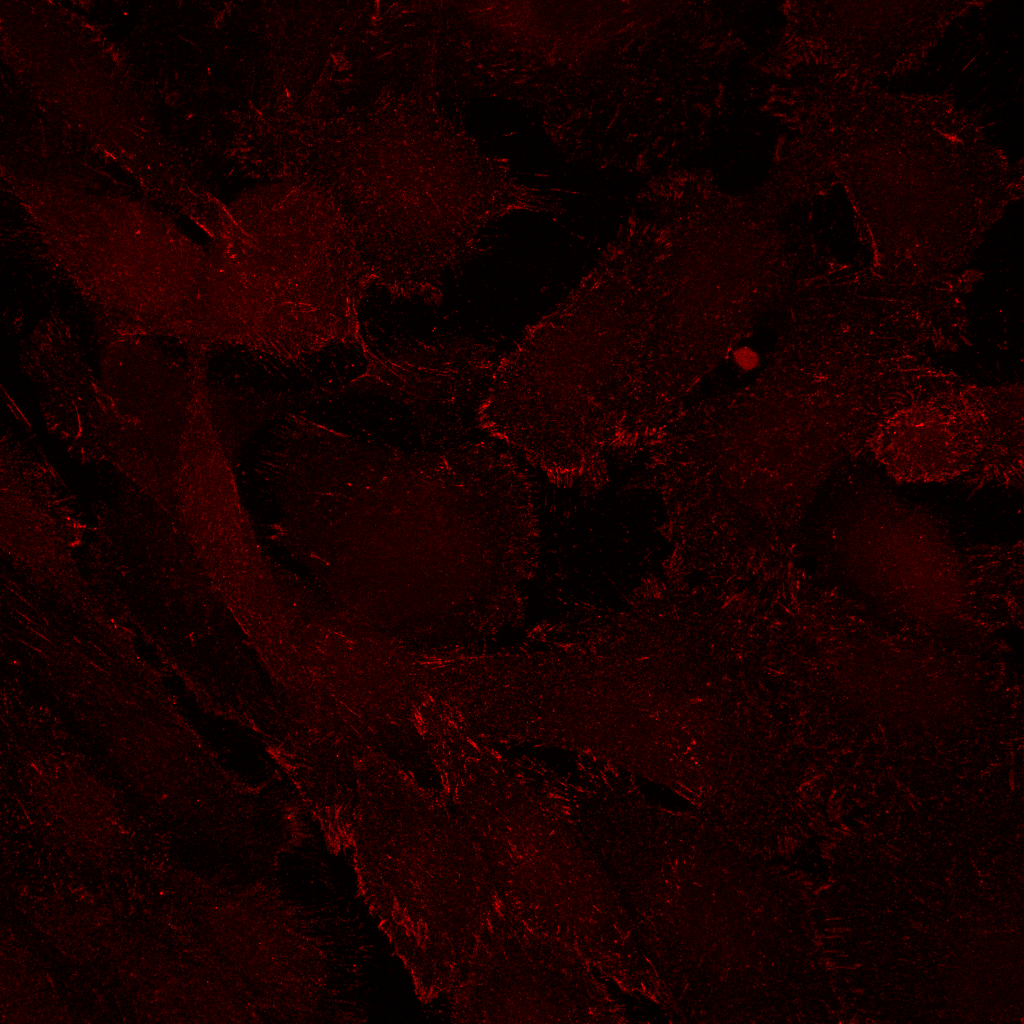

Supplement: Soure data 3. [file elife-32490-fig3.zip › Figure 5/Panel a/WM793_LEC/LEC_WM793_activeITGB1_594_Max_c3.tif]

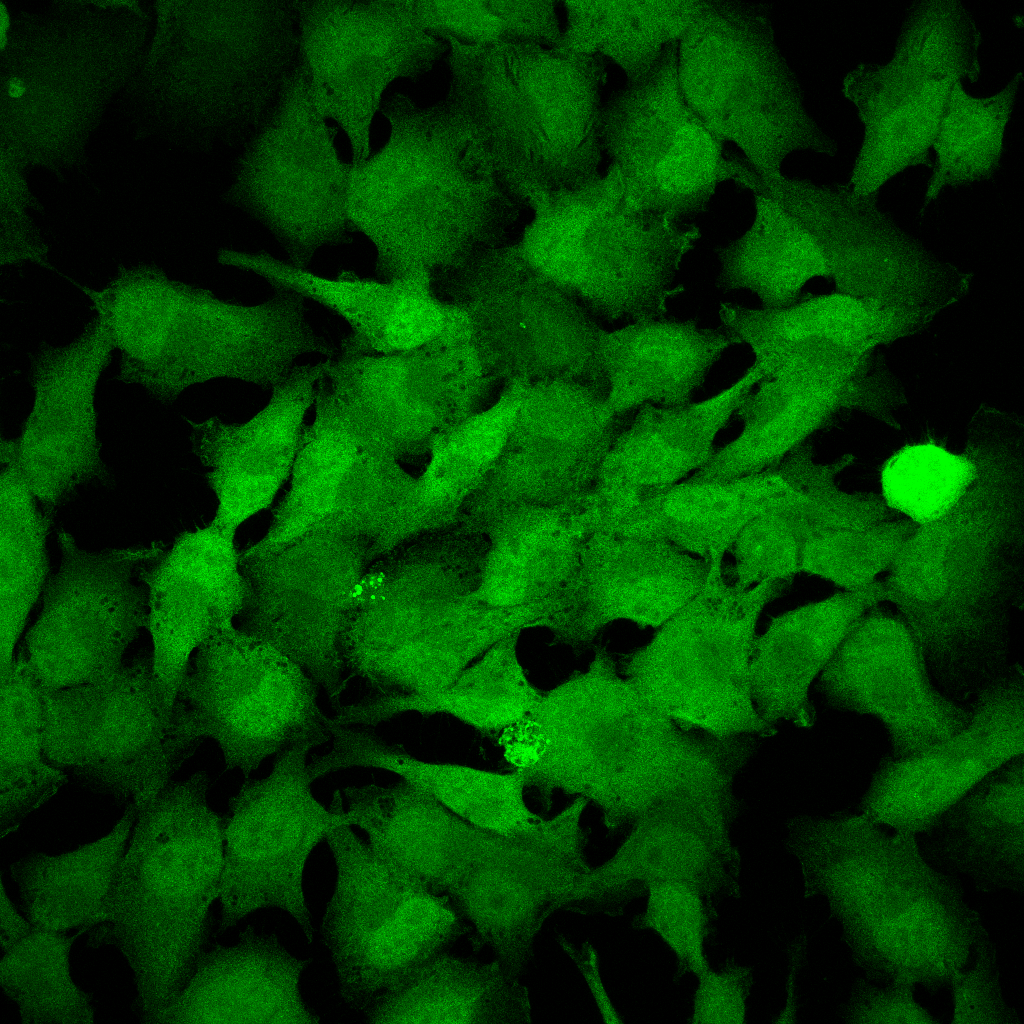

Supplement: Soure data 3. [file elife-32490-fig3.zip › Figure 5/Panel a/WM852/WM852_activeITGB1_2_594_Max_c1.tif]

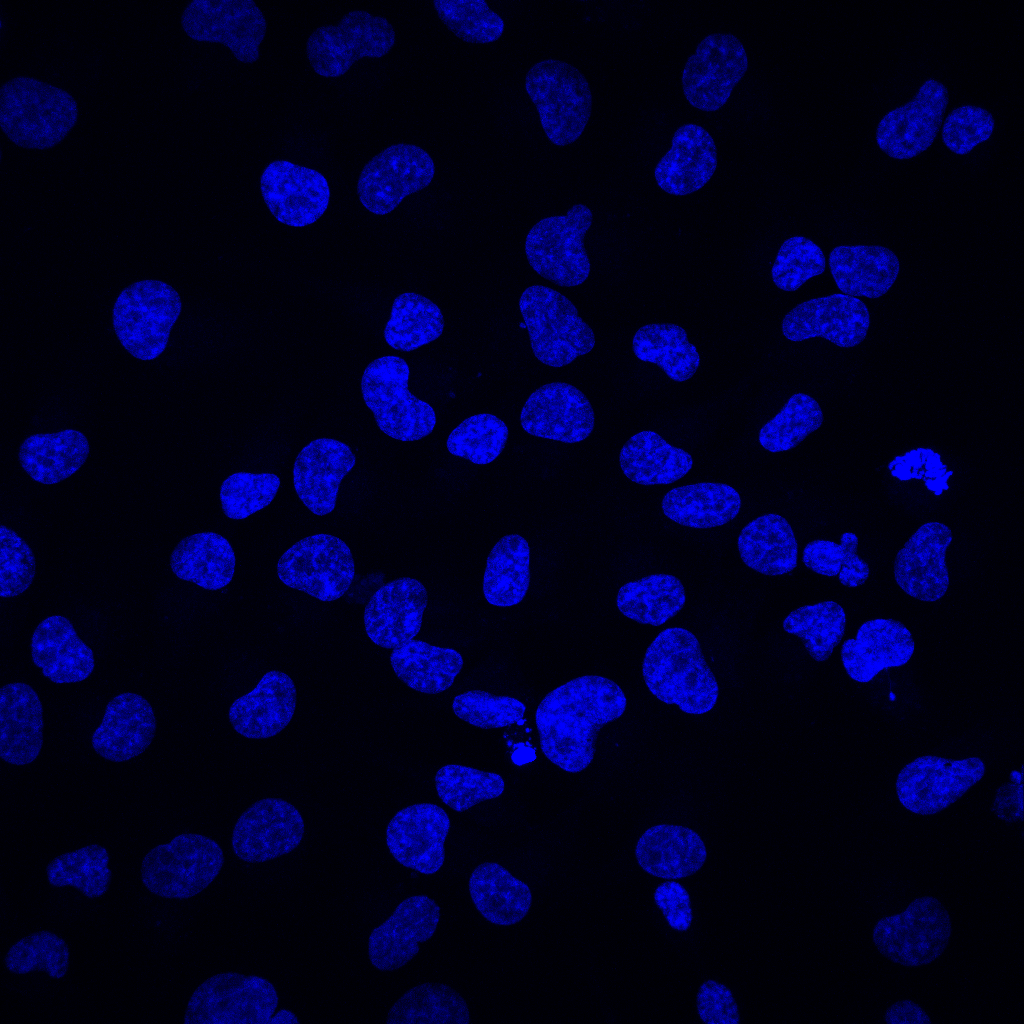

Supplement: Soure data 3. [file elife-32490-fig3.zip › Figure 5/Panel a/WM852/WM852_activeITGB1_594_2_Max_c2.tif]

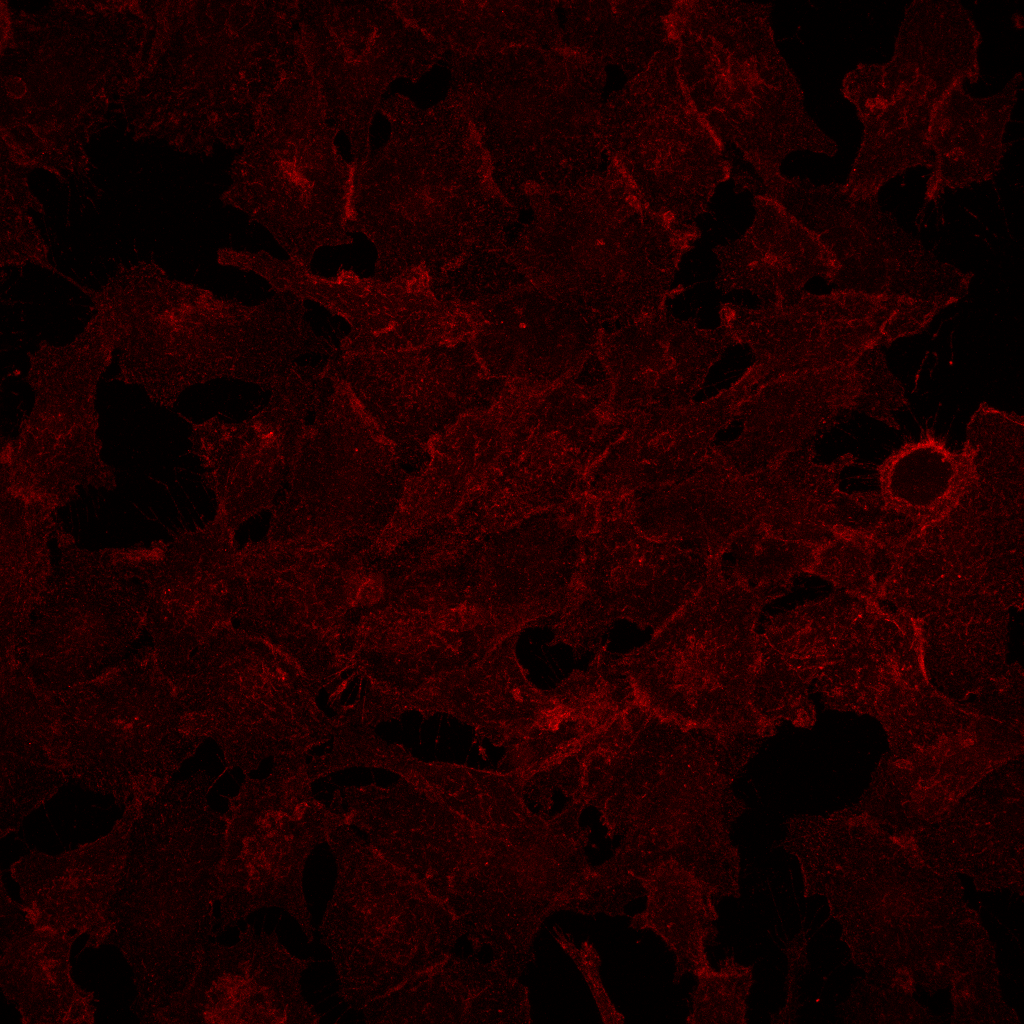

Supplement: Soure data 3. [file elife-32490-fig3.zip › Figure 5/Panel a/WM852/WM852_activeITGB1_594_2_Max_c3.tif]

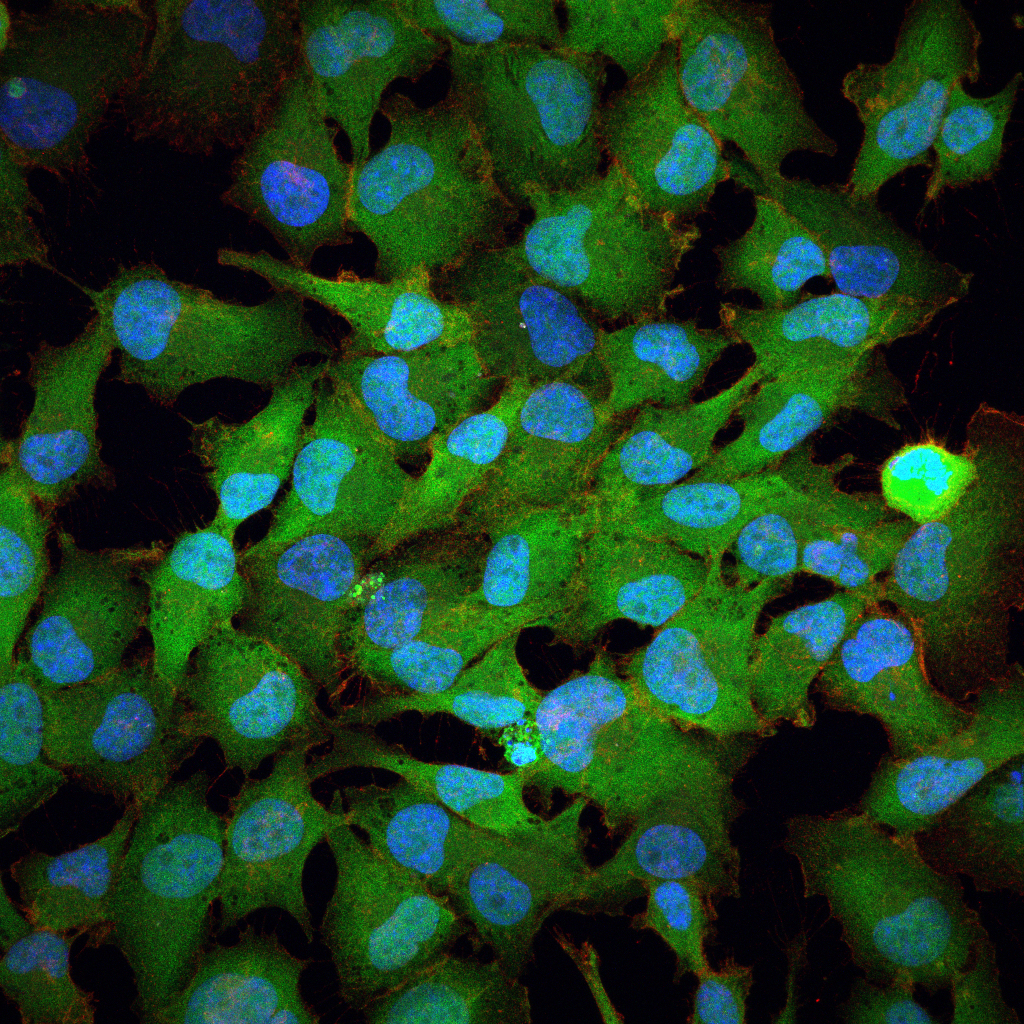

Supplement: Soure data 3. [file elife-32490-fig3.zip › Figure 5/Panel a/WM852/WM852_activeITGB1_594_Max_c1+2+3.tif]

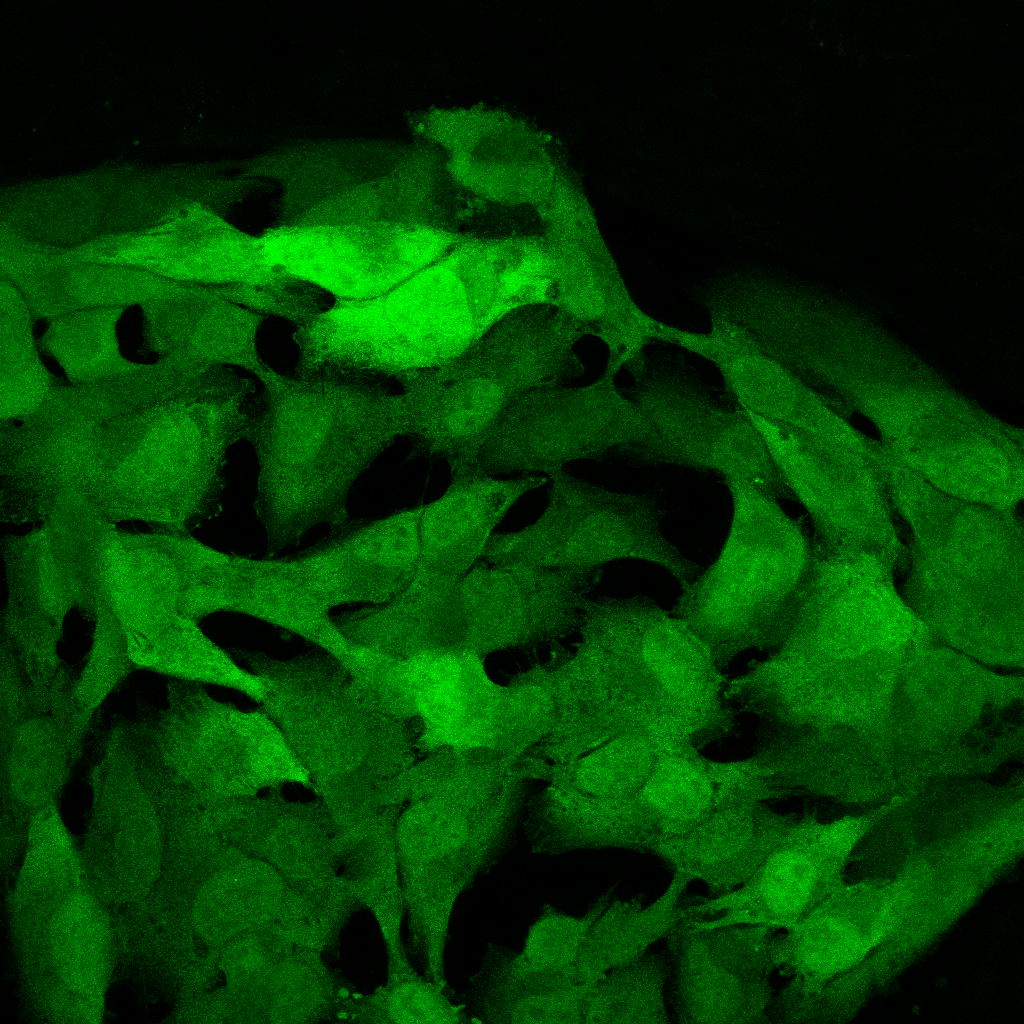

Supplement: Soure data 3. [file elife-32490-fig3.zip › Figure 5/Panel a/WM852_LEC/LEC_WM852_activeITGB1_594_Max_c1.tif]

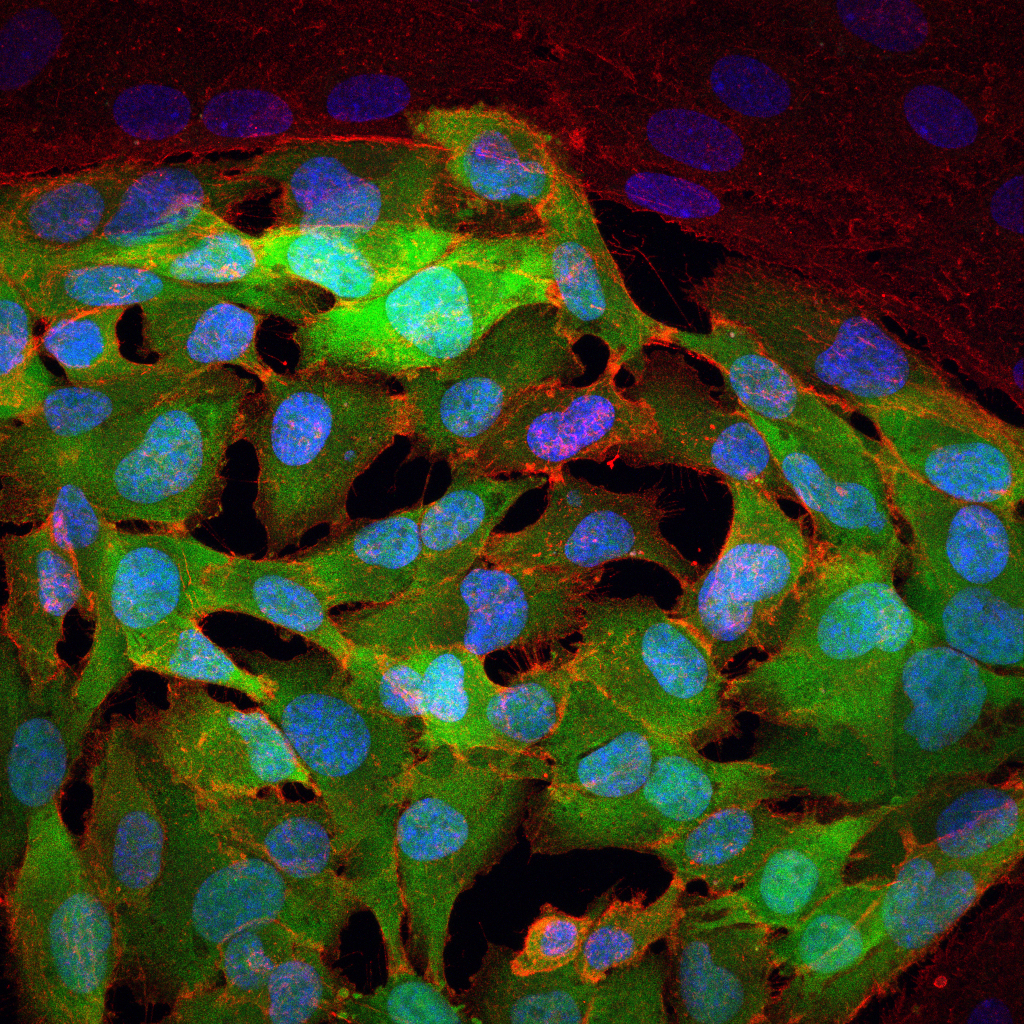

Supplement: Soure data 3. [file elife-32490-fig3.zip › Figure 5/Panel a/WM852_LEC/LEC_WM852_activeITGB1_594_Max_c1+2+3.tif]

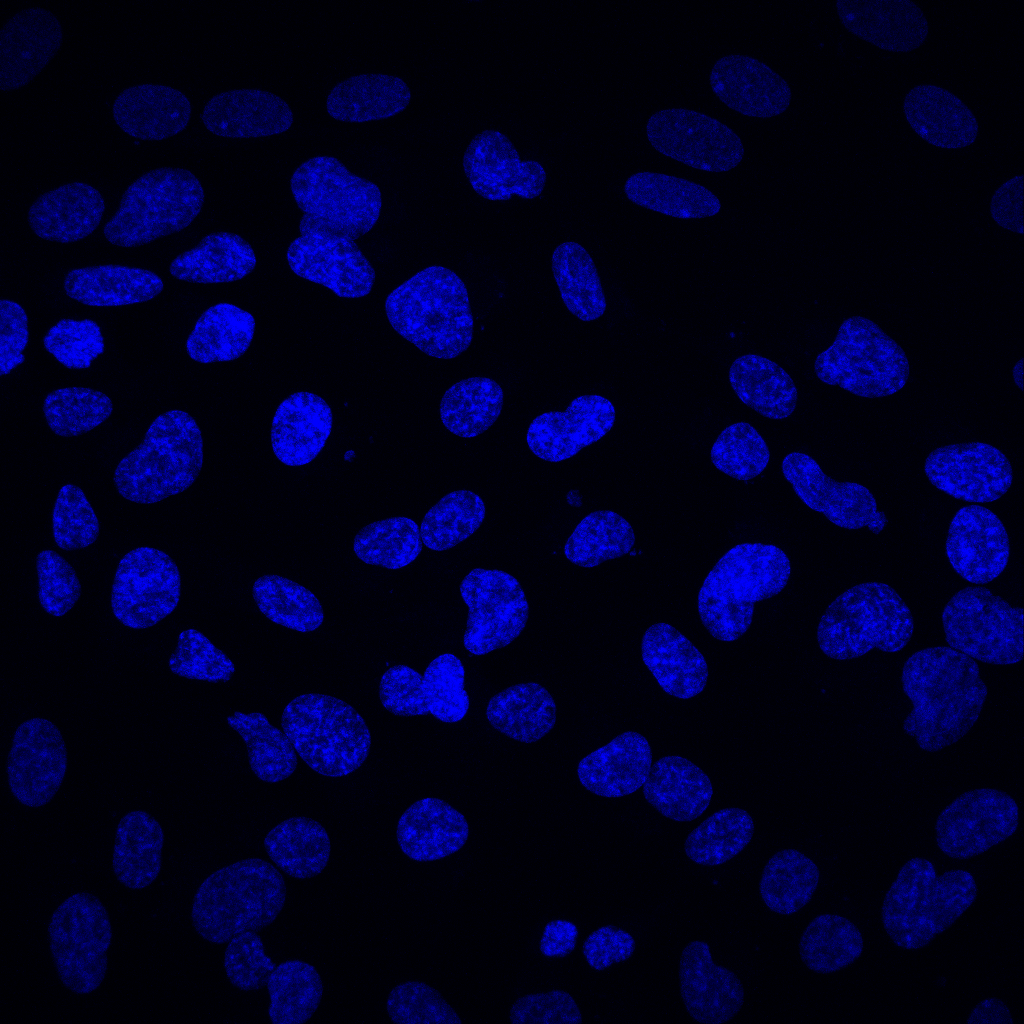

Supplement: Soure data 3. [file elife-32490-fig3.zip › Figure 5/Panel a/WM852_LEC/LEC_WM852_activeITGB1_594_Max_c2.tif]

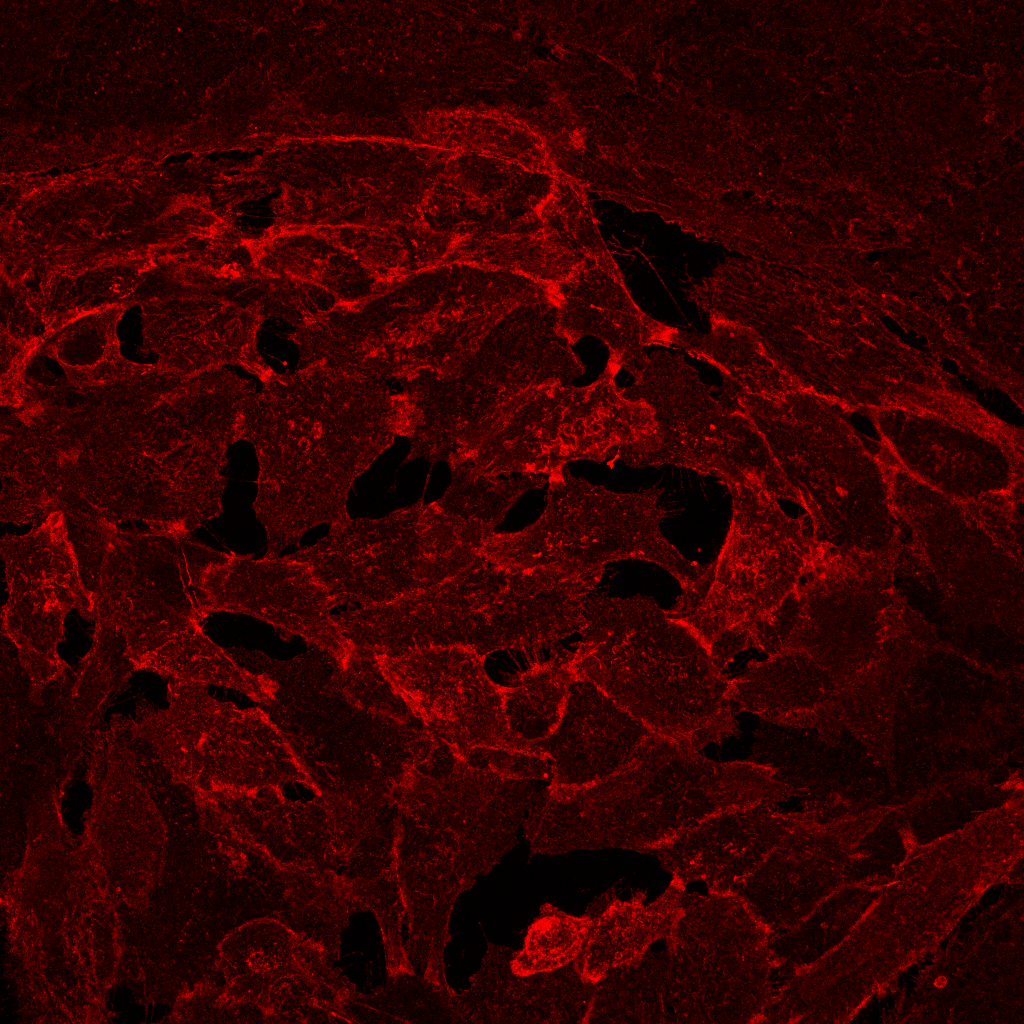

Supplement: Soure data 3. [file elife-32490-fig3.zip › Figure 5/Panel a/WM852_LEC/LEC_WM852_activeITGB1_594_Max_c3.tif]

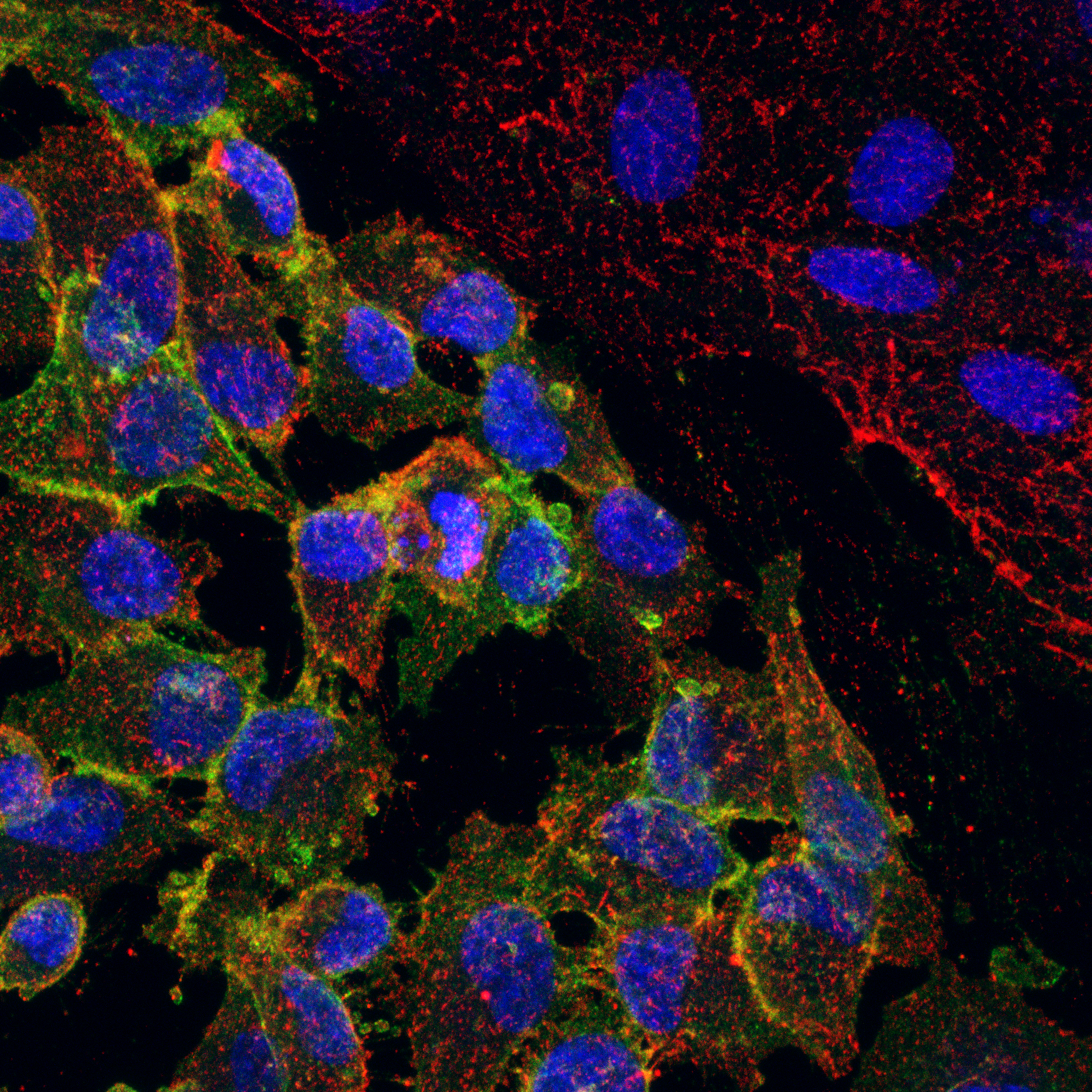

Supplement: Soure data 3. [file elife-32490-fig3.zip › Figure 5/Panel c/Image 49_Maximum intensity projection.tif]

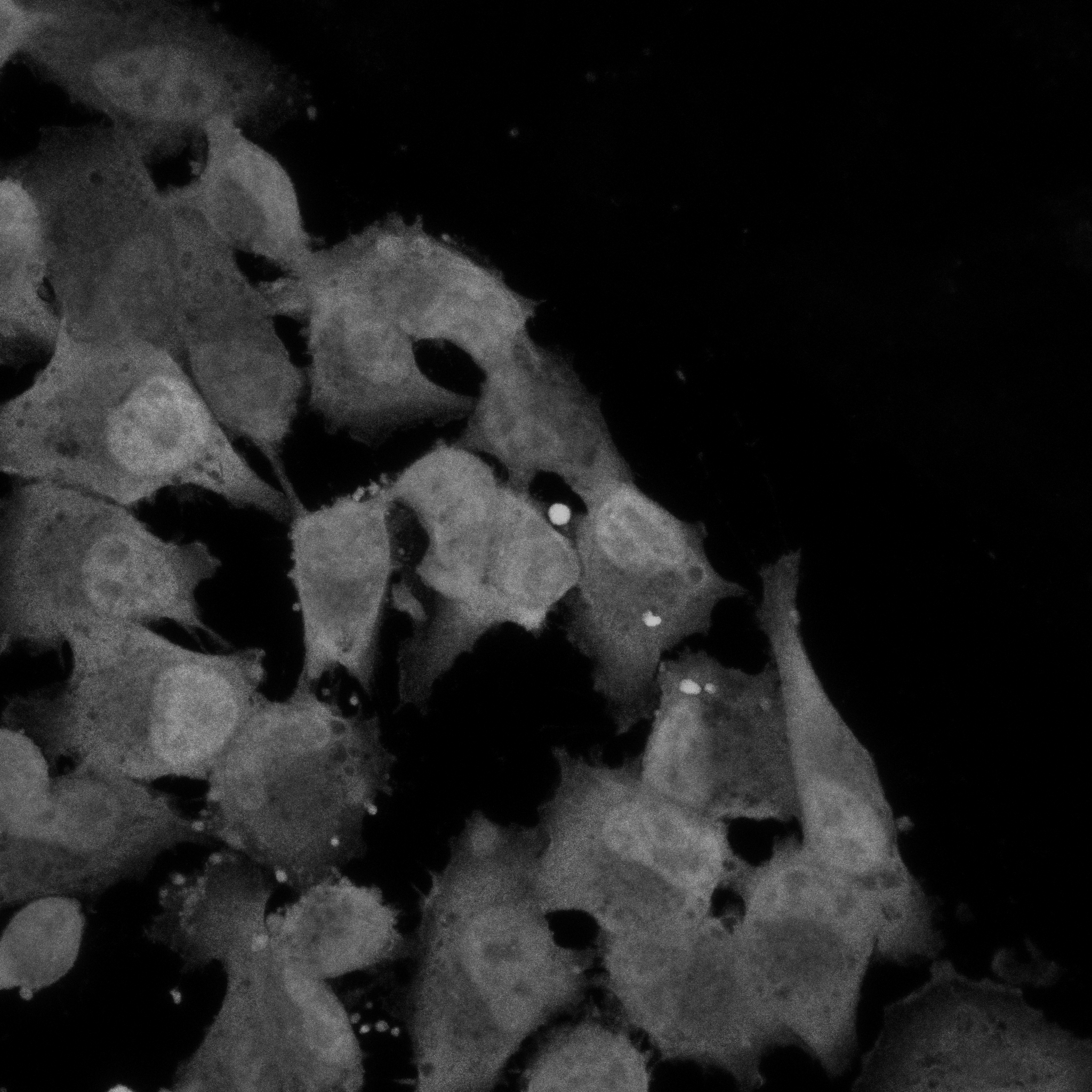

Supplement: Soure data 3. [file elife-32490-fig3.zip › Figure 5/Panel c/Image 49_Maximum intensity projection_c1.tif]

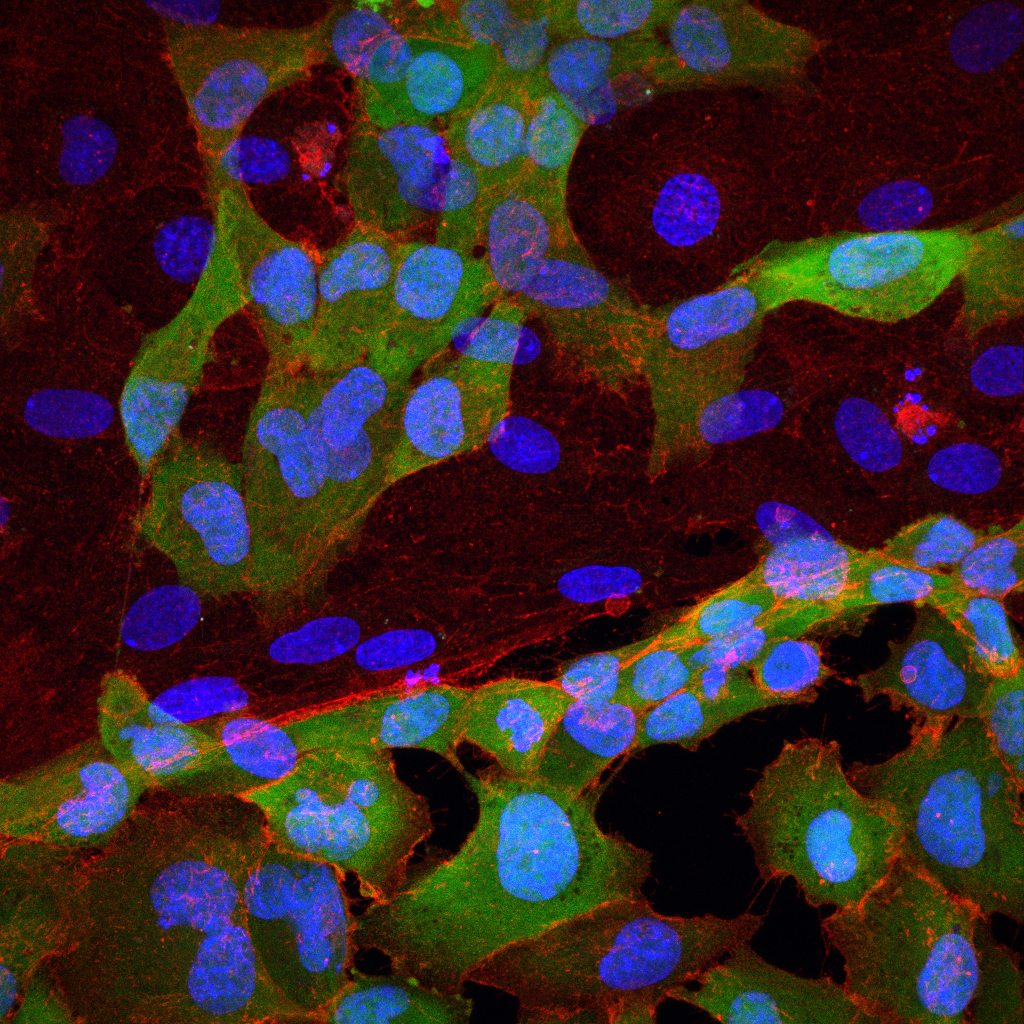

Supplement: Soure data 3. [file elife-32490-fig3.zip › Figure 5/Panel d/siCtrl/Image 22_Maximum intensity projection_c1+2+3.tif]

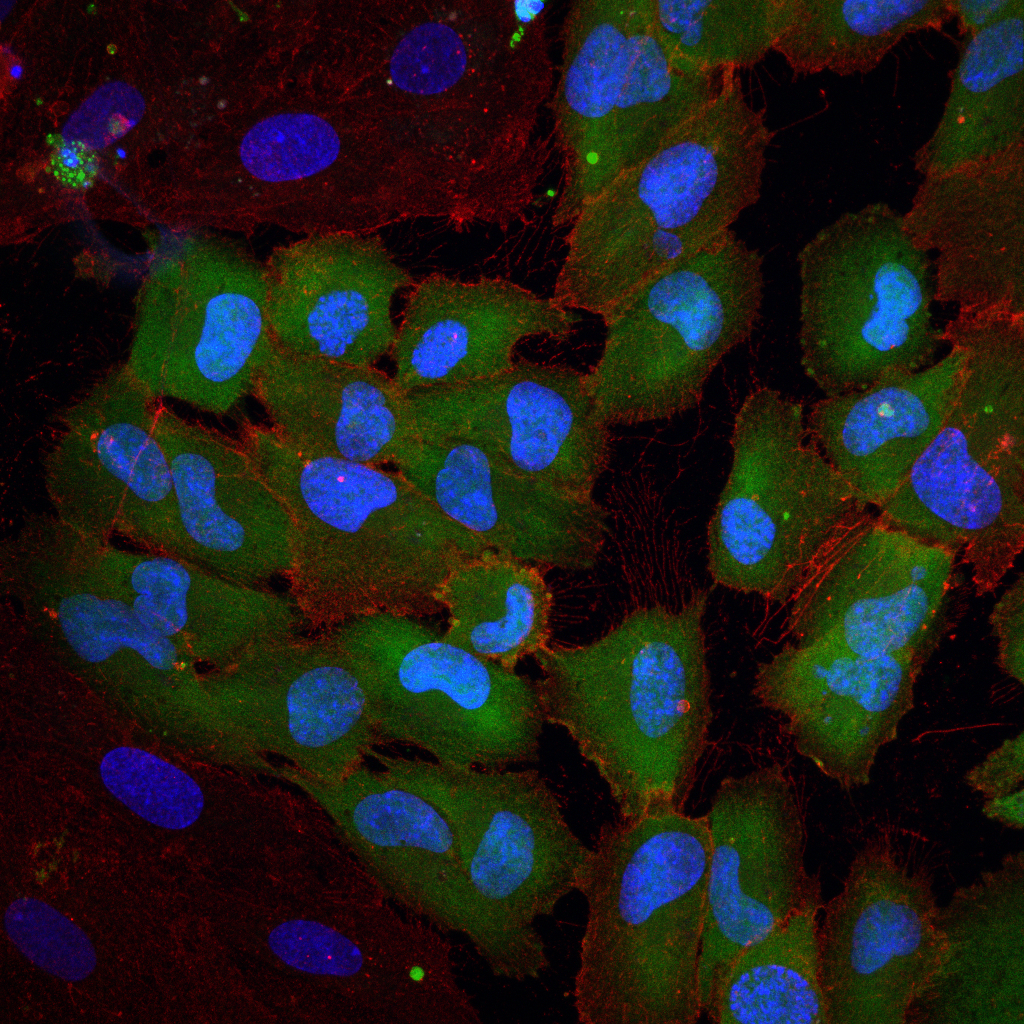

Supplement: Soure data 3. [file elife-32490-fig3.zip › Figure 5/Panel d/siMMP14/Image 21_Maximum intensity projection_c1+2+3.tif]

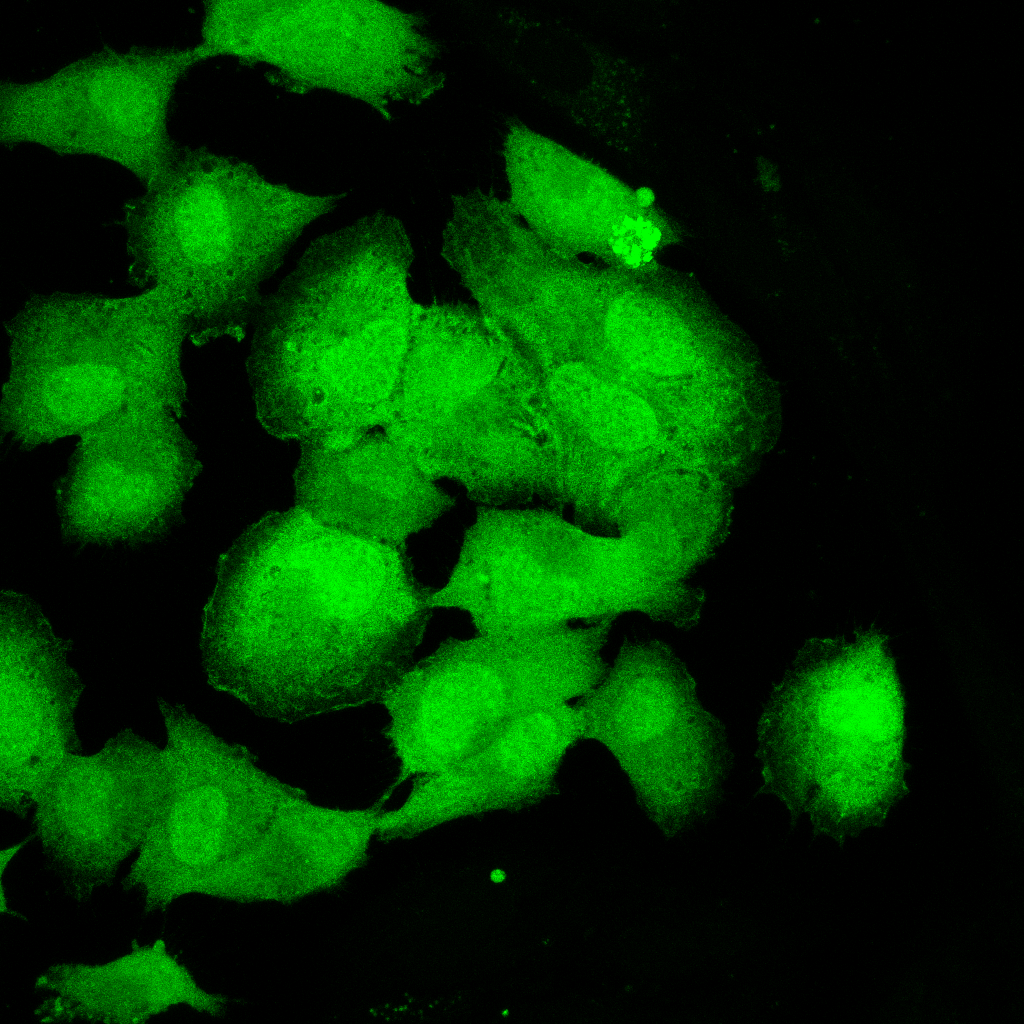

Supplement: Soure data 3. [file elife-32490-fig3.zip › Figure 5/Panel f/siCtrl/LEC_siSCR_totalITGB1_Maximum intensity projection_c1.tif]

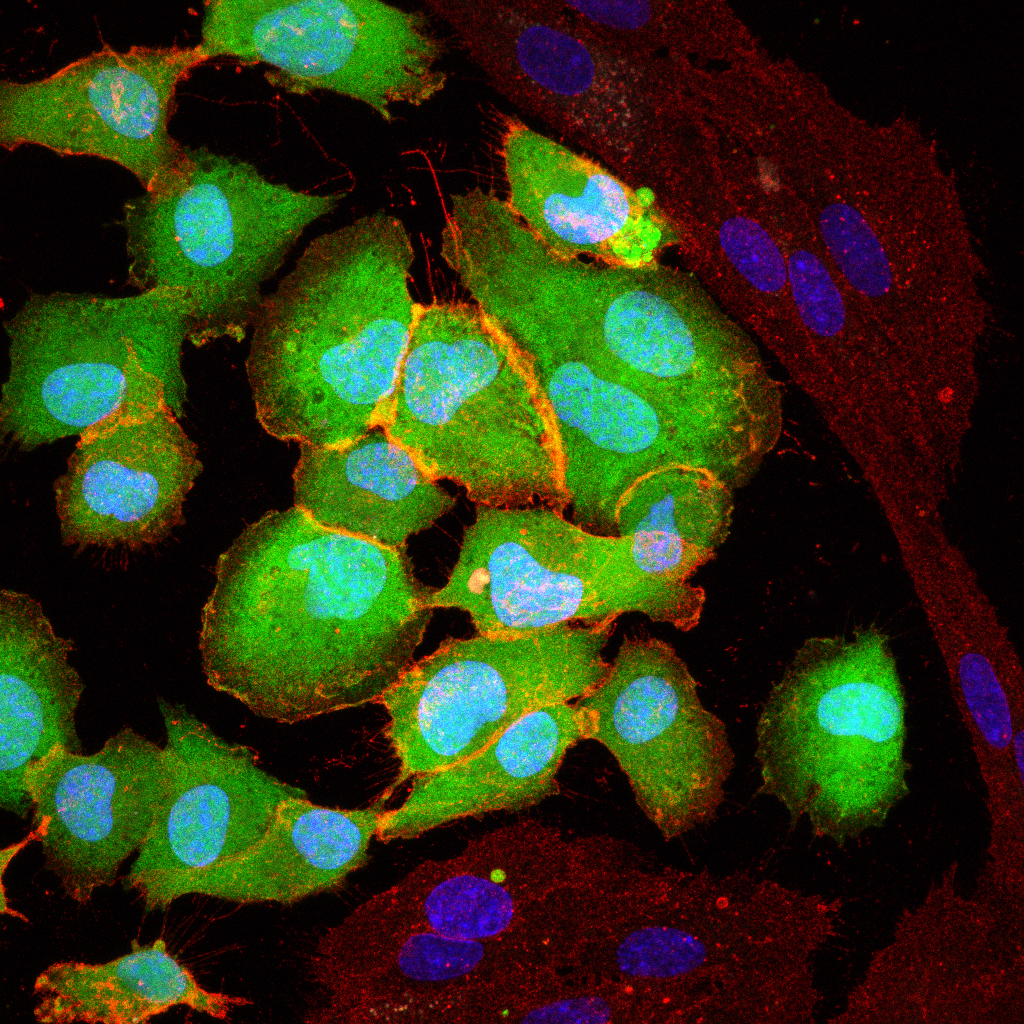

Supplement: Soure data 3. [file elife-32490-fig3.zip › Figure 5/Panel f/siCtrl/LEC_siSCR_totalITGB1_Maximum intensity projection_c1+2+3.tif]

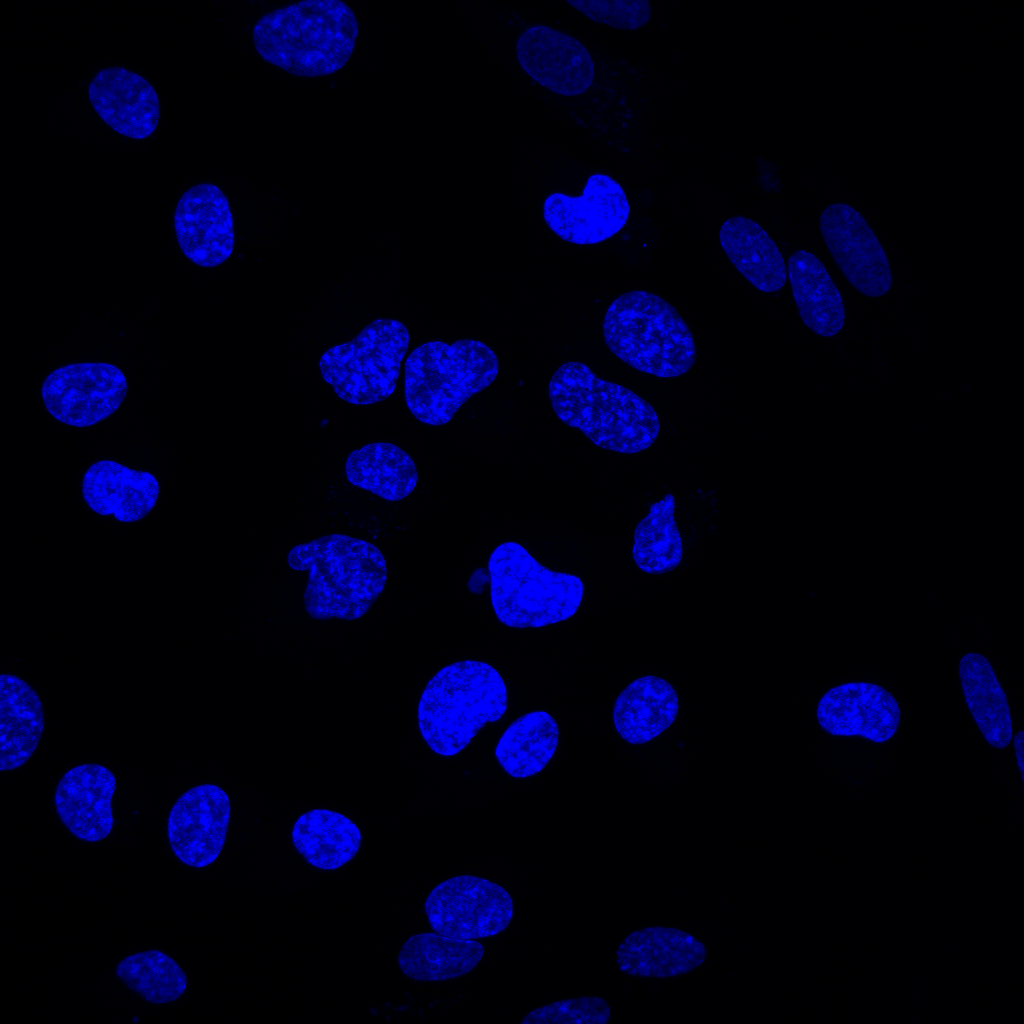

Supplement: Soure data 3. [file elife-32490-fig3.zip › Figure 5/Panel f/siCtrl/LEC_siSCR_totalITGB1_Maximum intensity projection_c2.tif]

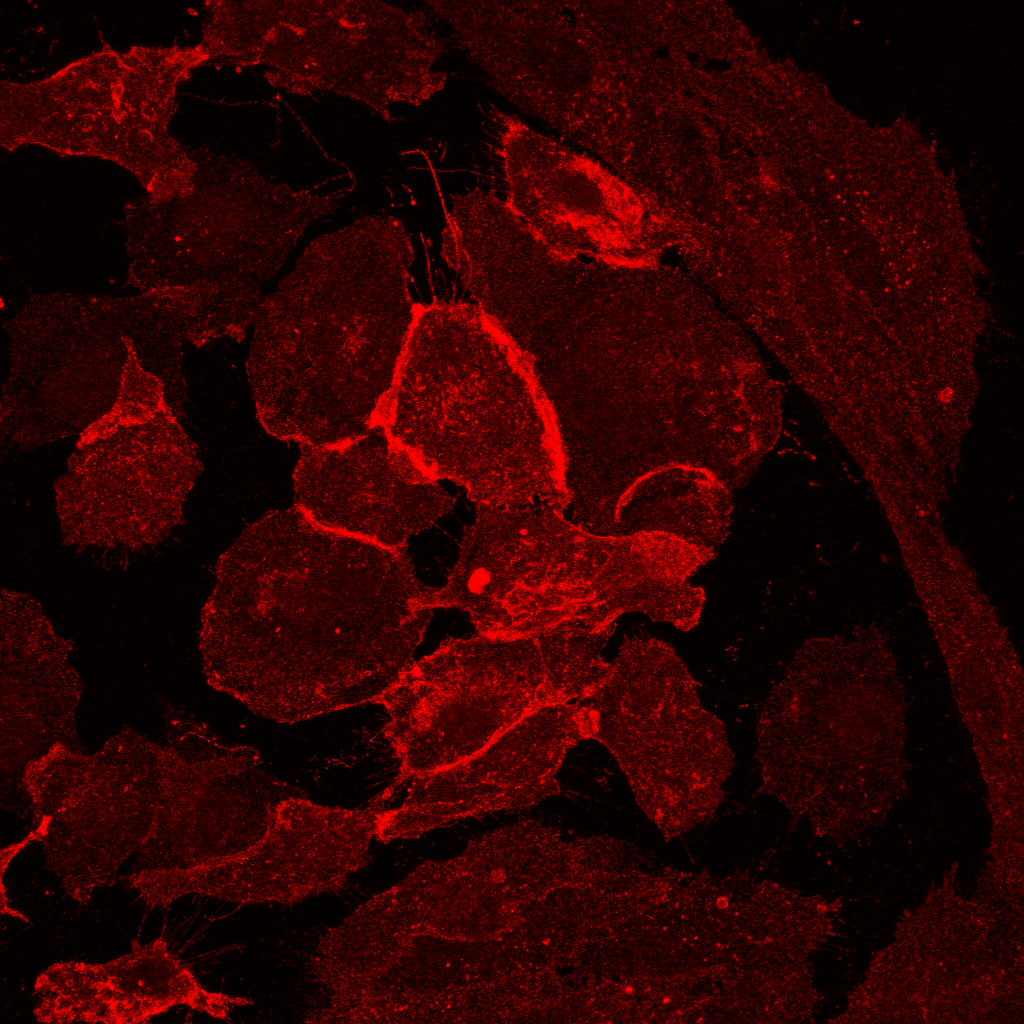

Supplement: Soure data 3. [file elife-32490-fig3.zip › Figure 5/Panel f/siCtrl/LEC_siSCR_totalITGB1_Maximum intensity projection_c3.tif]

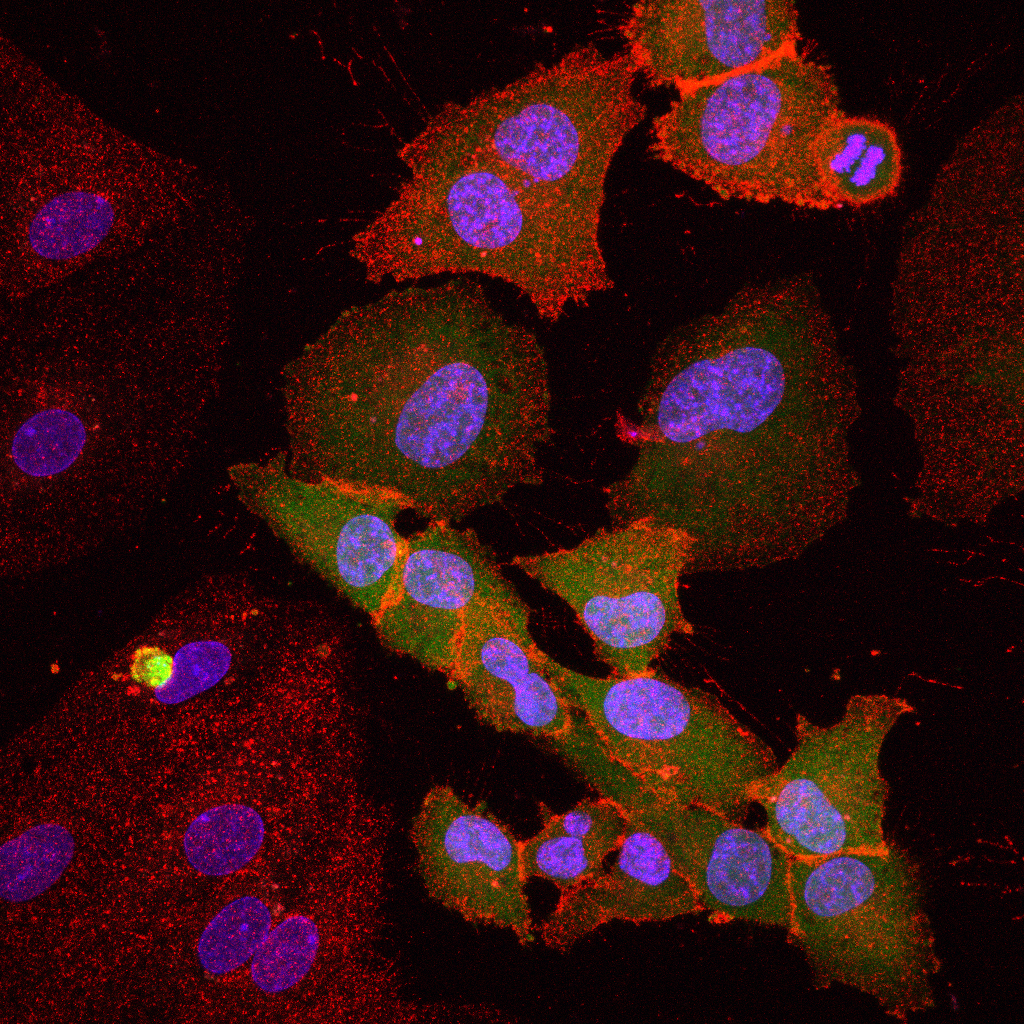

Supplement: Soure data 3. [file elife-32490-fig3.zip › Figure 5/Panel f/siMMP14/siMMP14_Maximum intensity projection.tif]

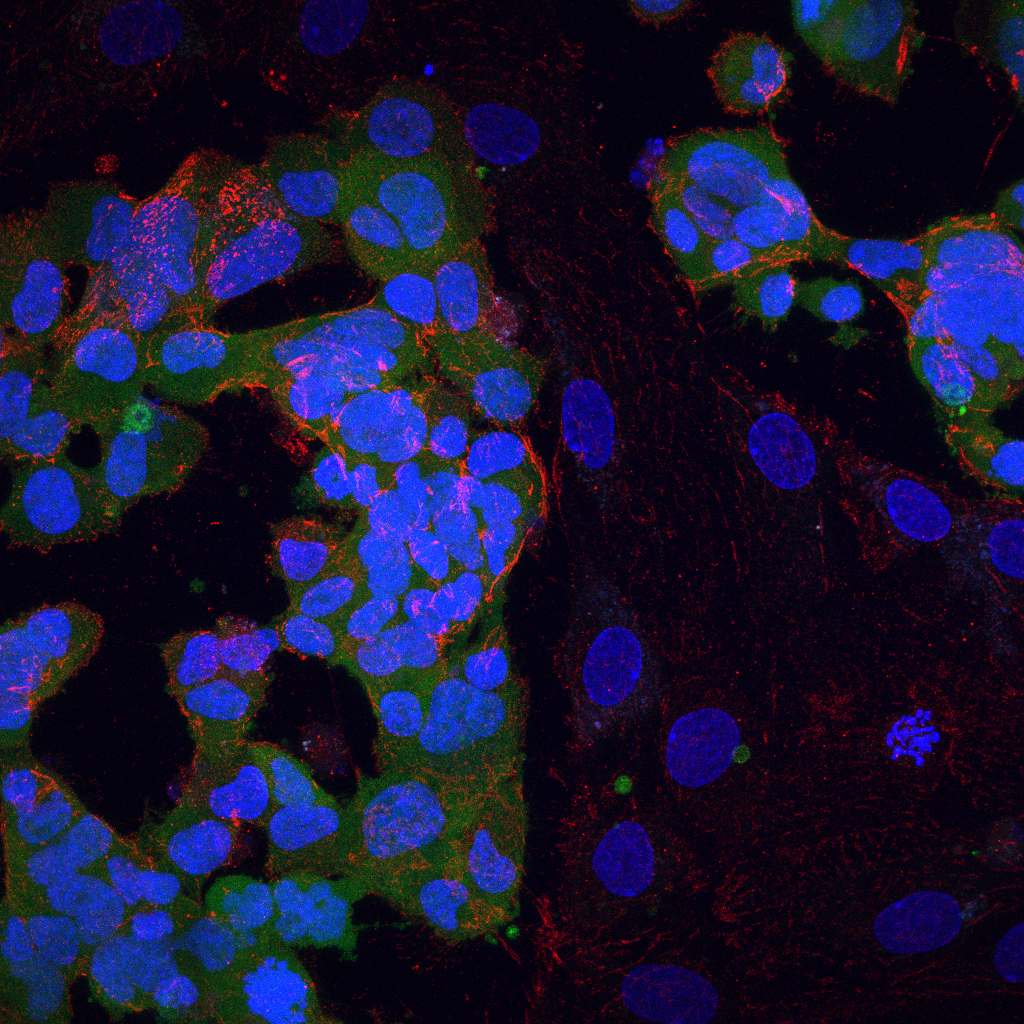

Supplement: Soure data 4. [file elife-32490-fig4.zip › Figure 5 - figure supplement 2/Panel a/siCtrl/Image 1_Maximum intensity projection_c1+2+3.tif]

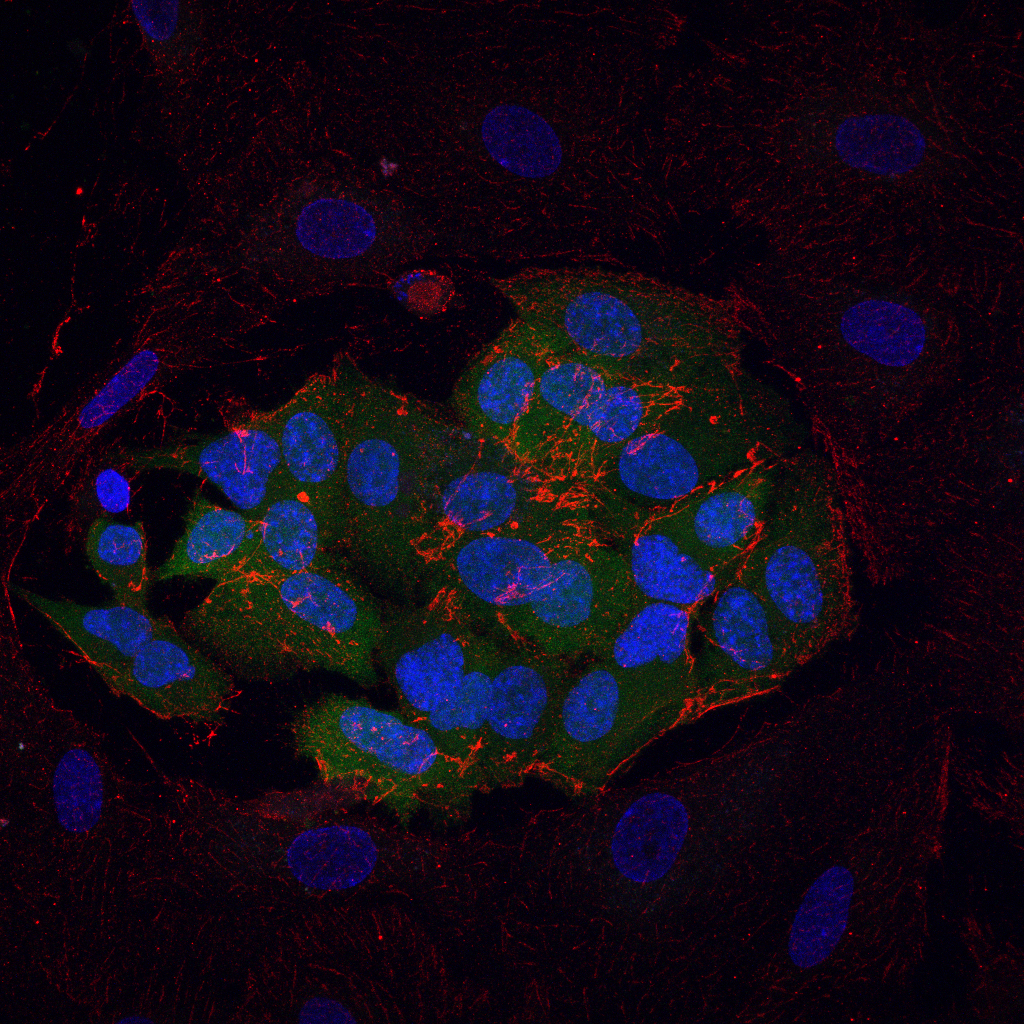

Supplement: Soure data 4. [file elife-32490-fig4.zip › Figure 5 - figure supplement 2/Panel a/siNotch3/LEC_siNotch3_12G10_Maximum intensity projection_c1+2+3.tif]

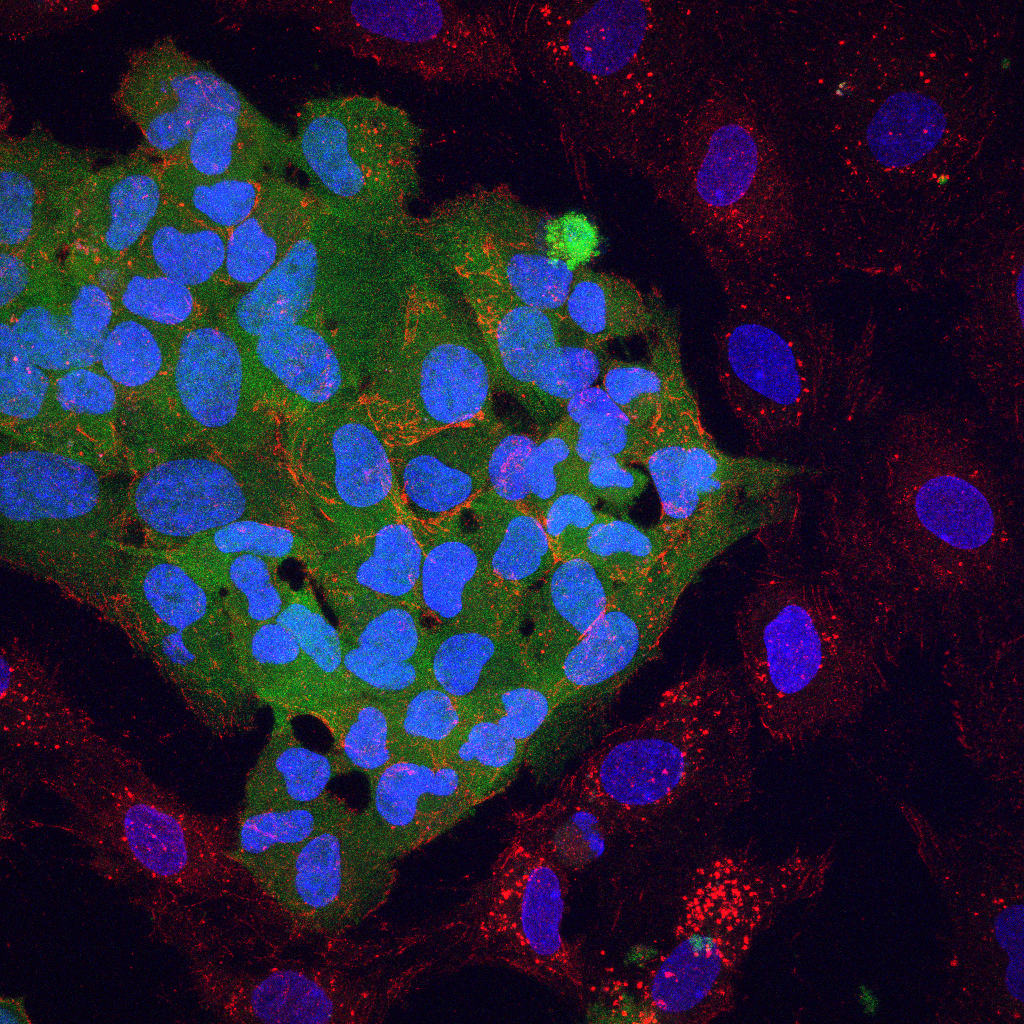

Supplement: Soure data 4. [file elife-32490-fig4.zip › Figure 5 - figure supplement 2/Panel b/siCtrl/Image 59_Maximum intensity projection.tif]

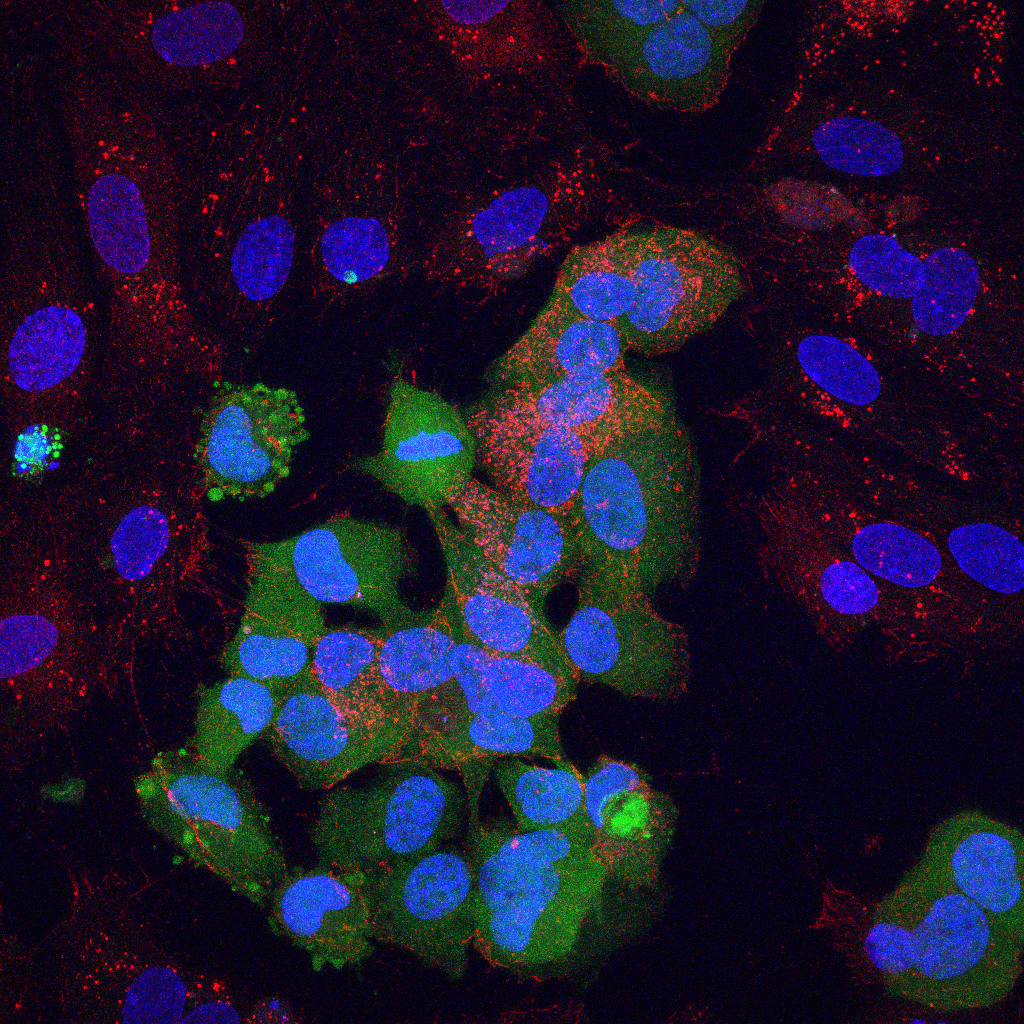

Supplement: Soure data 4. [file elife-32490-fig4.zip › Figure 5 - figure supplement 2/Panel b/siNotch3/LEC_siNotch3_9eg7_Maximum intensity projection.tif]

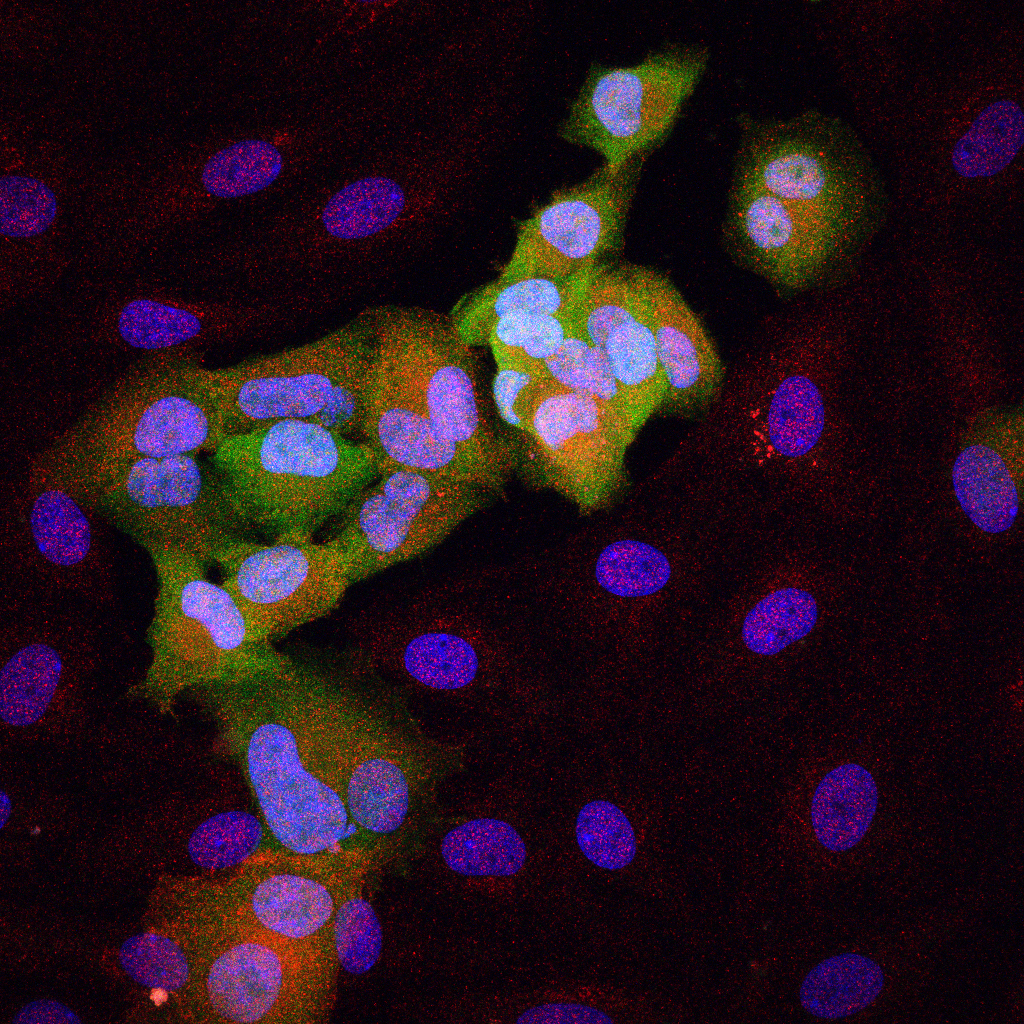

Supplement: Soure data 4. [file elife-32490-fig4.zip › Figure 5 - figure supplement 2/Panel d/AIIB2/LEC_AIIB2_Notch3_Maximum intensity projection.tif]

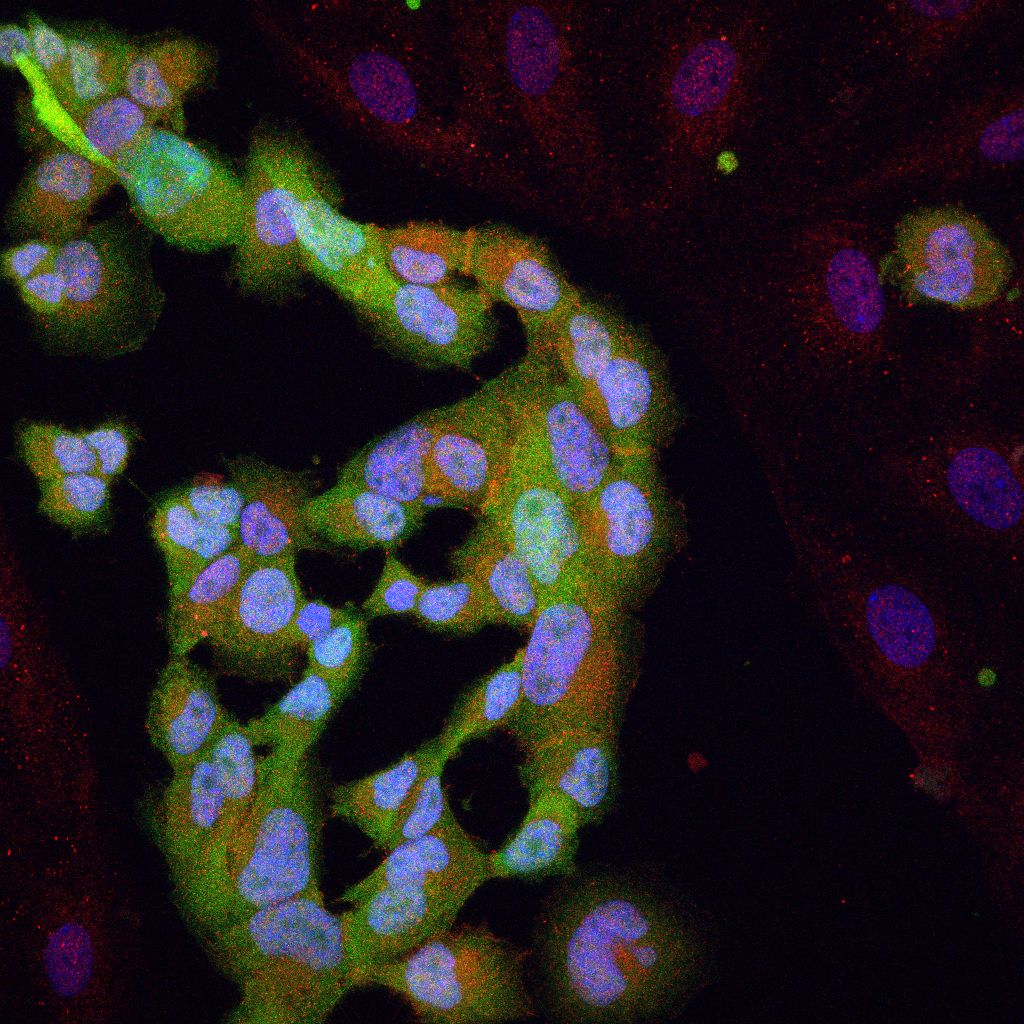

Supplement: Soure data 4. [file elife-32490-fig4.zip › Figure 5 - figure supplement 2/Panel d/Control/LEC_Mock_Notch3_Maximum intensity projection.tif]

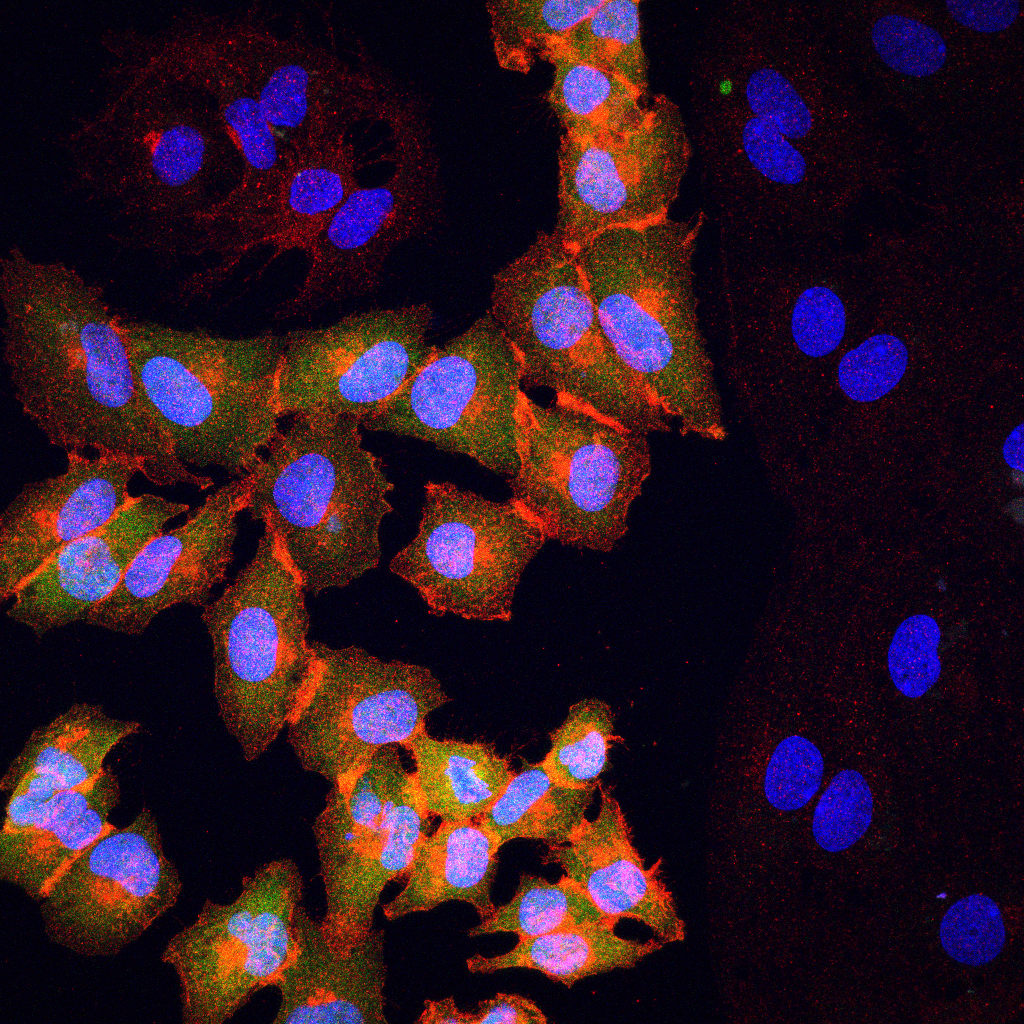

Supplement: Soure data 4. [file elife-32490-fig4.zip › Figure 5 - figure supplement 2/Panel e/AIIB2/LEC_AIIB2_MMP14_Maximum intensity projection.tif]

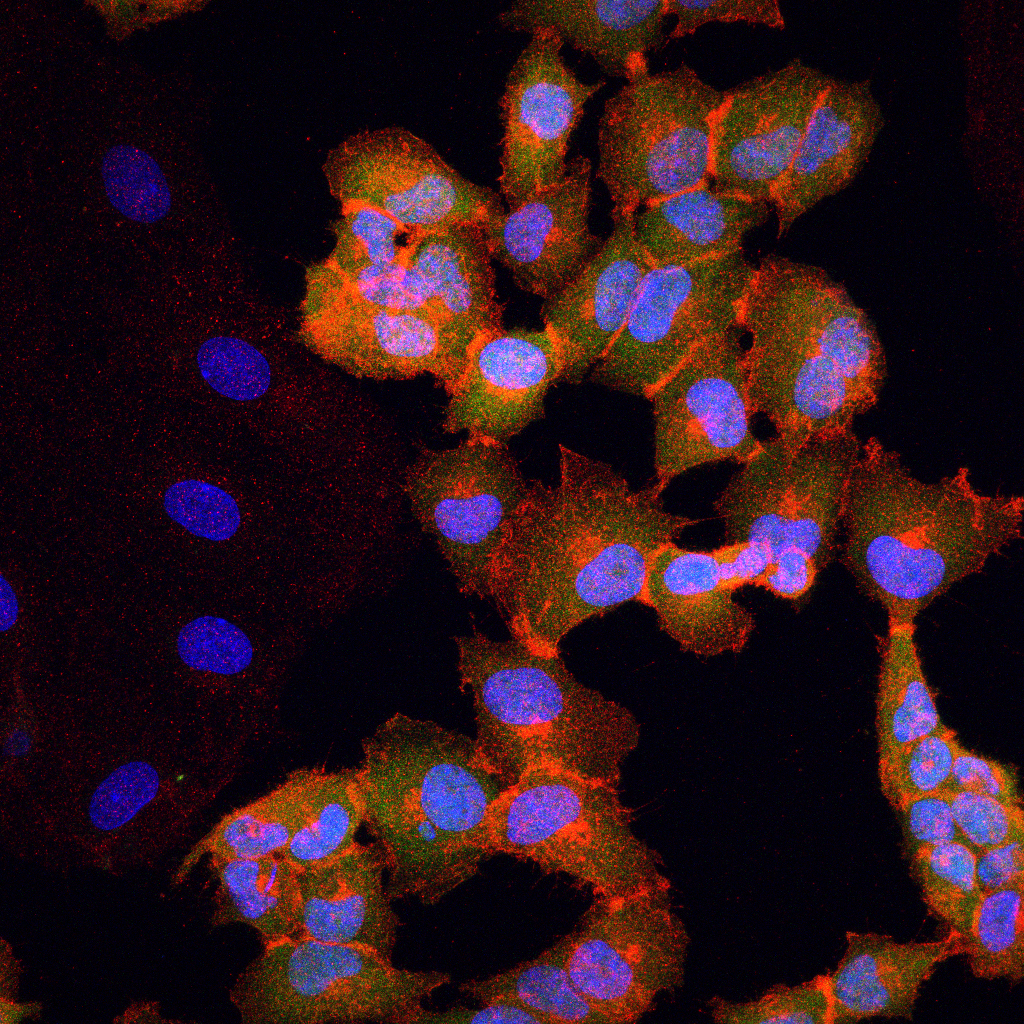

Supplement: Soure data 4. [file elife-32490-fig4.zip › Figure 5 - figure supplement 2/Panel e/Control/LEC_Mock_MMP14_Maximum intensity projection.tif]

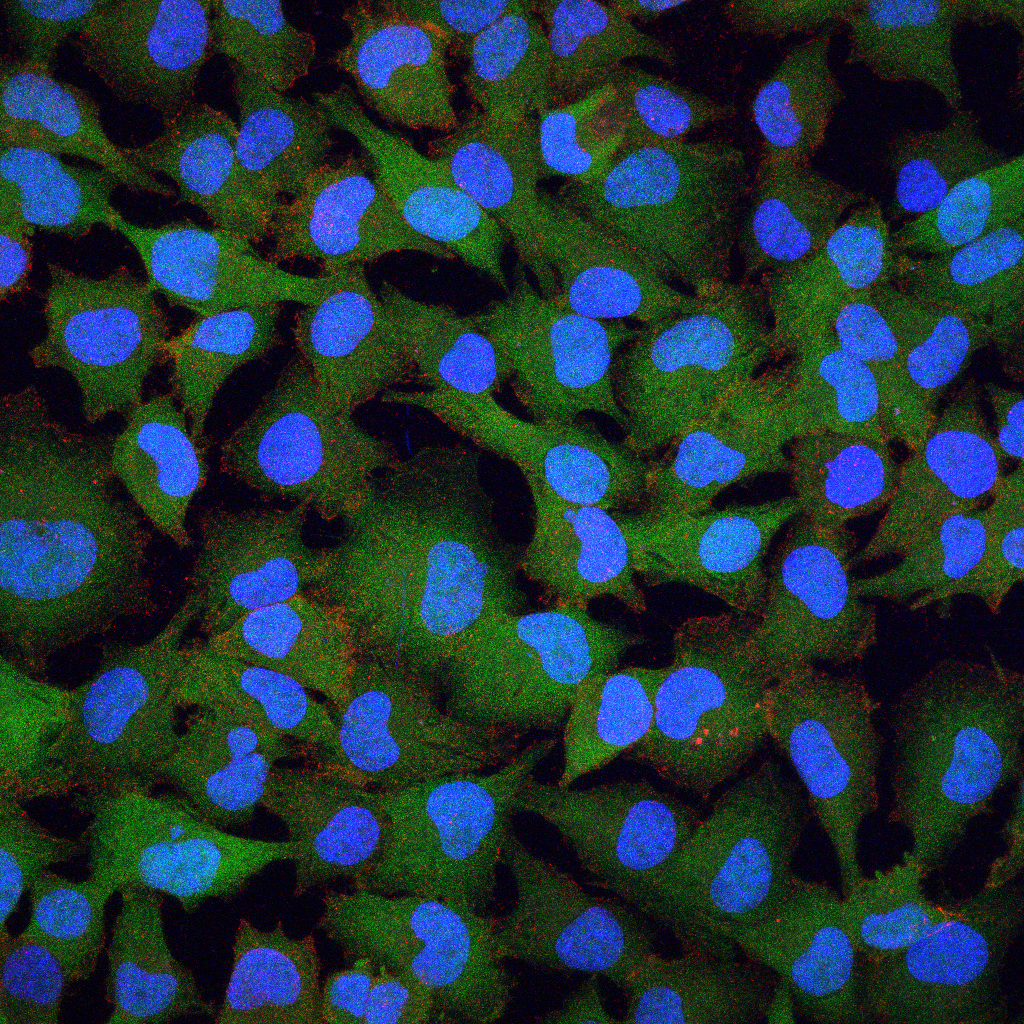

Supplement: Soure data 4. [file elife-32490-fig4.zip › Figure 5- figure supplement 1/Panel b/WM852/WM852_9eg7_Maximum intensity projection.tif]

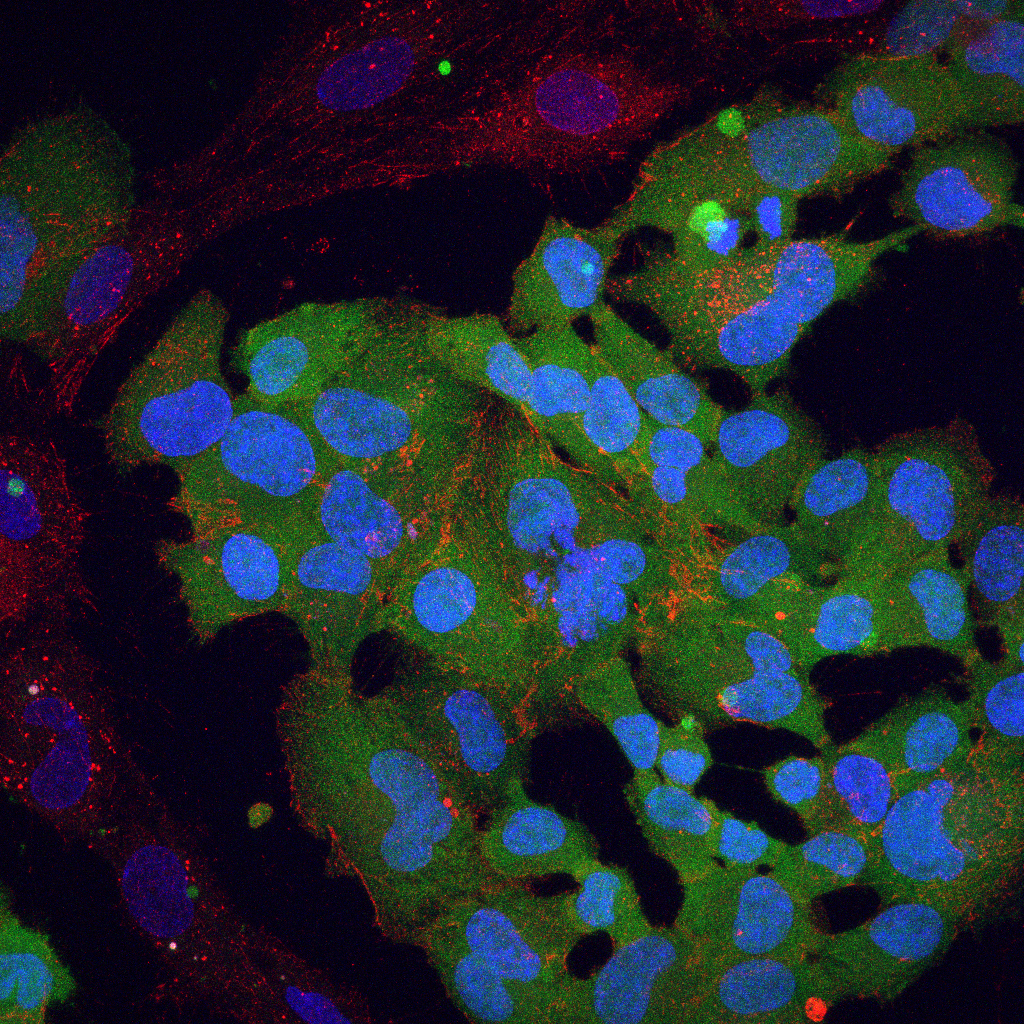

Supplement: Soure data 4. [file elife-32490-fig4.zip › Figure 5- figure supplement 1/Panel b/WM852_LEC/LEC_WM852_9eg7_Maximum intensity projection.tif]

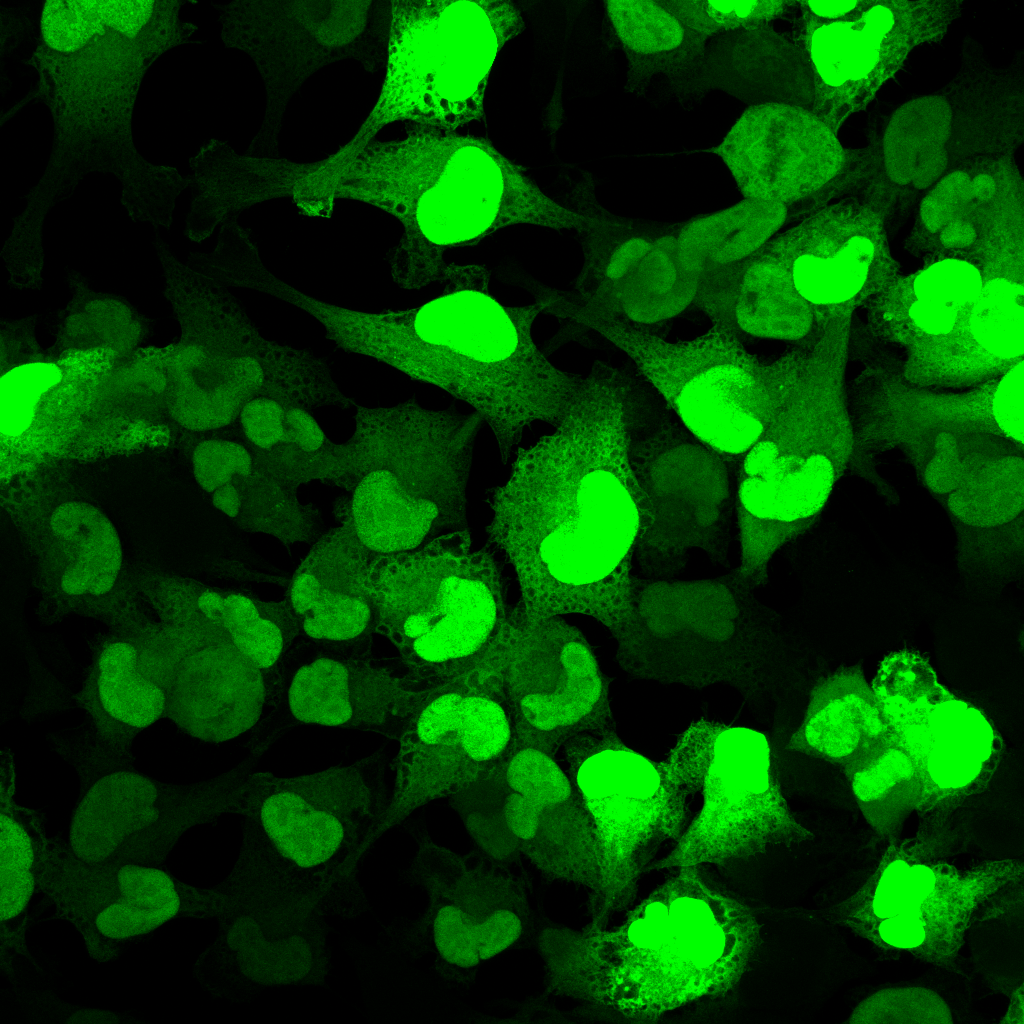

Supplement: Soure data 4. [file elife-32490-fig4.zip › Figure 5- figure supplement 1/Panel c/Bowes/Bowes_totalITGB1_594_Max_c1.tif]

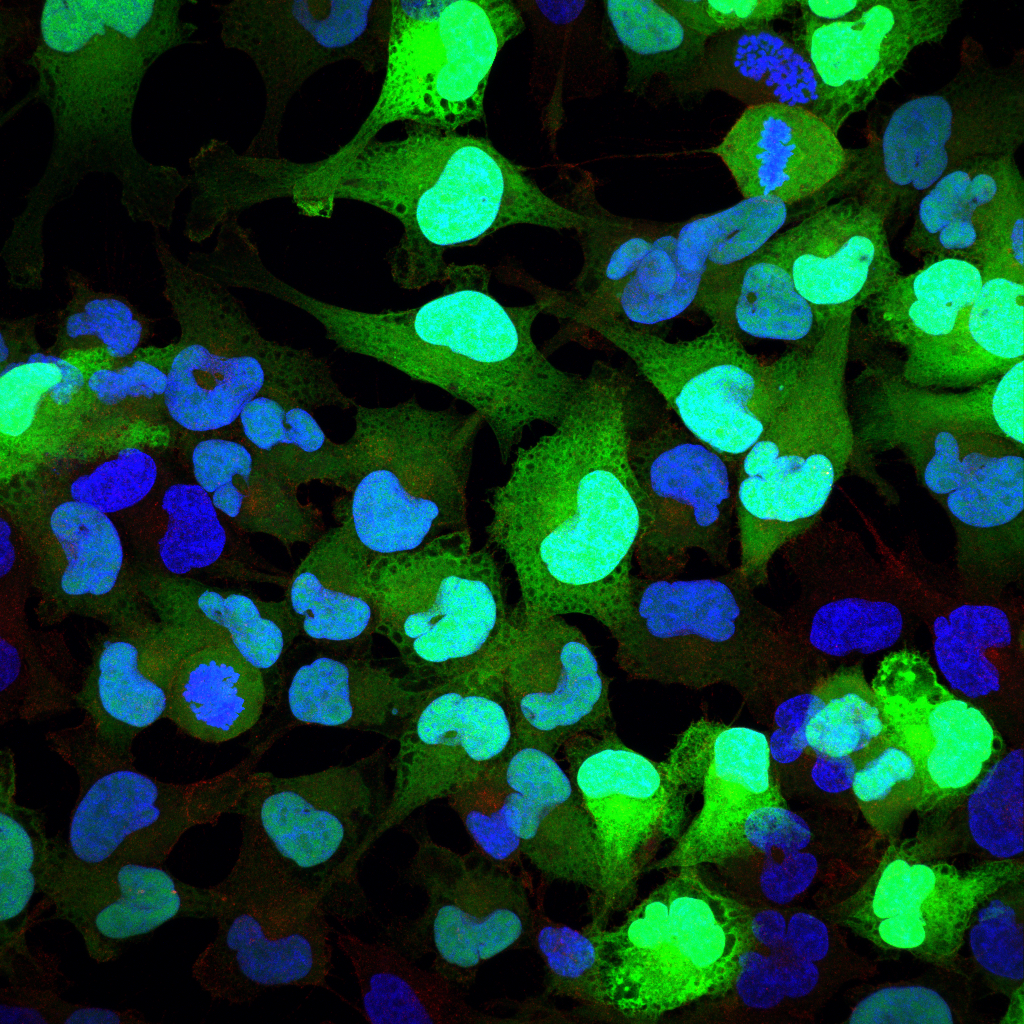

Supplement: Soure data 4. [file elife-32490-fig4.zip › Figure 5- figure supplement 1/Panel c/Bowes/Bowes_totalITGB1_594_Max_c1+2+3.tif]

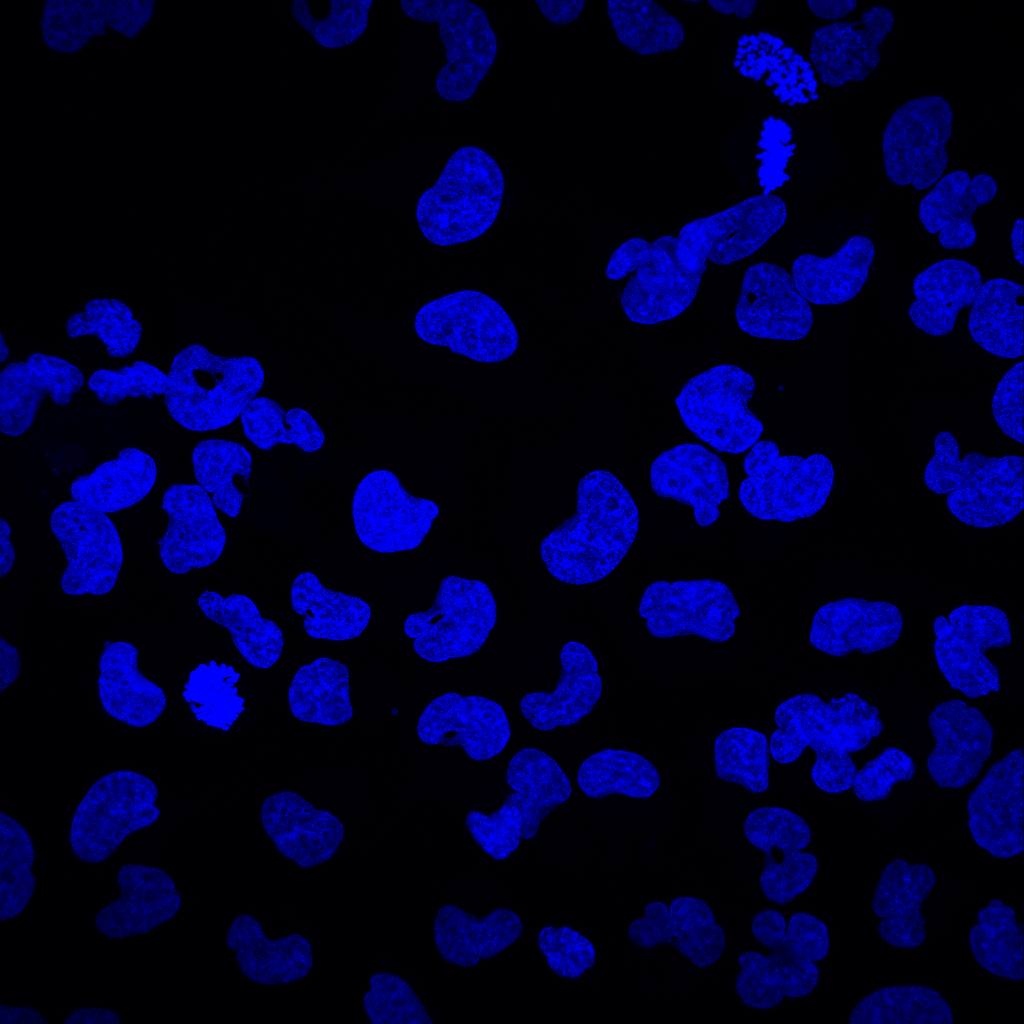

Supplement: Soure data 4. [file elife-32490-fig4.zip › Figure 5- figure supplement 1/Panel c/Bowes/Bowes_totalITGB1_594_Max_c2.tif]

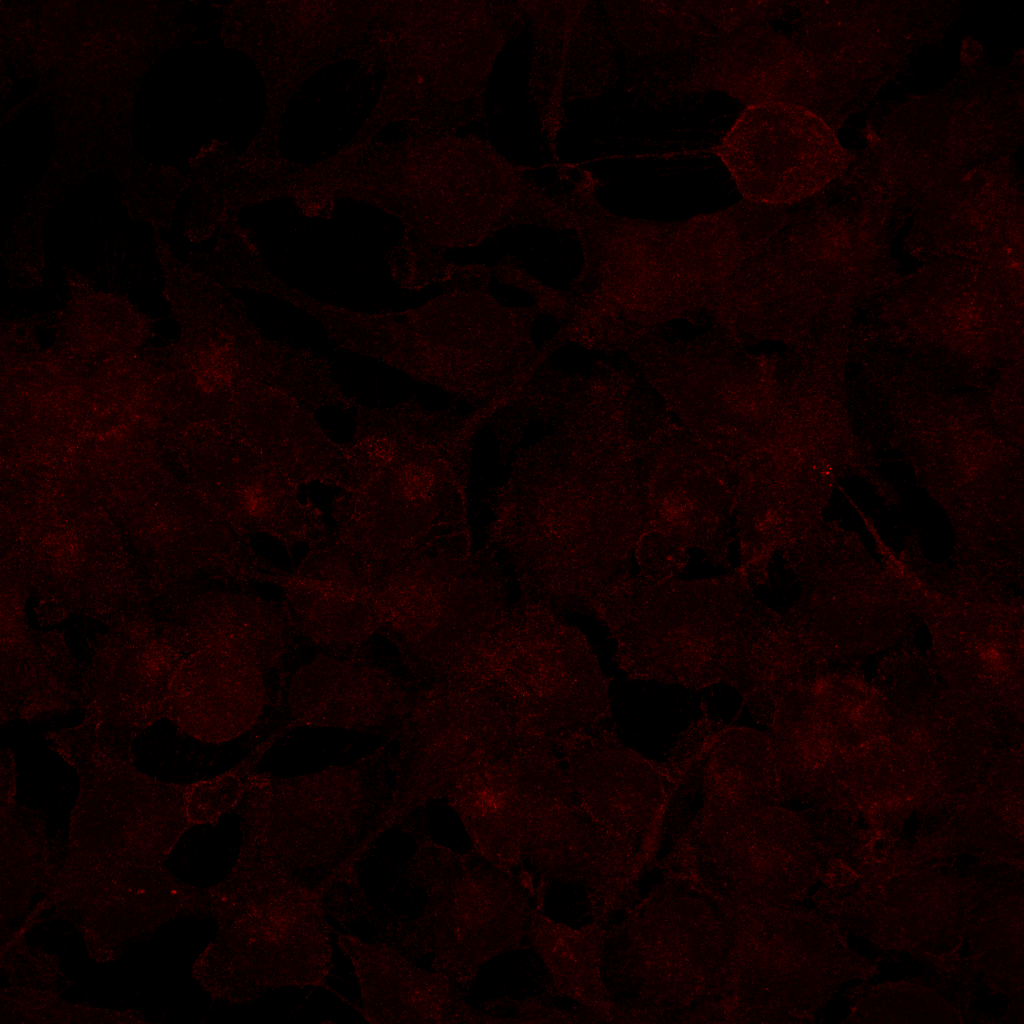

Supplement: Soure data 4. [file elife-32490-fig4.zip › Figure 5- figure supplement 1/Panel c/Bowes/Bowes_totalITGB1_594_Max_c3.tif]

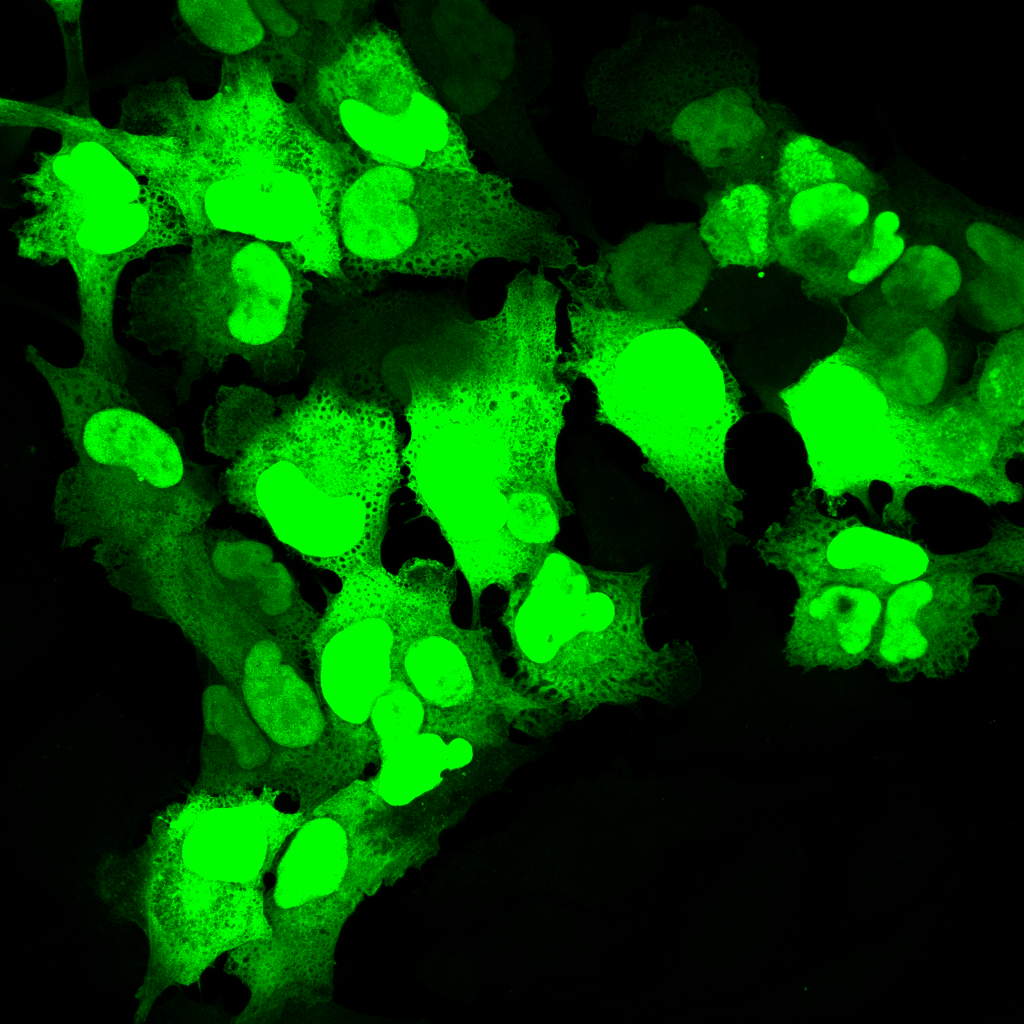

Supplement: Soure data 4. [file elife-32490-fig4.zip › Figure 5- figure supplement 1/Panel c/Bowes_LEC/LEC_Bowes_totalITGB1_594_Max_c1.tif]

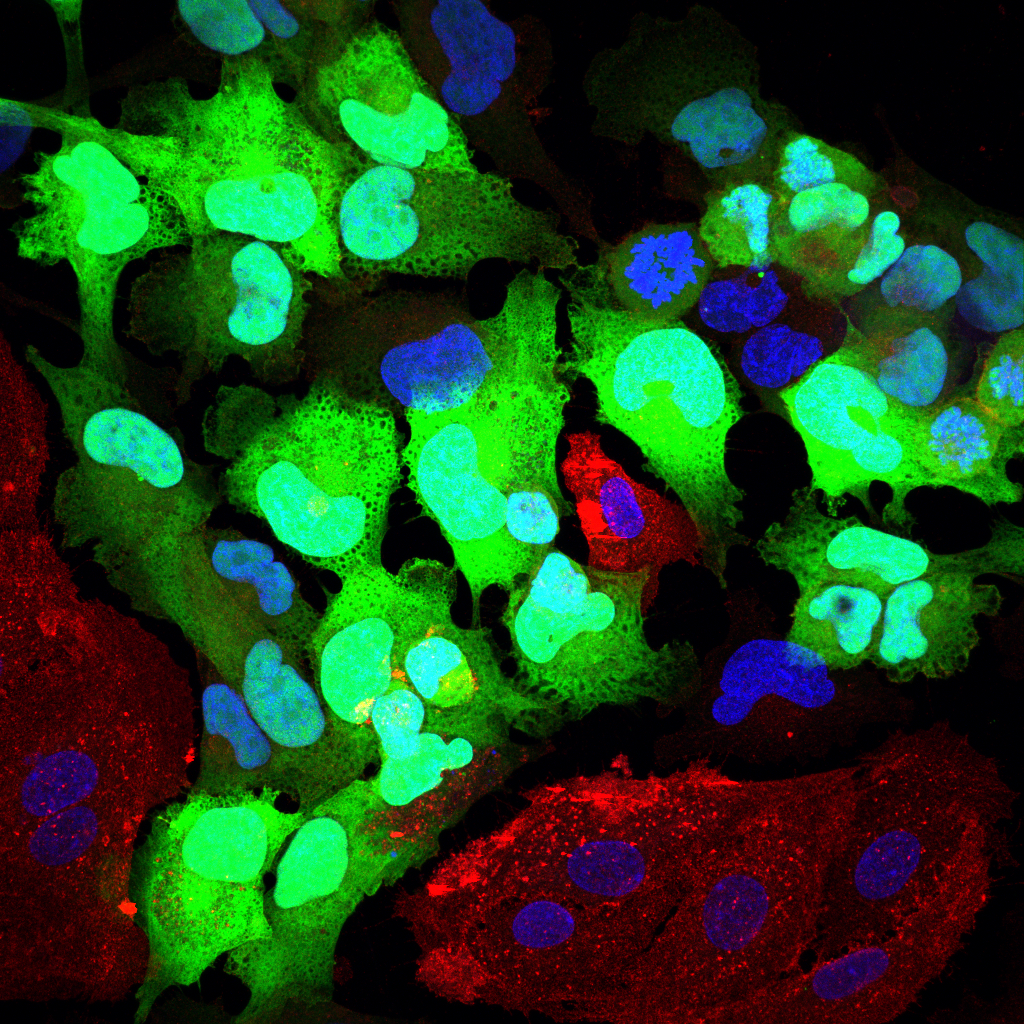

Supplement: Soure data 4. [file elife-32490-fig4.zip › Figure 5- figure supplement 1/Panel c/Bowes_LEC/LEC_Bowes_totalITGB1_594_Max_c1+2+3.tif]

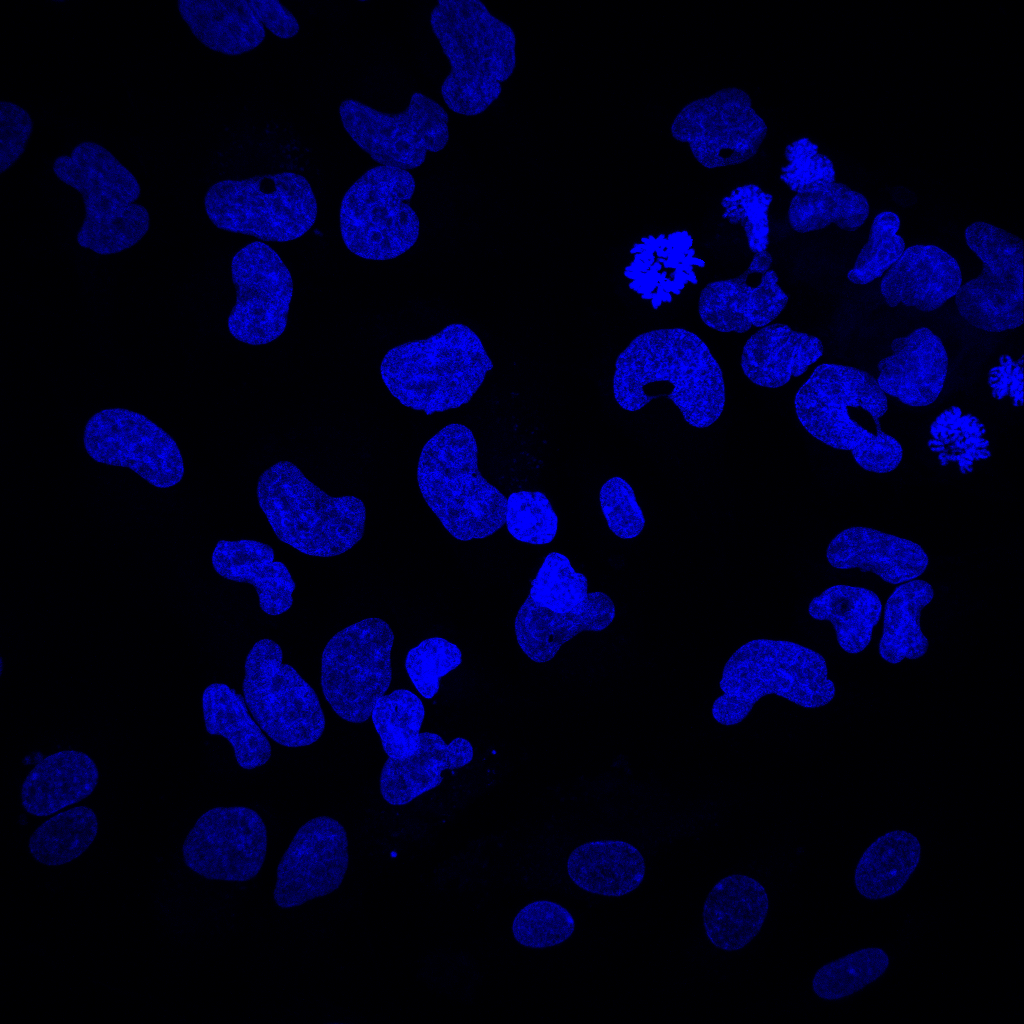

Supplement: Soure data 4. [file elife-32490-fig4.zip › Figure 5- figure supplement 1/Panel c/Bowes_LEC/LEC_Bowes_totalITGB1_594_Max_c2.tif]

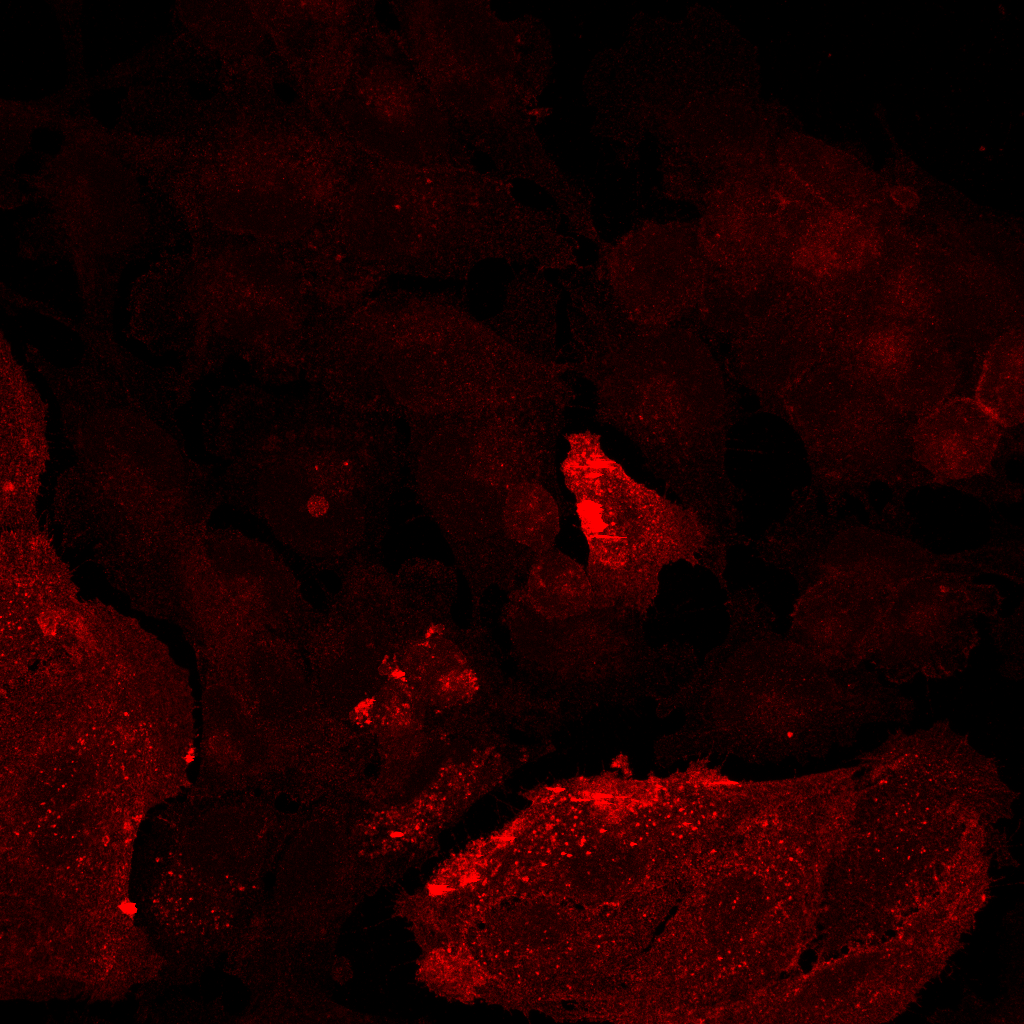

Supplement: Soure data 4. [file elife-32490-fig4.zip › Figure 5- figure supplement 1/Panel c/Bowes_LEC/LEC_Bowes_totalITGB1_594_Max_c3.tif]

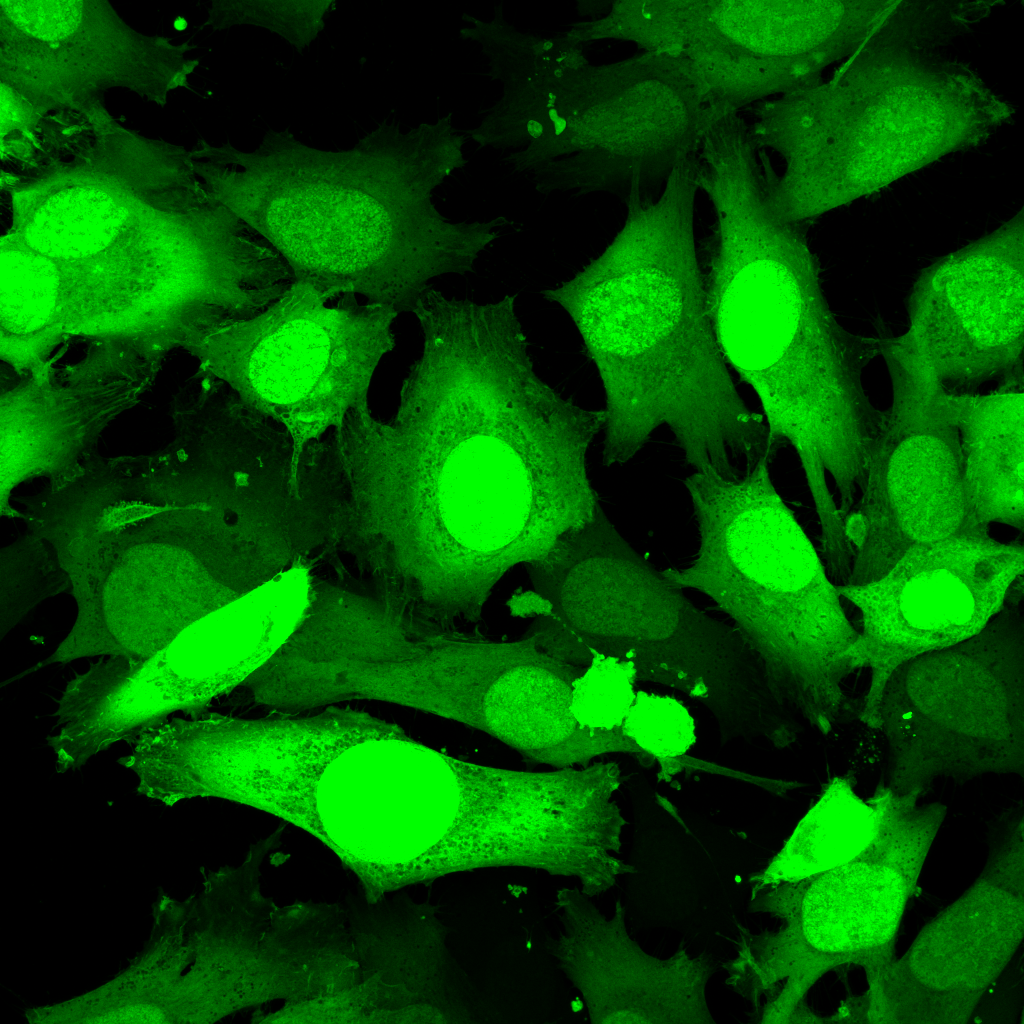

Supplement: Soure data 4. [file elife-32490-fig4.zip › Figure 5- figure supplement 1/Panel c/WM165/WM165_totalITGB1_594_Max_c1.tif]

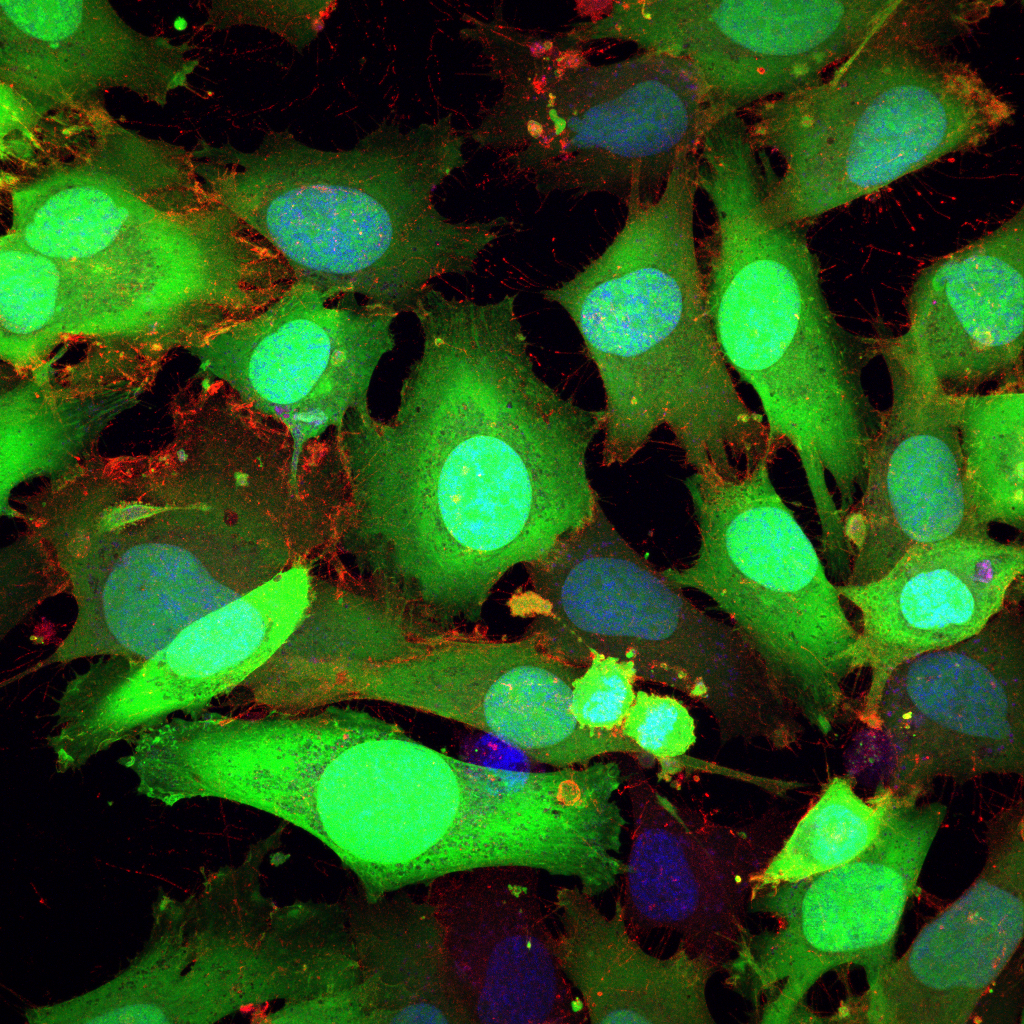

Supplement: Soure data 4. [file elife-32490-fig4.zip › Figure 5- figure supplement 1/Panel c/WM165/WM165_totalITGB1_594_Max_c1+2+3.tif]

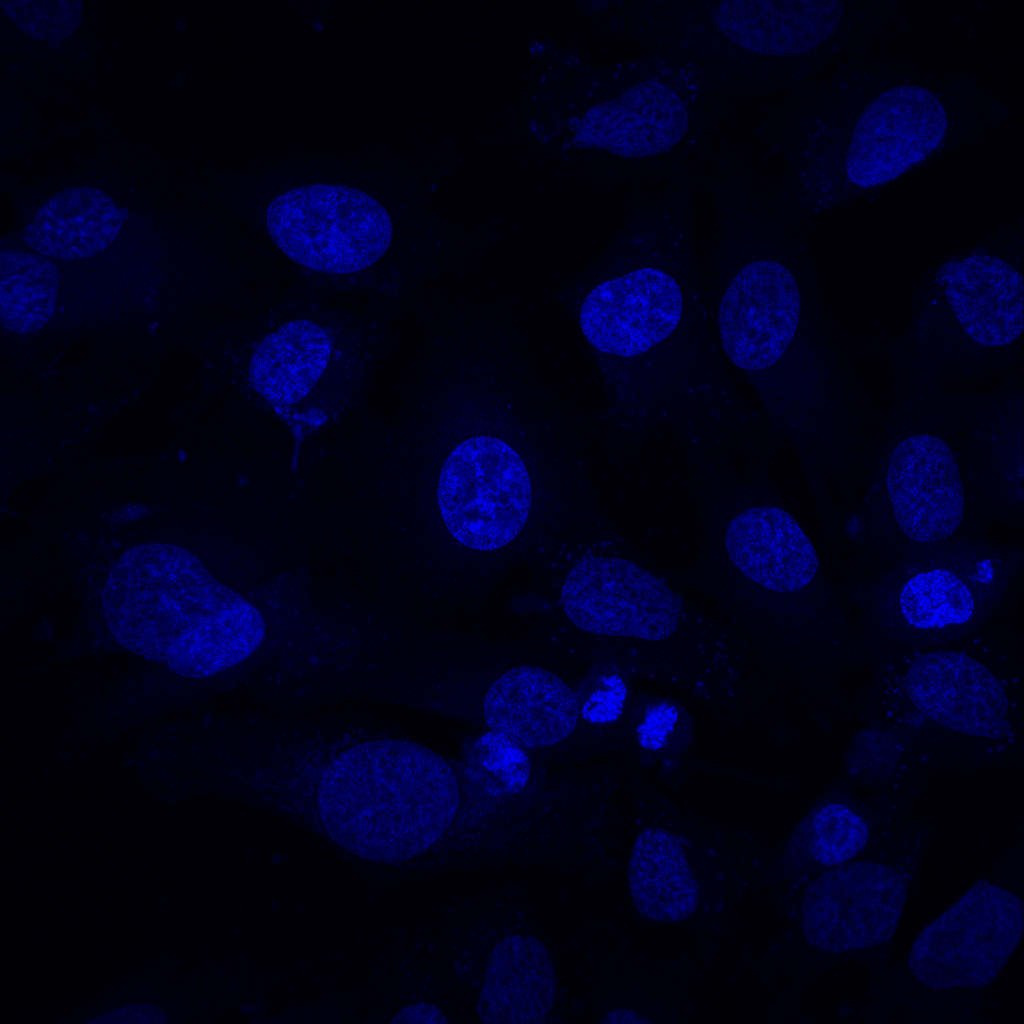

Supplement: Soure data 4. [file elife-32490-fig4.zip › Figure 5- figure supplement 1/Panel c/WM165/WM165_totalITGB1_594_Max_c2.tif]

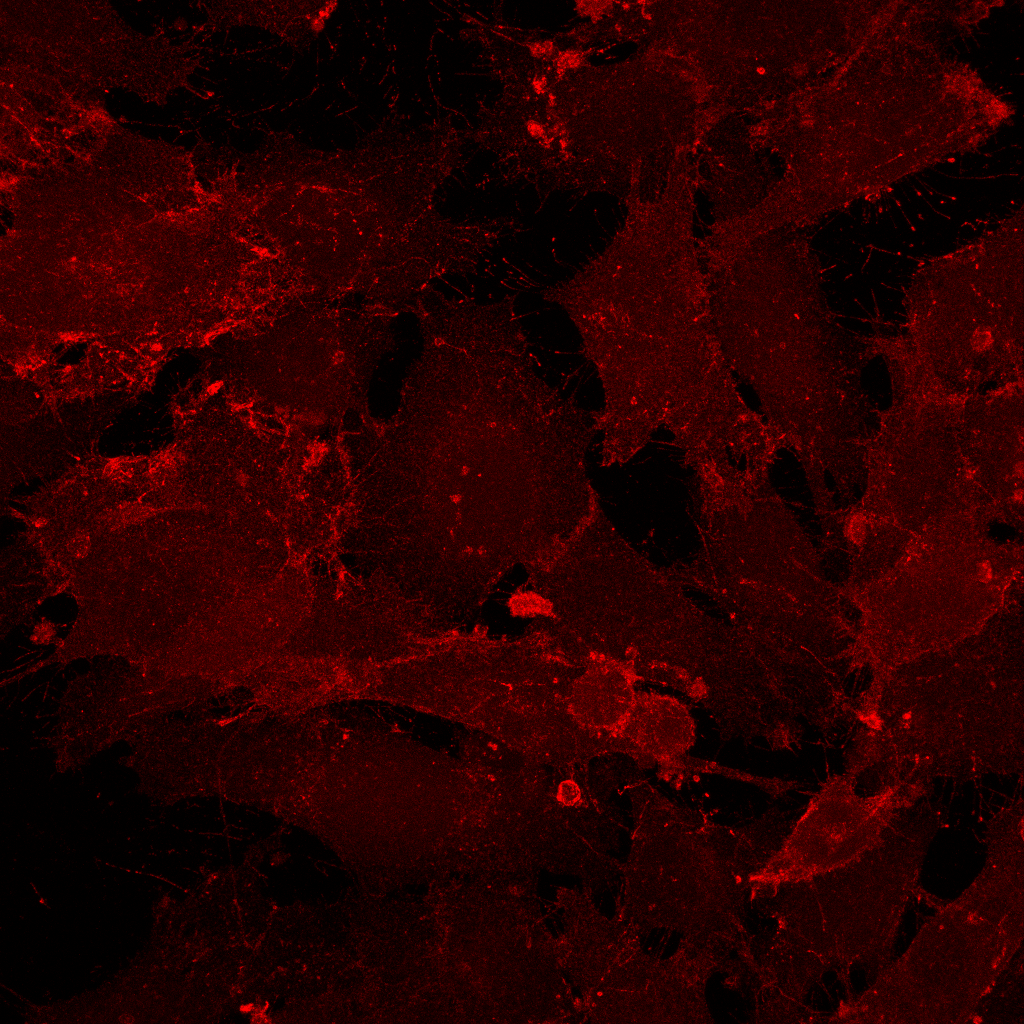

Supplement: Soure data 4. [file elife-32490-fig4.zip › Figure 5- figure supplement 1/Panel c/WM165/WM165_totalITGB1_594_Max_c3.tif]

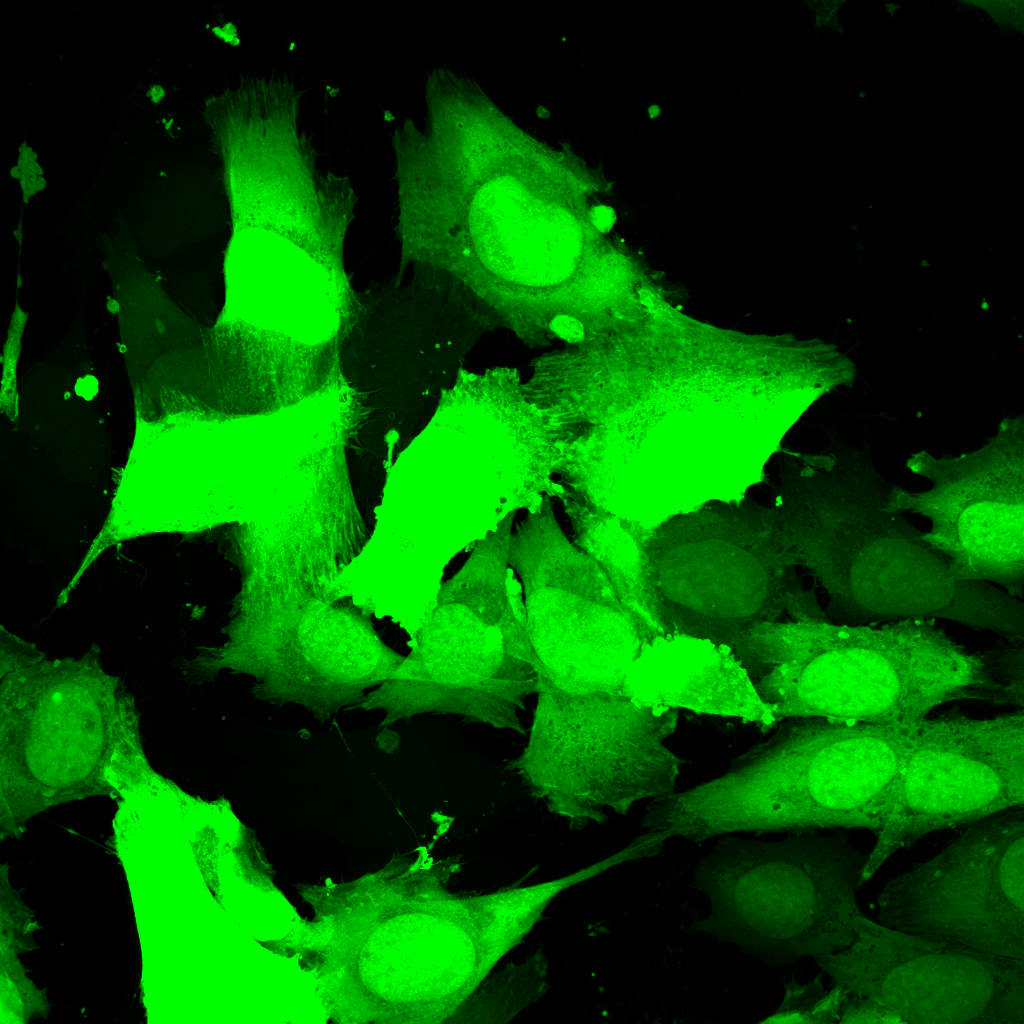

Supplement: Soure data 4. [file elife-32490-fig4.zip › Figure 5- figure supplement 1/Panel c/WM165_LEC/WM165_totalITGB1_594_Max_c1.tif]

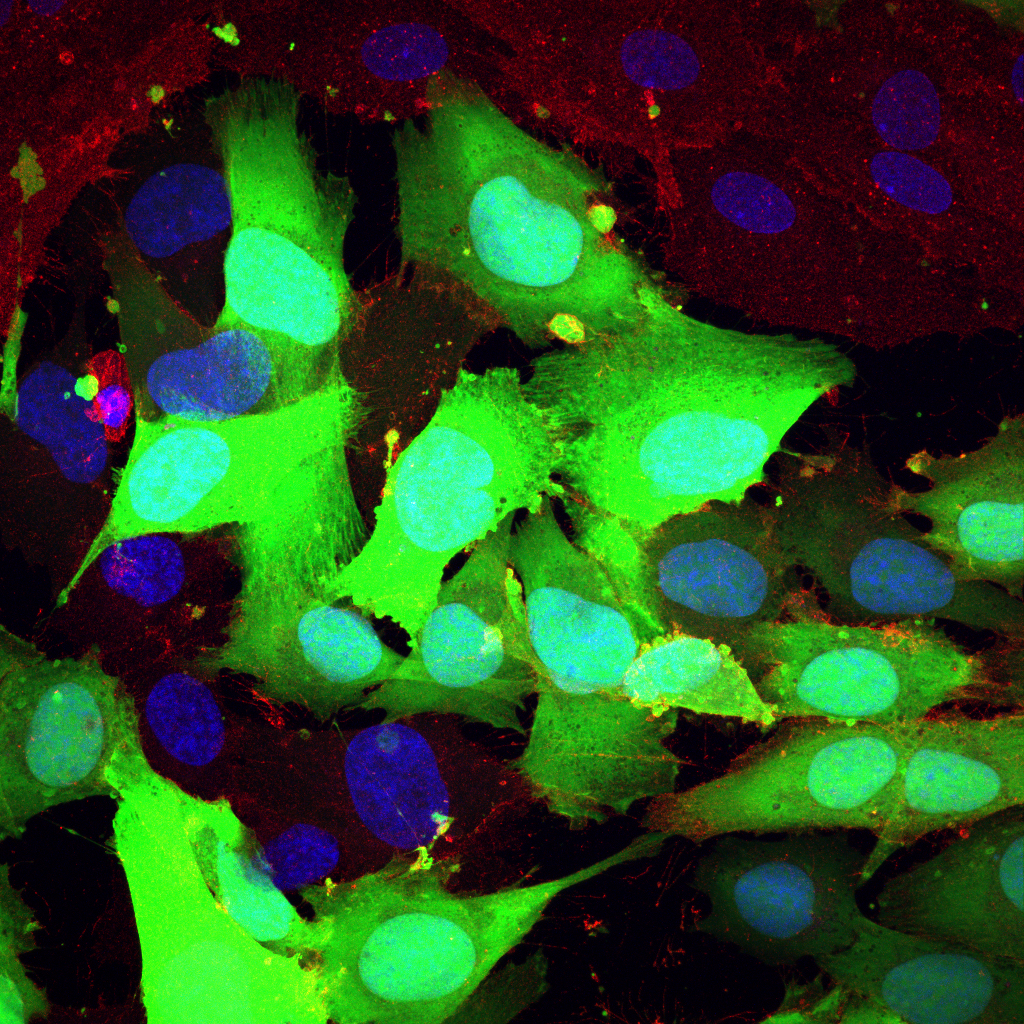

Supplement: Soure data 4. [file elife-32490-fig4.zip › Figure 5- figure supplement 1/Panel c/WM165_LEC/WM165_totalITGB1_594_Max_c1+2+3.tif]

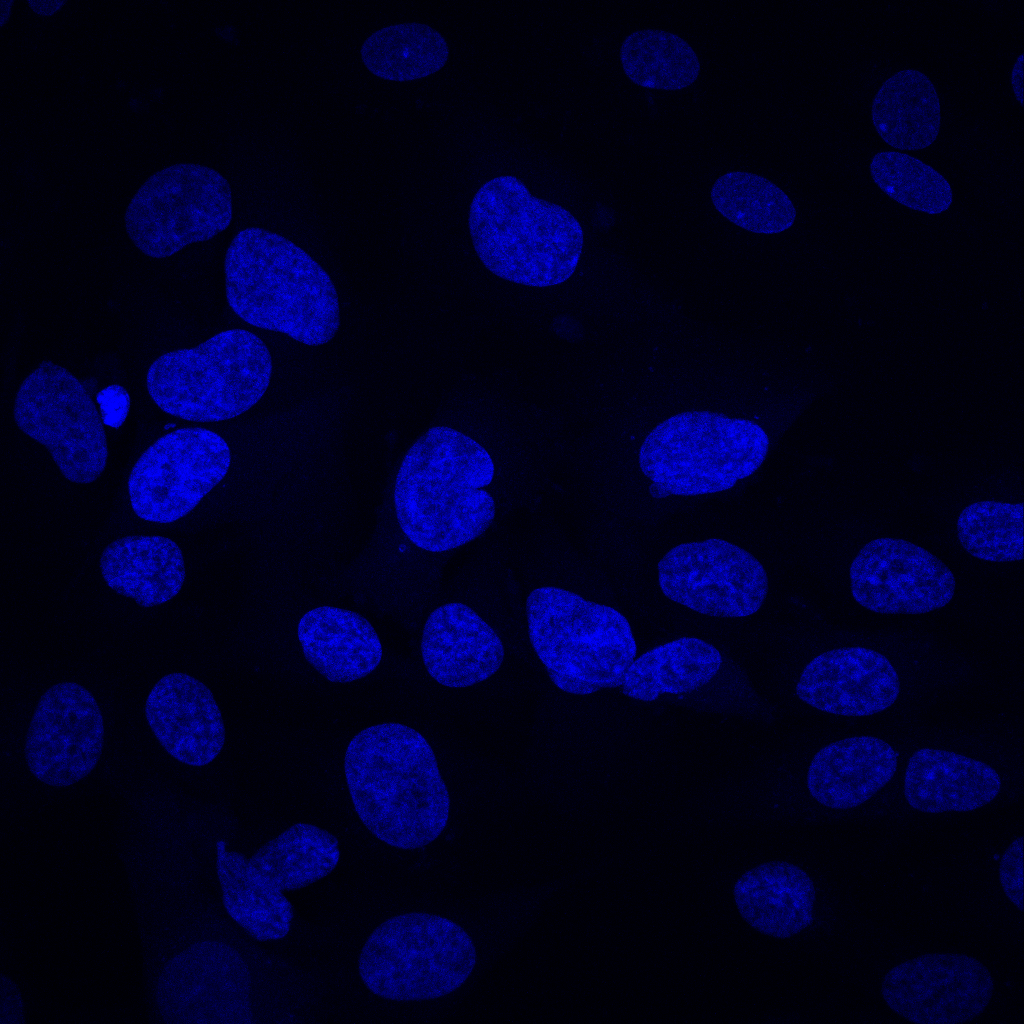

Supplement: Soure data 4. [file elife-32490-fig4.zip › Figure 5- figure supplement 1/Panel c/WM165_LEC/WM165_totalITGB1_594_Max_c2.tif]

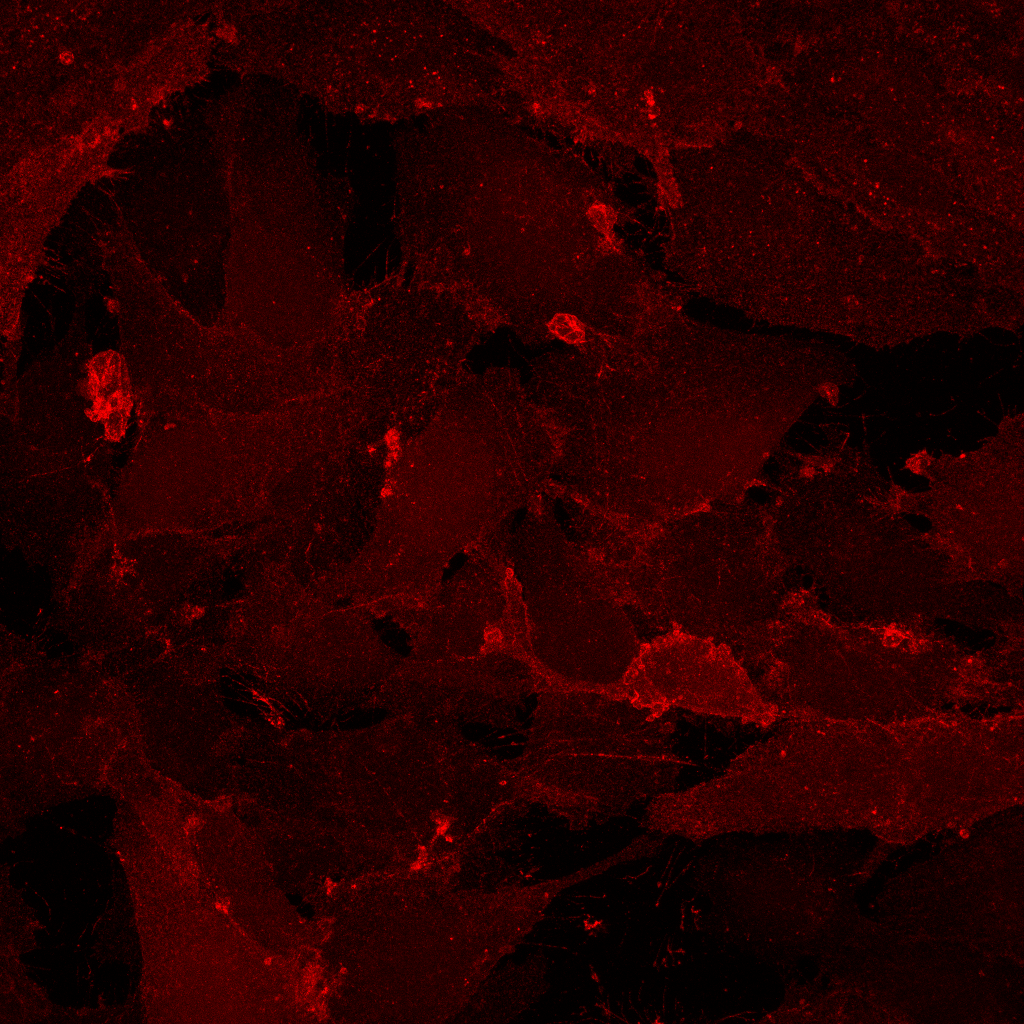

Supplement: Soure data 4. [file elife-32490-fig4.zip › Figure 5- figure supplement 1/Panel c/WM165_LEC/WM165_totalITGB1_594_Max_c3.tif]

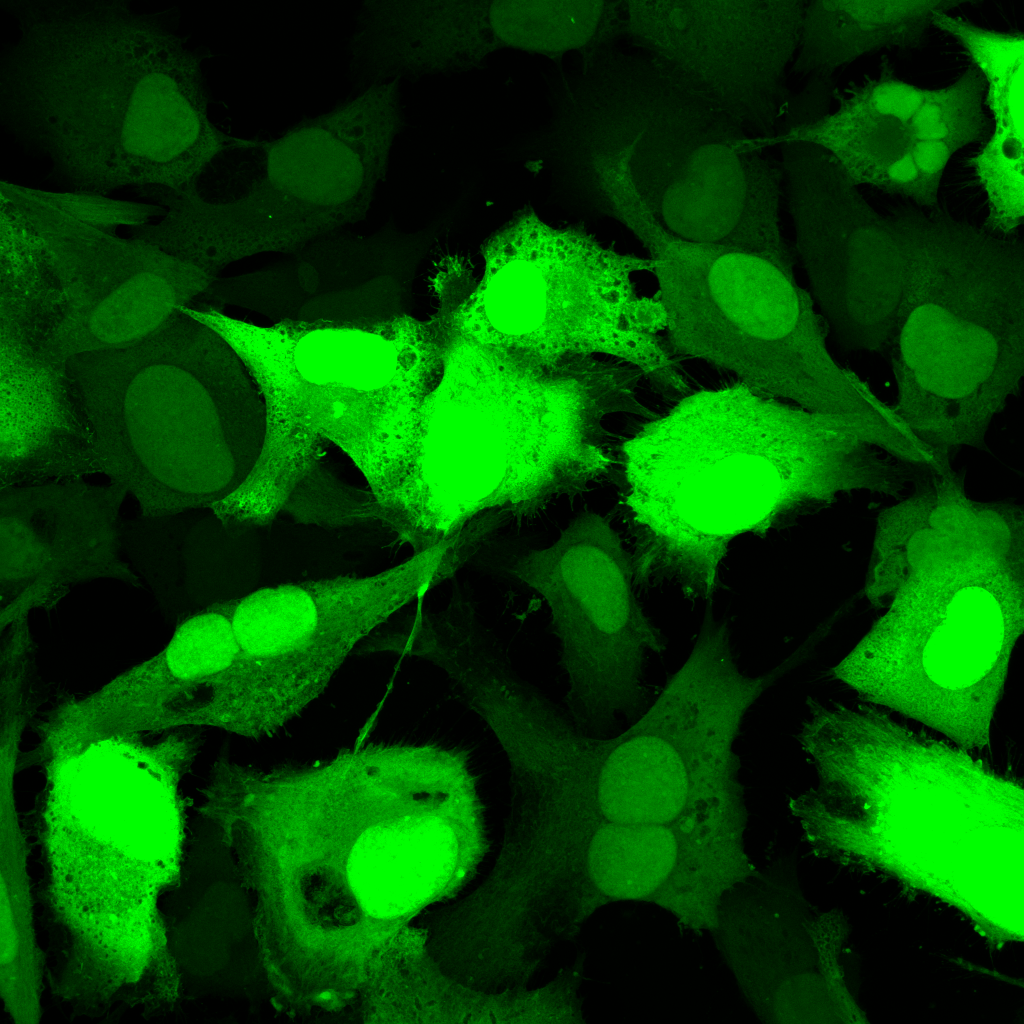

Supplement: Soure data 4. [file elife-32490-fig4.zip › Figure 5- figure supplement 1/Panel c/WM793/WM793_totalITGB1_594_Max_c1.tif]

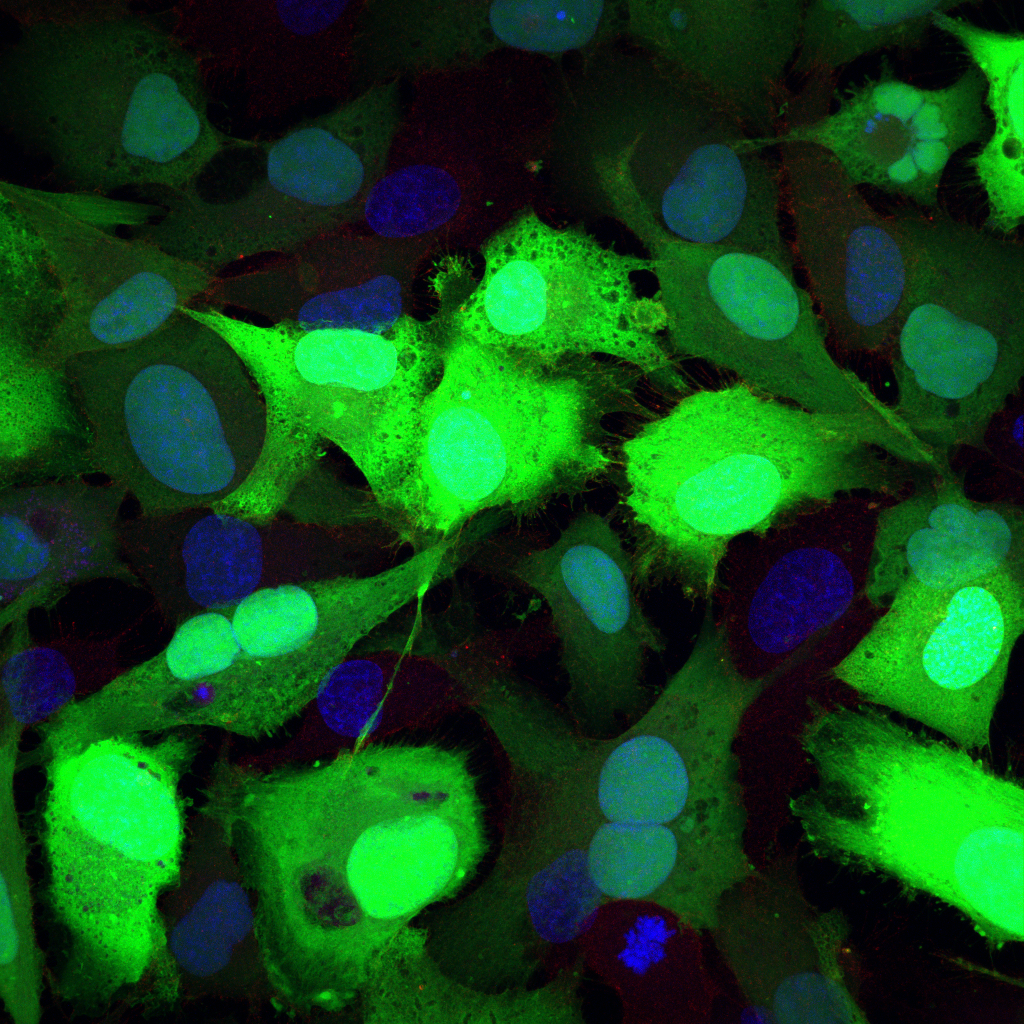

Supplement: Soure data 4. [file elife-32490-fig4.zip › Figure 5- figure supplement 1/Panel c/WM793/WM793_totalITGB1_594_Max_c1+2+3.tif]

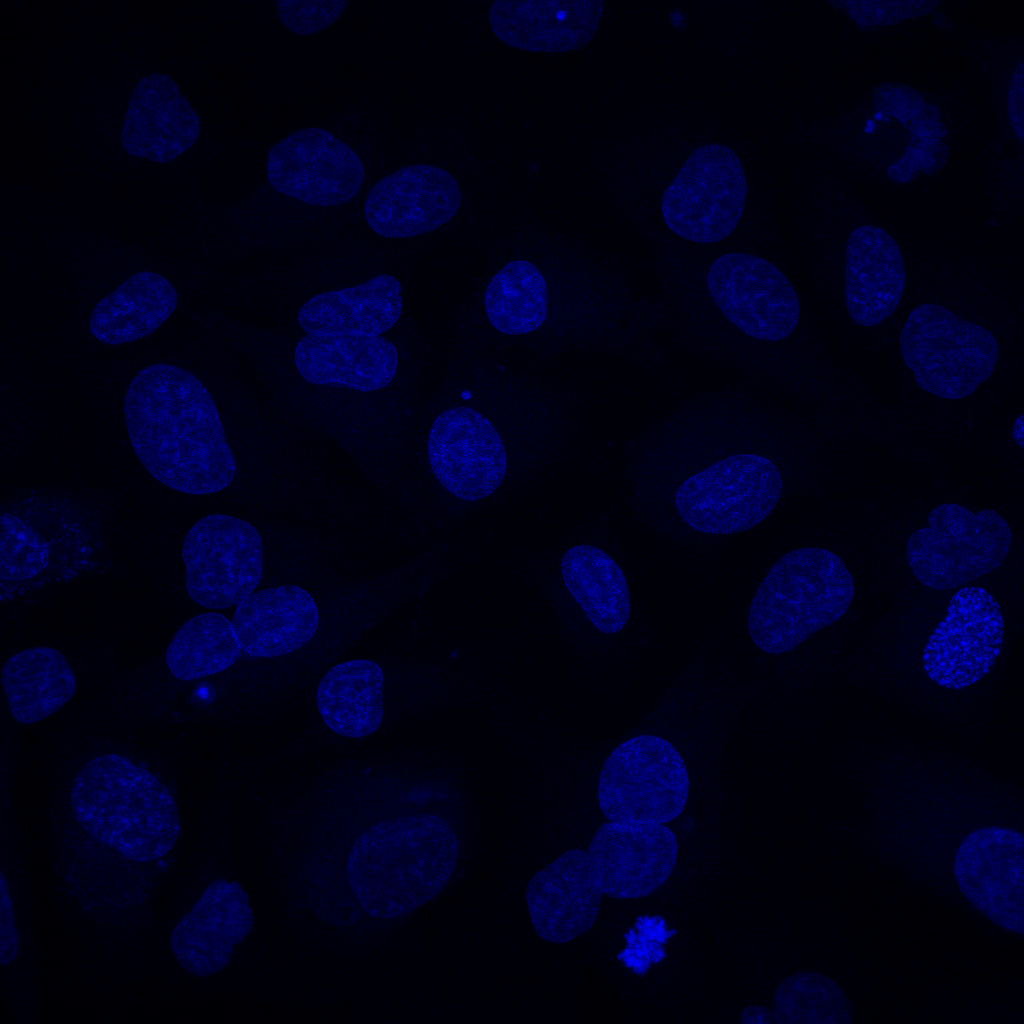

Supplement: Soure data 4. [file elife-32490-fig4.zip › Figure 5- figure supplement 1/Panel c/WM793/WM793_totalITGB1_594_Max_c2.tif]

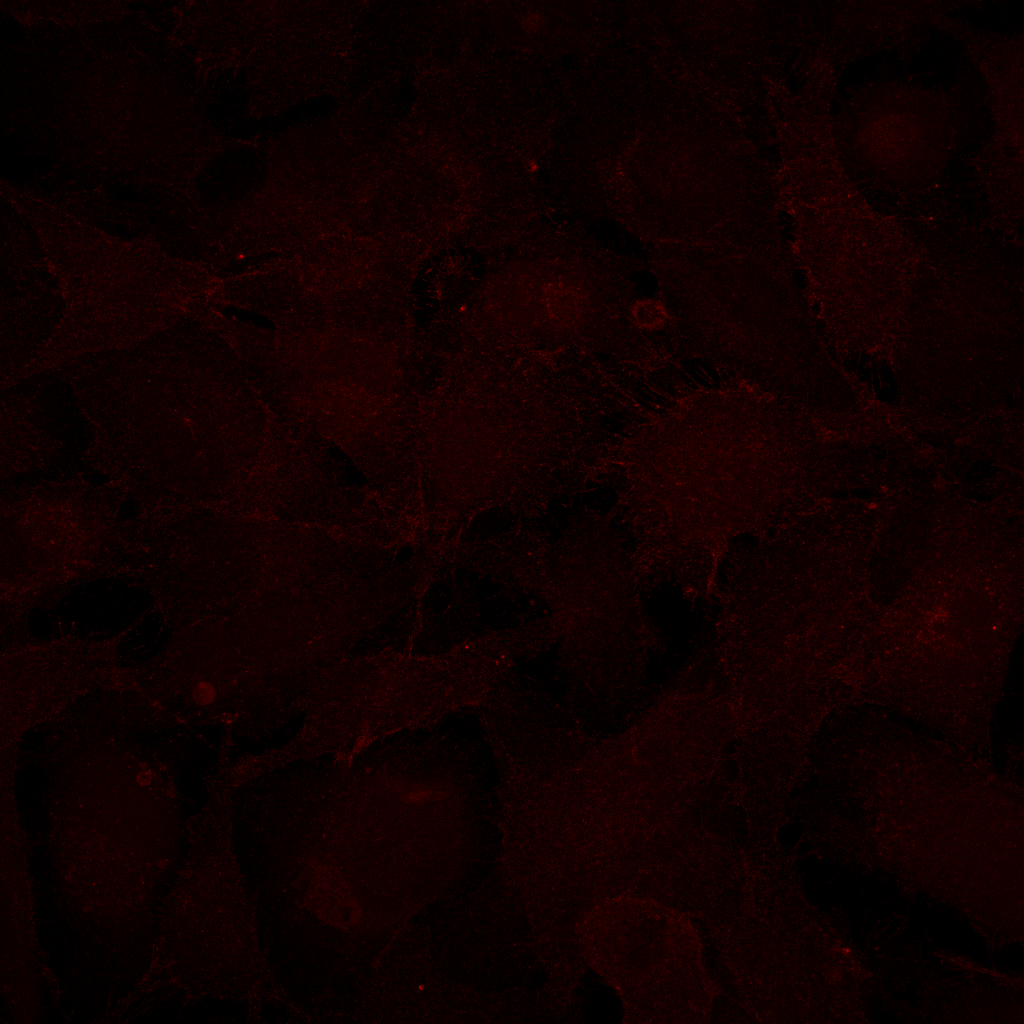

Supplement: Soure data 4. [file elife-32490-fig4.zip › Figure 5- figure supplement 1/Panel c/WM793/WM793_totalITGB1_594_Max_c3.tif]

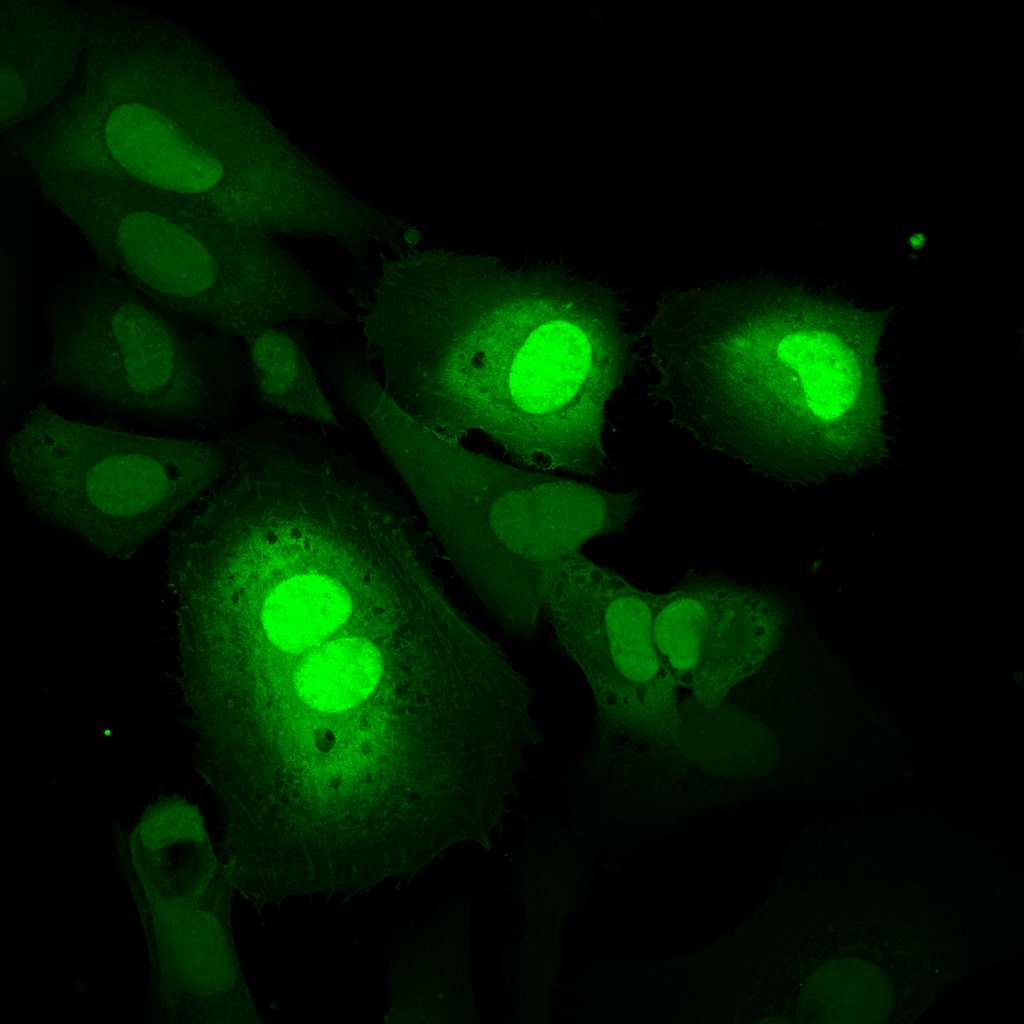

Supplement: Soure data 4. [file elife-32490-fig4.zip › Figure 5- figure supplement 1/Panel c/WM793_LEC/LEC_WM793_totalITGB1_594_Max_c1.tif]

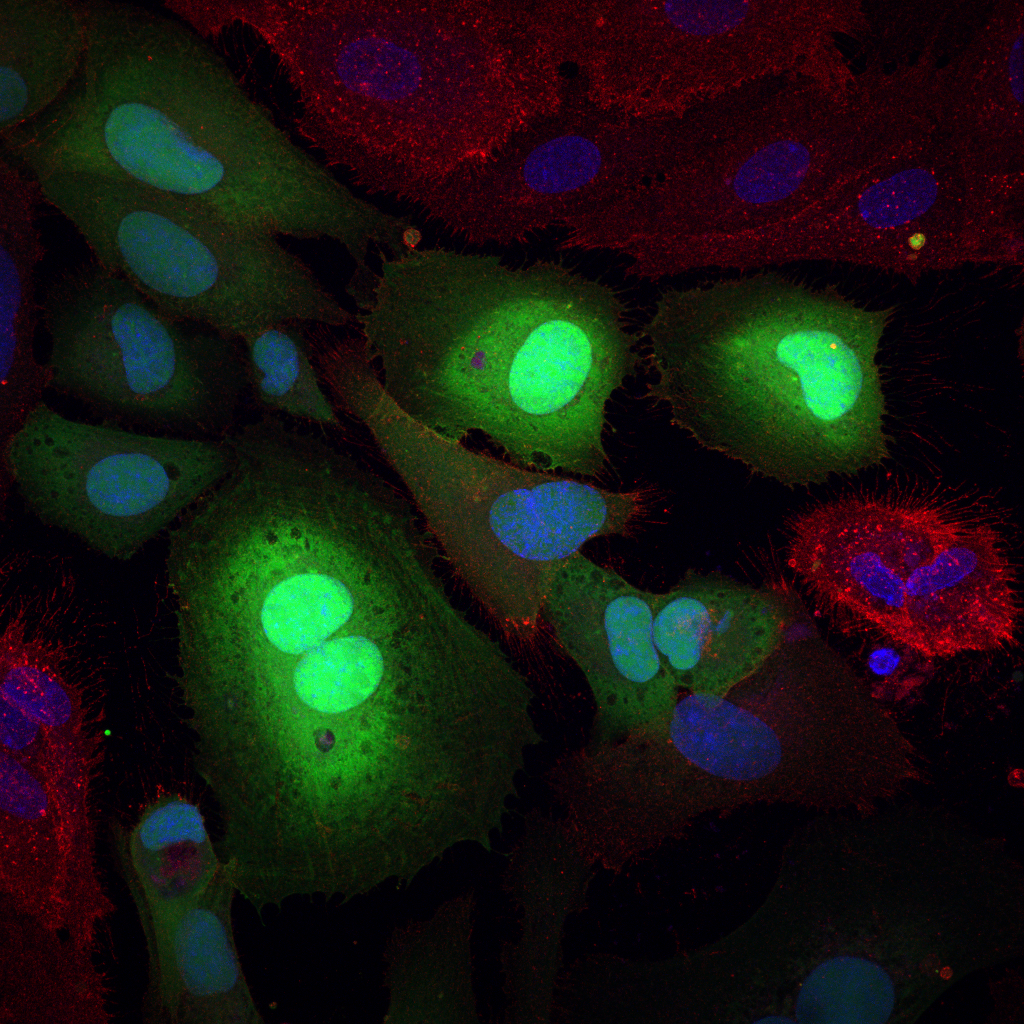

Supplement: Soure data 4. [file elife-32490-fig4.zip › Figure 5- figure supplement 1/Panel c/WM793_LEC/LEC_WM793_totalITGB1_594_Max_c1+2+3.tif]

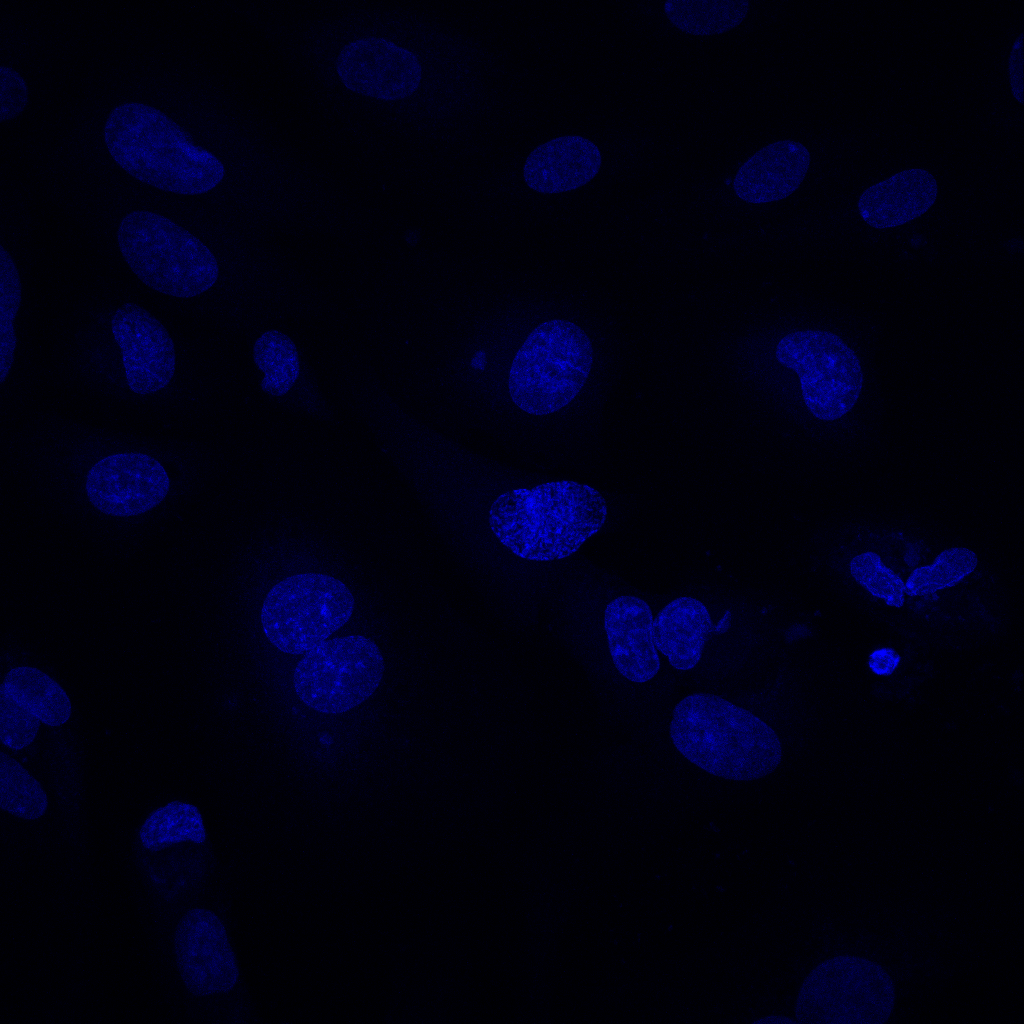

Supplement: Soure data 4. [file elife-32490-fig4.zip › Figure 5- figure supplement 1/Panel c/WM793_LEC/LEC_WM793_totalITGB1_594_Max_c2.tif]

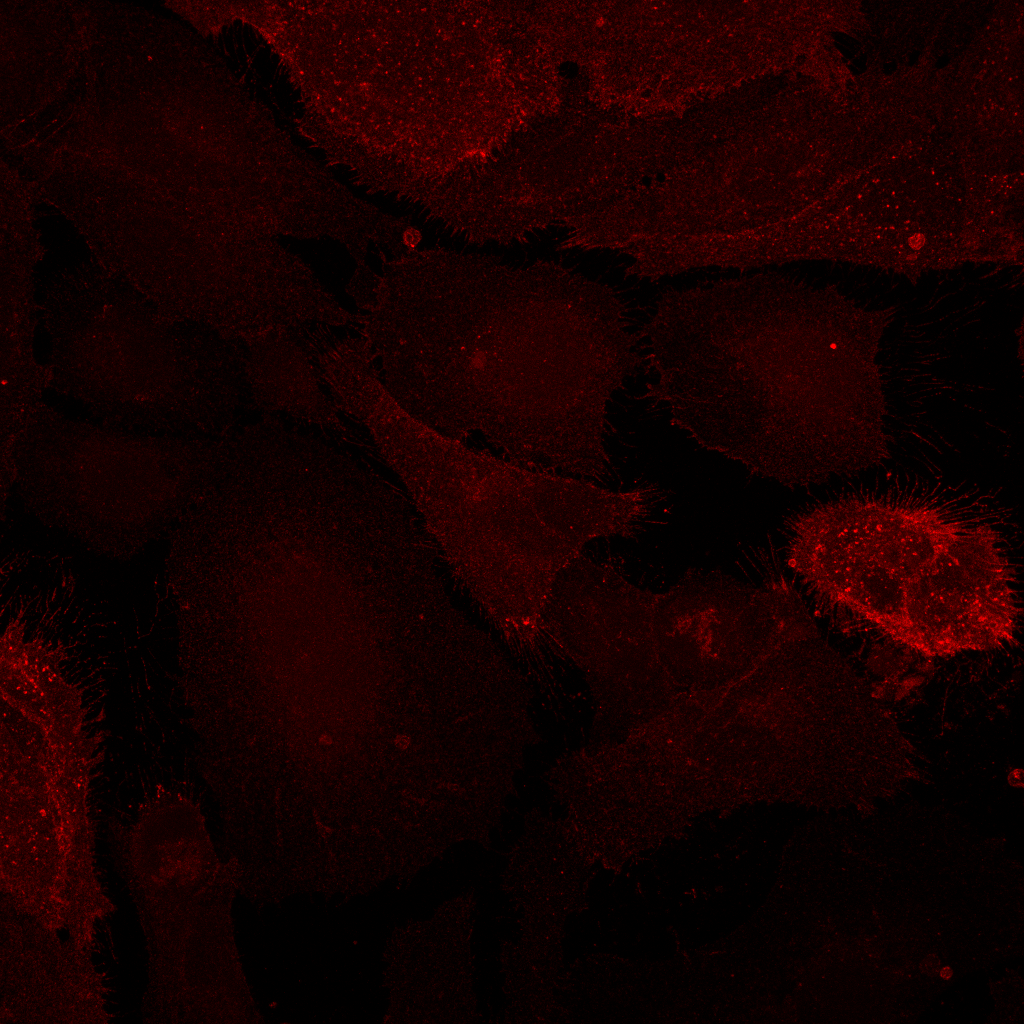

Supplement: Soure data 4. [file elife-32490-fig4.zip › Figure 5- figure supplement 1/Panel c/WM793_LEC/LEC_WM793_totalITGB1_594_Max_c3.tif]

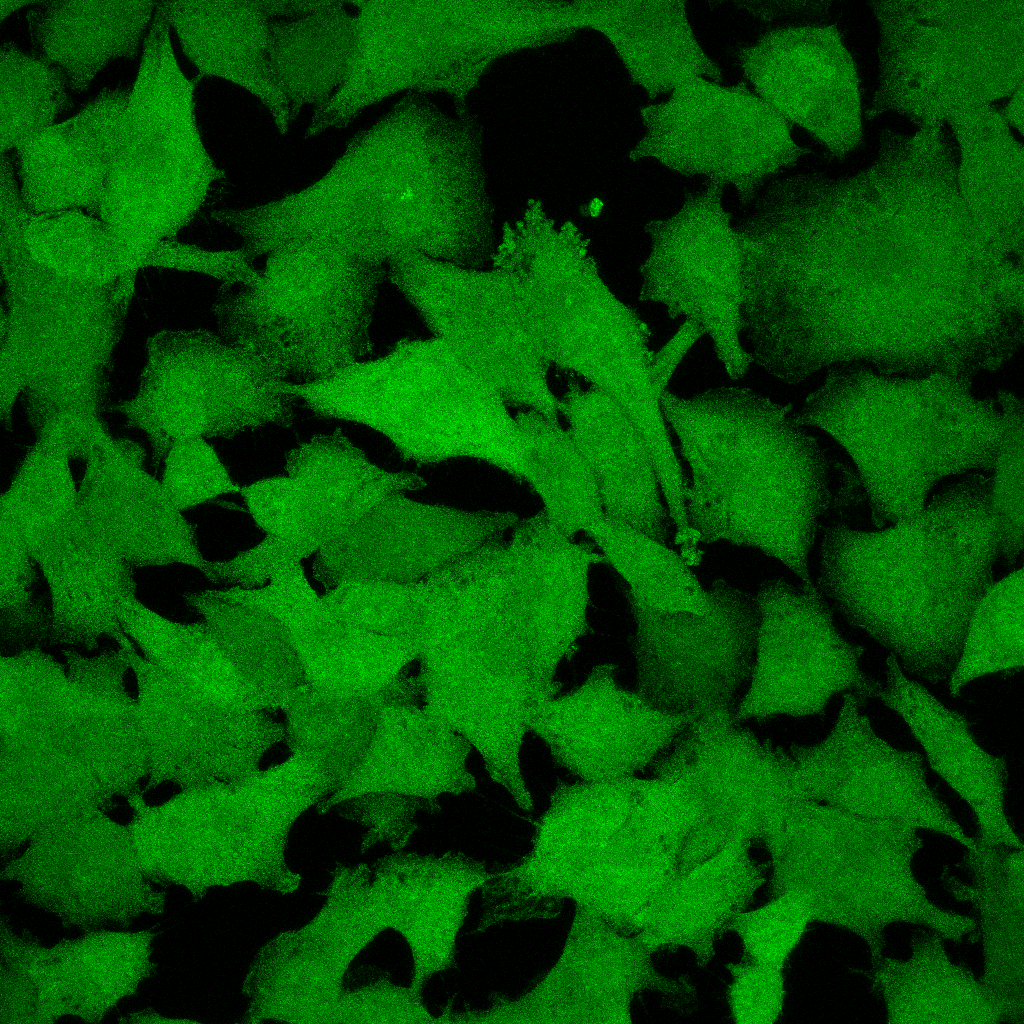

Supplement: Soure data 4. [file elife-32490-fig4.zip › Figure 5- figure supplement 1/Panel c/WM852/WM852_totalITGB1_594_Maximum intensity projection_c1.tif]

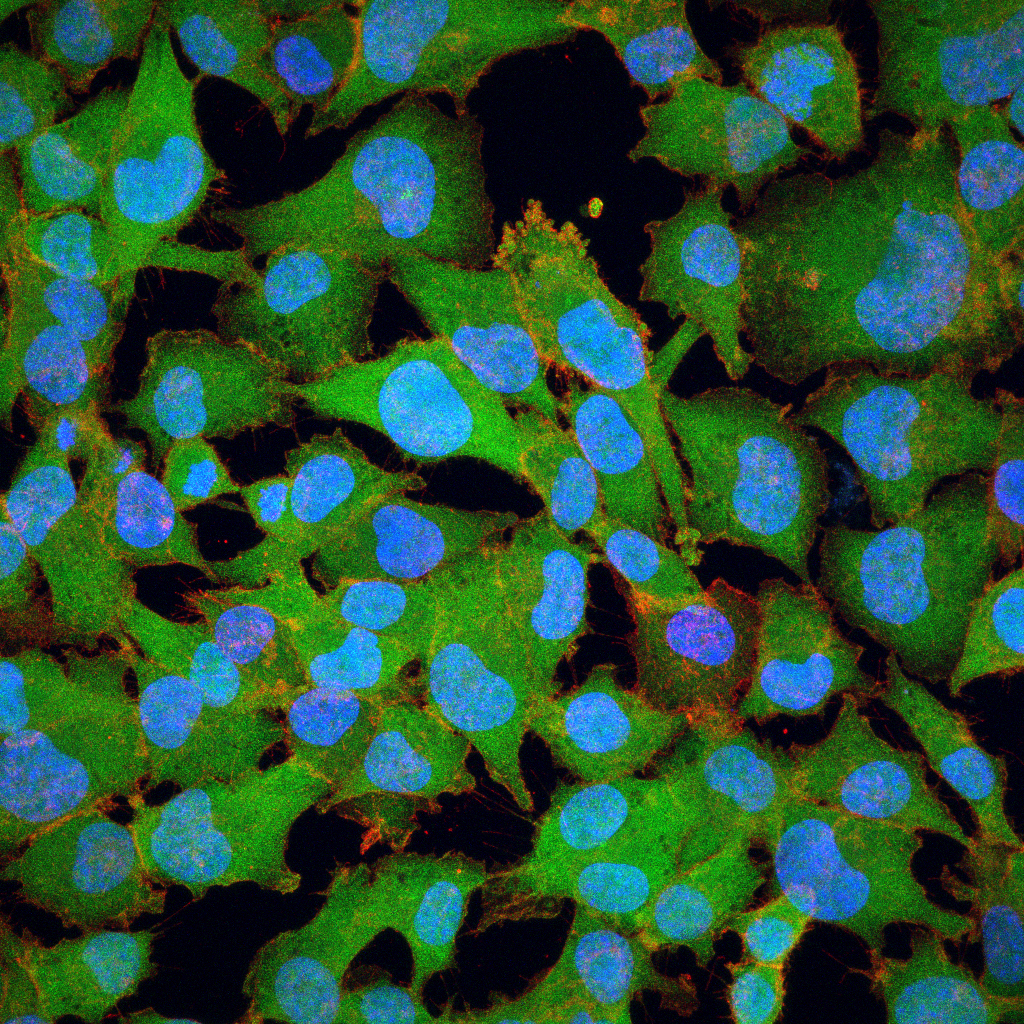

Supplement: Soure data 4. [file elife-32490-fig4.zip › Figure 5- figure supplement 1/Panel c/WM852/WM852_totalITGB1_594_Maximum intensity projection_c1+2+3.tif]

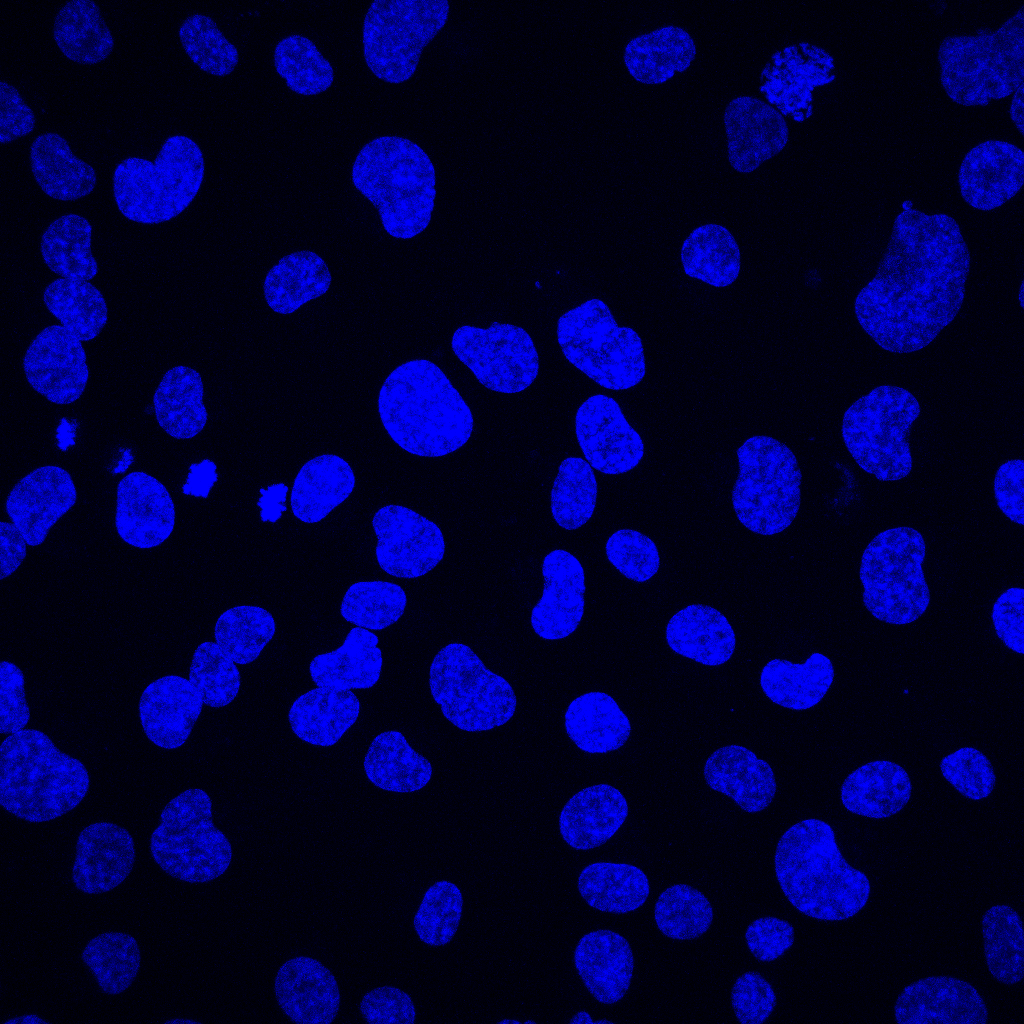

Supplement: Soure data 4. [file elife-32490-fig4.zip › Figure 5- figure supplement 1/Panel c/WM852/WM852_totalITGB1_594_Maximum intensity projection_c2.tif]

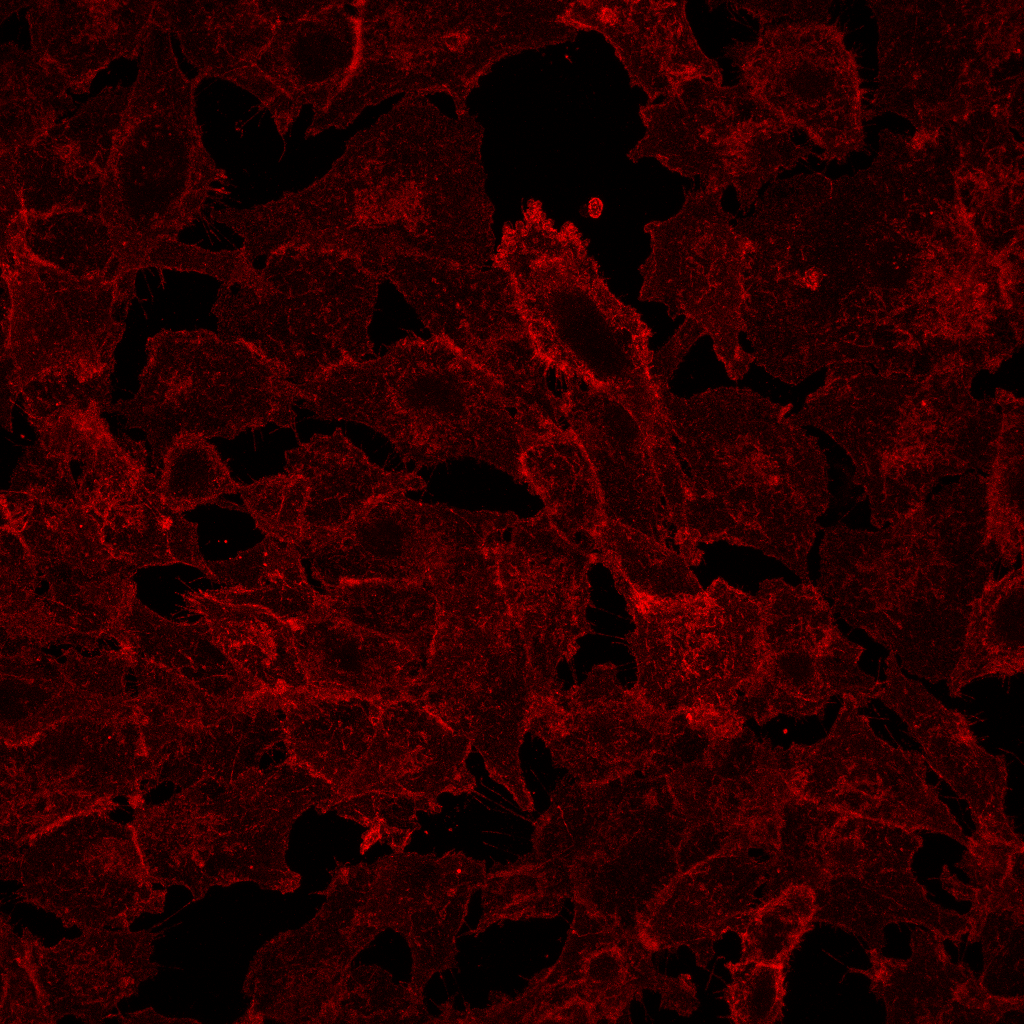

Supplement: Soure data 4. [file elife-32490-fig4.zip › Figure 5- figure supplement 1/Panel c/WM852/WM852_totalITGB1_594_Maximum intensity projection_c3.tif]

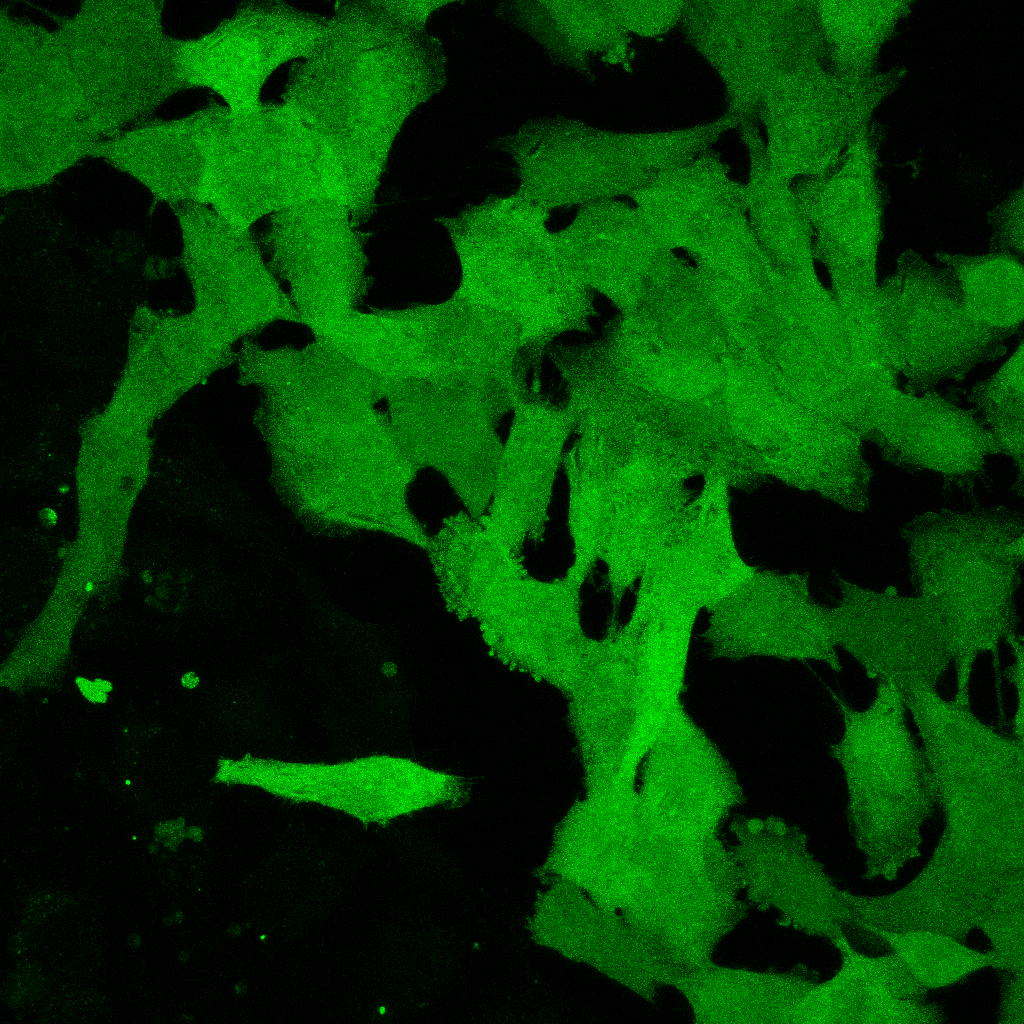

Supplement: Soure data 4. [file elife-32490-fig4.zip › Figure 5- figure supplement 1/Panel c/WM852_LEC/LEC_WM852_totalITGB1_594_Maximum intensity projection_c1.tif]

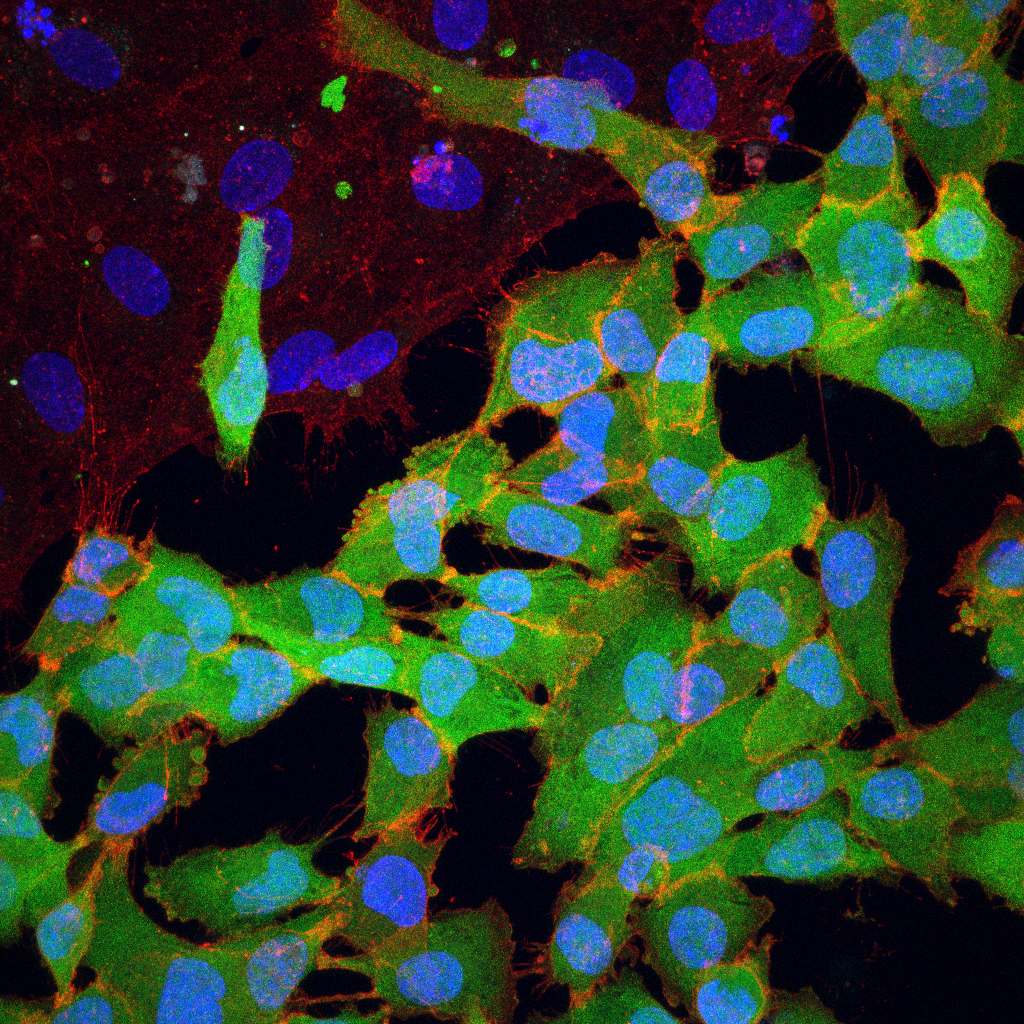

Supplement: Soure data 4. [file elife-32490-fig4.zip › Figure 5- figure supplement 1/Panel c/WM852_LEC/LEC_WM852_totalITGB1_594_Maximum intensity projection_c1+2+3.tif]

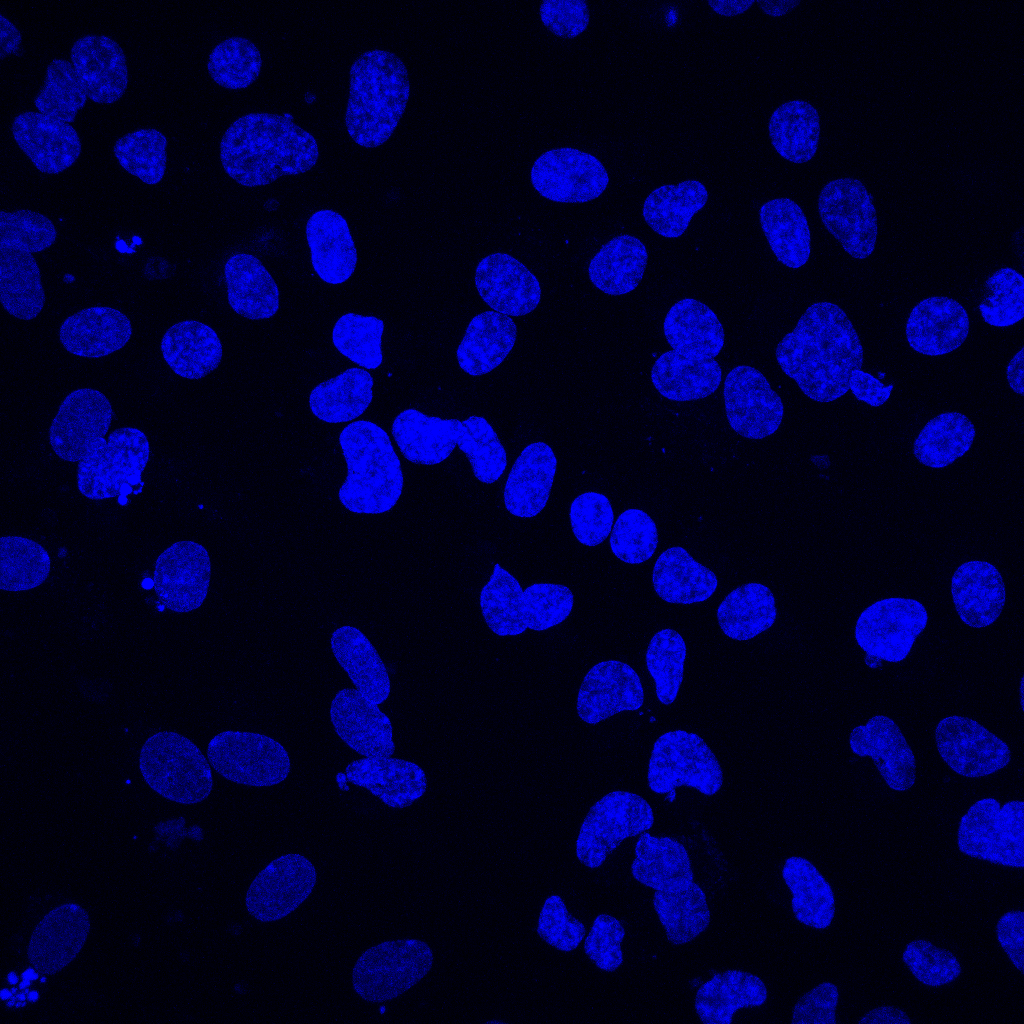

Supplement: Soure data 4. [file elife-32490-fig4.zip › Figure 5- figure supplement 1/Panel c/WM852_LEC/LEC_WM852_totalITGB1_594_Maximum intensity projection_c2.tif]

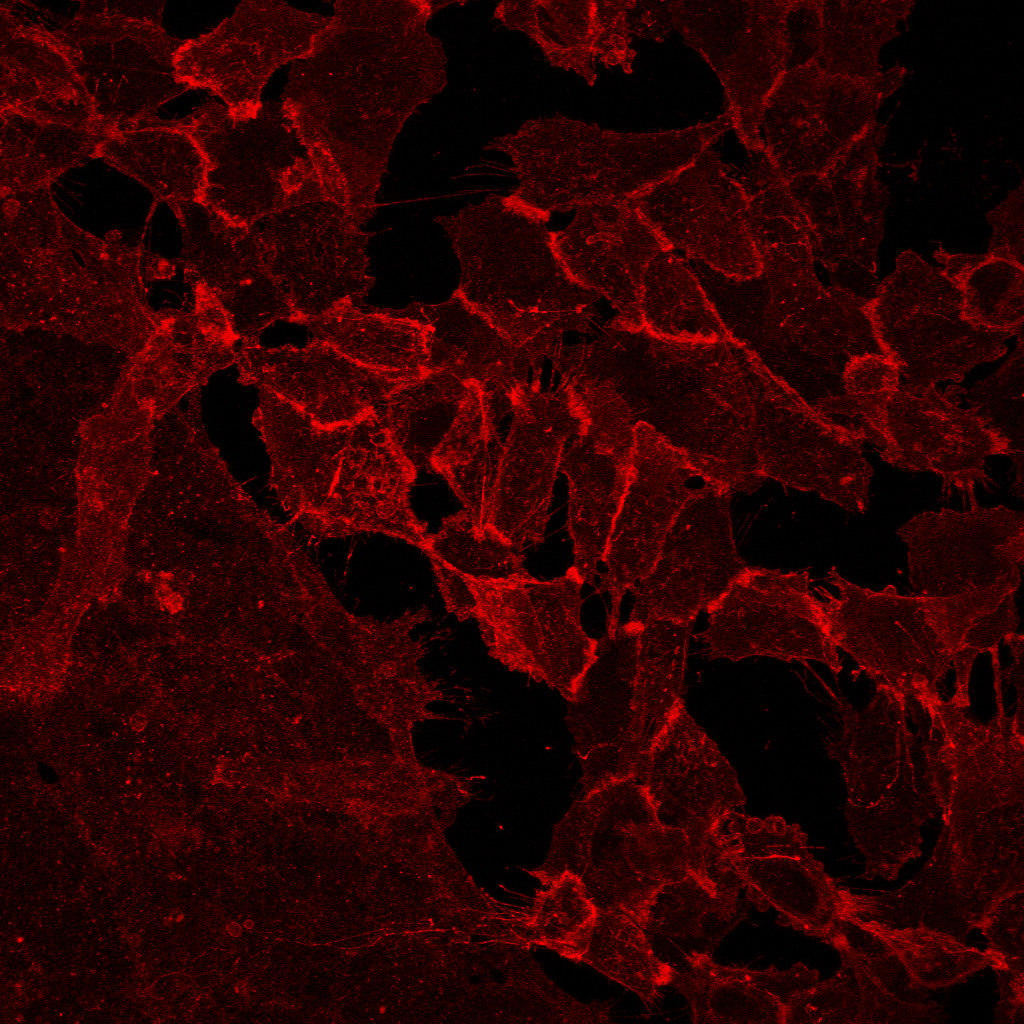

Supplement: Soure data 4. [file elife-32490-fig4.zip › Figure 5- figure supplement 1/Panel c/WM852_LEC/LEC_WM852_totalITGB1_594_Maximum intensity projection_c3.tif]
